# Photocatalytic C–H silylation of heteroarenes by using trialkylhydrosilanes†
†Electronic supplementary information (ESI) available: Tables S1–S6, Scheme S1–S8, Fig. S1–S4, experimental procedures and characterization for all of the new compounds. See DOI: 10.1039/c9sc00046a


**DOI:** 10.1039/c9sc00046a

**Published:** 2019-02-18

**Authors:** Shihui Liu, Peng Pan, Huaqiang Fan, Hao Li, Wei Wang, Yongqiang Zhang

**Affiliations:** a State Key Laboratory of Bioengineering Reactor , Shanghai Key Laboratory of New Drug Design and School of Pharmacy , East China University of Science and Technology , Shanghai 200237 , P. R. China . Email: yongqiangzhang@ecust.edu.cn ; Email: wwang@pharmacy.arizona.edu; b Department of Pharmacology and Toxicology , BIO5 Institute , University of Arizona , Tucson , Arizona 85721-0207 , USA

## Abstract

The efficient and selective C–H silylation of heteroarenes, especially the pharmaceutically relevant electron-deficient heteroarenes, represents a great challenge in organic synthesis. Herein we wish to report a distinctive visible light-promoted photocatalytic C–H silylation approach that enables the direct coupling of trialkylhydrosilanes with both electron-deficient and -rich heteroarenes as well as with cyano-substituted arenes in moderate to high yields and with good regioselectivity. The protocol features operational simplicity, mild reaction conditions, and the use of safe and readily available Na_2_S_2_O_8_, bis(trimethylsilyl) peroxide (BTMSPO) or ^i^Pr_3_SiSH as the radical initiators. Notably, the challenging bulky and inert trialkylhydrosilanes, such as (*t*-butyldimethyl)silane (^*t*^BuMe_2_SiH) and (triisopropyl)silane (^i^Pr_3_SiH), work smoothly with the protocol. Moreover, despite the higher stability of ^*t*^BuMe_2_Si silylation products, our studies revealed their great reactivity and versatility in diverse C–Si-based chemical transformations, providing an operationally simple, low-cost, and environmentally benign synthetic technology for molecule construction and elaboration.

## Abstract 2

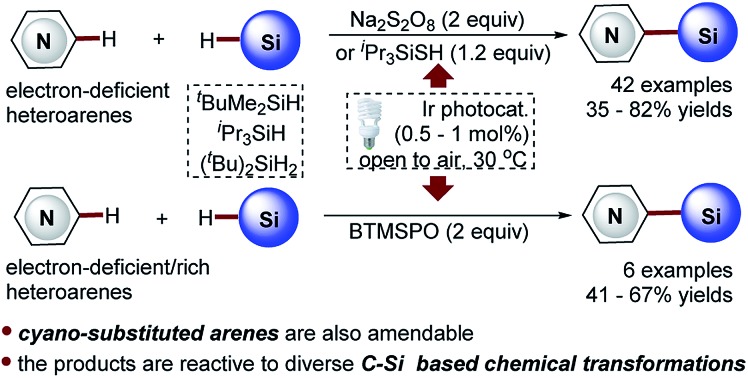
A distinctive visible light-promoted photocatalytic approach for the C–H silylation of heteroarenes by using trialkylhydrosilanes was developed.

## Introduction

**Scheme 1:**
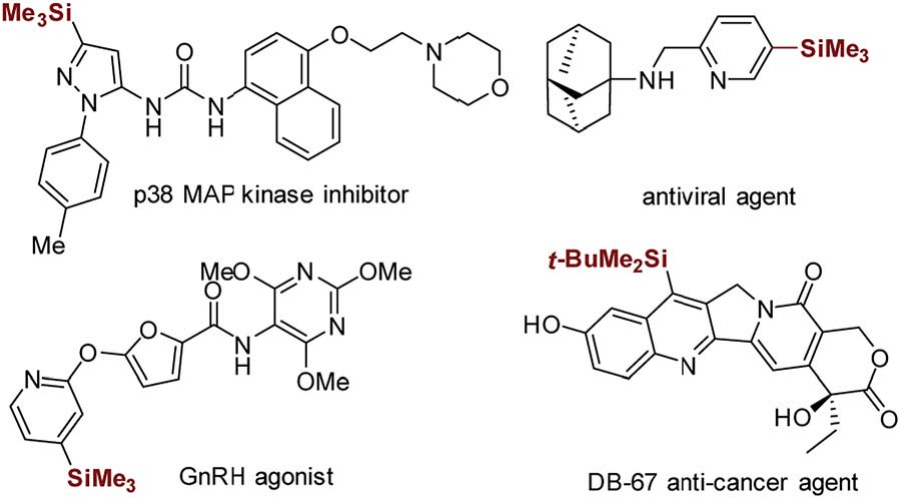
Selected bioactive compounds that contain heteroaryltrialkylsilane functionality.

Heteroaryltrialkylsilanes, especially the electron-deficient heteroaryltrialkylsilanes, represent a class of promising therapeutic agents with interesting biological properties (see the representative examples shown in Scheme 1).^1^–^4^ Moreover, these organosilicon functionalities serve as versatile heteroaryl handles for complex molecule synthesis owing to their high air and moisture stability, low toxicity, and ease of manipulation.^5^–^9^

The direct coupling of readily available and unfunctionalized heteroarenes with simple trialkylhydrosilanes offers a step-efficient and atom-economical synthetic tool to access this class of valuable targets. The quest for such synthetic methodologies underscores significant efforts leading to elegant and powerful catalytic C–H silylation approaches, including the transition-metal-catalyzed approach,^10^–^14^ Friedel–Crafts-type reaction,^15^–^18^ and a rather unorthodox method promoted by KO^*t*^Bu.^19^–^21^ However, these methods often rely on the use of precious iridium or ruthenium species at high temperature (80–135 °C) or the use of extremely strong Lewis acids or bases under strict water- and oxygen-free reaction conditions. Moreover, these methods are only compatible with electron-rich heteroarenes and small trialkylhydrosilanes, such as triethylhydrosilane (Et_3_SiH) and diethylmethylhydrosilane (Et_2_MeSiH). These drawbacks have significantly restricted the synthetic application of these methods, especially in the pharmaceutical sector.

**Scheme 2:**
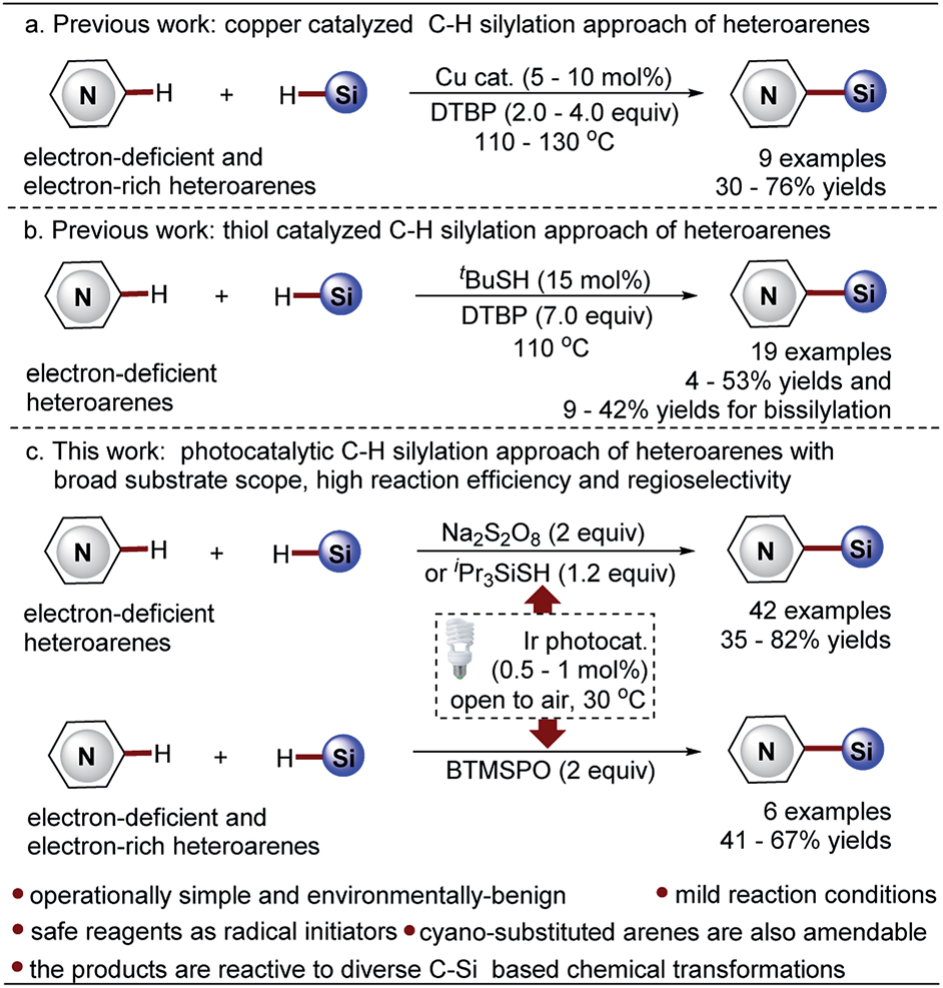
Minisci-type approaches for C–H silylation of heteroarenes.

Minisci-type C–H silylation offers a better solution (Scheme 2).^22^–^26^ The copper-catalyzed approach enables the introduction of trialkylsilyl groups to both electron-rich and -deficient heteroarenes in synthetically useful yields (30–76%) (Scheme 2a).^24^,^25^ It is noted that the electron-deficient heteroarenes display inferior reaction efficiency (32–40% yields) and only two pyridine examples were demonstrated. Furthermore, this process is carried out at a high reaction temperature (130 °C) in the presence of explosive peroxide (di-*tert*-butyl peroxide, DTBP), which causes significant safety concerns. Recently, inspired by the pioneering work of the Curran group,^4^ Maruoka and co-workers disclosed an interesting thiol-catalyzed approach for the C–H silylation of electron-deficient heteroarenes, such as pyridines, quinolones, pyrazines and quinoxalines, which could give the mono-silylation products in low yields (4–53%) accompanied with the formation of a significant amount of bis-silylation products (9–42% yield) (Scheme 2b).^26^ Notably, the *in situ* generated silyl radicals are highly reactive and non-clustered,^27^,^28^ thus enabling the construction of sterically demanding C–Si bonds and the synthesis of heteroaryltrialkylsilanes with bulky trialkylsilyl groups, such as *t*-butyldimethylsilyl and triisopropylsilyl groups. Nevertheless, the process is also performed at high temperature (110 °C) using a large excess of DTBP (7.0 equiv.), which causes significant safety concerns, as well as the difficulty controlling regioselectivity. Therefore, in this field, there is an unmet synthetic challenge for the development of a mild and truly efficient approach for the selective C–H silylation of heteroarenes, especially the pharmaceutically relevant electron-deficient heteroarenes. Such a method enabling the incorporation of the challenging bulky trialkylsilyl functionalities into heteroarenes will streamline the synthesis of synthetically and medicinally valuable heteroaryltrialkylsilanes, such as the anticancer drug candidate camptothecin derivative DB-67 (Scheme 1).^4^

Towards this end, we wish to report a distinctive visible light-promoted photocatalytic Minisci-type approach that enables the efficient and selective C–H silylation of both electron-deficient and -rich heteroarenes in moderate to high yields and with good regioselectivity by using trialkylhydrosilanes (Scheme 2c). The protocol features operational simplicity, mild reaction conditions, and the use of a significantly reduced amount of Na_2_S_2_O_8_ (2.0 equiv.), bis(trimethylsilyl)-peroxide (BTMSPO) (2.0 equiv.) or an alternative safe ^i^Pr_3_SiSH (1.2 equiv.) as the radical initiators. This study, therefore, provides a toolbox of powerful solutions for the synthesis of structurally diverse heteroaryltrialkylsilanes. While Na_2_S_2_O_8_ and ^i^Pr_3_SiSH serve as efficient promoters for the C–H silylation of electron-deficient heteroarenes, BTMSPO was proved effective for both electron-deficient and electron-rich heteroarenes as a more promising radical initiator. It is of note that the thiol-mediated sila-Minisci-type reaction is carried out under peroxide-free conditions with superior regioselectivity. The cyano-substituted arenes were also amenable to this method. Notably, the challenging bulky and inert trialkylhydrosilanes, such as (*t*-butyldimethyl)silane (^*t*^BuMe_2_SiH) and (triisopropyl)silane (^i^Pr_3_SiH), work smoothly with the protocol. Moreover, despite the higher stability of ^*t*^BuMe_2_Si silylation products, our studies revealed their great reactivity and versatility in diverse C–Si-based chemical transformations, providing an operationally simple, low-cost, and environmentally benign synthetic technology for molecule construction and elaboration.

## Results and discussion

Photocatalytic Minisci-type reactions for the construction of heteroaryl C–C bonds from two different and inert C–H bonds have been well developed.^29^–^31^ We questioned whether this strategy could be extended for the formation of heteroaryl C–Si bonds. This process is generally carried out under mild reaction conditions, which may provide an opportunity to address the issue of regioselectivity and minimize the production of undesired bis-silylation products in the present Minisci-type C–H silylation methods.^24^–^26^

### Optimization of reaction conditions

The initial investigation of the proposed photocatalytic C–H silylation reaction started by subjecting 6-methylisoquinoline **1a** and *t*-butyldimethylsilane (^*t*^BuMe_2_SiH) **2a** to the benzaldehyde-mediated photoredox reaction conditions established in our previous study using Na_2_S_2_O_8_ as the radical initiator.^31^ To our delight, the desired product was obtained in a tractable yield (20%). This outcome inspired us to systematically investigate the interesting reaction. Extensive reaction condition screening, including the screening of photocatalysts (Table S1†), the screening of oxidants (Table S2†), the screening of solvents (Table S3†), and the screening of the amount of **2a** (Table S4†), identified the optimal conditions as follows: in the presence of 23 W CFL, 1 mol% of [Ir(ppy)_2_(dtbbpy)]PF_6_, and 2 equiv. of Na_2_S_2_O_8_, the reaction of 6-methylisoquinoline **1a** with 5 equiv. of ^*t*^BuMe_2_SiH **2a** is conducted in a solvent mixture of DMSO : DCE = 1 : 1 (0.1 M) for 24 h at 30 °C (Table 1, entry 1). Under the optimal reaction conditions, the mono-silylation product **3a** was produced in high yield (77%, entry 1, Table 1). Nevertheless, a very small amount of the bis-silylation product **3a′** was also observed (**3a** : **3a′** = 15 : 1). The decrease of the amount of ^*t*^BuMe_2_SiH **2a** (3 equiv.) was detrimental to the reaction efficiency, while the regioselectivity remained unaffected (69% yield, **3a** : **3a′** = 14 : 1, entry 2, Table 1). Employing trifluoroacetic acid (TFA)^32^,^33^ as an additive or a stronger 34 W LED as an alternative visible light source resulted in slightly decreased yields and regioselectivity (70–74% yields, **3a** : **3a′** = 9 : 1 to 12 : 1, entries 3–4, Table 1). The use of thiol as an additive in this process, which was shown an efficient promotor in sila-Minisci-type reactions,^4^,^26^ had little effect on the reaction outcome (77% yield, **3a** : **3a′** = 13 : 1, entry 5, Table 1). Interestingly, the removing of Na_2_S_2_O_8_ in the presence of thiol was also able to produce **3a** in a synthetically useful yield with better regioselectivity (27% yield, **3a** only, entry 6, Table 1). This study offers the possibility of developing a peroxide-free photocatalytic Minisci-type C–H silylation reaction with better site selectivity. Oxygen is not essential for this process (76–77% yields, entries 7–8, Table 1). Control experiments show that visible light, photocatalyst, and Na_2_S_2_O_8_ are essential for this process, again suggesting the visible-light-driven photochemical nature of this reaction (entries 9–11, Table 1).

**Table 1:** Optimization and validation of the reaction conditions^*a*^

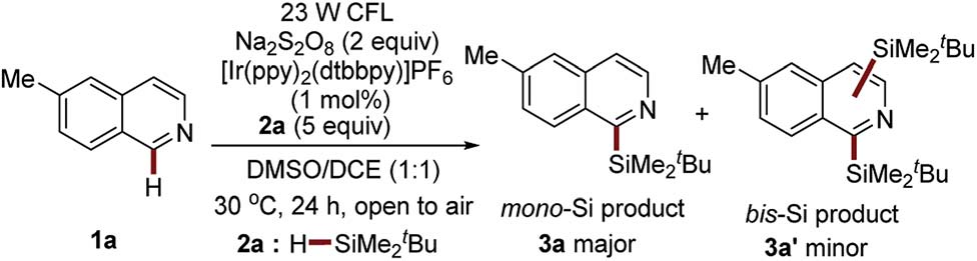			
Entry	Reaction conditions	Yield (**3a**)^*b*^	Ratio (**3a** : **3a′**)^*c*^
1	Standard conditions	77%	15 : 1
2	**2a** (3 equiv.)	69%	14 : 1
3	TFA^*d*^ (1.0 equiv.)	70%	9 : 1
4	34 W blue LED	74%	12 : 1
5	^*t*^BuSH (20 mol%)	77%	13 : 1
6	^*t*^BuSH (20 mol%)^*e*^	27%	**3a** only
7	Under O_2_ (1 atm)	76%	13 : 1
8	Under N_2_ (1 atm)^*f*^	77%	15 : 1
9	In darkness	n.d.^*g*^	—
10	No photocat.	16%	—
11	No Na_2_S_2_O_8_	n.d.^*g*^	—

^*a*^Standard reaction conditions: 23 W CFL, **1a** (0.5 mmol), **2a** (2.5 mmol), photocatalyst (1 mol%), Na_2_S_2_O_8_ (1.0 mmol), solvent mixture (5.0 mL, DMSO : DCE = 1 : 1), air, 30 °C, 24 h, unless otherwise noted.

^*b*^Isolated yields were reported.

^*c*^Regiomeric ratio (r.r.) determined using ^1^H NMR spectroscopy.

^*d*^TFA, trifluoroacetic acid.

^*e*^Performed in the absence of Na_2_S_2_O_8_.

^*f*^The reaction mixture was degassed *via* freeze–pump–thaw (three times) and refilled with N_2_.

^*g*^Not detected.

### Reaction scope

#### The substrate scope of the Na_2_S_2_O_8_-mediated photocatalytic sila-Minisci-type reaction

**Scheme 3:**
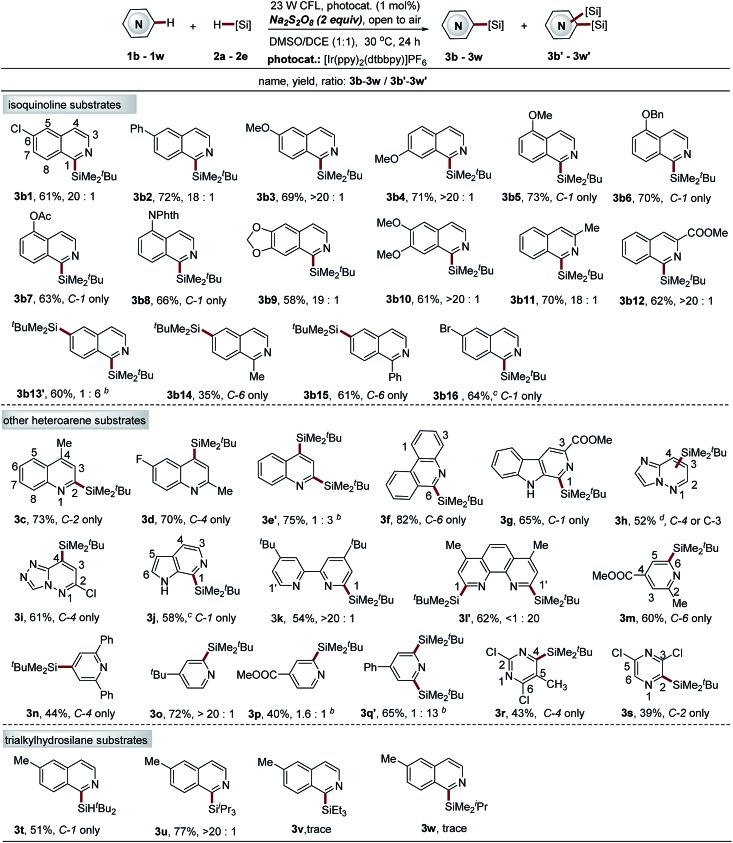
Direct C–H silylation of diverse electron-deficient heteroarenes *via* Na_2_S_2_O_8_-mediated photocatalytic Minisci-type reaction. ^*a*^See general procedure A for the experimental details unless otherwise noted; isolated yields are reported. ^*b*^Regiomeric ratio (r.r.) determined using ^1^H NMR spectroscopy; **3b13′**, mono-Si (C-1) : bis-Si (C-1 and C-6) = 1 : 6 (r.r.); **3e′**, mono-Si product (C-2) : bis-Si product (C-2 and C-4) = 1 : 3 (r.r.); **3p**, mono-Si product (C-2) : bis-Si product (C-2 and C-5) = 1.6 : 1 (r.r.); **3q′**, mono-Si product (C-2) : bis-Si product (C-2 and C-6) = 1 : 13 (r.r.). ^*c*^The thiol-mediated C–H silylation approach was employed; isolated yields are reported; please see general procedure B for the experimental details. ^*d*^C-4 product : C-3 product = 2 : 1.

Having established the optimal reaction conditions, we then examined the substrate scope of the process. Structurally diverse electron-deficient heteroarenes could undergo C–H silylation with trialkylhydrosilanes in moderate to high yields and regioselectivity (Scheme 3). However, the electron-rich heteroarenes, such as indole, benzofuran and benzothiophene, are not amenable to this protocol (data not shown here). The reactions proceeded well for the isoquinoline substrates with both electron-withdrawing and electron-donating groups, providing the products **3b1–b12** in moderate to high yields (58–73%) and excellent site selectivity (C-1 position). In particular, 6,7-dimethoxyisoquinoline and isoquinoline fused with a 1,3-dioxolane at the 6 and 7 positions, which are highly sensitive to oxidation, proved to be effective reactants (**3b9–b10**, 58–61% yields). A variety of functional groups, such as chlorine, ester, cyano, protected hydroxyl and amino, were well tolerated, providing handles for further synthetic elaboration. Notably, many HAT-sensitive (hydrogen atom transfer) C–H bonds, especially those C–H bonds α to oxygen (CH_3_O–, PhCH_2_O–, –O–CH_2_–O–), were also endurable, suggesting the excellent Si–H bond selectivity of this protocol. However, the isoquinoline without any substituted groups displayed poor regioselectivity (the second silyl group tends to be installed at the C-6 position), producing the bis-silylation product (C-1 and C-6 positions) in moderate yield (**3b13′**, 60%, **3b13′** : **3b13** = 6 : 1). We developed a new alternative thiol-mediated photocatalytic C–H silylation reaction with better regioselectivity to solve this issue (see Scheme 5). Interestingly, blocking the C-1 position of isoquinoline mainly results in the formation of C-6 silylation products, albeit with slightly decreased reaction efficiency (**3b14–b15**, 35–61% yields). Furthermore, 6-bromoisoquinoline, which is not tolerated with this protocol, was also demonstrated as a suitable substrate of the thiol-mediated reaction, providing the exclusive mono-silylation product in good yield (**3b16**, 64%).

Having demonstrated the utility of this protocol in the installation of the bulky *t*-butyldimethylsilyl group onto isoquinoline structures, we next examined other pharmaceutically relevant electron-deficient heteroarenes. The quinolines with substituted groups at the 2 or 4 positions worked smoothly, giving the mono-silylation products in high yields (**3c–3d**, 70–73%). However, the quinoline without any substituted groups also displayed poor regioselectivity accompanied with the formation of a significant amount of bis-silylation product (C-2 and C-4 positions) (**3e′**, 75% yield, **3e′** : **3e** = 3 : 1). Phenanthridine, β-carboline, as well as the pyridine structures fused with five-membered nitrogen-containing heteroarenes, such as imidazole and 1,2,4-triazole, also proved to be effective reactants (**3f–3i**, 52–82% yields), while a poor result was observed for 6-azaindole (pyridine structure fused with pyrrole). Nonetheless, the thiol-mediated approach is compatible with this substrate, albeit with slightly decreased yield (**3j**, 58%). Notably, the silyl group was installed at the pyridine ring with excellent site selectivity (C-1 position), which provides a complementary C–H silylation approach to that of Grubbs's, where a silyl group is incorporated on the pyrrole ring of 6-azaindole (C-5 position).^19^ Widely used ligand molecules, including 2,2′-bipyridine and 1,10-phenanthroline, were also identified as suitable substrates for this protocol. The mono-silylation product at the C-1 position was observed for 2,2′-bipyridine (**3k**, 54% yield), while 1,10-phenanthroline is highly reactive, providing the bis-silylation product (**3l′**, C-1 and C-1′ positions, 62% yield). The thiol-mediated approach cannot improve the regioselectivity (data not shown here). In combination with diverse C–Si-based transformations, this protocol holds great potential for ligand modification. Various pyridine substrates also worked well to give the mono-silylation products at the most electrophilic sites (C-2, C-4, or C-6 position) (**3m–3p**, 40–72% yields). However, the introduction of a phenyl group at the C-4 position of pyridine simultaneously activates both C-2 and C-6 positions, thus mainly giving the bis-silylation product (**3q′**, 65% yield, **3q** : **3q′** = 1 : 13). The thiol-mediated approach cannot further improve this site selectivity either (data not shown here). The pyrimidine and pyrazine substrates were also amenable but with low yields (**3r–3s**, 39–43%). However, the formation of only trace products was observed for quinazoline, quinoxaline, benzothiazole, benzimidazole and benzoxazole structures (data not shown here), which might be attributed to the instability of the products under the reaction conditions.

Various trialkylsilanes were then examined to further extend the substrate scope. Notably, the large silyl groups, including di-*tert*-butylsilyl (^*t*^Bu_2_SiH–) and triisopropylsilyl (^i^Pr_3_Si–) were readily introduced at the C-1 position of 6-methyl-isoquinoline 1a in moderate to high yields and with excellent site selectivity (**3t–3u**, 51–77%). A limitation is also realized for the protocol. Only trace products were observed for small trialkylsilanes, such as triethylsilane and isopropyldimethylsilane. The thiol-mediated approach was not compatible with these small triethylsilanes either. This result might also be attributed to the instability of the resulting products under the reaction conditions (strong acid and oxidant) (Table S5, ESI†).

**Scheme 4:**
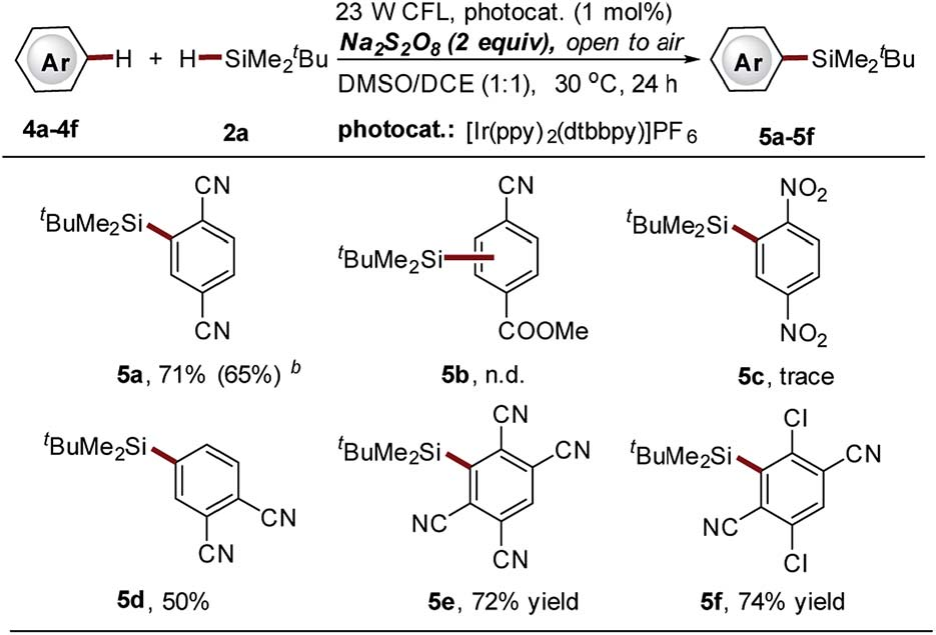
Direct C–H silylation of cyano-substituted benzenes *via* Na_2_S_2_O_8_-mediated photocatalytic Minisci-type reaction. ^*a*^See general procedure A for the experimental details, unless otherwise noted; isolated yields are reported. ^*b*^Thiol-mediated photocatalytic C–H silylation approach was employed; isolated yields are reported; see general procedure B for the experimental details.

The observed regioselective installation of a silyl group onto the benzene ring of isoquinolines (C-6 position, **3b14–b15**, Scheme 3) inspired us to explore the potential of our method in the C–H silylation of non-heteroaromatic substrates. Various substituted benzenes were screened (data not shown here). However, only 1,4-dicyanobenzene was identified as a suitable substrate, giving the mono-silylation product in high yield (**5a**, 71%, Scheme 4). The use of the thiol-mediated approach cannot further improve the reaction (**5a**, 65% yield, Scheme 4) accompanied by the use of more ^*t*^BuMe_2_SiH (10 equiv.). Polycyano substitution was proved essential for this transformation. Good to high yields were observed (**5d–5f**, 50–74% yields, Scheme 4). It should be noted that cyano-substituted benzenes represent a class of frequently used coupling partners in reported photoredox reactions, where the cyano group is directly converted to other functional groups *via* substitution.^34^,^35^ However, the substitution of the cyano group with a silyl group was not observed in this process.

#### Thiol-mediated photocatalytic Minisci-type reaction with better regioselectivity

The poor site selectivity of the Na_2_S_2_O_8_-mediated photocatalytic sila-Minisci-type reaction in the C–H silylation of non-substituted isoquinoline and quinoline triggered us to further explore the thiol-mediated photocatalytic C–H silylation approach (entry 6, Table 1) using isoquinoline and ^*t*^BuMe_2_SiH **2a** as the model substrates (Table S6, ESI†). The careful evaluation of the reaction conditions enabled the identification of the optimized protocol (23 W CFL, 0.5 mol% of Ir(ppy)_3_, 1.2 equiv. of ^i^Pr_3_SiSH, 10 equiv. of ^*t*^BuMe_2_SiH in three batches, DMA/DCE, open to air, 30 °C, 24 h), which displays higher regioselectivity but inferior reaction efficiency (**3b13**, 71% yield, **3b13** : **3b13′** > 20 : 1, Scheme 5A). The same trend was also observed in the C–H silylation of various substituted isoquinoline substrates (Scheme S1†). Meanwhile, the excellent regioselectivity of this approach was further supported by the fact that the silyl group was mainly installed at the C-2 position of quinoline with moderate yield (**3e**, 66%, **3e** : **3e′** = 8 : 1, Scheme 5B). It should be noted that the poor site selectivity and the use of explosive peroxides represent two challenging issues of Minisci-type reactions.^32^,^33^ This thiol-mediated photocatalytic process provides a powerful alternative for the development of peroxide-free Minisci-type reactions with significantly improved regioselectivity.

**Scheme 5:**
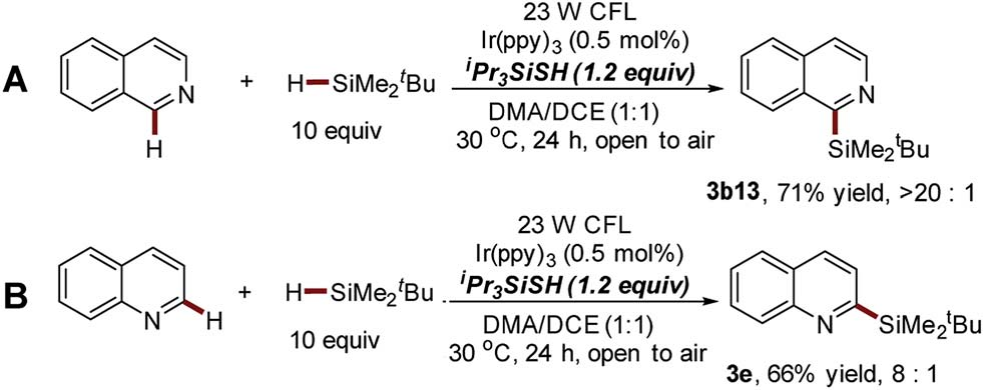
Direct C–H silylation of non-substituted isoquinoline and quinoline *via* thiol-mediated photocatalytic Minisci-type reaction.

### Mechanistic studies

**Scheme 6:**
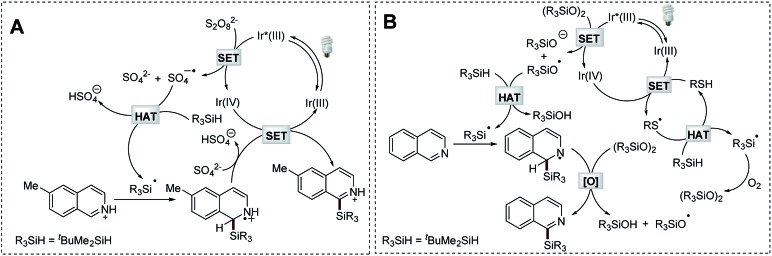
Proposed mechanisms.

To understand the mechanistic nature of the Na_2_S_2_O_8_-mediated photocatalytic Minisci-type C–H silylation reaction, we firstly performed a study to probe and verify the radical engaged process. The formation of silanol and siloxane structures was observed by GC-MS analysis (Fig. S1 and Scheme S2†). This fact and the radical trapping experiments (Scheme S3†) suggest the formation of a silyl radical in the process. Inferior reaction efficiency was observed in the control reactions employing bis(*tert*-butyldimethylsilyl)-peroxide (^*t*^BuMe_2_SiOOSiMe_2_^*t*^Bu) as the radical initiator (43–45% yields, Scheme S4B and 4C†), which might be *in situ* generated in the process. Considering the fact that 2 equiv. of Na_2_S_2_O_8_ was typically required for effective transformation, it is reasonable to conclude that the sulfate radical anion (generated from the photocatalytic decomposition of Na_2_S_2_O_8_) might function as the major driving force to perform HAT of *t*-butyldimethylsilane owing to the polar effect.^34^ Based on these studies and the emission quenching experiment (Fig. S2†), as well as the established mechanism of photocatalytic Minisci-type reaction,^29^,^31^ a plausible reaction mechanism is proposed (Scheme 6A). The photocatalytic decomposition of Na_2_S_2_O_8_ produces a sulfate radical anion, which promotes the formation of a *t*-butyldimethylsilyl radical *via* HAT. The resulting silyl radical undergoes nucleophilic addition with the protonated 6-methylisoquinoline at the C-1 position, followed by deprotonation and oxidation to provide the desired product.

The formation of a silyl radical was also validated in the thiol-mediated photocatalytic Minisci-type C–H silylation reaction through radical trapping and GC-MS experiments (Fig. S3, Scheme S5†). Furthermore, both oxygen and *t*-butyldimethylsilane proved essential for this transformation (Scheme S6B and S6C†). The replacement of thiol with ^*t*^BuMe_2_SiOOSiMe_2_^*t*^Bu, which might be *in situ* generated in this process, provided the product in a comparable yield (62%, Scheme S6D†). Considering the fact that peroxides are typically required in Minisci-type reactions^32^,^33^ and oxygen-centered radical species are able to perform HAT more efficiently,^36^ as well as the emission quenching experiment (Fig. S4†), a mechanism employing this newly formed organic peroxide as the radical initiator was proposed (Scheme 6B). The sulfur radical generated from the photoredox process promotes the formation of the organic peroxide ^*t*^BuMe_2_SiOOSiMe_2_^*t*^Bu, which provides the siloxyl radical *via* photocatalytic decomposition. This oxygen-centered radical serves as an efficient mediator for HAT of *t*-butyldimethylsilane. The resulting silyl radical undergoes nucleophilic addition with isoquinoline at the C-1 position, followed by oxidation to give the desired product.

**Scheme 7:**
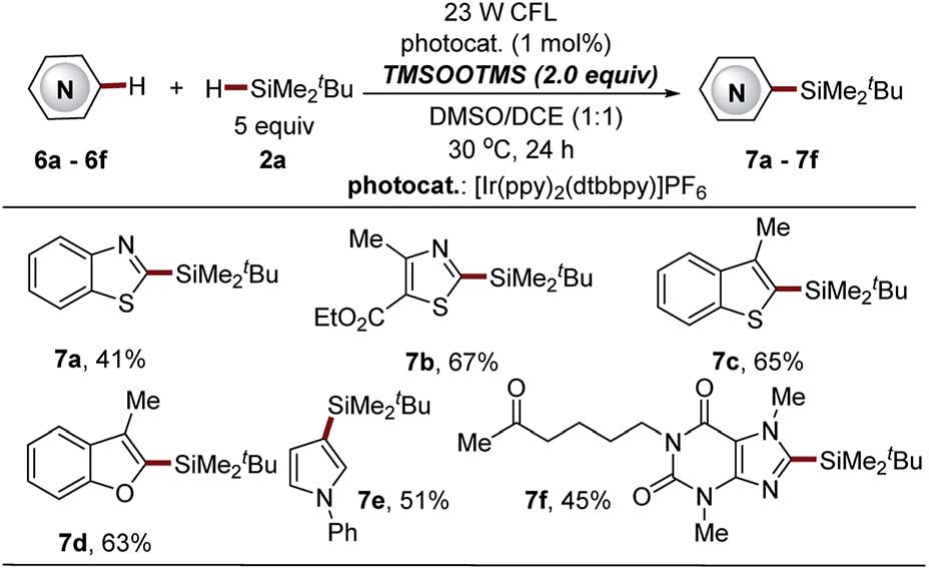
Direct C–H silylation of heteroarenes *via* BTMSPO-mediated photocatalytic Minisci-type reaction. ^*a*^See general procedure C for the experimental details in the ESI,† unless otherwise noted; isolated yields are reported.

Bis(trimethylsilyl)-peroxide (BTMSPO), which is safe and highly soluble in most organic solvents, is widely used in various oxidation reactions in place of hydrogen peroxide (H_2_O_2_).^37^–^40^ The investigation of the reaction mechanism triggered us to further explore the feasibility of the use of BTMSPO as a radical initiator in photocatalytic sila-Minisci-type C–H silylation. Surprisingly, electron-deficient heteroarenes, such as benzothiazole, thiazole and a naturally occurred imidazole structure (pentoxifylline), as well as the electron-rich heteroarenes (benzofuran, benzothiophene and pyrrole structures) were demonstrated as suitable substrates (**7a–7f**, 41–67% yields, Scheme 7). It is of note that these heteroarenes are not amenable to both Na_2_S_2_O_8_- and thiol-mediated reactions. However, this method displayed inferior reaction efficiency in the C–H silylation of isoquinoline, quinoline and pyridine structures (data not shown here). The small trialkylsilanes were also not amenable to this protocol. Further investigation of this promising C–H silylation approach is still ongoing in our laboratory.

### Synthetic applications

#### Late-stage, regioselective modification of bioactive structures

**Scheme 8:**
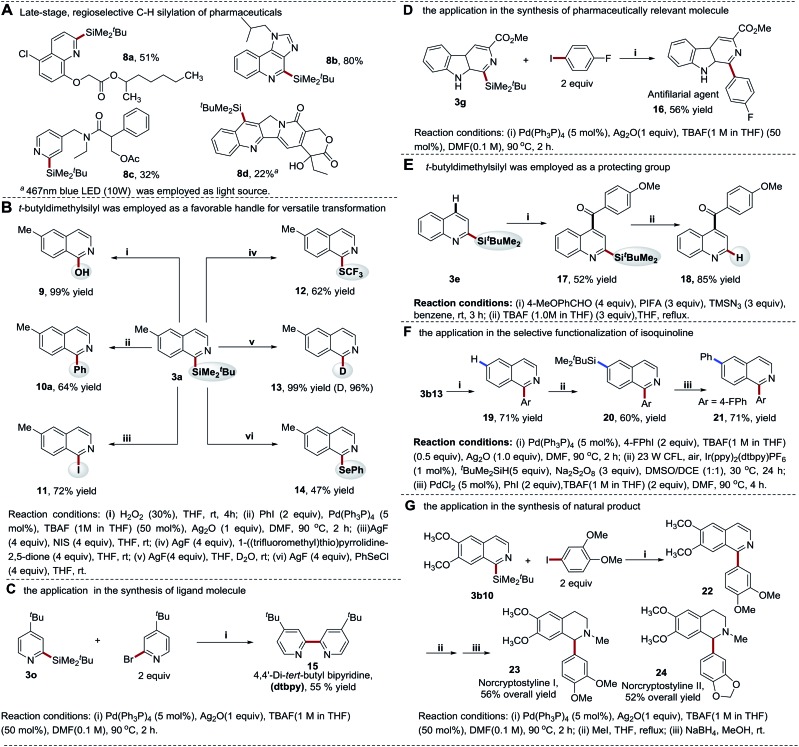
Synthetic applications.

The direct silylation of lead compounds by incorporating trialkylsilyl functionalities can produce metabolically stable organosilicon structures with enhanced lipophilicity and membrane permeability,^1^,^2^ thus offering a useful tool in drug discovery. To demonstrate the synthetic utility of our methods in this study, we evaluated the late-stage C–H functionalization of complex drug molecules (Scheme 8A). We chose three structurally diverse drugs, anticholinergic tropicamide, immunomodulator imiquimod analogue and herbicide cloquintocet-mexyl, for the C–H silylation using the more efficient Na_2_S_2_O_8_-mediated photocatalytic reaction conditions. Notably, the reactions proceeded smoothly, yielding the Si-containing target compounds **8a–8c** in 32–80% yields with excellent chemo- and regio-selectivity. Furthermore, slightly modified reaction conditions by employing a 467 nm blue LED (10 W) as an alternative light source also enabled the direct C–H silylation of camptothecin, producing the anticancer drug candidate DB-67 in a synthetically useful yield (**8d**, 22%, Scheme 8A). Despite the low yield, this method represents the most efficient approach for the synthesis of this valuable target owing to its excellent regioselectivity.^4^

#### Organic transformations of heteroaryl(*tert*-butyldimethylsilyl)silanes

Trialkylsilyl moieties in heteroaryltrialkylsilanes are versatile handles for diverse C–Si based organic transformations.^5^,^6^ Generally, less hindered and smaller sized trialkylsilyl groups are used. However, to the best of our knowledge, heteroaromatics equipped with the larger and inert *tert*-butyldimethylsilyl group have not been used in these transformations. We demonstrated that this class of stable organosilicon functionalities is able to undergo a variety of transformations (Scheme 8B). For example, C-1 Si-directed Hiyama–Denmark cross-coupling^41^–^43^ and Fleming–Tamao oxidation^44^ furnished 1-arylated and 1-oxo isoquinoline structures in good to excellent yields (**9**, 99%; **10a**, 64%). Furthermore, the capacity of this large silyl group-based Hiyama–Denmark cross-coupling reaction was further demonstrated in the coupling of **3a** with various aryl and heteroaryl halides (Scheme S7†), as well as the facile synthesis of pharmaceutical agent **16** (ref. 45) and ligand molecule **15** (Scheme 8C and D).

In addition to the cross-coupling reactions, the silyl group can also be converted to other functionalities. With silver fluoride (AgF) as an activating reagent, the silyl moiety was smoothly converted to iodine (I), thiotrifluoromethyl (SCF_3_), phenylselanyl (SePh), as well as deuterium (D) at the C-1 position of isoquinoline in 47–99% yields *via* nucleophilic substitutions (Scheme 8B). This provides a powerful tool to facilely access the heteroarenes with diverse substituted groups, enabling the quick study of the structure–activity relationship (SAR) of bioactive molecules. Furthermore, the excellent site selectivity of thiol-mediated photocatalytic C–H silylation approach enables the selective manipulation of different C–H bonds of heteroarenes in combination with the new coupling technology (Scheme 8E and F). It is of note that fewer reaction steps and higher yields enable the quick diversification of quinoline and isoquinoline structures. To further demonstrate the utility of our strategy, a new synthetic route towards the synthesis of benzylisoquinoline alkaloids norcryptostyline I–II was developed (Scheme 8G). These two naturally occurring target compounds were readily assembled *via* a modular synthetic approach in 52–56% yields (overall yields of three steps), which provides an alternative efficient method for the synthesis of this class of natural products.^46^,^47^

## Conclusions

In conclusion, motivated by the lack of efficient methods for the direct coupling of the bulky and inert trialkylhydrosilanes with unfunctionalized heteroarenes, especially the medicinally valuable electron-deficient heteroarenes, we have developed a novel photocatalytic Minisci-type C–H silylation approach employing Na_2_S_2_O_8_, BTMSPO, or an alternative safe ^i^Pr_3_SiSH as the radical initiators. While Na_2_S_2_O_8_ and ^i^Pr_3_SiSH serve as efficient promoters for the C–H silylation of electron-deficient heteroarenes, BTMSPO was proved effective for both electron-deficient and electron-rich heteroarenes as a more promising radical initiator. This method employs visible light as the energy source under mild reaction conditions and features broad substrate scope and operational simplicity, allowing the synthesis of structurally diverse heteroaryltrialkylsilanes in moderate to high yields and with good regioselectivity. It is of note that the thio-mediated reaction displays superior regioselectivity and cyano-substituted arenes were also amenable to this method. Furthermore, the silylation products with a large ^*t*^BuMe_2_Si group show great synthetic versatility in diverse C–Si-based chemical transformations. To the best of our knowledge, the employment of this silyl functionality in cross-coupling reactions has not been reported before. Moreover, the applications of our method are further demonstrated in the synthesis of natural products and other complex molecules, as well as the late-stage functionalization. Further studies of this powerful radical-based chemistry for new C–Si bond formation are being pursued in our laboratories.

## Conflicts of interest

There are no conflicts to declare.

## Supplementary Material

Supplementary information
