# Supplementary material for: Photocatalytic C–H silylation of heteroarenes by using trialkylhydrosilanes
Source: Chem Sci. 2019 Feb 18;10(13):3817–25. doi: 10.1039/c9sc00046a (PMC6457191; doi:10.1039/c9sc00046a)

Electronic Supplementary Material (ESI) for Chemical Science.
This journal is © The Royal Society of Chemistry 2019

Supporting information

Photocatalytic C-H Silylation of Heteroarenes by Using Trialkylhydrosilanes

Shihui Liu,[†] Peng Pan,[†] Huaqiang Fan,[†] Hao Li,[†] Wei Wang,[†‡] and Yongqiang Zhang[†]**

[†] State Key Laboratory of Bioengineering Reactor, Shanghai Key Laboratory of New Drug Design and School of Pharmacy, East China University of Science and Technology, Shanghai 200237, P. R. China

[‡] Department of Pharmacology and Toxicology, and BIO5 Institute, University of Arizona, Tucson, Arizona 85721-0207, USA

* Correspondence to: Professor Yongqiang Zhang (Email: yongqiangzhang@ecust.edu.cn) and Professor Wei Wang (E-mail: wwang@pharmacy.arizona.edu)

Table of Contents

1. The optimization of $Na_2S_2O_8$-mediated photocatalytic C-H silylation……................................…S2
2. The chemical stability study of heteroaryltrialkylsilanes………………...……….……………...S4
3. The study of thiol-mediated photocatalytic C-H silylation……….…………….......................S5
4. The application of our methodology in organic synthesis.....……………………………......……S7
5. The study of the mechanism of $Na_2S_2O_8$ mediated photocatalytic C-H silylation……….........S11
6. The study of the mechanism of thiol-mediated photocatalytic C-H silylation...............………......S13
7. Experiment Procedures and Product Characterization...……………………..………………..…S15
8. References……………………………..……………………………..…………………..…S43
9. Spectral Data for Products………………..……………………………..……..………..…...S44

1. The optimization of $Na_2S_2O_8$-mediated photocatalytic C-H silylation.

Table S1. The optimization of photocatalyst. [a]

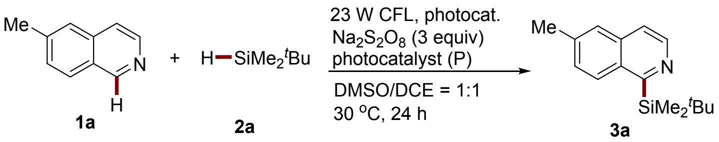

entry	photocat.	yield [b]
1	**P1** (100 mol%)	20% [c]
2	**P1** (100 mol%)	23%
3	**P2** (5 mol%)	25%
4	**P3** (5 mol%)	31%
5	**P4** (5 mol%)	40%
6	**P5** (5 mol%)	trace
7	**P6** (1 mol%)	55%
8	**P7** (1 mol%)	trace
9	**P8** (1 mol%)	trace
10	**P9** (1 mol%)	60%
11	**P9** (0.5 mol%)	53%
12	**P9** (2 mol%)	49%

[a] Conditions employed 23 W CFL, **1a** (0.5 mmol), **2a** (2.5 mmol), photocat. (1 - 100 mol%), $Na_2S_2O_8$ (1.5 mmol), a solvent mixture (2.5 mL, DMSO : DCE = 1 : 1), air, 30 °C, 24 h, unless otherwise noted; [b] Isolated yields were reported; [c] The reaction mixture was degassed via freeze pump thaw (× 3 times) and refilled with N_2.

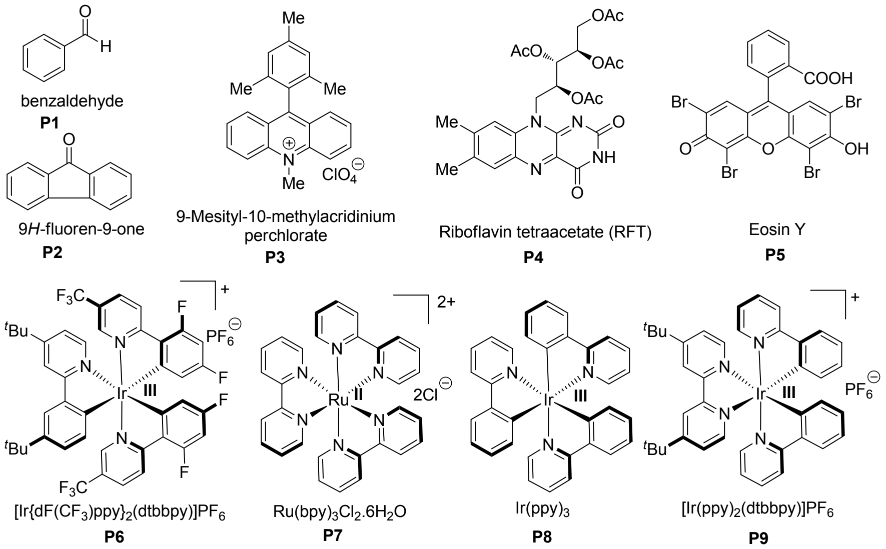

Table S2. The optimization of the oxidant. [a]

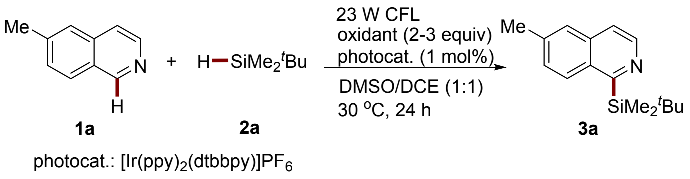

entry	oxidant	yield [b]
1	$Na_2S_2O_8$ (3 equiv)	60%
2	$(NH_4)_2S_2O_8$ (3 equiv)	40%
3	$K_2S_2O_8$ (3 equiv)	51%
4	PIDA (3 equiv)	n.d [c]
5	BPO (75%) (3 equiv)	46%
6	O_2	n.d [c]
7	H_2O_2(30%) (3 equiv)	6%
8	TBHP (70%) (3 equiv)	35%
9	$Na_2S_2O_8$ (2 equiv)	67%
10	$Na_2S_2O_8$ (1 equiv)	54%

[a] Conditions employed **1a** (0.5 mmol), **2a** (2.5 mmol), photocat. (1 mol%), 30 °C, oxidant (1.0 - 1.5 mmol), a solvent mixture (1.5 mL, DMSO:DCE = 1:1), air, 30 °C, 24 h, unless otherwise noted; [b] Isolated yields were reported; [c] not detected.

Table S3. The optimization of the reaction solvent. [a]

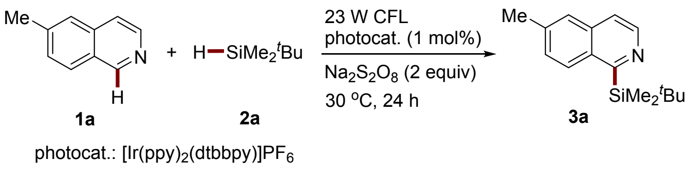

entry	solvent	yield [b]
1	DMSO/DCE (1:1)	67%
2	DMSO	32%
3	DCE	n.d [c]
4	MeCN	trace
5	EtOH	n.d [c]
6	DMSO/$CH_3COOC_2H_5$ (1:1)	19%
7	DMSO/MeCN (1:1)	10%
8	DMSO/Acetone (1:1)	25%
9	DMSO/DCE (1:1)	77% [d]
10	DMSO/DCE (3:1)	67% [d]
11	DMSO/DCE (1:3)	33% [d]

[a] Conditions employed **1a** (0.5 mmol), **2a** (2.5 mmol), photocat. (1 mol%), $Na_2S_2O_8$ (1.0 mmol) , solvent (2.5 mL), 30 °C, 24 h, air, unless otherwise noted; [b] Isolated yields were reported; [c] not detected; [d] 5.0 mL of solvent mixture (DMSO:DCE = 1:1) was employed as the reaction solvent.

Table S4. The optimization of the amount of trialkylhydrosilane. [a]

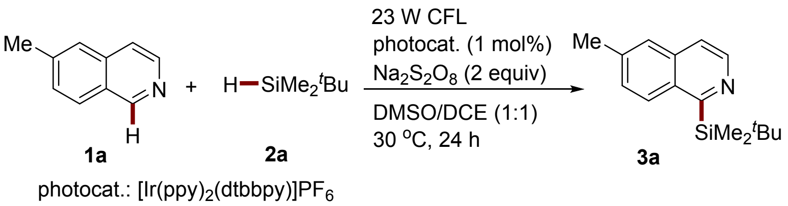

entry	*tert*-butyldimethylsilane	yield [b]
1	5 equiv	77%
2	10 equiv	79%
3	5 equiv	70% [c]
4	5 equiv	66% [d]

[a] Conditions employed 23 W CFL, **1a** (0.5 mmol), **2a** (1.0 - 5.0 mmol), photocat. (1 mol%), $Na_2S_2O_8$ (1.0 mmol), a solvent mixture (5 mL, DMSO : DCE = 1:1), 30 °C, 24 h, air, unless otherwise noted; [b] Isolated yields were reported; [c] The reaction time was extended to 48 h; [d] The reaction time was shorten to 12 h.

2. The chemical stability study of heteroaryltrialkylsilanes

Table S5. The chemical stability study of heteroaryltrialkylsilanes. [a]

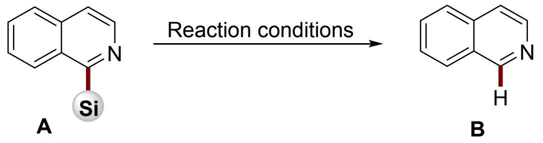

Reaction conditions	**Si** = $^tBuMe_2Si$	**Si** = Et_3Si
Standard reaction conditions ($Na_2S_2O_8$) [b]	**A** (100%)	**A** (21%), **B** (74%)
Standard reaction conditions ($^iPr_3SiSH$) [b]	**A** (100%)	**A** (70%), **B** (30%)
HCl (1 mol/L), 25 °C, 24 h	**A** (100%)	**A** (80%) + **B** (20%)
HCl (1 mol/L), 50 °C, 24 h	**A** (100%)	**A** (10%) + **B** (90%)
HCl (1 mol/L), 80 °C, 24 h	**A** (92%) + **B** (8%)	**B** (100%)
NaOH (1 mol/L), 25 °C, 24 h	**A** (100%)	**A** (100%)
NaOH (1 mol/L), 50 °C, 24 h	**A** (100%)	**A** (70%) + **B** (30%)
NaOH (1 mol/L), 80 °C, 24 h	**A** (100%)	**A** (30%) + **B** (70%)
TBAF (1 mol/L), 25 °C, 24 h	**A** (95%) + **B** (5%)	**B** (100%)
TBAF (1 mol/L), 50 °C, 24 h	**A** (30%) + **B** (70%)	-
TBAF (1 mol/L), 80 °C, 24 h	**B** (100%)	-

[a] Isolated yields were reported for the each reaction; [b] See general procedure **A** and **B** for the experimental details unless otherwise noted.

Notes: Heteroaryltriethylsilane was prepared according to the known procedure, and spectroscopic data is in accordance with literature.[1]

3. The study of thiol-mediated photocatalytic Minisci-type C-H silylation of heteroarenes

Table S6. The optimization of the reaction conditions. [a]

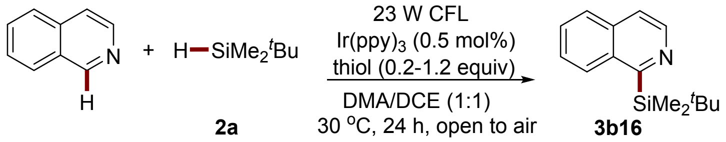

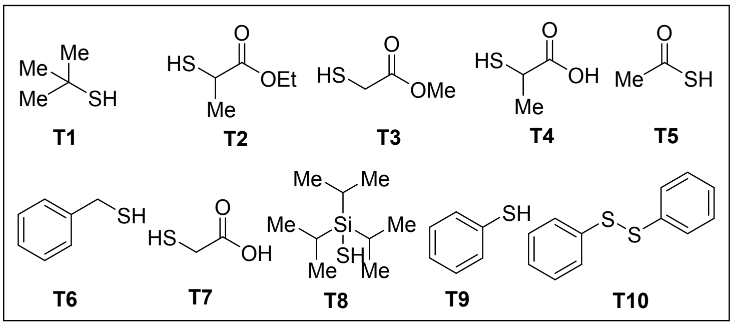

entry	thiol	2a	additive	yield [b]
1	**T1** (0.2 equiv)	5 equiv (1 batch)	-	38%
2	**T2** (0.2 equiv)	5 equiv (1 batch)	-	33%
3	**T3** (0.2 equiv)	5 equiv (1 batch)	-	32%
4	**T4** (0.2 equiv)	5 equiv (1 batch)	-	25%
5	**T5** (0.2 equiv)	5 equiv (1 batch)	-	31%
6	**T6** (0.2 equiv)	5 equiv (1 batch)	-	18%
7	**T7** (0.2 equiv)	5 equiv (1 batch)	-	23%
8	**T8** (0.2 equiv)	5 equiv (1 batch)	-	43%
9	**T9** (0.2 equiv)	5 equiv (1 batch)	-	15%
10	**T10** (0.2 equiv)	5 equiv (1 batch)	-	0%
11	**T8** (1.0 equiv)	5 equiv (1 batch)	-	53%
12	**T8** (1.2 equiv)	5 equiv (1 batch)	-	58%
13	**T8** (1.6 equiv)	5 equiv (1 batch)	-	52%
14	**T8** (1.2 equiv)	5 equiv (3 batches)	-	64%
15	**T8** (1.2 equiv)	10 equiv (3 batches)	-	71%
16	**T8** (1.2 equiv)	10 equiv (3 batches)	-	71% [c]
17	**T8** (1.2 equiv)	10 equiv (3 batches)	-	72% [d]
18	**T8** (1.2 equiv)	10 equiv (3 batches)	CF_3SO_3H	70%
19	**T8** (1.2 equiv)	10 equiv (3 batches)	$Sc(OTf)_3$	63%
20	**T8** (1.2 equiv)	10 equiv (3 batches)	-	n.d [e,f]
21	**T8** (1.2 equiv)	10 equiv (3 batches)	-	71% [g]
22	**T8** (1.2 equiv)	10 equiv (3 batches)	-	n.d [f, h]
23	**T8** (1.2 equiv)	10 equiv (3 batches)	-	n.d [f, i]
24	-	10 equiv (3 batches)	-	n.d [f]

[a] Conditions employed 23 W CFL, isoquinoline (0.5 mmol), **2a** (2.5 - 5.0 mmol), $Ir(ppy)_3$ (0.0025 mmol), thiol (0.1 - 0.6 mmol), 30 °C, 24 h, air, a solvent mixture (2.0 mL, DMA:DCE = 1:1), unless otherwise noted; [b] Isolated yields were reported; [c] The reaction time was extended to 36h; [d] 34 W blue LED was used as light source; [e] The reaction mixture was degassed via freeze pump thaw (× 3 times) and refilled with N_2; [f] Not detected; [g] Performed under oxygen (1atm); [h] Performed in darkness; [i] Performed in the absence of $Ir(ppy)_3$.

Notes: The initial screening campaign identified $Ir(ppy)_3$ (0.5 mol%) and the solvent mixture (DMA/DCE = 1 : 1, 0.25M) as the optimal reaction conditions (data not shown here). Various thiol additives were then screened using 5 equiv of ^{t}BuMe$_2$SiH (**2a**) as coupling partner. The thiol with large

steric hindrance displayed better reaction efficiency (entry 1 and entry 8, 38% - 43% yield). $^{i}Pr_3SiSH$ (1.2 equiv) provided the best reaction outcome (entry 12, 58% yield). The stability of thiol additives under oxidation might help to explain this result. The use of 10 equiv of $^{t}BuMe_2SiH$ in three batches showed the best reaction efficiency (entry 15, 71% yield). The extension of reaction time to 36 h, the use of 34 W blue LED as an alternative light source, as well as the addition of acid as additives, have little effect on the reaction efficiency (entries 16 - 19, 63% - 71% yield). The oxygen was proved essential for this transformation, while the running of the reaction under O_2 cannot further improve the yield (entry 21, 71% yield). Furthermore, control reactions show that visible light, Ir(ppy)$_3$, and thiol, are also essential for this process (entries 22 - 24, Table 1). Therefore, the reaction conditions screening campaign identified the optimal conditions as: in the prescene of 23 W CFL, 0.5 mol% of Ir(ppy)$_3$, 1.2 equiv of $^{i}Pr_3SiSH$, treating heteroarenes with 10 equiv of $^{t}BuMe_2SiH$ (three batches) in solvent mixture (DMA : DCE = 1:1, 0.25 M) for 24 h at 30 °C under air (entry 15).

Scheme S1. Direct C-H silylation of substituted isoquinolines via thiol-mediated photocatalytic Minisci-type reaction. [a]

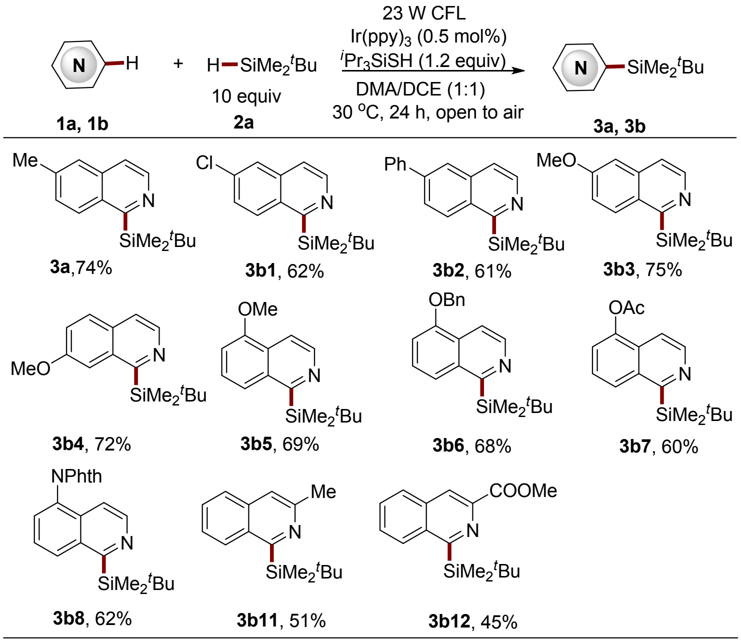

[a] See general procedure **B** for the experimental details unless otherwise noted; the isolated yields were reported.

Notes: Only C-1 *mono*-silylation products were detected.

4. The study of the mechanism of $Na_2S_2O_8$-mediated photocatalytic C-H silylation

Figure S1. The GC-MS study of the reaction mixture.

GC-MS data of the coupling reaction of 6-methylisoquinoline **1a** with *tert*-butyldimethylsilane **2a**.

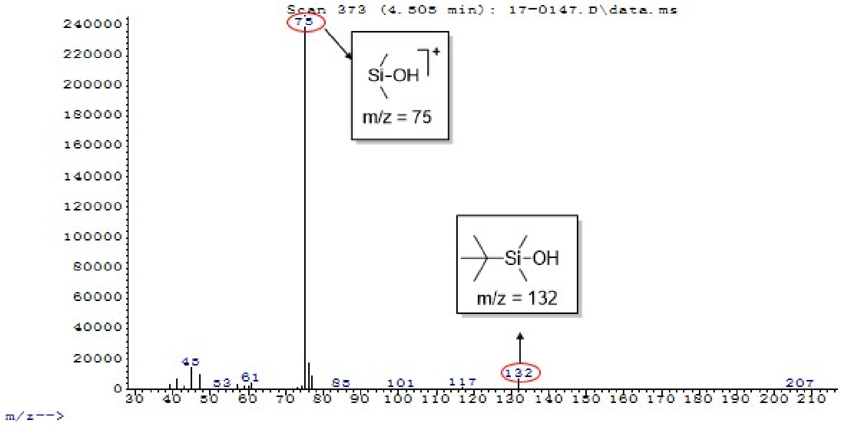

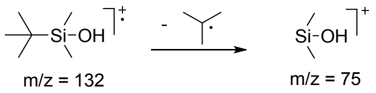

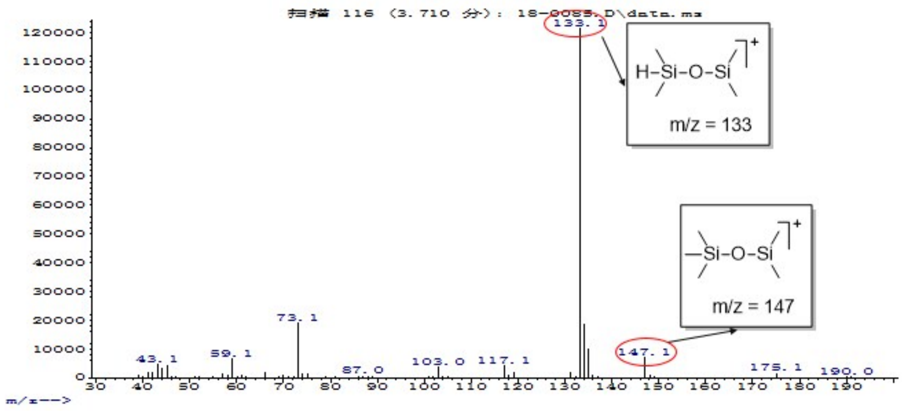

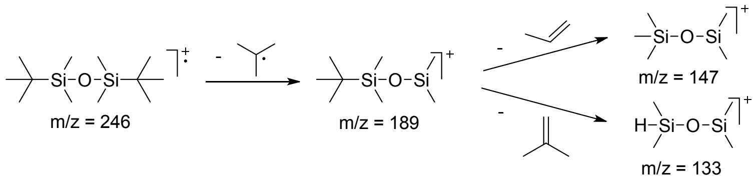

GC-MS data of the coupling reaction of 6-methylisoquinoline **1a** with triethylsilane **2d**

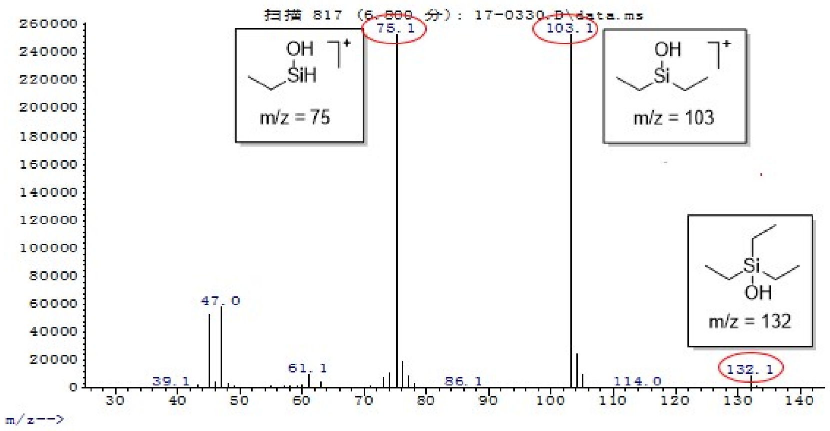

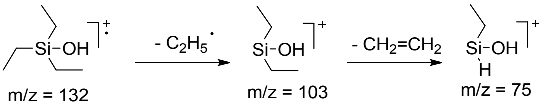

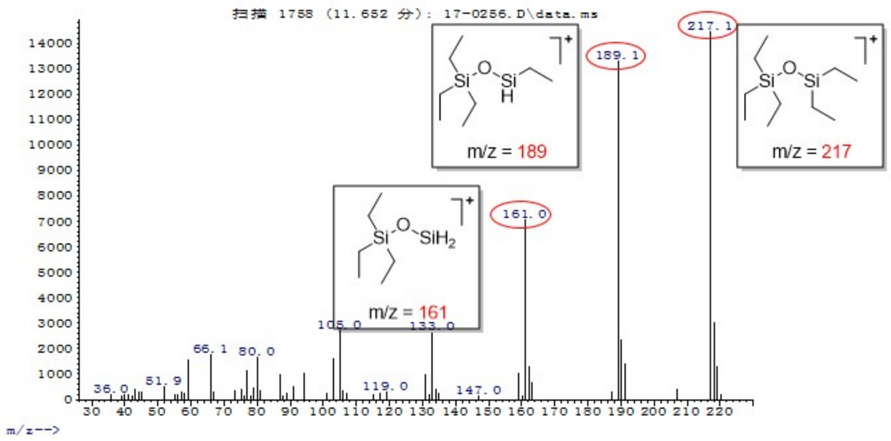

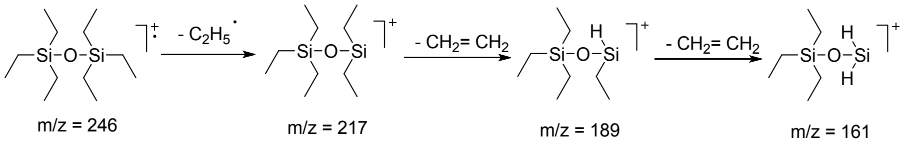

Notes: The reaction solution was directly used for GC-MS analysis. The formation of silanol and siloxane was observed by GC-MS, which indicates that silyl radicals were formed in this process and further validates the radical based mechanism (sila-Minisci-type reaction).[3]

Scheme S2. The proposed mechanisms of the formation of silanol and siloxane.

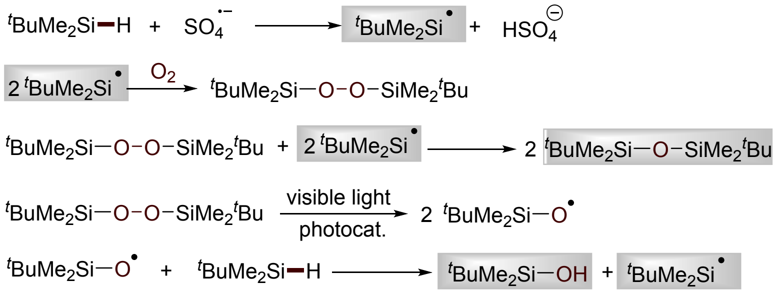

Scheme S3. The radical trapping experiment.

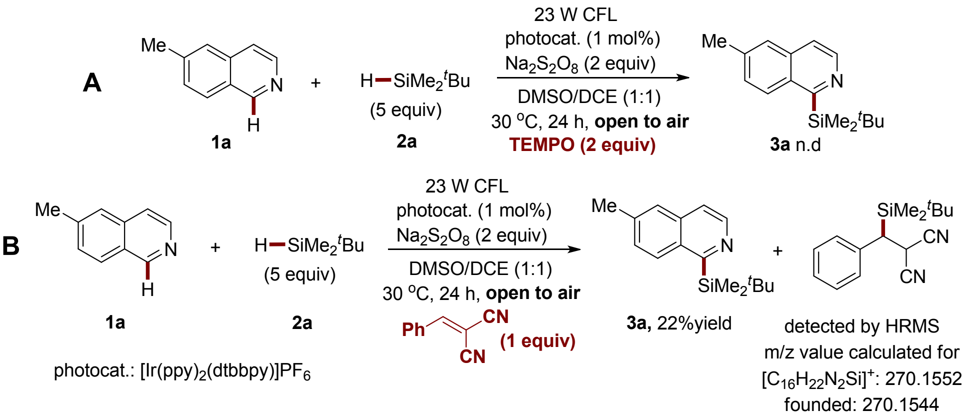

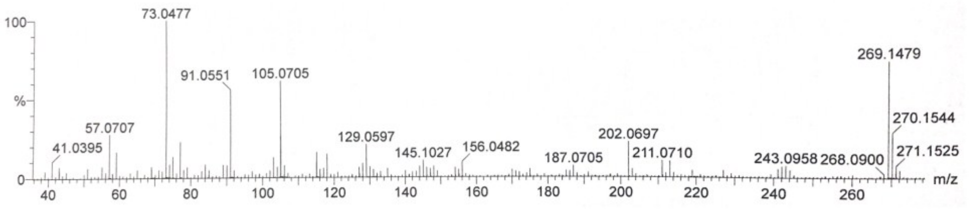

Notes: TEMPO, (*E*)-(2-(phenylsulfonyl)vinyl)benzene, methyl acrylate, and 2-benzylidenemalononitrile were employed to trap the silyl radial. The reaction was shut down by the presence of TEMPO, which is indicative of a radical engaged pathway (Scheme 3A). However, only the radial trapping product was detected using 2-benzylidenemalononitrile as additive (Scheme 3B).

Scheme S4. The control reactions employing ${}^tBuMe_2SiOOSiMe_2{}^tBu$ as the radical initiator.

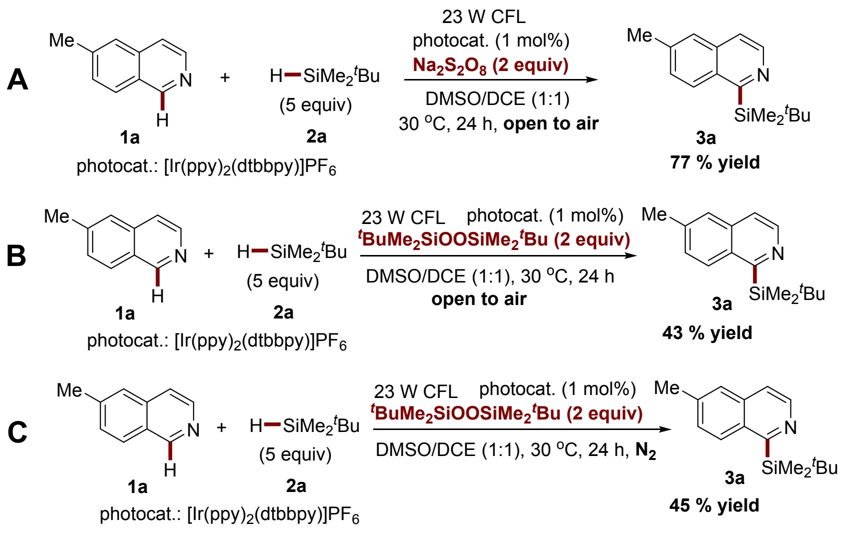

Notes: ${}^tBuMe_2SiOOSiMe_2{}^tBu$ was prepared according to known procedure, and spectroscopic data is in accordance with literature.[3]

Figure S2. Emission Quenching Experiment

Emission intensities were recorded using a FluoroMax-4 (Horiba Scientific) fluorescence spectrophotometer. All $Ir(ppy)_2(dtbbpy)PF_6$ solutions were excited at 337 nm and the emission intensity was collected at 603 nm. In a typical experiment, to a 1 × 10^{-6} M solution of $Ir(ppy)_2(dtbbpy)PF_6$ in DMSO was added the appropriate amount of a quencher in a screw-top quartz cuvette.

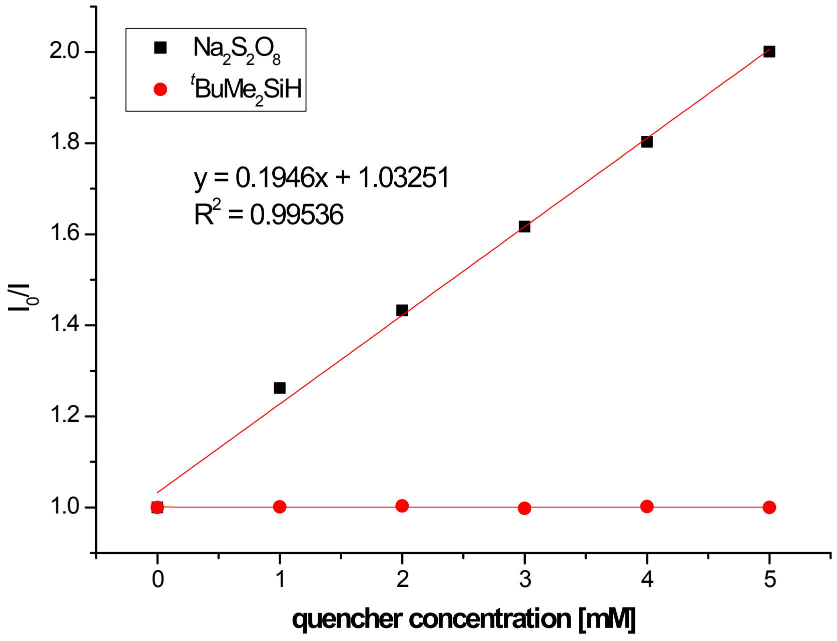

5. The study of the mechanism of thiol-mediated photocatalytic C-H silylation

Figure S3. GC-MS study of the reaction mixture.

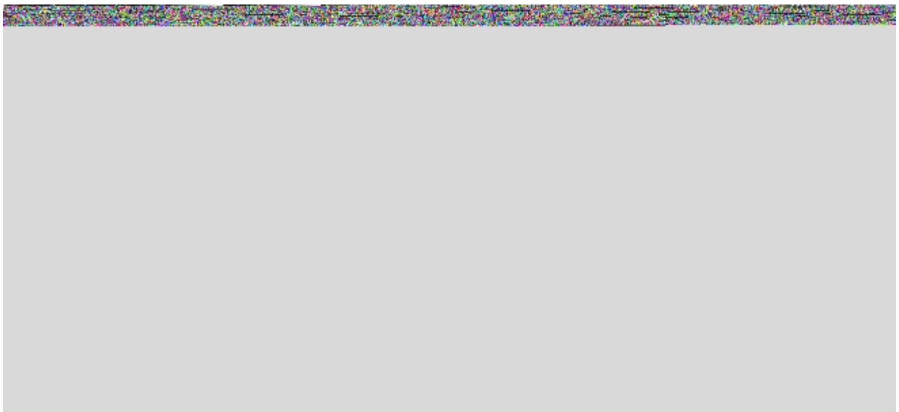

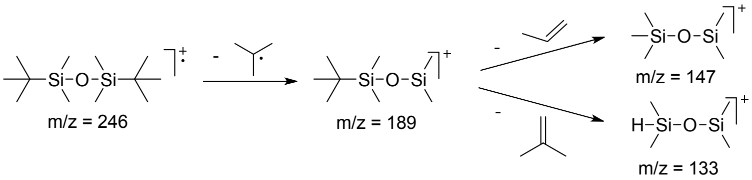

Scheme S5. The radical quenching and trapping experiments.

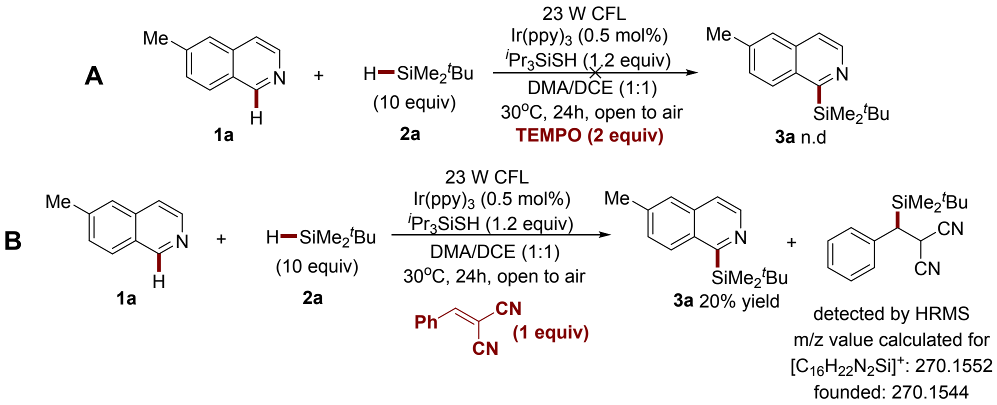

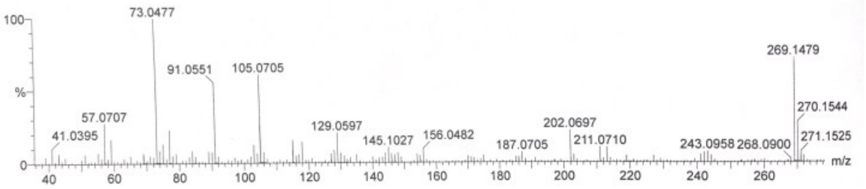

Notes: The reaction was completely inhibited by the presence of TEMPO, further suggesting the radical based mechanism (Scheme S5A). However, only the use of 2-benzylidenemalononitrile as additive is able to produce the radial trapping product accompanied with the formation of little target product **3a** (Scheme S5B).

Scheme S6. The control reactions.

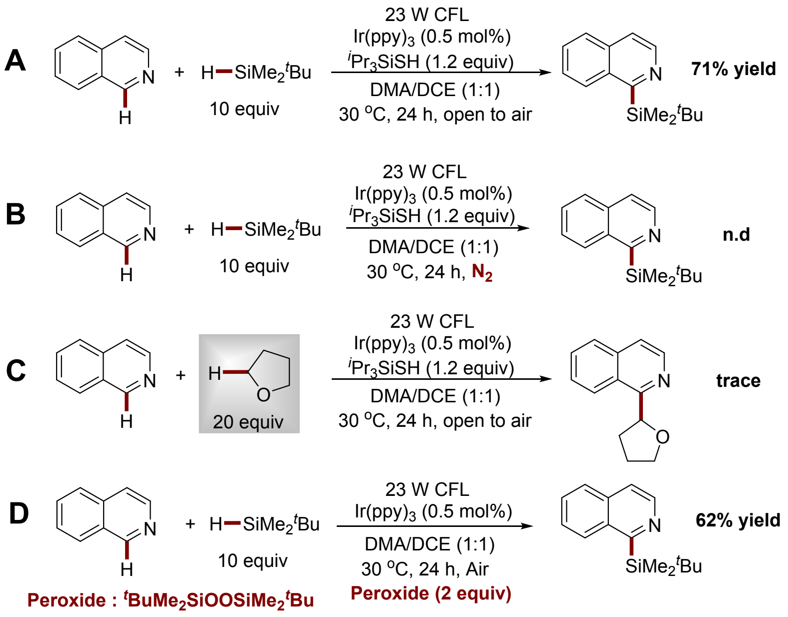

Notes: No product was detected in the absence of air (O_2) (Scheme S6B). Furthermore, the use of THF (20 equiv) as coupling partner in place of ^{t}BuMe$_2$SiH resulted in the generation of trace desired product (Scheme S6C). These results, as well as the fact that the use of ^{t}BuMe$_2$SiOOSiMe$_2$^{t}Bu (2 equiv) as radical initiator led to comparable yield (62% yield, Scheme S6D), indicate that the *in-situ* generated ^{t}BuMe$_2$SiOOSiMe$_2$^{t}Bu might be involved in this process to mediate the sila-Minisci-type reaction.

Figure S4. Emission Quenching Experiment.

Emission intensities were recorded using a FluoroMax-4 (Horiba Scientific) fluorescence spectrophotometer. All Ir(ppy)$_3$ solutions were excited at 350 nm and the emission intensity was collected at 527 nm. In a typical experiment, to a 1×10^{-6} M solution of Ir(ppy)$_3$ in DMSO was added the appropriate amount of a quencher in a screw-top quartz cuvette.

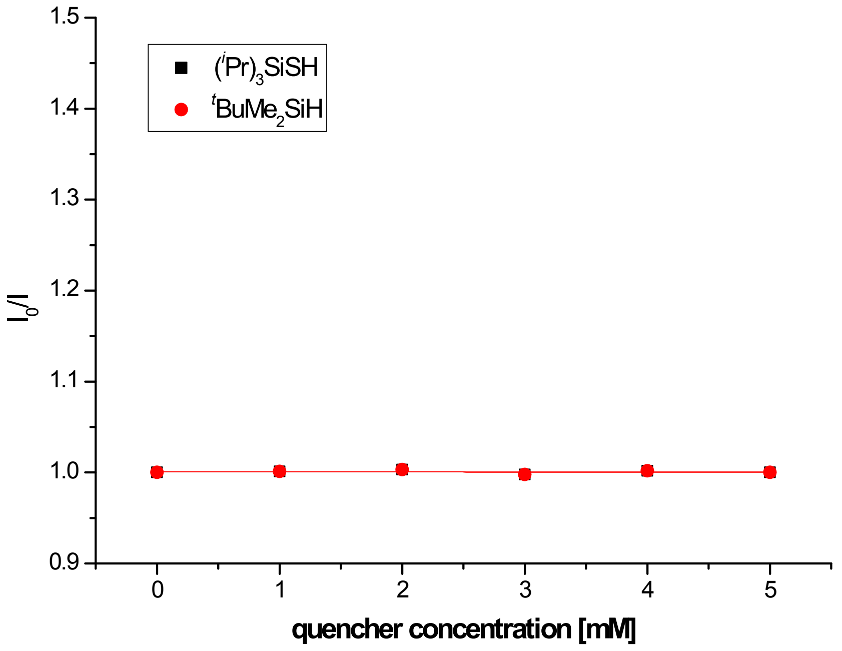

6. The synthetic application of our method

Scheme S7. The study of Hiyama-Denmark cross-coupling of heteroaryl(*tert*-butyldimethyl)silane. [a]

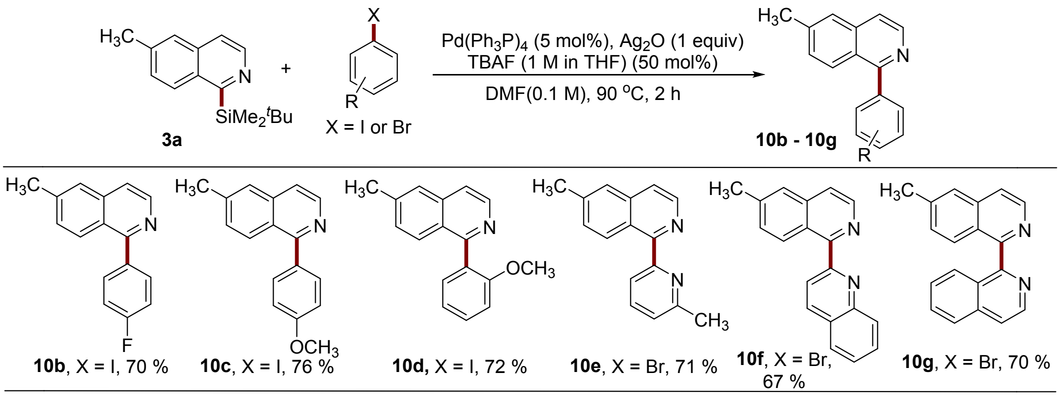

[a] See General procedure **D** for the experimental details unless otherwise noted; isolated yields were reported.

Scheme S8. The comparison of two synthetic routes for the synthesis of norcryptostylines.

Reported synthetic route: [2]

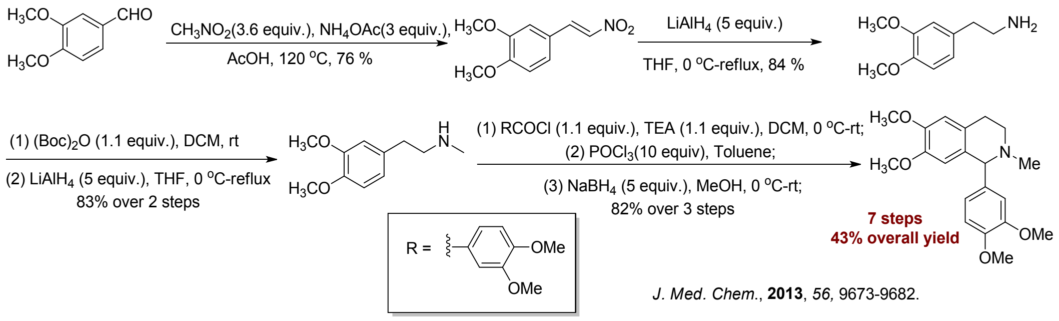

Our synthetic route:

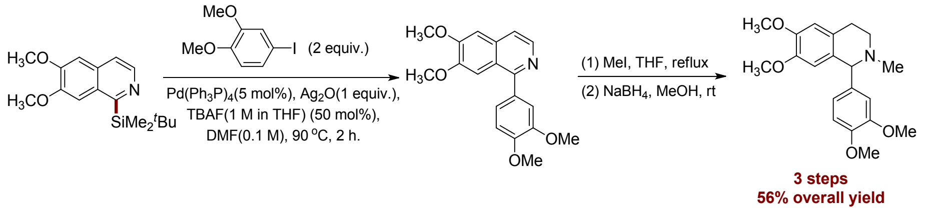

7. Experiment Procedures and Product Characterization

Commercial reagents and solvents were used as received, unless otherwise stated. Organic solution was concentrated under reduced pressure on a Büchi rotary evaporator using an isopropyl alcohol-dry ice bath. Analytical thin layer chromatography (TLC) was performed on 0.25 mm silica gel plates (Qingdao Haiyang Chemical China), and the compounds were visualized with a UV light at 254 nm. Further visualization was achieved by staining with iodine. Flash chromatography was performed on silica gel 200–300 mesh (purchased from Qingdao Haiyang Chemical China) with commercial solvents (purchased from Adamas-beta®). The ^{1}H and ^{13}C NMR spectra were recorded on a Bruker AM 400 Spectrometer (400 and 100 MHz for ^{1}H and ^{13}C NMR, respectively) and are internally referenced to residual solvent signals (note: $CDCl_3$ referenced at 7.26 and 77.00 ppm in ^{1}H and ^{13}C NMR, respectively; d^{6}-DMSO referenced at 2.50 and 39.52 ppm in ^{1}H and ^{13}C NMR, respectively). Multiplicities were given as s (singlet), d (doublet), t (triplet), dd (double of doublet), and m (multiplets). Coupling constants were reported in Hertz (Hz). Data for ^{13}C NMR are reported in terms of chemical shift. High-resolution mass spectrometry (HRMS) was recorded on Waters LCT Premier XE spectrometer.

General Procedure A:

To a 10 mL vial equipped with a Teflon septum and magnetic stir bar were added the corresponding heterocycles (0.5 mmol, 1.0 equiv.), the corresponding silane (2.5 mmol, 5.0 equiv.), $Na_2S_2O_8$ (1.0 mmol, 2.0 equiv.) and $Ir(ppy)_2(dtbpy)PF_6$ (0.005 mmol, 0.01 equiv.) The vial was sealed and placed under atmosphere, then DMSO/DCE (1:1) (5 mL, 0.1 M) were added. The reaction was placed in between 2 × 23 W fluorescent lamps (approximately 5 cm from each lamp) and irradiated for 24 hours. The reaction mixture was diluted with saturated $NaHCO_3$ aqueous solution, extracted with ethyl acetate (3 × 20 mL), the combined organic extracts were washed with brine (30 mL), dried over anhydrous Na_2SO_4 and concentrated in vacuo. Purification of the crude product by flash chromatography on silica gel using the indicated solvent system afforded the desired product.

General Procedure B:

To a 10 mL vial equipped with a Teflon septum and magnetic stir bar were added the corresponding heterocycles (0.5 mmol, 1.0 equiv.), the corresponding silane (1.67 mmol, 3.33 equiv.), triisopropylsilanethiol (0.6 mmol, 1.2 equiv.) and $Ir(ppy)_3$ (0.0025 mmol, 0.005 equiv.) The vial was sealed and placed under atmosphere, then DMA/DCE (1:1) (2 mL, 0.25 M) were added. The reaction was placed in between 2 × 23 W fluorescent lamps (approximately 5 cm from each lamp) and irradiated for 24 hours. In this period, another two batch (1.67 mmol, 3.33 equiv.) added after every 8 hours. The reaction mixture was diluted with saturated $NaHCO_3$ aqueous solution, extracted with ethyl acetate (3 × 20 mL), the combined organic extracts were washed with brine (30 mL), dried over anhydrous Na_2SO_4 and concentrated in vacuo. Purification of the crude product by flash chromatography on silica gel using the indicated solvent system afforded the desired product.

General Procedure C:

To a 10 mL vial equipped with a Teflon septum and magnetic stir bar were added the corresponding heterocycles (0.5 mmol, 1.0 equiv.), the corresponding silane (2.5 mmol, 5.0 equiv.), BTMSPO (1.0

mmol, 2.0 equiv.) and $Ir(ppy)_2(dtbpy)PF_6$ (0.005 mmol, 0.01 equiv.) The vial was sealed and placed under atmosphere, then DMSO/DCE (1:1) (5 mL, 0.1 M) were added. The reaction was placed in between 2 × 23 W fluorescent lamps (approximately 5 cm from each lamp) and irradiated for 24 hours. The reaction mixture was diluted with H_2O, extracted with ethyl acetate (3 × 20 mL), the combined organic extracts were washed with brine (30 mL), dried over anhydrous Na_2SO_4 and concentrated in vacuo. Purification of the crude product by flash chromatography on silica gel using the indicated solvent system afforded the desired product.

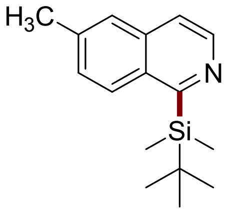

1-(*tert*-butyldimethylsilyl)-6-methylisoquinoline (3a): According to the general procedure A, 6-methylisoquinoline (0.5 mmol, 1 equiv.), *tert*-butyldimethylsilane (290 mg, 2.5 mmol, 5 equiv.), $Na_2S_2O_8$ (238 mg, 1.0 mmol, 2.0 equiv.), $Ir(ppy)_2(dtbpy)PF_6$ (4.6 mg, 0.005 mmol, 0.01 equiv.) and 5 mL DMSO/DCE (1:1) (0.1 M) were used. The product was isolated by flash chromatography (2% ethyl acetate/hexane) as a colorless solid (100 mg, 77 %). And according to the general procedure B, the product was obtained (96 mg, 74 %). ^1H NMR (400 MHz, $CDCl_3$) δ 8.67 (d, J = 5.6 Hz, 1H), 8.15 (d, J = 8.6 Hz, 1H), 7.56 (s, 1H), 7.47 (d, J = 5.6 Hz, 1H), 7.39 (dd, J = 8.6, 1.7 Hz, 1H), 2.53 (s, 3H), 0.97 (s, 9H), 0.56 (s, 6H); ^{13}C NMR (100 MHz, $CDCl_3$) δ 169.49, 142.63, 139.24, 134.83, 132.53, 128.59, 128.41, 126.37, 119.41, 27.09, 21.75, 17.96, -3.08; HRMS (ESI) Calcd. for $C_{16}H_{24}NSi$ [$(M+H)^+$] 258.1678, found 258.1676.

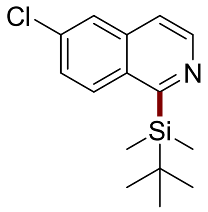

1-(*tert*-butyldimethylsilyl)-6-chloroisoquinoline (3b1): According to the general procedure A, 6-chloroisoquinoline (0.5 mmol, 1 equiv.), *tert*-butyldimethylsilane (72 mg, 2.5 mmol, 5 equiv.), $Na_2S_2O_8$ (238 mg, 1.0 mmol, 2.0 equiv.), $Ir(ppy)_2(dtbpy)PF_6$ (4.6 mg, 0.005 mmol, 0.01 equiv.) and 5 mL DMSO/DCE (1:1) (0.1 M) were used. The product was isolated by flash chromatography (2% ethyl acetate/hexane) as a colorless solid(85 mg, 61 %). And according to the general procedure B, the product was obtained (86 mg, 62 %). ^1H NMR (400 MHz, $CDCl_3$) δ 8.71 (d, J = 5.6 Hz, 1H), 8.18 (d, J = 9.0 Hz, 1H), 7.78 (d, J = 1.7 Hz, 1H), 7.49 (dd, J = 12.6, 3.8 Hz, 2H), 0.95 (s, 9H), 0.54 (s, 6H); ^{13}C NMR (100 MHz, $CDCl_3$) δ 170.43, 143.40, 135.44, 135.23, 132.10, 130.31, 127.41, 126.25, 118.88, 27.00, 17.92, -3.13; HRMS (ESI) Calcd. for $C_{15}H_{21}ClNSi$ [$(M+H)^+$] 278.1132, found 278.1124.

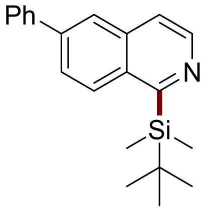

1-(*tert*-butyldimethylsilyl)-6-phenylisoquinoline (3b2): According to the general procedure A, 6-phenylisoquinoline (0.5 mmol, 1 equiv.), *tert*-butyldimethylsilane (290 mg, 2.5 mmol, 5 equiv.), $Na_2S_2O_8$ (238 mg, 1.0 mmol, 2.0 equiv.), $Ir(ppy)_2(dtbpy)PF_6$ (4.6 mg, 0.005 mmol, 0.01 equiv.) and 5 mL DMSO/DCE (1:1) (0.1 M) were used. The product was isolated by flash chromatography (2% ethyl acetate/hexane) as a colorless solid (115 mg, 72 %). And according to the general procedure B, the product was obtained (97 mg, 61 %). ^1H NMR (400 MHz, $CDCl_3$) δ 8.74 (d, J = 5.6 Hz, 1H), 8.32 (d, J = 8.8 Hz, 1H), 7.99 (d, J = 1.7 Hz, 1H), 7.84 (dd, J = 8.8, 1.8 Hz, 1H), 7.73 (dd, J = 5.2, 3.3 Hz, 2H), 7.62 (d, J = 5.6 Hz, 1H), 7.51 (dd, J = 10.2, 4.7 Hz, 2H), 7.43 (ddd, J = 7.4, 3.8, 1.2 Hz, 1H), 0.99 (s, 9H), 0.59 (s, 6H); ^{13}C NMR (100 MHz, $CDCl_3$) δ 169.87, 142.82, 141.80, 140.14, 134.96, 133.03, 129.22, 128.98, 128.03, 127.49, 126.19, 125.18, 120.18, 27.10, 18.01, -3.06; HRMS (ESI) Calcd. for $C_{21}H_{26}NSi$ [$(M+H)^+$] 320.1835, found 320.1841.

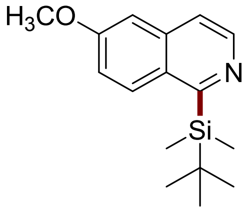

1-(*tert*-butyldimethylsilyl)-6-methoxyisoquinoline (3b3): According to the general procedure A, 6-methoxyisoquinoline (0.5 mmol, 1 equiv.), *tert*-butyldimethylsilane (290 mg, 2.5 mmol, 5 equiv.), $Na_2S_2O_8$ (238 mg, 1.0 mmol, 2.0 equiv.), $Ir(ppy)_2(dtbpy)PF_6$ (4.6 mg, 0.005 mmol, 0.01 equiv.) and 5 mL DMSO/DCE (1:1) (0.1 M) were used. The product was isolated by flash chromatography (2% ethyl acetate/hexane) as a colorless oil (94 mg, 69 %). And according to the general procedure B, the product was obtained (102 mg, 75 %). ^1H NMR (400 MHz, $CDCl_3$) δ 8.62 (d, J = 5.7 Hz, 1H), 8.14 (d, J = 9.2 Hz, 1H), 7.46 (d, J = 5.7 Hz, 1H), 7.18 (dd, J = 9.2, 2.6 Hz, 1H), 7.04 (d, J = 2.5 Hz, 1H), 3.94 (s, 3H), 0.95 (s, 9H), 0.54 (s, 6H); ^{13}C NMR (100 MHz, $CDCl_3$) δ 168.99, 159.71, 143.01, 136.59, 130.37, 130.12, 119.29, 119.21, 104.83, 55.37, 27.07, 17.96, -3.06; HRMS (ESI) Calcd. for $C_{16}H_{24}NOSi$ [$(M+H)^+$] 274.1627, found 274.1624.

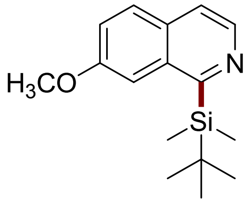

1-(*tert*-butyldimethylsilyl)-7-methoxyisoquinoline (3b4): According to the general procedure A, 7-methoxyisoquinoline (0.5 mmol, 1 equiv.), *tert*-butyldimethylsilane (290 mg, 2.5 mmol, 5 equiv.), $Na_2S_2O_8$ (238 mg, 1.0 mmol, 2.0 equiv.), $Ir(ppy)_2(dtbpy)PF_6$ (4.6 mg, 0.005 mmol, 0.01 equiv.) and 5 mL DMSO/DCE (1:1) (0.1 M) were used. The product was isolated by flash chromatography (2% ethyl acetate/hexane) as a colorless solid (95 mg, 71 %). And according to the general procedure B, the product was obtained (96 mg, 72 %). ^1H NMR (400 MHz, $CDCl_3$) δ 8.61 (d, J = 5.5 Hz, 1H), 7.70 (d, J = 8.9 Hz, 1H), 7.55 (d, J = 2.4 Hz, 1H), 7.49 (d, J = 5.5 Hz, 1H), 7.30 (dd, J = 8.9, 2.5 Hz, 1H), 3.94 (s, 3H), 0.98 (s, 9H), 0.57 (s, 6H); ^{13}C NMR (100 MHz, $CDCl_3$) δ 167.78, 157.51, 141.13, 135.10, 130.01, 128.91, 122.21, 119.67, 106.62, 55.27, 27.14, 18.29, -3.20; HRMS (ESI) Calcd. for $C_{16}H_{24}NOSi$ [$(M+H)^+$] 274.1627, found 274.1625.

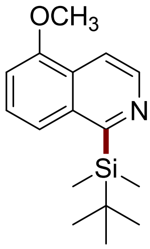

1-(*tert*-butyldimethylsilyl)-5-methoxyisoquinoline (3b5): According to the general procedure A, 5-methoxyisoquinoline (0.5 mmol, 1 equiv.), *tert*-butyldimethylsilane (290 mg, 2.5 mmol, 5 equiv.), $Na_2S_2O_8$ (238 mg, 1.0 mmol, 2.0 equiv.), $Ir(ppy)_2(dtbpy)PF_6$ (4.6 mg, 0.005 mmol, 0.01 equiv.) and 5 mL DMSO/DCE (1:1) (0.1 M) were used. The product was isolated by flash chromatography (2% ethyl acetate/hexane) as a colorless solid (100 mg, 73 %). And according to the general procedure B, the product was obtained (95 mg, 69 %). ^1H NMR (400 MHz, $CDCl_3$) δ 8.73 (d, J = 5.8 Hz, 1H), 7.97 (dd, J = 5.8, 0.6 Hz, 1H), 7.82 (d, J = 8.6 Hz, 1H), 7.49 – 7.42 (m, 1H), 6.94 (d, J = 7.7 Hz, 1H), 3.99 (s, 3H), 0.96 (s, 9H), 0.55 (s, 6H); ^{13}C NMR (100 MHz, $CDCl_3$) δ 169.11, 154.84, 142.32, 134.61, 127.15, 126.20, 120.58, 113.92, 106.41, 55.57, 27.10, 18.01, -3.03; HRMS (ESI) Calcd. for $C_{16}H_{24}NOSi$ [$(M+H)^+$] 274.1627, found 274.1620.

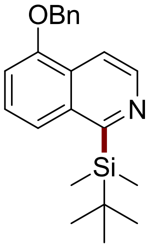

5-(benzyloxy)-1-(*tert*-butyldimethylsilyl)isoquinoline (3b6): According to the general procedure A, 5-(benzyloxy)isoquinoline (0.5 mmol, 1 equiv.), *tert*-butyldimethylsilane (290 mg, 2.5 mmol, 5 equiv.), $Na_2S_2O_8$ (238 mg, 1.0 mmol, 2.0 equiv.), $Ir(ppy)_2(dtbpy)PF_6$ (4.6 mg, 0.005 mmol, 0.01 equiv.) and 5 mL DMSO/DCE (1:1) (0.1 M) were used. The product was isolated by flash chromatography (2% ethyl acetate/hexane) as a colorless oil (123 mg, 70 %). And according to the general procedure B, the product was obtained (119 mg, 68 %). ^1H NMR (400 MHz, $CDCl_3$) δ 8.73 (d, J = 5.8 Hz, 1H), 8.06 (d, J = 5.7 Hz, 1H), 7.83 (d, J = 8.6 Hz, 1H), 7.52 (d, J = 7.3 Hz, 2H), 7.46 - 7.41 (m, 3H), 7.37 (d, J = 7.2 Hz, 1H), 7.03 (d, J = 7.7 Hz, 1H), 5.25 (s, 2H), 0.96 (s, 9H), 0.55 (s, 6H); ^{13}C NMR (100 MHz, $CDCl_3$) δ 169.18, 153.91, 142.37, 136.67, 134.70, 128.65, 128.09, 127.35, 126.20, 120.91, 114.10, 107.87, 70.24, 27.12, 18.03, -3.02; HRMS (ESI) Calcd. for $C_{22}H_{28}NOSi$ [$(M+H)^+$] 350.1940, found 350.1943.

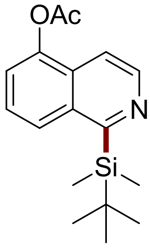

1-(*tert*-butyldimethylsilyl)isoquinolin-5-yl acetate (3b7): According to the general procedure A, isoquinolin-5-yl acetate (0.5 mmol, 1 equiv.), *tert*-butyldimethylsilane (290 mg, 2.5 mmol, 5 equiv.), $Na_2S_2O_8$ (238 mg, 1.0 mmol, 2.0 equiv.), $Ir(ppy)_2(dtbpy)PF_6$ (4.6 mg, 0.005 mmol, 0.01 equiv.) and 5 mL DMSO/DCE (1:1) (0.1 M) were used. The product was isolated by flash chromatography (2%

ethyl acetate/hexane) as a colorless oil (95 mg, 63 %). And according to the general procedure B, the product was obtained (90 mg, 60 %). ^{1}H NMR (400 MHz, $CDCl_3$) δ 8.74 (d, *J* = 5.8 Hz, 1H), 8.15 (d, *J* = 8.5 Hz, 1H), 7.56 (dd, *J* = 15.2, 7.2 Hz, 2H), 7.42 (d, *J* = 7.5 Hz, 1H), 2.47 (s, 3H), 0.97 (s, 9H), 0.55 (s, 6H); ^{13}C NMR (100 MHz, $CDCl_3$) δ 170.54, 169.27, 145.97, 142.93, 134.63, 128.32, 126.65, 125.80, 121.23, 112.91, 27.05, 20.95, 17.97, -3.05; HRMS (ESI) Calcd. for $C_{17}H_{24}NO_2Si$ $[(M+H)^+]$ 302.1576, found 302.1578.

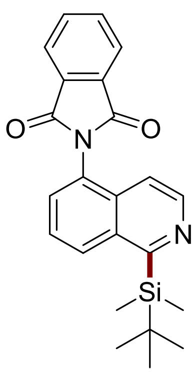

2-(1-(*tert*-butyldimethylsilyl)isoquinolin-5-yl)isoindoline-1,3-dione (3b8): According to the general procedure A, 2-(isoquinolin-5-yl)isoindoline-1,3-dione (0.5 mmol, 1 equiv.), *tert*-butyldimethylsilane (290 mg, 2.5 mmol, 5 equiv.), $Na_2S_2O_8$ (238 mg, 1.0 mmol, 2.0 equiv.), $Ir(ppy)_2(dtbpy)PF_6$ (4.6 mg, 0.005 mmol, 0.01 equiv.) and 5 mL DMSO/DCE (1:1) (0.1 M) were used. The product was isolated by flash chromatography (2 % ethyl acetate/hexane) as a colorless solid (128 mg, 66 %). And according to the general procedure B, the product was obtained (120 mg, 62 %). ^{1}H NMR (400 MHz, $CDCl_3$) δ 8.33 (d, *J* = 8.6 Hz, 1H), 8.01 (dd, *J* = 5.4, 3.1 Hz, 2H), 7.87 – 7.77 (m, 4H), 7.59 (d, *J* = 8.5 Hz, 1H), 7.51 (dd, *J* = 7.3, 0.9 Hz, 1H), 0.98 (s, 9H), 0.41 (s, 6H); ^{13}C NMR (100 MHz, $CDCl_3$) δ 170.03, 167.62, 149.12, 135.58, 132.08, 131.82, 128.05, 127.74, 127.50, 127.05, 126.65, 124.48, 123.95, 26.60, 17.16, -6.25; HRMS (ESI) Calcd. for $C_{23}H_{25}N_2O_2Si$ $[(M+H)^+]$ 389.1685, found 389.1891.

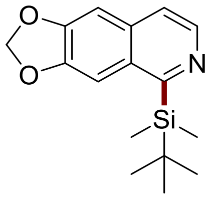

5-(*tert*-butyldimethylsilyl)-[1,3]dioxolo[4,5-g]isoquinoline (3b9): According to the general procedure A, [1,3]dioxolo[4,5-g]isoquinoline (0.5 mmol, 1 equiv.), *tert*-butyldimethylsilane (290 mg, 2.5 mmol, 5 equiv.), $Na_2S_2O_8$ (238 mg, 1.0 mmol, 2.0 equiv.), $Ir(ppy)_2(dtbpy)PF_6$ (4.6 mg, 0.005 mmol, 0.01 equiv.) and 5 mL DMSO/DCE (1:1) (0.1 M) were used. The product was isolated by flash chromatography (10% ethyl acetate/hexane) as a colorless solid (83 mg, 58 %). ^{1}H NMR (400 MHz, $CDCl_3$) δ 8.55 (d, *J* = 5.5 Hz, 1H), 7.50 (s, 1H), 7.40 (d, *J* = 5.5 Hz, 1H), 7.04 (s, 1H), 6.08 (s, 2H), 0.95 (s, 9H), 0.53 (s, 6H).^{13}C NMR (100 MHz, $CDCl_3$) δ 147.73, 141.79, 133.01, 119.70, 104.48, 103.10, 101.42, 29.69, 27.07, 18.02, -3.00; HRMS (ESI) Calcd. for $C_{16}H_{22}NO_2Si$ $[(M+H)^+]$ 288.1420, found 288.1431.

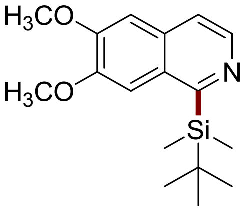

1-(*tert*-butyldimethylsilyl)-6,7-dimethoxyisoquinoline (3b10): According to the general procedure A, 6,7-dimethoxyisoquinoline (0.5 mmol, 1 equiv.), *tert*-butyldimethylsilane (290 mg, 2.5 mmol, 5 equiv.), $Na_2S_2O_8$ (238 mg, 1.0 mmol, 2.0 equiv.), $Ir(ppy)_2(dtbpy)PF_6$ (4.6 mg, 0.005 mmol, 0.01 equiv.) and 5 mL DMSO/DCE (1:1) (0.1 M) were used. The product was isolated by flash chromatography (10 % ethyl acetate/hexane) as a colorless solid (92 mg, 61 %). ^1H NMR (400 MHz, $CDCl_3$) δ 8.58 (d, J = 5.5 Hz, 1H), 7.54 (s, 1H), 7.42 (d, J = 5.5 Hz, 1H), 7.03 (s, 1H), 4.01 (d, J = 2.6 Hz, 6H), 0.96 (s, 9H), 0.56 (s, 6H); ^{13}C NMR (100 MHz, $CDCl_3$) δ 166.25, 151.8, 149.21, 141.76, 131.27, 130.28, 118.79, 107.19, 105.16, 55.91, 55.77, 27.09, 18.28, -3.26; HRMS (ESI) Calcd. for $C_{17}H_{26}NO_2Si$ [$(M+H)^+$] 304.1733, found 304.1740.

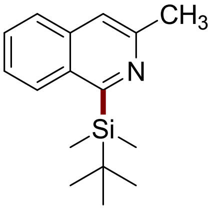

1-(*tert*-butyldimethylsilyl)-3-methylisoquinoline (3b11): According to the general procedure A, 3-methylisoquinoline (0.5 mmol, 1 equiv.), *tert*-butyldimethylsilane (290 mg, 2.5 mmol, 5 equiv.), $Na_2S_2O_8$ (238 mg, 1.0 mmol, 2.0 equiv.), $Ir(ppy)_2(dtbpy)PF_6$ (4.6 mg, 0.005 mmol, 0.01 equiv.) and 5 mL DMSO/DCE (1:1) (0.1 M) were used. The product was isolated by flash chromatography (2% ethyl acetate/hexane) as a colorless oil (90 mg, 70 %). And according to the general procedure B, the product was obtained (66 mg, 51 %). ^1H NMR (400 MHz, $CDCl_3$) δ 8.19 (d, J = 8.4 Hz, 1H), 7.70 (d, J = 8.2 Hz, 1H), 7.56 (t, J = 7.5 Hz, 1H), 7.46 (t, J = 7.5 Hz, 1H), 7.36 (s, 1H), 2.70 (s, 3H), 0.98 (s, 9H), 0.53 (s, 6H); ^{13}C NMR (100 MHz, $CDCl_3$) δ 169.37, 150.80, 135.32, 132.03, 128.83, 128.30, 127.02, 125.24, 117.54, 27.06, 24.46, 17.91, -3.16; HRMS (ESI) Calcd. for $C_{16}H_{24}NSi$ [$(M+H)^+$] 258.1678, found 258.1676.

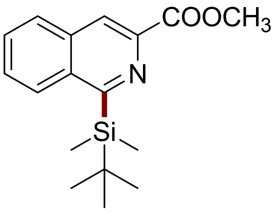

methyl 1-(*tert*-butyldimethylsilyl)isoquinoline-3-carboxylate (3b12): According to the general procedure A, methyl isoquinoline-3-carboxylate (0.5 mmol, 1 equiv.), *tert*-butyldimethylsilane (290 mg, 2.5 mmol, 5 equiv.), $Na_2S_2O_8$ (238 mg, 1.0 mmol, 2.0 equiv.), $Ir(ppy)_2(dtbpy)PF_6$ (4.6 mg, 0.005 mmol, 0.01 equiv.) and 5 mL DMSO/DCE (1:1) (0.1 M) were used. The product was isolated by flash chromatography (2% ethyl acetate/hexane) as a colorless oil (93 mg, 62 %). And according to the general procedure B, the product was obtained (83 mg, 55 %). ^1H NMR (400 MHz, $CDCl_3$) δ 8.44 (s, 1H), 8.33 – 8.22 (m, 1H), 7.93 (dd, J = 6.6, 2.8 Hz, 1H), 7.76 – 7.62 (m, 2H), 4.02 (s, 3H), 1.00 (s, 9H), 0.57 (s, 6H); ^{13}C NMR (100 MHz, $CDCl_3$) δ 170.98, 167.04, 141.05, 134.86, 134.23, 129.70, 129.07, 128.57, 128.41, 123.07, 52.46, 27.00, 17.94, -3.39; HRMS (ESI) Calcd. for $C_{17}H_{24}NO_2Si$

[(M+H)+] 302.1576, found 302.1567.

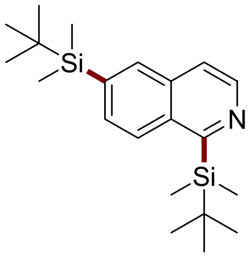

1,6-bis(*tert*-butyldimethylsilyl)isoquinoline (3b13'): According to the general procedure A, isoquinoline (0.5 mmol, 1 equiv.), *tert*-butyldimethylsilane (72 mg, 2.5 mmol, 5 equiv.), $Na_2S_2O_8$ (238 mg, 1.0 mmol, 2.0 equiv.), $Ir(ppy)_2(dtbpy)PF_6$ (4.6 mg, 0.005 mmol, 0.01 equiv.) and 5 mL DMSO/DCE (1:1) (0.1 M) were used. The product was isolated by flash chromatography (2% ethyl acetate/hexane) as a colorless solid (107 mg, 60 %). ^1H NMR (400 MHz, $CDCl_3$) δ 8.72 (d, J = 5.6 Hz, 1H), 8.19 (d, J = 8.4 Hz, 1H), 7.94 (s, 1H), 7.68 (dd, J = 8.4, 1.1 Hz, 1H), 7.55 (d, J = 5.6 Hz, 1H), 0.97 (s, 9H), 0.92 (s, 9H), 0.55 (s, 6H), 0.37 (s, 6H); ^{13}C NMR (100 MHz, $CDCl_3$) δ 169.99, 142.58, 134.61, 133.94, 133.49, 131.48, 126.91, 119.90, 27.11, 26.51, 18.00, 17.05, -3.06, -6.18. HRMS (ESI) Calcd. for $C_{16}H_{24}NOSi$ [$(M+H)^+$] 358.2381, found 358.2387.

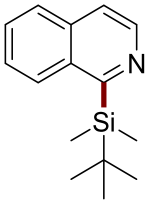

1-(*tert*-butyldimethylsilyl)isoquinoline (3b13): According to the general procedure B, isoquinoline (0.5 mmol, 1 equiv.), *tert*-butyldimethylsilane (580 mg, 5 mmol, 10 equiv., 3 batches), triisopropylsilanethiol (0.6 mmol, 1.2 equiv.), $Ir(ppy)_3$ (1.6 mg, 0.0025 mmol, 0.005 equiv.) and 2 mL DMA/DCE (1:1) (0.25 M) were used. The product was isolated by flash chromatography (2% ethyl acetate/hexane) as a colorless solid (87 mg, 71 %). ^1H NMR (400 MHz, $CDCl_3$) δ 8.72 (d, J = 5.6 Hz, 1H), 8.26 (d, J = 8.4 Hz, 1H), 7.80 (d, J = 8.2 Hz, 1H), 7.63 (dd, J = 7.9, 7.0 Hz, 1H), 7.60 – 7.51 (m, 2H), 0.97 (s, 9H), 0.57 (s, 6H); ^{13}C NMR (100 MHz, $CDCl_3$) δ 170.17, 142.50, 134.52, 133.98, 129.16, 128.63, 127.55, 126.35, 119.86, 27.08, 17.98, -3.04; HRMS (ESI) Calcd. for $C_{15}H_{22}NSi$ [$(M+H)^+$] 244.1522, found 244.1509.

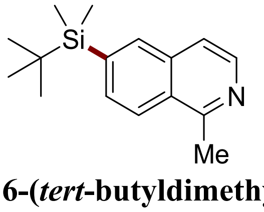

6-(*tert*-butyldimethylsilyl)-1-methylisoquinoline (3b14): According to the general procedure A, 1-methylisoquinoline (0.5 mmol, 1 equiv.), *tert*-butyldimethylsilane (290 mg, 2.5 mmol, 5 equiv.), $Na_2S_2O_8$ (238 mg, 1.0 mmol, 2.0 equiv.), $Ir(ppy)_2(dtbpy)PF_6$ (4.6 mg, 0.005 mmol, 0.01 equiv.) and 5 mL DMSO/DCE (1:1) (0.1 M) were used. The product was isolated by flash chromatography (2% ethyl acetate/hexane) as a colorless oil (45 mg, 35%). ^1H NMR (400 MHz, $CDCl_3$) δ 8.40 (d, J = 5.8 Hz, 1H), 8.07 (d, J = 8.3 Hz, 1H), 7.95 (s, 1H), 7.72 (dd, J = 8.3, 0.9 Hz, 1H), 7.52 (d, J = 5.7 Hz, 1H), 2.96 (s, 3H), 0.91 (s, 9H), 0.37 (s, 6H); ^{13}C NMR (100 MHz, $CDCl_3$) δ 158.47, 141.40, 134.91, 134.14, 132.27, 123.94, 119.41, 29.69, 26.47, 17.01, -6.21; HRMS (ESI) Calcd. for $C_{16}H_{24}NSi$ [$(M+H)^+$] 258.1678, found 258.1687.

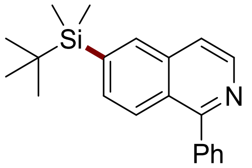

6-(*tert*-butyldimethylsilyl)-1-phenylisoquinoline (3b15): According to the general procedure A, 1-phenylisoquinoline (0.5 mmol, 1 equiv.), *tert*-butyldimethylsilane (290 mg, 2.5 mmol, 5 equiv.), $Na_2S_2O_8$ (238 mg, 1.0 mmol, 2.0 equiv.), $Ir(ppy)_2(dtbpy)PF_6$ (4.6 mg, 0.005 mmol, 0.01 equiv.) and 5 mL DMSO/DCE (1:1) (0.1 M) were used. The product was isolated by flash chromatography (2% ethyl acetate/hexane) as a colorless oil (98 mg, 61 %). ^1H NMR (400 MHz, $CDCl_3$) δ 8.62 (d, J = 5.7 Hz, 1H), 8.10 – 7.99 (m, 2H), 7.70 (dd, J = 7.9, 1.4 Hz, 2H), 7.66 (dd, J = 6.6, 3.1 Hz, 2H), 7.57 – 7.47 (m, 3H), 0.92 (s, 9H), 0.38 (s, 6H); ^{13}C NMR (100 MHz, $CDCl_3$) δ 160.57, 142.13, 141.26, 139.45, 135.83, 133.90, 132.31, 129.89, 128.55, 128.30, 126.67, 125.76, 119.95, 26.48, 17.03, -6.20; HRMS (ESI) Calcd. for $C_{21}H_{26}NSi$ [$(M+H)^+$] 320.1835, found 320.1837.

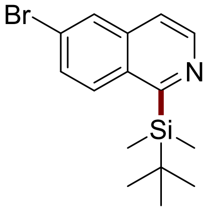

6-bromo-1-(*tert*-butyldimethylsilyl)isoquinoline (3b16): According to the general procedure B, 6-bromoisoquinoline (0.5 mmol, 1 equiv.), *tert*-butyldimethylsilane (580 mg, 5 mmol, 10 equiv., 3 batches), triisopropylsilanethiol (0.6 mmol, 1.2 equiv.), $Ir(ppy)_3$ (1.6 mg, 0.0025 mmol, 0.005 equiv.) and 2 mL DMA/DCE (1:1) (0.25 M) were used. The product was isolated by flash chromatography (2% ethyl acetate/hexane) as a colorless solid (103 mg, 64 %). ^1H NMR (400 MHz, $CDCl_3$) δ 8.72 (d, J = 5.7 Hz, 1H), 8.11 (d, J = 9.0 Hz, 1H), 7.97 (d, J = 2.0 Hz, 1H), 7.63 (dd, J = 9.0, 2.0 Hz, 1H), 7.47 (d, J = 5.6 Hz, 1H), 0.94 (s, 9H), 0.54 (s, 6H); ^{13}C NMR (100 MHz, $CDCl_3$) δ 170.57, 143.38, 135.77, 132.26, 130.29, 129.93, 129.64, 123.79, 118.71, 27.00, 17.93, -3.13; HRMS (ESI) Calcd. for $C_{15}H_{21}BrNSi$ [$(M+H)^+$] 322.0627, found 322.0628.

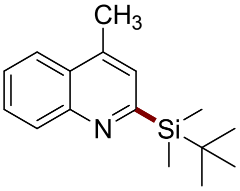

2-(*tert*-butyldimethylsilyl)-4-methylquinoline (3c): According to the general procedure A, 4-methylquinoline (0.5 mmol, 1 equiv.), *tert*-butyldimethylsilane (290 mg, 2.5 mmol, 5 equiv.), $Na_2S_2O_8$ (238 mg, 1.0 mmol, 2.0 equiv.), $Ir(ppy)_2(dtbpy)PF_6$ (4.6 mg, 0.005 mmol, 0.01 equiv.) and 5 mL DMSO/DCE (1:1) (0.1 M) were used. The product was isolated by flash chromatography (2% ethyl acetate/hexane) as a colorless oil (94 mg, 73 %). ^1H NMR (400 MHz, $CDCl_3$) δ 8.21 (dd, J = 8.4, 0.5 Hz, 1H), 7.99 (dd, J = 8.3, 0.7 Hz, 1H), 7.70 (ddd, J = 8.3, 6.9, 1.3 Hz, 1H), 7.55 (ddd, J = 8.2, 6.9, 1.2 Hz, 1H), 7.44 (d, J = 0.6 Hz, 1H), 2.71 (d, J = 0.8 Hz, 3H), 1.02 (s, 9H), 0.45 (s, 6H); ^{13}C NMR (100 MHz, $CDCl_3$) δ 168.52, 148.37, 140.21, 130.82, 128.38, 127.26, 126.97, 126.03, 123.59, 26.73, 18.61, 17.21, -6.15; HRMS (ESI) Calcd. for $C_{16}H_{24}NSi$ [$(M+H)^+$] 258.1678, found 258.1668.

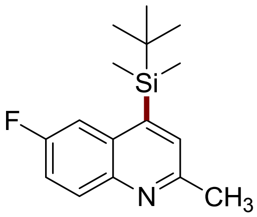

4-(*tert*-butyldimethylsilyl)-6-fluoro-2-methylquinoline (3d): According to the general procedure A, 6-fluoro-2-methylquinoline (0.5 mmol, 1 equiv.), *tert*-butyldimethylsilane (290 mg, 2.5 mmol, 5 equiv.), $Na_2S_2O_8$ (238 mg, 1.0 mmol, 2.0 equiv.), $Ir(ppy)_2(dtbpy)PF_6$ (4.6 mg, 0.005 mmol, 0.01 equiv.) and 5 mL DMSO/DCE (1:1) (0.1 M) were used. The product was isolated by flash chromatography (2% ethyl acetate/hexane) as a colorless solid (97 mg, 70 %). ^{1}H NMR (400 MHz, $CDCl_3$) δ 8.01 (dd, *J* = 9.2, 5.8 Hz, 1H), 7.62 (dd, *J* = 10.6, 2.8 Hz, 1H), 7.46 – 7.36 (m, 2H), 2.72 (s, 3H), 0.93 (s, 9H), 0.50 (s, 6H).^{13}C NMR (100 MHz, $CDCl_3$) δ 160.70, 158.26, 156.42, 156.40, 146.31, 146.25, 144.39 (s), 131.60, 131.51, 131.12, 131.03, 130.92, 118.65, 118.40, 112.28, 112.06, 26.96, 25.07, 17.57, -3.53; HRMS (ESI) Calcd. for $C_{16}H_{23}FNSi$ [$(M+H)^+$] 276.1584, found 276.1589.

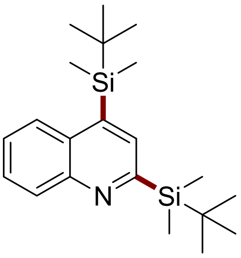

2,4-bis(*tert*-butyldimethylsilyl)quinolone (3e'): According to the general procedure A, quinoline (0.5 mmol, 1 equiv.), *tert*-butyldimethylsilane (290 mg, 2.5 mmol, 5 equiv.), $Na_2S_2O_8$ (238 mg, 1.0 mmol, 2.0 equiv.), $Ir(ppy)_2(dtbpy)PF_6$ (4.6 mg, 0.005 mmol, 0.01 equiv.) and 5 mL DMSO/DCE (1:1) (0.1 M) were used. The product was isolated by flash chromatography (2% ethyl acetate/hexane) as a colorless oil (123 mg, 75 %, mixture of **3e'** and **3e**). ^{1}H NMR (400 MHz, $CDCl_3$) δ 8.21 (t, *J* = 7.6 Hz, 1.3 H), 8.08 – 8.00 (m, 1.3 H), 7.79 (s, 0.3 H), 7.76 (s, 1H), 7.73 – 7.63 (m, 1.3 H), 7.61 (d, *J* = 8.2 Hz, 0.3 H), 7.56 – 7.46 (m, 1.3 H), 1.00 (s, 12H), 0.96 (s, 11 H), 0.54 (s, 6 H), 0.45 (s, 8 H); ^{13}C NMR (100 MHz, $CDCl_3$) δ 166.54, 148.11, 141.84, 134.27, 132.61, 131.34, 131.21, 130.19, 128.91, 128.77, 128.04, 127.61, 127.15, 126.27, 126.05, 125.71, 26.98, 26.68, 17.70, 17.21, -3.33, -6.17, -6.23. HRMS (ESI) Calcd. for $C_{21}H_{36}NSi_2$ [$(M+H)^+$] 358.2381, found 358.2387.

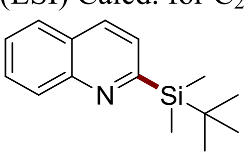

2-(*tert*-butyldimethylsilyl)quinolone (3e): According to the general procedure B, quinoline (0.5 mmol, 1 equiv.), *tert*-butyldimethylsilane (580 mg, 5 mmol, 10 equiv., 3 batches), triisopropylsilanethiol (0.6 mmol, 1.2 equiv.), $Ir(ppy)_3$ (1.6 mg, 0.0025 mmol, 0.005 equiv.) and 2 mL DMA/DCE (1:1) (0.25 M) were used. The product was isolated by flash chromatography (2% ethyl acetate/hexane) as a colorless oil (81 mg, 66 %). ^{1}H NMR (400 MHz, $CDCl_3$) δ 8.17 (d, *J* = 8.4 Hz, 1H), 8.02 (d, *J* = 8.2 Hz, 1H), 7.77 (d, *J* = 8.1 Hz, 1H), 7.72 – 7.66 (m, 1H), 7.59 (d, *J* = 8.2 Hz, 1H), 7.53 – 7.48 (m, 1H), 0.97 (s, 9H), 0.41 (s, 6H); ^{13}C NMR (100 MHz, $CDCl_3$) δ 169.05, 148.73, 132.60, 130.20, 128.77, 127.61, 127.16, 126.26, 126.06, 26.67, 17.20, -6.18; HRMS (ESI) Calcd. for $C_{15}H_{22}NSi$ [$(M+H)^+$] 244.1522, found 244.1526.

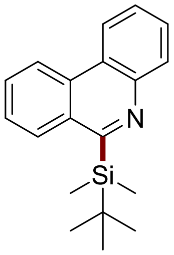

6-(*tert*-butyldimethylsilyl)phenanthridine (3f): According to the general procedure A, phenanthridine (0.5 mmol, 1 equiv.) , *tert*-butyldimethylsilane (290 mg, 2.5 mmol, 5 equiv.), $Na_2S_2O_8$ (238 mg, 1.0 mmol, 2.0 equiv.), $Ir(ppy)_2(dtbpy)PF_6$ (4.6 mg, 0.005 mmol, 0.01 equiv.) and 5 mL DMSO/DCE (1:1) (0.1 M) were used. The product was isolated by flash chromatography (2% ethyl acetate/hexane) as a colorless solid (120 mg, 82 %). ^{1}H NMR (400 MHz, $CDCl_3$) δ 8.66 (d, *J* = 8.3 Hz, 1H), 8.57 (d, *J* = 8.1 Hz, 1H), 8.34 (d, *J* = 8.1 Hz, 1H), 8.24 (d, *J* = 8.0 Hz, 1H), 7.84 – 7.76 (m, 1H), 7.75 – 7.70 (m, 1H), 7.66 (ddd, *J* = 8.0, 7.1, 1.1 Hz, 2H), 1.03 (s, 9H), 0.61 (s, 6H). ^{13}C NMR (100 MHz, $CDCl_3$) δ 171.63, 130.88, 130.69, 129.56, 129.49, 128.16, 126.97, 126.56, 123.06, 122.27, 121.78, 27.19, 18.10, -3.07; HRMS (ESI) Calcd. for $C_{19}H_{24}NSi$ [$(M+H)^+$] 294.1678, found 294.1676.

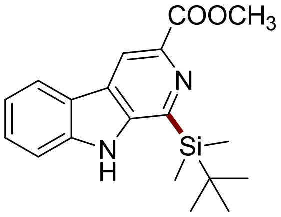

Methyl 1-(*tert*-butyldimethylsilyl)-9H-pyrido[3,4-b]indole-3-carboxylate (3g): According to the general procedure A, Methyl-9H-pyrido[3,4-b]indole-3-carboxylate (this substrate was prepared according to known procedure [4]) (0.5 mmol, 1 equiv.), *tert*-butyldimethylsilane (290 mg, 2.5 mmol, 5 equiv.), $Na_2S_2O_8$ (238 mg, 1.0 mmol, 2.0 equiv.), $Ir(ppy)_2(dtbpy)PF_6$ (4.6 mg, 0.005 mmol, 0.01 equiv.) and 5 mL DMSO/DCE (1:1) (0.1 M) were used. The product was isolated by flash chromatography (10% ethyl acetate/hexane) as a colorless solid (111mg, 65 %). ^{1}H NMR (400 MHz, $CDCl_3$) δ 8.78 (s, 1H), 8.35 (s, 1H), 8.19 (d, *J* = 7.9 Hz, 1H), 7.64 – 7.52 (m, 2H), 7.34 (ddd, *J* = 8.0, 6.4, 1.7 Hz, 1H), 4.02 (s, 3H), 1.00 (s, 9H), 0.59 (s, 6H); ^{13}C NMR (100 MHz, $CDCl_3$) δ 167.23, 148.86, 142.80, 140.06, 128.63, 126.68, 121.89, 121.66, 120.83, 116.86, 111.67, 52.36, 26.64, 17.83, -5.05; HRMS (ESI) Calcd. for $C_{19}H_{25}N_2O_2Si$ [$(M+H)^+$] 341.1685, found 341.1683.

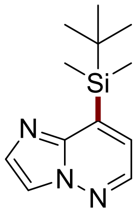

8-(*tert*-butyldimethylsilyl)imidazo[1,2-*b*]pyridazine (3h): According to the general procedure A, imidazo[1,2-*b*]pyridazine (0.5 mmol, 1 equiv.), *tert*-butyldimethylsilane (290 mg, 2.5 mmol, 5 equiv.), $Na_2S_2O_8$ (238 mg, 1.0 mmol, 2.0 equiv.), $Ir(ppy)_2(dtbpy)PF_6$ (4.6 mg, 0.005 mmol, 0.01 equiv.) and 5 mL DMSO/DCE (1:1) (0.1 M) were used. The product was isolated by flash chromatography (10 % ethyl acetate/hexane) as a colorless solid (41 mg, 35 %). ^{1}H NMR (400 MHz, $CDCl_3$) δ 8.21 (d, *J* = 4.3 Hz, 1H), 7.92 (d, *J* = 1.2 Hz, 1H), 7.78 (d, *J* = 1.1 Hz, 1H), 7.04 (d, *J* = 4.3 Hz, 1H), 0.95 (s, 9H),

0.48 (s, 6H); ^{13}C NMR (100 MHz, $CDCl_3$) δ 142.16, 141.80, 138.56, 133.20, 123.30, 115.73, 26.88, 17.33, -5.47; HRMS (ESI) Calcd. for $C_{12}H_{20}N_3Si$ [$(M+H)^+$] 234.1421, found 234.1427.

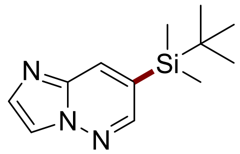

7-(*tert*-butyldimethylsilyl)imidazo[1,2-*b*]pyridazine (3h'): According to the general procedure A, imidazo[1,2-*b*]pyridazine (0.5 mmol, 1 equiv.), *tert*-butyldimethylsilane (290 mg, 2.5 mmol, 5 equiv.), $Na_2S_2O_8$ (238 mg, 1.0 mmol, 2.0 equiv.), $Ir(ppy)_2(dtbpy)PF_6$ (4.6 mg, 0.005 mmol, 0.01 equiv.) and 5 mL DMSO/DCE (1:1) (0.1 M) were used. The product was isolated by flash chromatography (20 % ethyl acetate/hexane) as a colorless solid (20 mg, 17 %). ^{1}H NMR (400 MHz, $CDCl_3$) δ 8.28 (d, J = 1.4 Hz, 1H), 8.05 (d, J = 0.9 Hz, 1H), 7.95 (s, 1H), 7.76 (d, J = 0.7 Hz, 1H), 0.91 (s, 9H), 0.33 (s, 6H); ^{13}C NMR (100 MHz, $CDCl_3$) δ 146.56, 138.72, 133.67, 132.19, 126.61, 116.32, 26.24, 16.96, -6.53; HRMS (ESI) Calcd. for $C_{12}H_{20}N_3Si$ [$(M+H)^+$] 234.1421, found 234.1427.

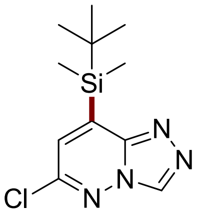

8-(*tert*-butyldimethylsilyl)-6-chloro-[1,2,4]triazolo[4,3-*b*]pyridazine (3i): According to the general procedure A, 6-chloro-[1,2,4]triazolo[4,3-*b*]pyridazine (0.5 mmol, 1 equiv.), *tert*-butyldimethylsilane (290 mg, 2.5 mmol, 5 equiv.), $Na_2S_2O_8$ (238 mg, 1.0 mmol, 2.0 equiv.), $Ir(ppy)_2(dtbpy)PF_6$ (4.6 mg, 0.005 mmol, 0.01 equiv.) and 5 mL DMSO/DCE (1:1) (0.1 M) were used. The product was isolated by flash chromatography (20 % ethyl acetate/hexane) as a colorless solid (72 mg, 61 %). ^{1}H NMR (400 MHz, $CDCl_3$) δ 9.00 (s, 1H), 7.07 (s, 1H), 0.98 (s, 9H), 0.51 (s, 6H); ^{13}C NMR (100 MHz, $CDCl_3$) δ 149.12, 145.49, 142.08, 137.92, 128.52, 26.76, 17.30, -5.75; HRMS (ESI) Calcd. for $C_{11}H_{18}ClN_4Si$ [$(M+H)^+$] 269.0984, found 269.0988.

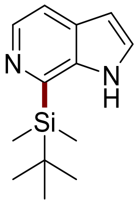

7-(*tert*-butyldimethylsilyl)-1H-pyrrolo[2,3-c]pyridine (3j): According to the general procedure B, 1H-pyrrolo[2,3-c]pyridine (0.5 mmol, 1 equiv.), *tert*-butyldimethylsilane (580 mg, 5 mmol, 10 equiv., 3 batches), triisopropylsilanethiol (0.6 mmol, 1.2 equiv.), $Ir(ppy)_3$ (1.6 mg, 0.0025 mmol, 0.005 equiv.) and 2 mL DMA/DCE (1:1) (0.25 M) were used. The product was isolated by flash chromatography (10% ethyl acetate/hexane) as a colorless solid (68 mg, 58%). ^{1}H NMR (400 MHz, $CDCl_3$) δ 8.47 (d, J = 5.4 Hz, 1H), 8.39 (s, 1H), 7.50 (d, J = 5.4 Hz, 1H), 7.35 (d, J = 3.1 Hz, 1H), 6.55 (d, J = 3.1 Hz, 1H), 0.95 (s, 9H), 0.52 (s, 6H); ^{13}C NMR (100 MHz, $CDCl_3$) δ 148.88, 139.66, 139.32, 130.46, 127.24, 114.64, 101.69, 26.59, 17.84, -5.01; HRMS (ESI) Calcd. for $C_{13}H_{21}N_2Si$ [$(M+H)^+$] 233.1474, found 233.1477.

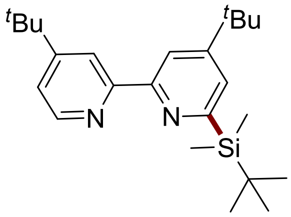

4,4'-di-*tert*-butyl-6-(*tert*-butyldimethylsilyl)-2,2'-bipyridine (3k): According to the general procedure A, 4,4'-di-*tert*-butyl-2,2'-bipyridine (0.5 mmol, 1 equiv.), *tert*-butyldimethylsilane (290 mg, 2.5 mmol, 5 equiv.), $Na_2S_2O_8$ (238 mg, 1.0 mmol, 2.0 equiv.), $Ir(ppy)_2(dtbpy)PF_6$ (4.6 mg, 0.005 mmol, 0.01 equiv.) and 5 mL DMSO/DCE (1:1) (0.1 M) were used. The product was isolated by flash chromatography (10% ethyl acetate/hexane) as a colorless solid (103 mg, 54 %). ^{1}H NMR (400 MHz, $CDCl_3$) δ 8.67 (d, J = 1.6 Hz, 1H), 8.58 (d, J = 5.2 Hz, 1H), 8.33 (d, J = 1.9 Hz, 1H), 7.49 (d, J = 1.9 Hz, 1H), 7.28 (dd, J = 5.3, 2.0 Hz, 1H), 1.38 (d, J = 5.0 Hz, 18H), 1.01 (s, 9H), 0.37 (s, 6H).^{13}C NMR (100 MHz, $CDCl_3$) δ 165.24, 160.51, 158.06, 157.13, 155.46, 148.76, 126.39, 120.41, 118.64, 116.59, 34.84, 34.80, 30.70, 30.51, 26.65, 16.95, -6.10; HRMS (ESI) Calcd. for $C_{24}H_{39}N_2Si$ [$(M+H)^+$] 383.2883, found 383.2887.

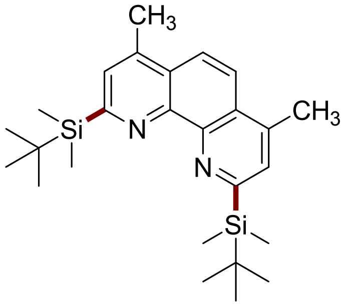

2,9-bis(*tert*-butyldimethylsilyl)-4,7-dimethyl-1,10-phenanthroline (3l'): According to the general procedure A, 4,7-dimethyl-1,10-phenanthroline (0.5 mmol, 1 equiv.), *tert*-butyldimethylsilane (290 mg, 2.5 mmol, 5 equiv.), $Na_2S_2O_8$ (238 mg, 1.0 mmol, 2.0 equiv.), $Ir(ppy)_2(dtbpy)PF_6$ (4.6 mg, 0.005 mmol, 0.01 equiv.) and 5 mL DMSO/DCE (1:1) (0.1 M) were used. The product was isolated by flash chromatography (10% ethyl acetate/hexane) as a colorless solid (135 mg,62 %). ^{1}H NMR (400 MHz, $CDCl_3$) δ 7.97 (s, 2H), 7.60 (s, 2H), 2.75 (s, 6H), 1.05 (s, 18H), 0.46 (s, 12H); ^{13}C NMR (100 MHz, $CDCl_3$) δ 167.93, 146.32, 140.09, 128.95, 126.71, 122.12, 26.84, 19.03, 17.17, -6.02; HRMS (ESI) Calcd. for $C_{26}H_{41}N_2Si_2$ [$(M+H)^+$] 437.2808, found 437.2811.

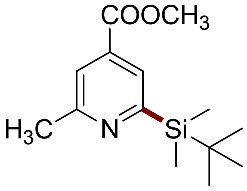

methyl 2-(*tert*-butyldimethylsilyl)-6-methylisonicotinate (3m): According to the general procedure A, methyl 6-methylisonicotinate (0.5 mmol, 1 equiv.), *tert*-butyldimethylsilane (290 mg, 2.5 mmol, 5 equiv.), $Na_2S_2O_8$ (238 mg, 1.0 mmol, 2.0 equiv.), $Ir(ppy)_2(dtbpy)PF_6$ (4.6 mg, 0.005 mmol, 0.01 equiv.) and 5 mL DMSO/DCE (1:1) (0.1 M) were used. The product was isolated by flash chromatography (2% ethyl acetate/hexane) as a colorless oil (80 mg, 60 %). ^{1}H NMR (400 MHz, $CDCl_3$) δ 7.79 (d, J = 0.7 Hz, 1H), 7.57 (d, J = 1.3 Hz, 1H), 3.93 (s, 3H), 2.63 (s, 3H), 0.91 (s, 9H), 0.32 (s, 6H).^{13}C NMR (100 MHz, $CDCl_3$) δ 167.65, 166.83, 159.01, 134.83, 125.20, 120.89, 52.44, 26.58, 24.83, 16.89, -6.27; HRMS (ESI) Calcd. for $C_{14}H_{24}NO_2Si$ [$(M+H)^+$] 266.1576, found 266.1578.

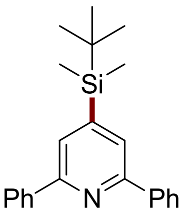

4-(*tert*-butyldimethylsilyl)-2,6-diphenylpyridine (3n): According to the general procedure A, 2,6-diphenylpyridine (0.5 mmol, 1 equiv.), *tert*-butyldimethylsilane (290 mg, 2.5 mmol, 5 equiv.), $Na_2S_2O_8$ (238 mg, 1.0 mmol, 2.0 equiv.), $Ir(ppy)_2(dtbpy)PF_6$ (4.6 mg, 0.005 mmol, 0.01 equiv.) and 5 mL DMSO/DCE (1:1) (0.1 M) were used. The product was isolated by flash chromatography (2% ethyl acetate/hexane) as a colorless oil (76 mg, 44 %). ^{1}H NMR (400 MHz, $CDCl_3$) δ 8.19 – 8.06 (m, 4H), 7.77 (s, 2H), 7.51 (t, *J* = 7.4 Hz, 4H), 7.43 (t, *J* = 7.3 Hz, 2H), 0.95 (s, 9H), 0.38 (s, 6H).^{13}C NMR (100 MHz, $CDCl_3$) δ 155.50, 149.22, 139.94, 128.78, 128.64, 127.14, 124.32, 26.43, 16.79, -6.46; HRMS (ESI) Calcd. for $C_{23}H_{28}NSi$ [(M+H)$^+$] 346.1986, found 346.1995.

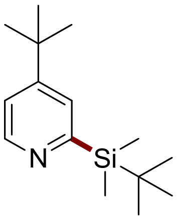

4-(*tert*-butyl)-2-(*tert*-butyldimethylsilyl)pyridine (3o): According to the general procedure A, 4-(*tert*-butyl)pyridine (0.5 mmol, 1 equiv.), *tert*-butyldimethylsilane (290 mg, 2.5 mmol, 5 equiv.), $Na_2S_2O_8$ (238 mg, 1.0 mmol, 2.0 equiv.), $Ir(ppy)_2(dtbpy)PF_6$ (4.6 mg, 0.005 mmol, 0.01 equiv.) and 5 mL DMSO/DCE (1:1) (0.1 M) were used. The product was isolated by flash chromatography (2% ethyl acetate/hexane) as a colorless oil (90 mg, 72 %). ^{1}H NMR (400 MHz, $CDCl_3$) δ 8.66 (dd, *J* = 5.3, 0.6 Hz, 1H), 7.47 (dd, *J* = 2.1, 0.7 Hz, 1H), 7.15 (dd, *J* = 5.3, 2.1 Hz, 1H), 1.28 (s, 9H), 0.89 (s, 9H), 0.31 (s, 6H); ^{13}C NMR (100 MHz, $CDCl_3$) δ 165.76, 156.84, 149.68, 126.81, 119.48, 34.36, 30.47, 26.50, 16.82, -6.33. HRMS (ESI) Calcd. for $C_{15}H_{28}NSi$ [(M+H)$^+$] 250.1986, found 250.1993.

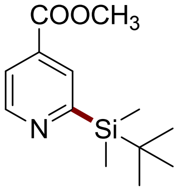

Methyl 2-(*tert*-butyldimethylsilyl)isonicotinate (3p): According to the general procedure A, 4-(tert-butyl)pyridine (0.5 mmol, 1 equiv.), *tert*-butyldimethylsilane (290 mg, 2.5 mmol, 5 equiv.), $Na_2S_2O_8$ (238 mg, 1.0 mmol, 2.0 equiv.), $Ir(ppy)_2(dtbpy)PF_6$ (4.6 mg, 0.005 mmol, 0.01 equiv.) and 5 mL DMSO/DCE (1:1) (0.1 M) were used. The product was isolated by flash chromatography (2% ethyl acetate/hexane) as a colorless oil (50 mg, 40 %). ^{1}H NMR (400 MHz, $CDCl_3$) δ 8.93 (dd, *J* = 5.0, 0.9 Hz, 1H), 8.00 (dd, *J* = 1.7, 1.0 Hz, 1H), 7.71 (dd, *J* = 5.0, 1.8 Hz, 1H), 3.94 (s, 3H), 0.90 (s, 9H), 0.34 (s, 6H); ^{13}C NMR (100 MHz, $CDCl_3$) δ 168.41, 166.39, 150.52, 134.68, 128.20, 121.39, 52.59, 26.48, 16.90, -6.36. HRMS (ESI) Calcd. for $C_{13}H_{22}NO_2Si$ [(M+H)$^+$] 252.1414, found 252.1417.

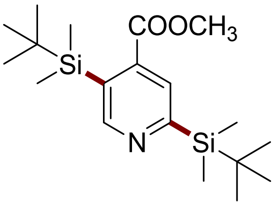

Methyl 2,5-bis(*tert*-butyldimethylsilyl)isonicotinate (3p'): According to the general procedure A, 4-(tert-butyl)pyridine (0.5 mmol, 1 equiv.), *tert*-butyldimethylsilane (290 mg, 2.5 mmol, 5 equiv.), $Na_2S_2O_8$ (238 mg, 1.0 mmol, 2.0 equiv.), $Ir(ppy)_2(dtbpy)PF_6$ (4.6 mg, 0.005 mmol, 0.01 equiv.) and 5 mL DMSO/DCE (1:1) (0.1 M) were used. The product was isolated by flash chromatography (2% ethyl acetate/hexane) as a colorless oil (46 mg, 25 %). ^{1}H NMR (400 MHz, $CDCl_3$) δ 9.01 (d, J = 0.9 Hz, 1H), 7.65 (d, J = 0.9 Hz, 1H), 3.89 (s, 3H), 0.94 (s, 9H), 0.92 (s, 9H), 0.33 (s, 6H), 0.29 (s, 6H); ^{13}C NMR (100 MHz, $CDCl_3$) δ 169.22, 167.48, 156.52, 143.00, 129.09, 127.79, 52.43, 27.21, 26.54, 17.95, 16.98, -3.89, -6.44. HRMS (ESI) Calcd. for $C_{19}H_{36}NO_2Si_2$ [(M+H)$^+$] 366.2279, found 366.2283.

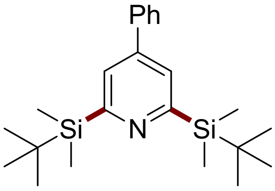

2,6-bis(*tert*-butyldimethylsilyl)-4-phenylpyridine (3q'): According to the general procedure A, 4-phenylpyridine (0.5 mmol, 1 equiv.), *tert*-butyldimethylsilane (290 mg, 2.5 mmol, 5 equiv.), $Na_2S_2O_8$ (238 mg, 1.0 mmol, 2.0 equiv.), $Ir(ppy)_2(dtbpy)PF_6$ (4.6 mg, 0.005 mmol, 0.01 equiv.) and 5 mL DMSO/DCE (1:1) (0.1 M) were used. The product was isolated by flash chromatography (2% ethyl acetate/hexane) as a colorless oil (125 mg, 65 %). ^{1}H NMR (400 MHz, $CDCl_3$) δ 7.64 – 7.58 (m, 2H), 7.55 (s, 2H), 7.48 (t, J = 7.4 Hz, 2H), 7.41 (t, J = 7.3 Hz, 1H), 0.94 (s, 18H), 0.33 (s, 12H); ^{13}C NMR (100 MHz, $CDCl_3$) δ 128.97, 128.37, 127.24, 126.22, 26.71, 16.96, -6.17; HRMS (ESI) Calcd. for $C_{23}H_{38}NSi_2$ [(M+H)$^+$] 384.2543, found 384.2541.

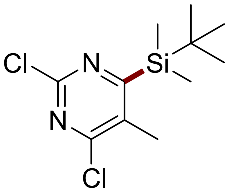

4-(*tert*-butyldimethylsilyl)-2,6-dichloro-5-methylpyrimidine (3r): According to the general procedure A, 2,4-dichloro-5-methylpyrimidine (0.5 mmol, 1 equiv.), *tert*-butyldimethylsilane (290 mg, 2.5 mmol, 5 equiv.), $Na_2S_2O_8$ (238 mg, 1.0 mmol, 2.0 equiv.), $Ir(ppy)_2(dtbpy)PF_6$ (4.6 mg, 0.005 mmol, 0.01 equiv.) and 5 mL DMSO/DCE (1:1) (0.1 M) were used. The product was isolated by flash chromatography (2 % ethyl acetate/hexane) as a colorless solid (59 mg, 43 %). ^{1}H NMR (400 MHz, $CDCl_3$) δ 2.41 (s, 3H), 0.95 (s, 9H), 0.39 (s, 6H); ^{13}C NMR (100 MHz, $CDCl_3$) δ 180.59, 162.17, 156.50, 134.59, 26.61, 17.99, 17.19, -4.16; HRMS (ESI) Calcd. for $C_{11}H_{19}Cl_2N_2Si$ [(M+H)$^+$] 277.0689, found 277.0693.

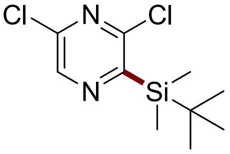

2-(*tert*-butyldimethylsilyl)-3,5-dichloropyrazine (3s): According to the general procedure A, 2,6-dichloropyrazine (0.5 mmol, 1 equiv.), *tert*-butyldimethylsilane (290 mg, 2.5 mmol, 5 equiv.), $Na_2S_2O_8$ (238 mg, 1.0 mmol, 2.0 equiv.), $Ir(ppy)_2(dtbpy)PF_6$ (4.6 mg, 0.005 mmol, 0.01 equiv.) and 5 mL DMSO/DCE (1:1) (0.1 M) were used. The product was isolated by flash chromatography (1 % ethyl

acetate/hexane) as a colorless solid (51 mg, 39 %). ^{1}H NMR (400 MHz, $CDCl_3$) δ 8.66 (s, 1H), 0.95 (s, 9H), 0.42 (s, 6H); ^{13}C NMR (100 MHz, $CDCl_3$) δ 159.02, 153.38, 146.69, 142.32, 26.68, 18.10, -4.80; HRMS (ESI) Calcd. for $C_{10}H_{17}Cl_2N_2Si$ [$(M+H)^+$] 263.0533, found 263.0535.

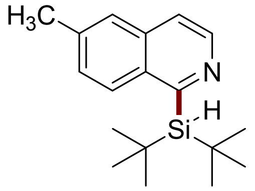

1-(di-*tert*-butylsilyl)-6-methylisoquinoline (3t): According to the general procedure A, 6-methylisoquinoline (0.5 mmol, 1 equiv.), di-*tert*-butylsilane (290 mg, 2.5 mmol, 5 equiv.), $Na_2S_2O_8$ (238 mg, 1.0 mmol, 2.0 equiv.), Ir(ppy)$_2$(dtbpy)PF_6 (4.6 mg, 0.005 mmol, 0.01 equiv.) and 5 mL DMSO/DCE (1:1) (0.1 M) were used. The product was isolated by flash chromatography (2% ethyl acetate/hexane) as a colorless oil (73 mg, 51%). ^{1}H NMR (400 MHz, $CDCl_3$) δ 8.65 (d, J = 5.6 Hz, 1H), 8.44 (d, J = 8.4 Hz, 1H), 7.55 (s, 1H), 7.47 (d, J = 5.6 Hz, 1H), 7.40 (dd, J = 8.6, 1.3 Hz, 1H), 4.63 (s, 1H), 2.53 (s, 3H), 1.09 (s, 18H); ^{13}C NMR (100 MHz, $CDCl_3$) δ 168.66, 142.57, 139.43, 134.42, 128.83, 128.56, 126.14, 119.21, 28.92, 21.83, 19.53. HRMS (ESI) Calcd. for $C_{18}H_{28}NSi$ [$(M+H)^+$] 286.1986, found 286.1989.

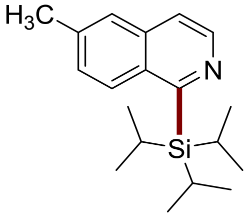

6-**methyl-1-(triisopropylsilyl)isoquinoline (3u):** According to the general procedure A, 6-methylisoquinoline (0.5 mmol, 1 equiv.), triisopropylsilane (290 mg, 2.5 mmol, 5 equiv.), $Na_2S_2O_8$ (238 mg, 1.0 mmol, 2.0 equiv.), Ir(ppy)$_2$(dtbpy)PF_6 (4.6 mg, 0.005 mmol, 0.01 equiv.) and 5 mL DMSO/DCE (1:1) (0.1 M) were used. The product was isolated by flash chromatography (2% ethyl acetate/hexane) as a colorless oil (115 mg, 77%). ^{1}H NMR (400 MHz, $CDCl_3$) δ 8.66 (d, J = 5.6 Hz, 1H), 8.07 (d, J = 8.6 Hz, 1H), 7.56 (s, 1H), 7.46 (d, J = 5.6 Hz, 1H), 7.39 (dd, J = 8.6, 1.6 Hz, 1H), 2.53 (s, 3H), 1.80 - 1.69 (m, 3H), 1.15 (d, J = 7.6 Hz, 18 H); ^{13}C NMR (100 MHz, $CDCl_3$) δ 168.54, 142.70, 139.10, 134.83, 132.83, 128.56, 128.17, 126.48, 119.19, 21.75, 19.03, 13.04. HRMS (ESI) Calcd. for $C_{19}H_{30}NSi$ [$(M+H)^+$] 300.2142, found 300.2144.

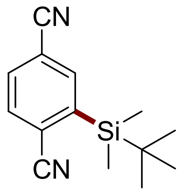

2-(*tert*-butyldimethylsilyl)terephthalonitrile (5a): According to the general procedure A, terephthalonitrile (0.5 mmol, 1 equiv.), *tert*-butyldimethylsilane (290 mg, 2.5 mmol, 5 equiv.), $Na_2S_2O_8$ (238 mg, 1.0 mmol, 2.0 equiv.), Ir(ppy)$_2$(dtbpy)PF_6 (4.6 mg, 0.005 mmol, 0.01 equiv.) and 5 mL DMSO/DCE (1:1) (0.1 M) were used. The product was isolated by flash chromatography (2% ethyl acetate/hexane) as a colorless solid (86 mg, 71%). And according to the general procedure B, the product was obtained (79 mg, 65%). ^{1}H NMR (400 MHz, $CDCl_3$) δ 7.84 (d, J = 1.3 Hz, 1H), 7.80 (d, J = 8.0 Hz, 1H), 7.73 (dd, J = 8.0, 1.6 Hz, 1H), 0.92 (s, 9H), 0.48 (s, 6H); ^{13}C NMR (100 MHz, $CDCl_3$)

δ 144.45, 139.23, 134.11, 132.18, 121.87, 118.96, 117.53, 115.25, 26.40, 17.85, -5.25; HRMS (ESI) Calcd. for $C_{14}H_{19}N_2Si$ [$(M+H)^+$] 243.1318, found 243.1322.

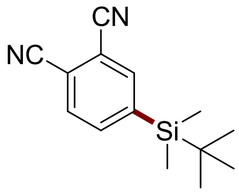

4-(*tert*-butyldimethylsilyl)phthalonitrile (5d): According to the general procedure A, phthalonitrile (0.5 mmol, 1 equiv.), *tert*-butyldimethylsilane (290 mg, 2.5 mmol, 5 equiv.), $Na_2S_2O_8$ (238 mg, 1.0 mmol, 2.0 equiv.), $Ir(ppy)_2(dtbpy)PF_6$ (4.6 mg, 0.005 mmol, 0.01 equiv.) and 5 mL DMSO/DCE (1:1) (0.1 M) were used. The product was isolated by flash chromatography (2% ethyl acetate/hexane) as a colorless solid (59 mg, 50 %). ^1H NMR (400 MHz, $CDCl_3$) δ 7.89 (s, 1H), 7.84 (dd, J = 7.7, 1.1 Hz, 1H), 7.76 (d, J = 7.7 Hz, 1H), 0.87 (s, 9H), 0.33 (s, 6H); ^{13}C NMR (100 MHz, $CDCl_3$) δ 146.75, 138.85, 138.54, 131.83, 115.80, 115.52, 114.69, 26.15, 16.79, -6.55; HRMS (ESI) Calcd. for $C_{14}H_{19}N_2Si$ [$(M+H)^+$] 243.1318, found 243.1321.

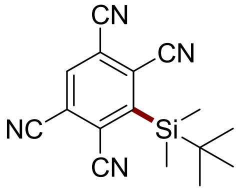

3-(*tert*-butyldimethylsilyl)benzene-1,2,4,5-tetracarbonitrile (5e): According to the general procedure A, 1,2,4,5-tetracarbonitrile (0.5 mmol, 1 equiv.), *tert*-butyldimethylsilane (72 mg, 2.5 mmol, 5 equiv.), $Na_2S_2O_8$ (238 mg, 1.0 mmol, 2.0 equiv.), $Ir(ppy)_2(dtbpy)PF_6$ (4.6 mg, 0.005 mmol, 0.01 equiv.) and 5 mL DMSO/DCE (1:1) (0.1 M) were used. The product was isolated by flash chromatography (2% ethyl acetate/hexane) as a colorless solid (105 mg,72 %). ^1H NMR (400 MHz, $CDCl_3$) δ 8.20 (s, 1H), 1.06 (s, 9H), 0.75 (s, 6H); ^{13}C NMR (100 MHz, $CDCl_3$) δ 152.12, 136.64, 126.30, 121.89, 115.18, 113.29, 26.61, 19.81, -2.11; HRMS (ESI) Calcd. for $C_{16}H_{17}N_4Si$ [$(M+H)^+$] 293.1222, found 293.1231.

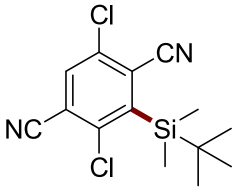

3-(*tert*-butyldimethylsilyl)-2,5-dichloroterephthalonitrile (5f): According to the general procedure A, 2,5-dichloroterephthalonitrile (0.5 mmol, 1 equiv.), *tert*-butyldimethylsilane (290 mg, 2.5 mmol, 5 equiv.), $Na_2S_2O_8$ (238 mg, 1.0 mmol, 2.0 equiv.), $Ir(ppy)_2(dtbpy)PF_6$ (4.6 mg, 0.005 mmol, 0.01 equiv.) and 5 mL DMSO/DCE (1:1) (0.1 M) were used. The product was isolated by flash chromatography (2% ethyl acetate/hexane) as a colorless solid (115 mg, 74%). ^1H NMR (400 MHz, $CDCl_3$) δ 7.82 (s, 1H), 1.04 (s, 9H), 0.66 (s, 6H); ^{13}C NMR (100 MHz, $CDCl_3$) δ 146.43, 141.98, 137.79, 134.62, 124.42, 119.12, 115.60, 114.27, 27.05, 19.84, -0.13; HRMS (ESI) Calcd. for $C_{14}H_{17}Cl_2N_2Si$ [$(M+H)^+$] 311.0538, found 311.0540.

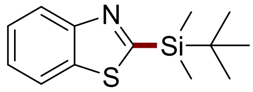

2-(*tert*-butyldimethylsilyl)benzo[*d*]thiazole (7a): According to the general procedure C, benzo[*d*]thiazole (0.5 mmol, 1 equiv.), *tert*-butyldimethylsilane (290 mg, 2.5 mmol, 5 equiv.), TMSOOTMS (138 mg, 1.0 mmol, 2.0 equiv.), $Ir(ppy)_2(dtbpy)PF_6$ (4.6 mg, 0.005 mmol, 0.01 equiv.) and 5 mL DMSO/DCE (1:1) (0.1 M) were used. The product was isolated by flash chromatography (hexane) as a colorless solid (50 mg, 41 %). ^{1}H NMR (400 MHz, $CDCl_3$) δ 8.20 (d, J = 8.2 Hz, 1H), 7.97 (d, J = 7.9 Hz, 1H), 7.49 (t, J = 7.6 Hz, 1H), 7.40 (t, J = 7.5 Hz, 1H), 1.02 (s, 9H), 0.47 (s, 6H); ^{13}C NMR (101 MHz, $CDCl_3$) δ 174.79, 156.17, 136.14, 125.62, 125.00, 123.44, 121.48, 26.34, 16.98, -5.45; HRMS (ESI) Calcd. for $C_{13}H_{20}NSSi$ [$(M+H)^+$] 250.1080, found 250.1085.

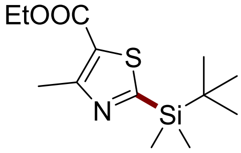

ethyl 2-(*tert*-butyldimethylsilyl)-4-methylthiazole-5-carboxylate (7b): According to the general procedure C, ethyl 4-methylthiazole-5-carboxylate (0.5 mmol, 1 equiv.), *tert*-butyldimethylsilane (290 mg, 2.5 mmol, 5 equiv.), TMSOOTMS (138 mg, 1.0 mmol, 2.0 equiv.), $Ir(ppy)_2(dtbpy)PF_6$ (4.6 mg, 0.005 mmol, 0.01 equiv.) and 5 mL DMSO/DCE (1:1) (0.1 M) were used. The product was isolated by flash chromatography (3 % ethyl acetate/hexane) as a colorless solid (95 mg, 67 %). ^{1}H NMR (400 MHz, $CDCl_3$) δ 4.32 (q, J = 7.1 Hz, 2H), 2.79 (s, 3H), 1.36 (t, J = 7.1 Hz, 3H), 0.96 (s, 9H), 0.36 (s, 6H); ^{13}C NMR (100 MHz, $CDCl_3$) δ 176.30, 162.45, 162.33, 124.42, 61.12, 26.20, 17.38, 16.82, 14.31, -5.59; HRMS (ESI) Calcd. for $C_{13}H_{24}NO_2SSi$ [$(M+H)^+$] 286.1292, found 286.1296.

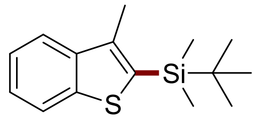

***tert*-butyldimethyl(3-methylbenzo[*b*]thiophen-2-yl)silane (7c):** According to the general procedure C, 3-methylbenzo[*b*]thiophene (0.5 mmol, 1 equiv.), *tert*-butyldimethylsilane (290 mg, 2.5 mmol, 5 equiv.), TMSOOTMS (138 mg, 1.0 mmol, 2.0 equiv.), $Ir(ppy)_2(dtbpy)PF_6$ (4.6 mg, 0.005 mmol, 0.01 equiv.) and 5 mL DMSO/DCE (1:1) (0.1 M) were used. The product was isolated by flash chromatography (hexane) as a colorless solid (85 mg, 65 %). ^{1}H NMR (400 MHz, $CDCl_3$) δ 7.88 (d, J = 7.7 Hz, 1H), 7.76 (d, J = 7.3 Hz, 1H), 7.43 - 7.34 (m, 2H), 2.55 (s, 3H), 1.00 (s, 9H), 0.46 (s, 6H); ^{13}C NMR (100 MHz, $CDCl_3$) δ 142.95, 141.77, 139.40, 132.65, 124.08, 123.55, 121.99, 121.71, 26.70, 18.23, 15.26, -3.83; HRMS (EI) Calcd. for $C_{15}H_{22}SSi$ [M^+] 262.1211, found 262.1214.

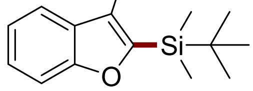

***tert*-butyldimethyl(3-methylbenzofuran-2-yl)silane (7d):** According to the general procedure C, 3-methylbenzo[*b*]thiophene (0.5 mmol, 1 equiv.), *tert*-butyldimethylsilane (290 mg, 2.5 mmol, 5 equiv.), TMSOOTMS (138 mg, 1.0 mmol, 2.0 equiv.), $Ir(ppy)_2(dtbpy)PF_6$ (4.6 mg, 0.005 mmol, 0.01 equiv.) and 5 mL DMSO/DCE (1:1) (0.1 M) were used. The product was isolated by flash chromatography

(hexane) as a colorless solid (78 mg, 63 %). ^{1}H NMR (400 MHz, $CDCl_3$) δ 7.54 – 7.49 (m, 1H), 7.46 (d, *J* = 8.1 Hz, 1H), 7.30 - 7.20 (m, 2H), 2.33 (s, 3H), 0.97 (s, 9H), 0.38 (s, 6H); ^{13}C NMR (100 MHz, $CDCl_3$) δ 157.49, 156.29, 129.84, 126.15, 124.14, 121.64, 119.15, 111.09, 26.40, 17.78, 9.22, -5.66; HRMS (EI) Calcd. for $C_{15}H_{22}OSi$ [M^+] 246.1440, found 246.1442.

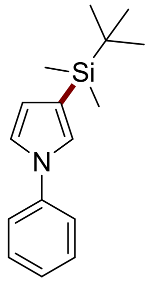

3-(*tert*-butyldimethylsilyl)-1-phenyl-1*H*-pyrrole (7e): According to the general procedure C, 3-methylbenzo[*b*]thiophene (0.5 mmol, 1 equiv.), *tert*-butyldimethylsilane (290 mg, 2.5 mmol, 5 equiv.), TMSOOTMS (138 mg, 1.0 mmol, 2.0 equiv.), Ir(ppy)$_2$(dtbpy)PF_6 (4.6 mg, 0.005 mmol, 0.01 equiv.) and 5 mL DMSO/DCE (1:1) (0.1 M) were used. The product was isolated by flash chromatography (hexane) as a colorless solid (66 mg, 51 %). ^{1}H NMR (400 MHz, $CDCl_3$) δ 7.44 – 7.39 (m, 4H), 7.26 (s, 1H), 7.17 (dd, *J* = 2.6, 2.1 Hz, 1H), 7.09 (t, *J* = 1.8 Hz, 1H), 6.40 (dd, *J* = 2.7, 1.6 Hz, 1H), 0.93 (s, 9H), 0.22 (s, 6H); ^{13}C NMR (100 MHz, $CDCl_3$) δ 140.46, 129.46, 125.55, 125.45, 120.53, 120.30, 117.22, 116.20, 26.48, 16.75, -5.32; HRMS (EI) Calcd. for $C_{16}H_{23}NSi$ [M^+] 257.1600, found 257.1602.

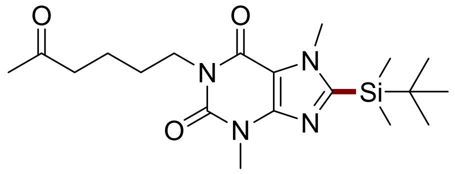

8-(*tert*-butyldimethylsilyl)-3,7-dimethyl-1-(5-oxohexyl)-1*H*-purine-2,6(3*H*,7*H*)-dione (7f): According to the general procedure C, 3,7-dimethyl-1-(5-oxohexyl)-3,4,5,7-tetrahydro-1*H*-purine-2,6-dione (0.5 mmol, 1 equiv.), *tert*-butyldimethylsilane (290 mg, 2.5 mmol, 5 equiv.), TMSOOTMS (138 mg, 1.0 mmol, 2.0 equiv.), Ir(ppy)$_2$(dtbpy)PF_6 (4.6 mg, 0.005 mmol, 0.01 equiv.) and 5 mL DMSO/DCE (1:1) (0.1 M) were used. The product was isolated by flash chromatography (20 % acetone/hexane) as a colorless solid (88 mg, 45 %). ^{1}H NMR (400 MHz, $CDCl_3$) δ 4.03 (s, 3H), 3.99 (t, *J* = 7.0 Hz, 2H), 3.56 (s, 3H), 2.49 (t, *J* = 7.0 Hz, 2H), 2.13 (s, 3H), 1.63 (dd, *J* = 6.5, 3.2 Hz, 4H), 0.97 (s, 9H), 0.40 (s, 6H); ^{13}C NMR (100 MHz, $CDCl_3$) δ 208.90, 157.09, 155.33, 151.51, 148.93, 109.45, 43.19, 40.62, 34.18, 29.93, 29.55, 27.42, 26.36, 20.94, 17.69, -4.98; HRMS (ESI) Calcd. for $C_{19}H_{33}N_4O_3Si$ [$(M+H)^+$] 393.2316, found 393.2318.

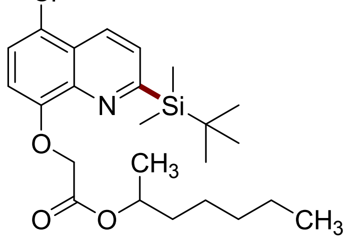

heptan-2-yl 2-((2-(*tert*-butyldimethylsilyl)-5-chloroquinolin-8-yl)oxy)acetate (8a): According to the general procedure A, heptan-2-yl 2-((5-chloroquinolin-8-yl)oxy)acetate (0.5 mmol, 1 equiv.), *tert*-butyldimethylsilane (290 mg, 2.5 mmol, 5 equiv.), $Na_2S_2O_8$ (238 mg, 1.0 mmol, 2.0 equiv.), $Ir(ppy)_2(dtbpy)PF_6$ (4.6 mg, 0.005 mmol, 0.01 equiv.) and 5 mL DMSO/DCE (1:1) (0.1 M) were used. The product was isolated by flash chromatography (5% ethyl acetate/hexane) as a colorless oil (115 mg, 51%).^{1}H NMR (400 MHz, $CDCl_3$) δ 8.39 (d, *J* = 8.5 Hz, 1H), 7.71 (d, *J* = 8.5 Hz, 1H), 7.47 (d, *J* = 8.3 Hz, 1H), 7.18 (d, *J* = 8.3 Hz, 1H), 5.11 (s, 2H), 5.03 (dd, *J* = 7.1, 5.8 Hz, 1H), 1.24 (d, *J* = 6.3 Hz, 8H), 0.96 (s, 10H), 0.88 – 0.82 (m, 4H), 0.42 (s, 6H); ^{13}C NMR (100 MHz, $CDCl_3$) δ 169.09, 168.70, 153.26, 141.81, 129.74, 127.05, 126.22, 126.09, 124.69, 115.33, 35.77, 31.54, 26.62, 24.94, 22.47, 19.90, 17.05, 13.94, -6.23. HRMS (ESI) Calcd. for $C_{24}H_{37}ClNO_3Si$ [$(M+H)^+$] 450.2226, found 450.2228.

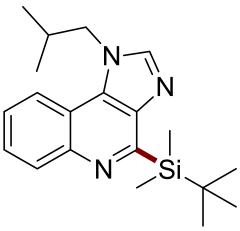

4-(*tert*-butyldimethylsilyl)-1-isobutyl-1H-imidazo[4,5-c]quinolone (8b): According to the general procedure A, 1-isobutyl-1H-imidazo[4,5-c]quinoline (0.5 mmol, 1 equiv.), *tert*-butyldimethylsilane (290 mg, 2.5 mmol, 5 equiv.), $Na_2S_2O_8$ (238 mg, 1.0 mmol, 2.0 equiv.), $Ir(ppy)_2(dtbpy)PF_6$ (4.6 mg, 0.005 mmol, 0.01 equiv.) and 5 mL DMSO/DCE (1:1) (0.1 M) were used. The product was isolated by flash chromatography (2% ethyl acetate/hexane) as a colorless oil (136 mg, 80%). ^{1}H NMR (400 MHz, $CDCl_3$) δ 8.36 (d, *J* = 8.1 Hz, 1H), 8.07 (dd, *J* = 8.2, 1.0 Hz, 1H), 7.88 (s, 1H), 7.65 (ddd, *J* = 8.4, 7.0, 1.4 Hz, 1H), 7.58 (ddd, *J* = 8.3, 7.0, 1.4 Hz, 1H), 4.30 (d, *J* = 7.4 Hz, 2H), 2.47 – 2.25 (m, 1H), 1.05 (s, 9H), 1.03 (s, 3H), 1.01 (s, 3H), 0.62 (s, 6H). ^{13}C NMR (100 MHz, $CDCl_3$) δ 164.69, 144.89, 143.60, 142.99, 131.70, 130.00, 126.35, 126.10, 119.80, 117.36, 54.98, 28.71, 27.04, 19.82, 17.67, -4.94. HRMS (ESI) Calcd. for $C_{20}H_{30}N_3Si$ [$(M+H)^+$] 340.2204, found 340.2208.

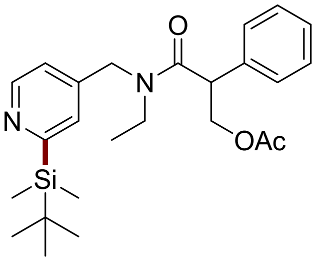

3-(((2-(*tert*-butyldimethylsilyl)pyridin-4-yl)methyl)(ethyl)amino)-3-oxo-2-phenylpropyl acetate (8c): According to the general procedure A, 3-(ethyl(pyridin-4-ylmethyl)amino)-3-oxo-2-phenylpropyl acetate (0.5 mmol, 1 equiv.), *tert*-butyldimethylsilane (580 mg, 5 mmol, 10 equiv.), $Na_2S_2O_8$ (238 mg, 1.0 mmol, 2.0 equiv.), $Ir(ppy)_2(dtbpy)PF_6$ (4.6 mg, 0.005 mmol, 0.01 equiv.) and 5 mL DMSO/DCE (1:1) (0.1 M) were used. The product was isolated by flash chromatography (5% ethyl acetate/hexane) as a colorless oil (70 mg, 32%). ^{1}H NMR (400 MHz, $CDCl_3$) δ 8.64(8.69) (d, *J* = 5.1 Hz, 1H), 7.37 – 7.19 (m, 6H), 6.92(6.89) (d, *J* = 5.0 Hz, 1H), 4.87(4.49) (d, *J* = 15.8 Hz, 1H), 4.69 – 4.62 (m, 1H), 4.38 – 4.13(3.92 – 3.88) (m, 3H), 3.43 - 3.36(3.68 – 3.63)(m, 1H), 3.13 – 3.10(3.24- 3.19) (m, 1H), 2.04(2.00) (s, 3H), 1.09 – 1.03 (m, 3H), 0.92 – 0.82 (m, 9H), 0.33 – 0.20 (m, 6H); ^{13}C NMR (100 MHz, $CDCl_3$) δ 170.87, 170.67, 166.61, 150.06, 149.75, 144.15, 143.44, 135.53, 135.26, 129.17, 128.57, 128.10, 128.01, 127.97, 127.47, 121.20, 119.97, 66.65, 66.45, 49.48, 48.27,

47.83, 47.59, 41.97, 41.57, 29.67, 26.47, 22.66, 20.93, 20.88, 16.88, 16.85, 14.10, 13.86, 12.40, -6.35, -6.40. HRMS (ESI) Calcd. for $C_{25}H_{37}N_2O_3Si$ $[(M+H)^+]$ 441.2568, found 441.2566.

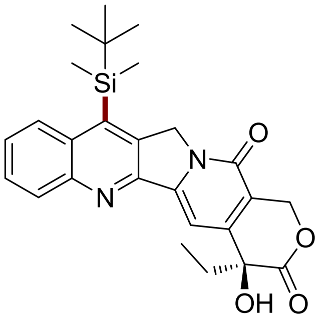

(*S*)-11-(*tert*-butyldimethylsilyl)-4-ethyl-4-hydroxy-1H-pyrano[3',4':6,7]indolizino[1,2-b]quinoline-3,14(4H,12H)-dione (8d): According to the general procedure A, Camptothecin (0.5 mmol, 1 equiv.), *tert*-butyldimethylsilane (580 mg, 5 mmol, 10 equiv.), $Na_2S_2O_8$ (238 mg, 1.0 mmol, 2.0 equiv.), $Ir(ppy)_2(dtbpy)PF_6$ (4.6 mg, 0.005 mmol, 0.01 equiv.) and 5 mL DMSO/DCE (4:1) (0.1 M) were used. Then irradiated at 467 nm Blue Led (Kessil Photoredox LED Lights PR160 Series) (25% intensity) for 24 hours. The product was isolated by flash chromatography (10% ethyl acetate/hexane) as a yellow solid (51 mg, 22%). ^1H NMR (400 MHz, $CDCl_3$) δ 8.24 (d, J = 3.5 Hz, 1H), 8.22 (d, J = 3.1 Hz, 1H), 7.81 – 7.76 (m, 1H), 7.68 (s, 1H), 7.65 – 7.60 (m, 1H), 5.76 (d, J = 16.3 Hz, 1H), 5.31 (t, J = 8.1 Hz, 3H), 3.72 (s, 1H), 1.90 (dt, J = 14.3, 6.8 Hz, 2H), 1.07 – 1.04 (m, 3H), 0.99 (s, 9H), 0.70 (s, 6H).
Spectroscopic data in accordance with literature.[5]

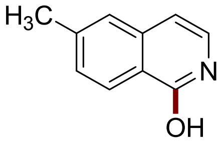

6-methylisoquinolin-1-ol (9): 1-(*tert*-butyldimethylsilyl)-6-methylisoquinoline **3a** (52 mg, 0.2 mmol) and 30% H_2O_2 (0.5 mL) were placed into a flask in tetrahydrofuran (1 mL). The reaction mixture was stirred at room temperature for 4 h. The reaction was quenched with saturated $NaHSO_3$ aqueous solution (10 mL) and extracted with ethyl acetate (3×10 mL). Organic layers were combined, dried over anhydrous Na_2SO_4 and concentrated to obtain the compound as brown oil, which was purified by column chromatography (20% ethyl acetate/hexane) to yield the title compound (31 mg, 99%) as a white solid. ^1H NMR (400 MHz, $CDCl_3$) δ 11.64 (s, 1H), 8.30 (d, J = 8.2 Hz, 1H), 7.39 – 7.29 (m, 2H), 7.17 (d, J = 7.1 Hz, 1H), 6.51 (d, J = 7.1 Hz, 1H), 2.49 (s, 3H); ^{13}C NMR (100 MHz, $CDCl_3$) δ 164.46, 143.25, 138.31, 128.43, 127.71, 127.15, 125.93, 123.73, 106.60, 21.83. HRMS (ESI) Calcd. for $C_{10}H_{10}NO$ $[(M+H)^+]$ 160.0757, found 160.0762.

General procedure D for Hiyama-Denmark cross-coupling of heteroarylsilane products:
To a 10 mL vial equipped with a Teflon septum and magnetic stir bar were added the corresponding aryl silane (0.5 mmol, 1.0 equiv.), the corresponding aryl iodides or bromide (1.0 mmol, 2 equiv.), $Pd(Ph_3P)_4$ (0.025 mmol, 0.05 equiv.) and Ag_2O (0.5 mmol, 1 equiv.). The vial was sealed and placed under an atmosphere of nitrogen, then anhydrous DMF (5 mL, 0.1 M) and TBAF (0.25 mL, 0.5 equiv., 1 mmol/L in THF) were added. The reaction was heated at 90 °C for 4 hours. The reaction mixture was diluted with H_2O, extracted with ethyl acetate (3 × 20 mL), the combined organic extracts were washed with brine (30 mL), dried over anhydrous Na_2SO_4 and concentrated in vacuo. Purification of

the crude product by flash chromatography on silica gel using the indicated solvent system afforded the desired product.

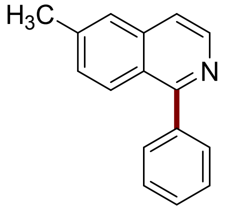

6-methyl-1-phenylisoquinoline (10a): According to the general procedure D for Hiyama-Denmark cross-coupling with 1-(*tert*-butyldimethylsilyl)-6-methylisoquinoline **3a** and 4-iodobenzene. The crude mixture was purified by flash column chromatography (20% ethyl acetate/hexane) to give the title compound as a white solid (70 mg, 64%). ^{1}H NMR (400 MHz, $CDCl_3$) δ 8.56 (d, *J* = 5.7 Hz, 1H), 7.99 (d, *J* = 8.7 Hz, 1H), 7.73 – 7.67 (m, 2H), 7.65 (s, 1H), 7.59 – 7.45 (m, 4H), 7.36 (dd, *J* = 8.7, 1.6 Hz, 1H), 2.55 (s, 3H); ^{13}C NMR (100 MHz, $CDCl_3$) δ 160.32, 142.21, 140.31, 139.61, 137.14, 129.84, 129.39, 128.47, 128.27, 127.36, 125.85, 125.05, 119.43, 21.83. HRMS (ESI) Calcd. for $C_{16}H_{14}N$ $[(M+H)^+]$ 220.1121, found 220.1119.

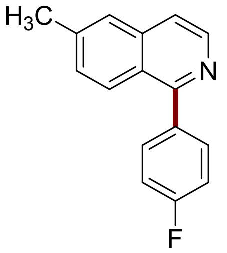

1-(4-fluorophenyl)-6-methylisoquinoline (10b): According to the general procedure D for Hiyama-Denmark cross-coupling with 1-(*tert*-butyldimethylsilyl)-6-methylisoquinoline **3a** and 1-fluoro-4-iodobenzene. The crude mixture was purified by flash column chromatography (20% ethyl acetate/hexane) to give the title compound as a white solid (83 mg, 70%). ^{1}H NMR (400 MHz, $CDCl_3$) δ 8.59 (d, *J* = 5.7 Hz, 1H), 7.99 (d, *J* = 8.7 Hz, 1H), 7.76 – 7.67 (m, 3H), 7.60 (d, *J* = 5.7 Hz, 1H), 7.42 (dd, *J* = 8.7, 1.6 Hz, 1H), 7.30 – 7.24 (m, 2H), 2.60 (s, 3H); ^{13}C NMR (100 MHz, $CDCl_3$) δ 164.24, 161.78, 159.18, 142.13), 140.47, 137.18, 135.65, 135.61, 131.68, 131.60, 129.57, 127.05, 125.95, 125.00, 119.58, 115.39, 115.18, 21.83. HRMS (ESI) Calcd. for $C_{16}H_{13}FN$ $[(M+H)^+]$ 238.1027, found 238.1029.

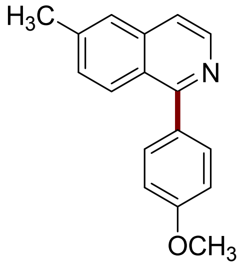

1-(4-methoxyphenyl)-6-methylisoquinoline (10c): According to the general procedure D for Hiyama-Denmark cross-coupling with 1-(*tert*-butyldimethylsilyl)-6-methylisoquinoline **3a** and 1-iodo-4-methoxybenzene. The crude mixture was purified by flash column chromatography (20% ethyl acetate/hexane) to give the title compound as a white solid (95 mg, 76%). ^{1}H NMR (400 MHz, $CDCl_3$) δ 8.53 (d, *J* = 5.7 Hz, 1H), 8.02 (d, *J* = 8.7 Hz, 1H), 7.71 – 7.59 (m, 3H), 7.52 (d, *J* = 5.7 Hz, 1H), 7.35 (dd, *J* = 8.7, 1.5 Hz, 1H), 7.06 (d, *J* = 8.7 Hz, 2H), 3.89 (s, 3H), 2.54 (s, 3H); ^{13}C NMR (100 MHz,

$CDCl_3$) δ 159.91, 142.17, 140.19, 137.21, 132.13, 131.20, 129.27, 127.43, 125.84, 125.06, 119.06, 113.70, 55.34, 21.80. HRMS (ESI) Calcd. for $C_{17}H_{16}NO$ [$(M+H)^+$] 250.1226, found 250.1233.

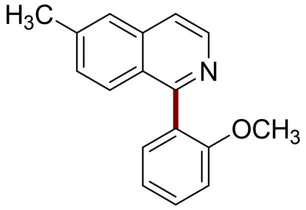

1-(2-methoxyphenyl)-6-methylisoquinoline (10d): According to the general procedure D for Hiyama-Denmark cross-coupling with 1-(*tert*-butyldimethylsilyl)-6-methylisoquinoline **3a** and 1-iodo-2-methoxybenzene. The crude mixture was purified by flash column chromatography (20% ethyl acetate/hexane) to give the title compound as a white solid (90 mg, 72%). ^1H NMR (400 MHz, $CDCl_3$) δ 8.57 (d, J = 5.7 Hz, 1H), 7.65 – 7.55 (m, 3H), 7.47 (ddd, J = 8.3, 7.6, 1.8 Hz, 1H), 7.38 (dd, J = 7.4, 1.7 Hz, 1H), 7.30 (dd, J = 8.6, 1.6 Hz, 1H), 7.11 (t, J = 7.4Hz, 1H), 7.05 (d, J = 8.3 Hz, 1H), 3.69 (s, 3H), 2.53 (s, 3H); ^{13}C NMR (100 MHz, $CDCl_3$) δ 158.57, 157.09, 142.20, 140.22, 136.48, 131.17, 129.92, 129.11, 128.64, 127.71, 126.12, 125.58, 120.68, 119.65, 111.05, 55.51, 21.88. HRMS (ESI) Calcd. for $C_{17}H_{16}NO$ [$(M+H)^+$] 250.1226, found 250.1231.

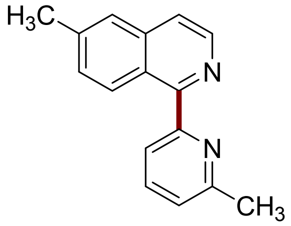

6-methyl-1-(6-methylpyridin-2-yl)isoquinoline (10e): According to the general procedure D for Hiyama-Denmark cross-coupling with 1-(*tert*-butyldimethylsilyl)-6-methylisoquinoline **3a** and 2-bromo-6-methylpyridine. The crude mixture was purified by flash column chromatography (50% ethyl acetate/hexane) to give the title compound as a white solid (83 mg, 71%). ^1H NMR (400 MHz, $CDCl_3$) δ 8.57 (d, J = 5.7 Hz, 1H), 8.37 (d, J = 8.7 Hz, 1H), 7.77 (t, J = 7.7 Hz, 1H), 7.69 (d, J = 7.6 Hz, 1H), 7.65 – 7.56 (m, 2H), 7.40 (dd, J = 8.7, 1.6 Hz, 1H), 7.26 (t, J = 3.8 Hz, 2H), 2.69 (s, 3H), 2.54 (s, 3H); ^{13}C NMR (100 MHz, $CDCl_3$) δ 157.63, 141.97, 140.30, 137.44, 136.96, 129.76, 127.48, 125.69, 125.07, 122.68, 122.06, 120.53, 24.62, 21.86. HRMS (ESI) Calcd. for $C_{16}H_{15}N_2$ [$(M+H)^+$] 235.1230, found 235.1227.

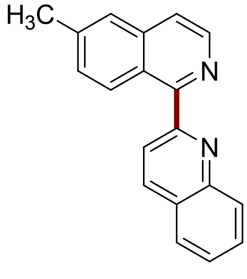

2-(6-methylisoquinolin-1-yl)quinolone (10f): According to the general procedure D for Hiyama-Denmark cross-coupling with 1-(*tert*-butyldimethylsilyl)-6-methylisoquinoline **3a** and 2-bromoquinoline. The crude mixture was purified by flash column chromatography (50% ethyl acetate/hexane) to give the title compound as a white solid (90 mg, 67%). ^1H NMR (400 MHz, $CDCl_3$) δ 8.69 (d, J = 8.7 Hz, 1H), 8.64 (d, J = 5.6 Hz, 1H), 8.36 (d, J = 8.5 Hz, 1H), 8.25 (d, J = 8.5 Hz, 1H), 8.13 (d, J = 8.5 Hz, 1H), 7.92 (d, J = 8.1 Hz, 1H), 7.79 (ddd, J = 8.4, 7.0, 1.4 Hz, 1H), 7.70 – 7.58 (m, 3H), 7.45 (dd, J = 8.8, 1.5 Hz, 1H), 2.56 (s, 3H); ^{13}C NMR (100 MHz, $CDCl_3$) δ 158.13, 157.15,

147.30, 142.00, 140.47, 137.61, 136.78, 130.12, 129.83, 129.68, 127.69, 127.62, 127.55, 127.01 (s), 125.77, 125.31, 122.73, 120.94, 21.91. HRMS (ESI) Calcd. for $C_{19}H_{15}N_2$ $[(M+H)^+]$ 271.1230, found 271,1234.

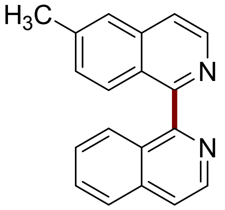

7-**methyl-1,1'-biisoquinoline (10g):** According to the general procedure D for Hiyama-Denmark cross-coupling with 1-(*tert*-butyldimethylsilyl)-6-methylisoquinoline **3a** and 1-bromoisoquinoline. The crude mixture was purified by flash column chromatography (50% ethyl acetate/hexane) to give the title compound as a white solid (95 mg, 70%). ^{1}H NMR (400 MHz, $CDCl_3$) δ 8.70 (d, J = 5.7 Hz, 1H), 8.66 (d, J = 5.7 Hz, 1H), 7.93 (d, J = 8.3 Hz, 1H), 7.80 (d, J = 5.6 Hz, 1H), 7.75 – 7.61 (m, 4H), 7.62 (d, J = 8.6 Hz, 1H), 7.46 (ddd, J = 8.2, 6.9, 1.1 Hz, 1H), 7.29 (dd, J = 8.6, 1.5 Hz, 1H), 2.54 (s, 3H); ^{13}C NMR (100 MHz, $CDCl_3$) δ 158.19, 157.67, 141.98, 141.89, 140.71, 137.12, 136.76, 130.28, 129.80 , 127.75, 127.47, 127.19, 126.90, 126.85, 126.19, 125.73, 120.97, 120.54, 21.95. HRMS (ESI) Calcd. for $C_{19}H_{15}N_2$ $[(M+H)^+]$ 271.1230, found 271.1231.

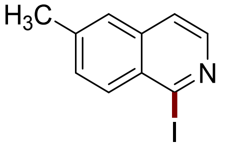

1-iodo-6-methylisoquinoline (11): 1-(*tert*-butyldimethylsilyl)-6-methylisoquinoline **3a** (52 mg, 0.2 mmol) and NIS (180 mg, 0.8 mmol) were placed into a Schlenk flask under nitrogen in anhydrous tetrahydrofuran (2 mL). Then anhydrous AgF (105 mg, 0.8 mmol) was added. After this addition, the reaction mixture was stirred at 80 ^{o}C for 4 h. The solvent was removed in vacuo and the crude was directly poured into a flash column chromatography (20% ethyl acetate/hexane) to give the title compound as a colorless oil (39 mg, 72%). ^{1}H NMR (400 MHz, $CDCl_3$) δ 8.19 (d, J = 5.6 Hz, 1H), 7.97 (d, J = 9.1 Hz, 1H), 7.55 – 7.43 (m, 3H), 2.55 (s, 3H); ^{13}C NMR (100 MHz, $CDCl_3$) δ 143.04, 141.70, 136.39, 132.61, 131.16, 130.42, 127.06, 126.10, 120.86, 21.72. HRMS (ESI) Calcd. for $C_{10}H_9IN$ $[(M+H)^+]$ 269.9774, found 269.9781.

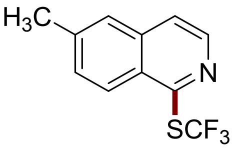

6-methyl-1-((trifluoromethyl)thio)isoquinoline (12): 1-(*tert*-butyldimethylsilyl)-6-methyl isoquinoline **3a** (52 mg, 0.2 mmol) and 1-((trifluoromethyl)thio)pyrrolidine-2,5-dione (159 mg, 0.8 mmol) were placed into a Schlenk flask under nitrogen in anhydrous tetrahydrofuran (2 mL). Then anhydrous AgF (105 mg, 0.8 mmol) was added. After this addition, the reaction mixture was stirred at 80 ^{o}C for 4 h. The solvent was removed in vacuo and the crude was directly poured into a flash column chromatography (20% ethyl acetate/hexane) to give the title compound as a colorless oil (30 mg, 62%). ^{1}H NMR (400 MHz, $CDCl_3$) δ 8.51 (d, J = 5.6 Hz, 1H), 8.23 (d, J = 8.7 Hz, 1H), 7.67 – 7.59 (m, 2H), 7.52 (dd, J = 8.7, 1.5 Hz, 1H), 2.57 (s, 3H); ^{13}C NMR (100 MHz, $CDCl_3$) δ 142.79, 141.71, 137.13, 130.81, 126.21, 125.77, 121.55, 21.89. HRMS (ESI) Calcd. for $C_{11}H_9F_3NS$ $[(M+H)^+]$ 244.0402, found 244.0406.

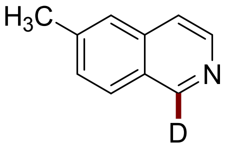

6-methylisoquinoline- 1- d (13): 1-(*tert*-butyldimethylsilyl)-6-methylisoquinoline **3a** (52 mg, 0.2 mmol) and D_2O (16 mg, 0.8 mmol) were placed into a Schlenk flask under nitrogen in anhydrous tetrahydrofuran (2 mL). Then anhydrous AgF (105 mg, 0.8 mmol) was added. After this addition, the reaction mixture was stirred at 80 °C for 4 h. The solvent was removed in vacuo and the crude was directly poured into a flash column chromatography (20% ethyl acetate/hexane) to give the title compound as a colorless oil (29 mg, 99%). ^{1}H NMR (400 MHz, $CDCl_3$) δ 9.18 (s, 0.02H), 8.47 (d, *J* = 5.7 Hz, 1H), 7.86 (d, *J* = 8.4 Hz, 1H), 7.63 – 7.50 (m, 2H), 7.43 (dd, *J* = 8.4, 1.3 Hz, 1H), 2.54 (s, 3H); ^{13}C NMR (100 MHz, $CDCl_3$) δ 151.52 (t, *J* = 107.6 Hz), 142.75, 140.79, 136.09, 129.53, 127.36, 126.98, 125.32, 120.02, 22.04. HRMS (ESI) Calcd. for $C_{10}H_8DN$ [$(M+H)^+$] 145.0871, found 145.0880.

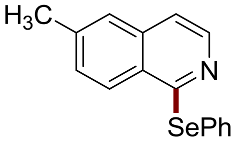

6-methyl-1-(phenylselanyl)isoquinoline (14): 1-(*tert*-butyldimethylsilyl)-6-methylisoquinoline **3a** (52 mg, 0.2 mmol) and phenyl hypochloroselenoite (153 mg, 0.8 mmol) were placed into a Schlenk flask under nitrogen in anhydrous tetrahydrofuran (2 mL). Then anhydrous AgF (105 mg, 0.8 mmol) was added. After this addition, the reaction mixture was stirred at 80 °C for 4 h. The solvent was removed in vacuo and the crude was directly poured into a flash column chromatography (20% ethyl acetate/hexane) to give the title compound as a colorless oil (28 mg, 47%). ^{1}H NMR (400 MHz, $CDCl_3$) δ 8.26 (d, *J* = 5.6 Hz, 1H), 8.16 (d, *J* = 8.6 Hz, 1H), 7.69 – 7.61 (m, 2H), 7.55 (s, 1H), 7.43 (dd, *J* = 8.6, 1.6 Hz, 1H), 7.38 (d, *J* = 5.6 Hz, 1H), 7.34 (dd, *J* = 4.2, 2.3 Hz, 3H); ^{13}C NMR (100 MHz, $CDCl_3$) δ 157.64, 142.87, 140.85, 136.24, 135.09, 129.73, 129.18, 128.52, 128.01, 127.77, 126.65, 126.17, 118.95, 21.82. HRMS (ESI) Calcd. for $C_{16}H_{14}NSe$ [$(M+H)^+$] 300.0286, found 300.0289.

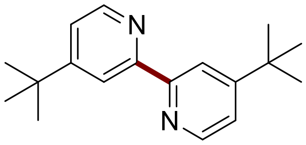

4,4'-di-*tert*-butyl-2,2'-bipyridine (15): According to the general procedure D for Hiyama-Denmark cross-coupling with 4-(*tert*-butyl)-2-(*tert*-butyldimethylsilyl)pyridine **3o** and 2-bromo-4-(*tert*-butyl)pyridine. The crude mixture was purified by flash column chromatography (50% ethyl acetate/hexane) to give the title compound as a white solid (74 mg, 55%). ^{1}H NMR (400 MHz, $CDCl_3$) δ 8.58 (dd, *J* = 5.3, 0.4 Hz, 2H), 8.40 (d, *J* = 1.4 Hz, 2H), 7.28 (dd, *J* = 5.2, 2.0 Hz, 2H), 1.36 (s, 19H); ^{13}C NMR (100 MHz, $CDCl_3$) δ 160.87, 156.44, 148.96, 120.64, 118.20, 34.90, 30.55. HRMS (ESI) Calcd. for $C_{18}H_{25}N_2$ [$(M+H)^+$] 269.2012, found 269.2015.

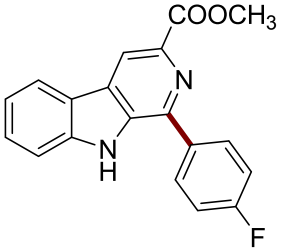

Methyl 1-(4-fluorophenyl)-9H-pyrido[3,4-b]indole-3-carboxylate (16): According to the general procedure D for Hiyama-Denmark cross-coupling methyl 1-(*tert*-butyldimethylsilyl)-9H-pyrido[3,4-b]indole-3-carboxylate **3g** and 1-fluoro-4-iodobenzene. The crude mixture was purified by flash column chromatography (20% ethyl acetate/hexane) to give the title compound as a white solid (90 mg, 56%). ^{1}H NMR (400 MHz, d^6-DMSO) δ 11.96 (s, 1H), 8.93 (s, 1H), 8.43 (d, J = 7.9 Hz, 1H), 8.07 (dd, J = 8.8, 5.5 Hz, 2H), 7.69 (d, J = 8.2 Hz, 1H), 7.66 – 7.58 (m, 1H), 7.47 (t, J = 8.9 Hz, 2H), 7.37 – 7.29 (m, 1H), 3.93 (s, 3H); ^{13}C NMR (100 MHz, d^6-DMSO) δ 165.97, 163.79, 161.34, 141.46, 141.05, 136.61, 134.47, 133.93, 130.85, 130.76, 129.23, 128.71, 122.03, 121.10, 120.43, 116.71, 115.76, 115.54, 112.71, 52.03. HRMS (ESI) Calcd. for $C_{19}H_{14}FN_2O_2$ $[(M+H)^+]$ 321.1034, found 321.1037.

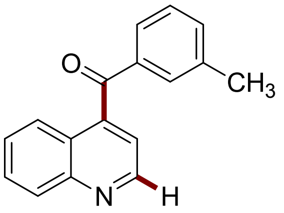

Quinolin-4-yl(m-tolyl)methanone (18): The 3-methylbenzoyl group was introduced according to the known procedure.[6] To a solution of 2-(*tert*-butyldimethylsilyl) quinoline **3e** (0.2 mmol), 3-methylbenzaldehyde (0.8 mmol) and $TMSN_3$ (0.4 mmol) in benzene (1.5 mL) phenyliodinebis(trifluroacetate) (PIFA) (0.4 mmol) was added portionwise in a 5-10 minutes period at room temperature. After stirring the reaction mixture for 2 h at room temperature, Et_3N (0.5 mL) was added and then stirred for 10 min. The solvents were removed under reduced pressure. The crude mixture was added to 1 mL H_2O, then TBAF (1.0 mL, 1 mmol/L in THF) were added, the reaction was heated at 80 °C for 4 hours. The reaction mixture was diluted with H_2O, extracted with ethyl acetate (3 × 20 mL), the combined organic extracts were washed with brine (30 mL), dried over anhydrous Na_2SO_4 and concentrated in vacuo. The product was isolated by flash chromatography (20% ethyl acetate/hexane) as a colorless oil (22 mg, 44%). ^{1}H NMR (400 MHz, $CDCl_3$) δ 9.01 (d, J = 4.3 Hz, 1H), 8.19 (d, J = 8.5 Hz, 1H), 7.82 (d, J = 9.0 Hz, 3H), 7.76 (ddd, J = 8.4, 6.9, 1.4 Hz, 1H), 7.52 (ddd, J = 8.2, 6.9, 1.2 Hz, 1H), 7.39 (d, J = 4.3 Hz, 1H), 7.26 (s, 1H), 6.94 (d, J = 9.0 Hz, 2H), 3.88 (s, 3H); ^{13}C NMR (100 MHz, $CDCl_3$) δ 194.51, 164.49, 149.52, 148.49, 145.12, 132.73, 129.98, 129.87, 129.55, 127.49, 125.47, 125.04, 119.21, 114.04, 55.60. HRMS (ESI) Calcd. for $C_{17}H_{14}NO$ $[(M+H)^+]$ 248.1070, found 248.1075.

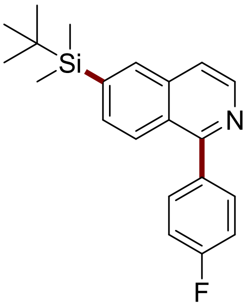

6-(*tert*-butyldimethylsilyl)-1-(4-fluorophenyl)isoquinoline (20): According to the general procedure D for Hiyama-Denmark cross-coupling with 1-(*tert*-butyldimethylsilyl)isoquinoline **3b13** (1.0 mmol) and 1-fluoro-4-iodobenzene(2.0 mmol, 2 equiv.). The crude mixture was purified by flash column

chromatography (50% ethyl acetate/hexane) to give the compound 1-(4-fluorophenyl)isoquinoline **19** as a white solid (159 mg, 71%). Then according to the general procedure A, 1-(4-fluorophenyl)isoquinoline **19** (0.5 mmol, 1 equiv.), *tert*-butyldimethylsilane (290 mg, 2.5 mmol, 5 equiv.), $Na_2S_2O_8$ (238 mg, 1.0 mmol, 2.0 equiv.), $Ir(ppy)_2(dtbpy)PF_6$ (4.6 mg, 0.005 mmol, 0.01 equiv.) and 5 mL DMSO/DCE (1:1) (0.1 M) were used. The product was isolated by flash chromatography (2% ethyl acetate/hexane) as a colorless oil (101 mg, 60 %). ^1H NMR (400 MHz, $CDCl_3$) δ 8.61 (d, *J* = 5.7 Hz, 1H), 8.08 – 7.95 (m, 2H), 7.75 – 7.60 (m, 4H), 7.23 (dd, *J* = 12.1, 5.3 Hz, 2H), 0.92 (s, 9H), 0.38 (s, 6H). ^{13}C NMR (100 MHz, $CDCl_3$) δ 164.35, 159.46, 142.08, 141.52, 135.91, 135.49, 134.02, 132.53, 131.79, 131.71, 126.65, 125.51, 120.16, 115.47, 115.26, 26.51, 17.07, -6.17; HRMS (ESI) Calcd. for $C_{21}H_{25}FNSi$ [$(M+H)^+$] 338.1740, found 338.1751.

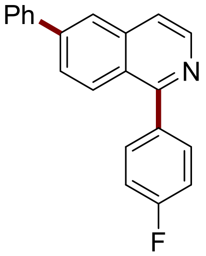

1-(4-fluorophenyl)-6-phenylisoquinoline (21): To a 10 mL vial equipped with a Teflon septum and magnetic stir bar were added 6-(*tert*-butyldimethylsilyl)-1-(4-fluorophenyl)isoquinoline **20** (0.5 mmol, 1.0 equiv.), 1-fluoro-4-iodobenzene (1.0 mmol, 2 equiv.), $PdCl_2$ (0.025 mmol, 0.05 equiv.). The vial was sealed and placed under an atmosphere of nitrogen, then anhydrous DMF (5 mL, 0.1 M) and TBAF (1.0 mL, 2.0 equiv., 1 mmol/L in THF) were added. The reaction was heated at 90 ^oC for 4 hours. The reaction mixture was diluted with H_2O, extracted with ethyl acetate (3 × 20 mL), the combined organic extracts were washed with brine (30 mL), dried over anhydrous Na_2SO_4 and concentrated in vacuo. Purification of the crude product by flash chromatography on silica gel (10% ethyl acetate/hexane) to give the title compound as a white solid (106 mg, 71%). ^1H NMR (400 MHz, $CDCl_3$) δ 8.63 (d, *J* = 5.7 Hz, 1H), 8.14 (d, *J* = 8.8 Hz, 1H), 8.08 (d, *J* = 1.5 Hz, 1H), 7.82 (dd, *J* = 8.8, 1.8 Hz, 1H), 7.78 – 7.67 (m, 5H), 7.53 (dd, *J* = 10.2, 4.7 Hz, 2H), 7.48 – 7.40 (m, 1H), 7.31 – 7.21 (m, 2H); ^{13}C NMR (100 MHz, $CDCl_3$) δ 164.32, 161.86, 159.37, 142.71, 142.43, 139.82, 137.25, 135.38, 131.73, 131.64, 129.02, 128.23, 127.85, 127.48, 127.04, 125.64, 124.63, 120.27, 115.48, 115.27. HRMS (ESI) Calcd. for $C_{21}H_{15}FN$ [$(M+H)^+$] 300.1183, found 300.1185.

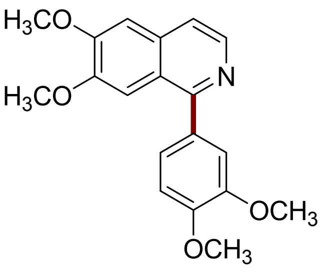

1-(3,4-dimethoxyphenyl)-6,7-dimethoxyisoquinoline (22): According to the general procedure D for Hiyama-Denmark cross-coupling with 1-(*tert*-butyldimethylsilyl)-6,7-dimethoxyisoquinoline **3b10** and 4-iodo-1,2-dimethoxybenzene. The crude mixture was purified by flash column chromatography (20% ethyl acetate/hexane) to give the title compound as a white solid (114 mg, 70%). ^1H NMR (400 MHz, $CDCl_3$) δ 8.45 (d, *J* = 5.5 Hz, 1H), 7.49 (d, *J* = 5.6 Hz, 1H), 7.44 (s, 1H), 7.28 (d, *J* = 1.7 Hz, 2H), 7.12 (s, 1H), 7.02 (d, *J* = 8.0 Hz, 1H), 4.04 (s, 3H), 3.97 (s, 3H), 3.94 (s, 3H), 3.88 (s, 3H); ^{13}C

NMR (100 MHz, $CDCl_3$) δ 157.70, 152.66, 149.92, 149.27, 148.89, 140.76, 133.86, 132.24, 122.43, 122.18, 118.55, 112.75, 110.74, 105.64, 104.93, 55.89. HRMS (ESI) Calcd. for $C_{19}H_{20}NO_4$ $[(M+H)^+]$ 326.1387, found 326.1391.

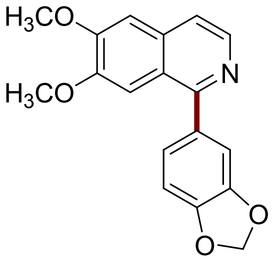

1-(benzo[d][1,3]dioxol-5-yl)-6,7-dimethoxyisoquinoline (22'): According to the general procedure D for Hiyama-Denmark cross-coupling with 1-(*tert*-butyldimethylsilyl)-6,7-dimethoxyisoquinoline **3b10** and 5-iodobenzo[d][1,3]dioxole. The crude mixture was purified by flash column chromatography (20% ethyl acetate/hexane) to give the title compound as a white solid (108 mg, 70%). ^1H NMR (400 MHz, $CDCl_3$) δ 8.42 (d, J = 5.6 Hz, 1H), 7.46 (d, J = 5.6 Hz, 1H), 7.39 (s, 1H), 7.17 (dd, J = 10.4, 2.4 Hz, 2H), 7.10 (s, 1H), 6.95 (d, J = 7.9 Hz, 1H), 6.04 (s, 2H), 4.03 (s, 3H), 3.88 (s, 3H); ^{13}C NMR (100 MHz, $CDCl_3$) δ 157.58, 152.63, 149.94, 147.81, 147.76, 141.01, 133.80, 123.46, 122.43, 118.62, 110.11, 108.19, 105.49, 104.94, 101.20, 56.02, 55.89. HRMS (ESI) Calcd. for $C_{18}H_{16}NO_4$ $[(M+H)^+]$ 310.1074, found 310.1077.

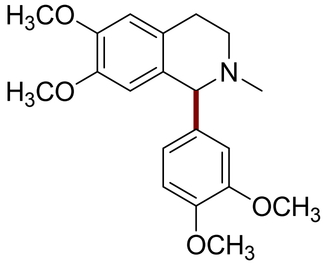

1-(3,4-dimethoxyphenyl)-6,7-dimethoxy-2-methyl-1,2,3,4-tetrahydroisoquinoline (23): To a stirred solution of 1-(3,4-dimethoxyphenyl)-6,7-dimethoxyisoquinoline (65 mg, 0.2 mmol) in THF (2 mL) at room temperature under nitrogen was added methyl iodide (284 mg, 2 mmol). The resulting solution was stirred in the dark at room temperature for 12 h during which time a yellow precipitate formed. The solvent was removed to dryness to afford the iodide salt as an yellow power. Sodium borohydride (15 mg, 0.4 mmol) was added in portions to the solution of this yellow power in methanol (2 mL) at 0 °C with constant stirring. The reaction mixture was stirred overnight at room temperature. The reaction mixture was quenched with water (20 mL) and extracted with ethyl acetate (3×10 mL). Organic layers were combined, dried over anhydrous Na_2SO_4 and concentrated to obtain the compound as brown oil, which was purified by column chromatography (10% methanol/dichloromethane) to yield the title compound (55 mg, 80%) as brown oil. ^1H NMR (400 MHz, $CDCl_3$) δ 6.86 (s, 1H), 6.81 (s, 2H), 6.60 (s, 1H), 6.13 (s, 1H), 4.44 (s, 1H), 3.93 – 3.77 (m, 9H), 3.58 (s, 3H), 3.28 (d, J = 7.4 Hz, 2H), 2.91 – 2.70 (m, 2H), 2.38 (s, 3H). ^{13}C NMR (100 MHz, $CDCl_3$) δ 149.20, 148.85, 147.92, 147.30, 135.53, 125.35, 122.53, 112.11, 111.11, 110.58, 110.30, 70.25, 55.95. 55.76, 51.39, 43.06, 29.61. HRMS (ESI) Calcd. for $C_{20}H_{26}NO_4$ $[(M+H)^+]$ 344.1856, found 344.1859.

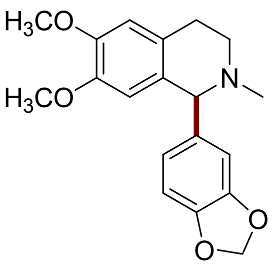

1-(benzo[d][1,3]dioxol-5-yl)-6,7-dimethoxy-2-methyl-1,2,3,4-tetrahydroisoquinoline (24): To a stirred solution of 1-(benzo[d][1,3]dioxol-5-yl)-6,7-dimethoxyisoquinoline (62 mg, 0.2 mmol) in THF (2 mL) at room temperature under nitrogen was added methyl iodide (284 mg, 2 mmol). The resulting solution was stirred in the dark at room temperature for 12 hr during which time a yellow precipitate formed. The solvent was removed to dryness to afford the iodide salt as an yellow power. Sodium borohydride (15 mg, 0.4 mmol) was added in portions to the solution of this yellow power in methanol (2 mL) at 0 °C with constant stirring. The reaction mixture was stirred overnight at room temperature. The reaction mixture was quenched with water (20 mL) and extracted with ethyl acetate (3×10 mL). Organic layers were combined, dried over anhydrous Na_2SO_4 and concentrated to obtain the compound as brown oil, which was purified by column chromatography (10% methanol/dichloromethane) to yield the title compound (49 mg, 75%) as brown oil. ^{1}H NMR (400 MHz, $CDCl_3$) δ 6.73 (d, J = 21.6 Hz, 3H), 6.59 (s, 1H), 6.16 (s, 1H), 5.94 (s, 2H), 4.30 (s, 1H), 3.85 (s, 3H), 3.62 (s, 3H), 3.14 (dd, J = 9.5, 6.5 Hz, 2H), 2.86 – 2.61 (m, 2H), 2.31 (s, 3H); ^{13}C NMR (100 MHz, $CDCl_3$) δ 147.85, 147.71, 147.22, 147.13,125.93, 123.32, 111.21, 110.62, 109.58, 107.59, 101.02, 69.98, 55.82, 55.77, 51.22, 43.48, 29.65. HRMS (ESI) Calcd. for $C_{19}H_{22}NO_4$ [$(M+H)^+$] 328.1543, found 328.1545.

8. References

1. Komiyama, T.; Minami, Y.; Hiyama, T. *Angew. Chem., Int. Ed.* **2016**, *55*, 15787.

2. Crestey, F.; A. Jensen, A. A.; Borch, M.; Andreasen,J. T.; Andersen, J.; Balle, T.; Kristensen, J. L. *J. Med. Chem.* **2013**, *56*, 9673.

3. Sakamoto, R.; Sakurai, S.; Maruoka, K. *Chem. Commun.* **2017**, *53*, 6484.

4. Ikeda, R.; Kimura, T.; Tsutsumi, T.; Tamura, S.; Sakai, N.; Konakahara, T. *Bioorg. Med. Chem. Lett.* **2012**, *22*, 3506.

5. Du, W.; Kaskar, B.; Blumbergs, P.; Subramanianb P.-K.; Curran, D. P. *Bioorg. Med. Chem.* **2003**, *11,* 451.

6. Matcha, K.; Antonchick, A. P. *Angew. Chem., Int. Ed.* **2013**, *52*, 2082.

9. Spectral Data for Products

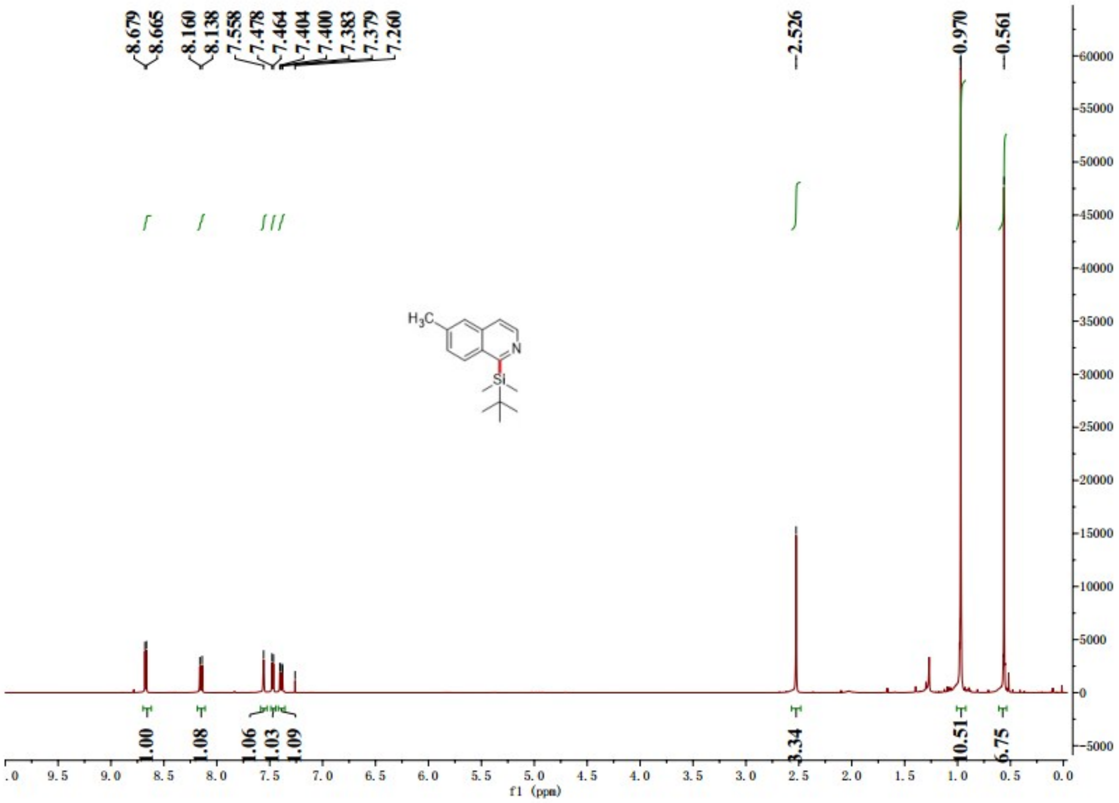

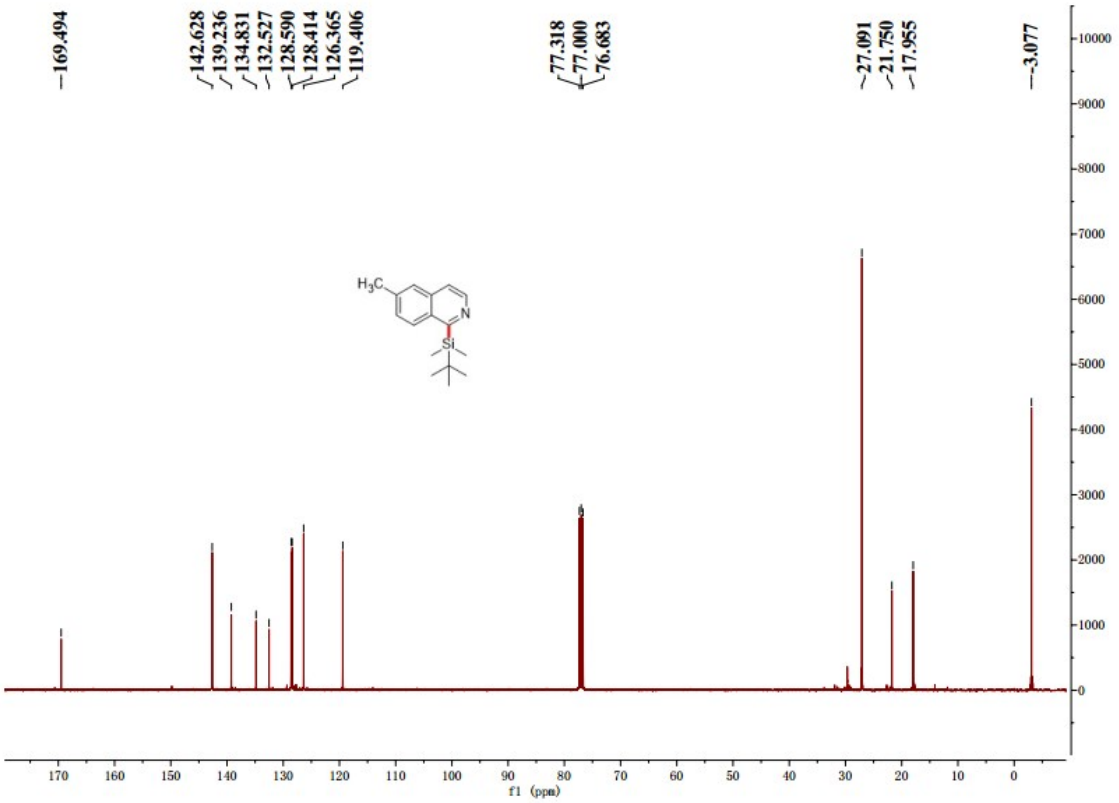

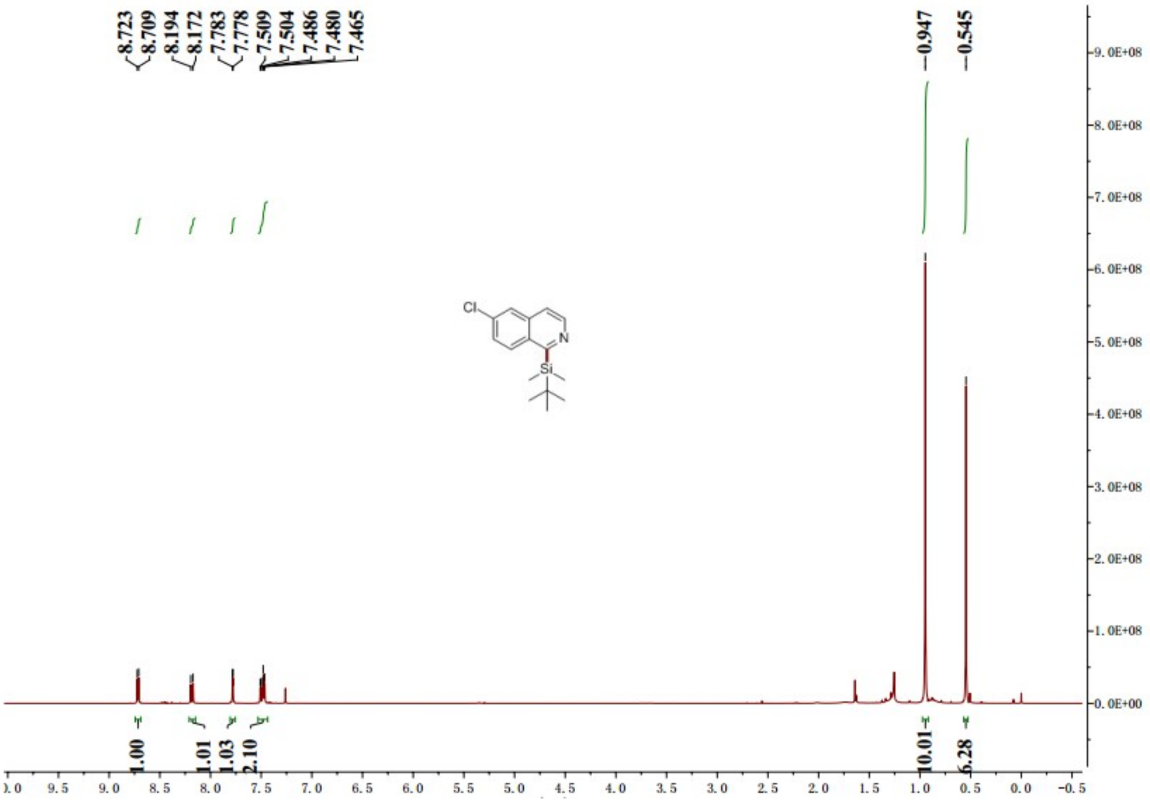
8.723
8.709
8.194
8.172
7.783
7.778
7.509
7.504
7.486
7.480
7.465
0.947
0.545
1.00
1.01
1.03
2.10
10.01
6.28
Cl
N
Si

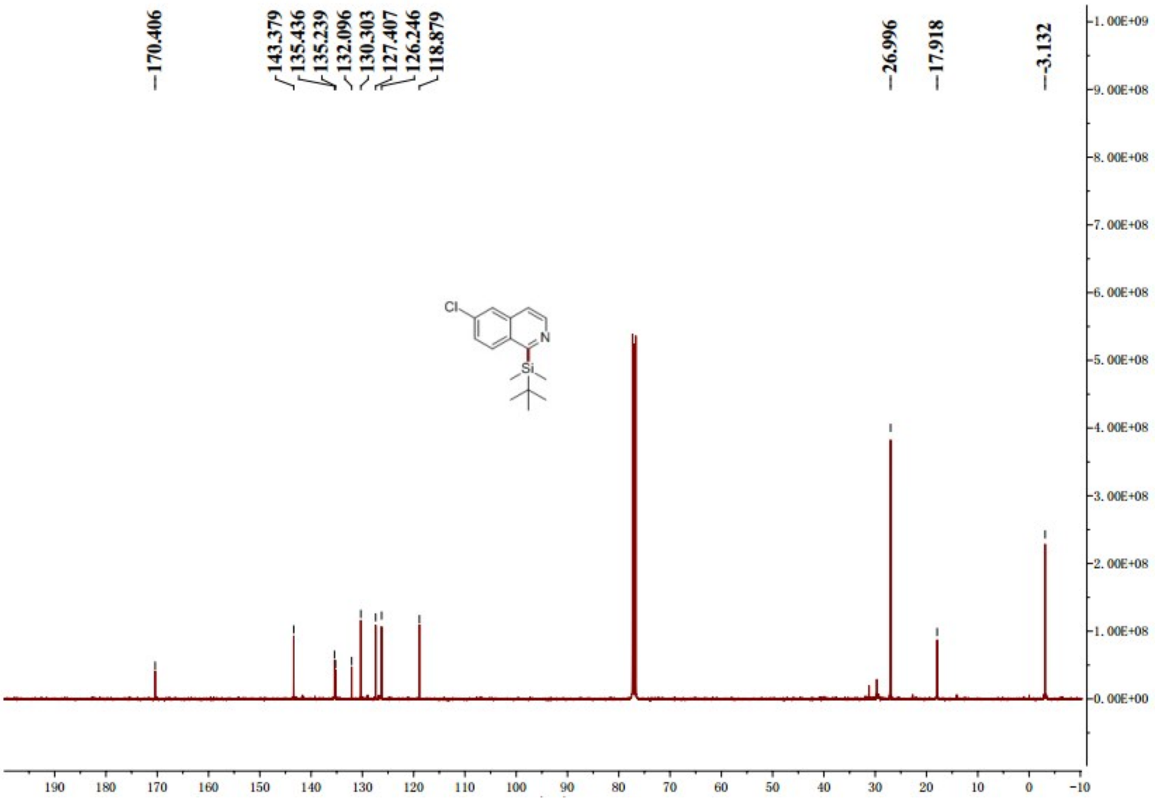
170.406
143.379
135.436
135.239
132.096
130.303
127.407
126.246
118.879
26.996
17.918
-3.132
Cl
N
Si

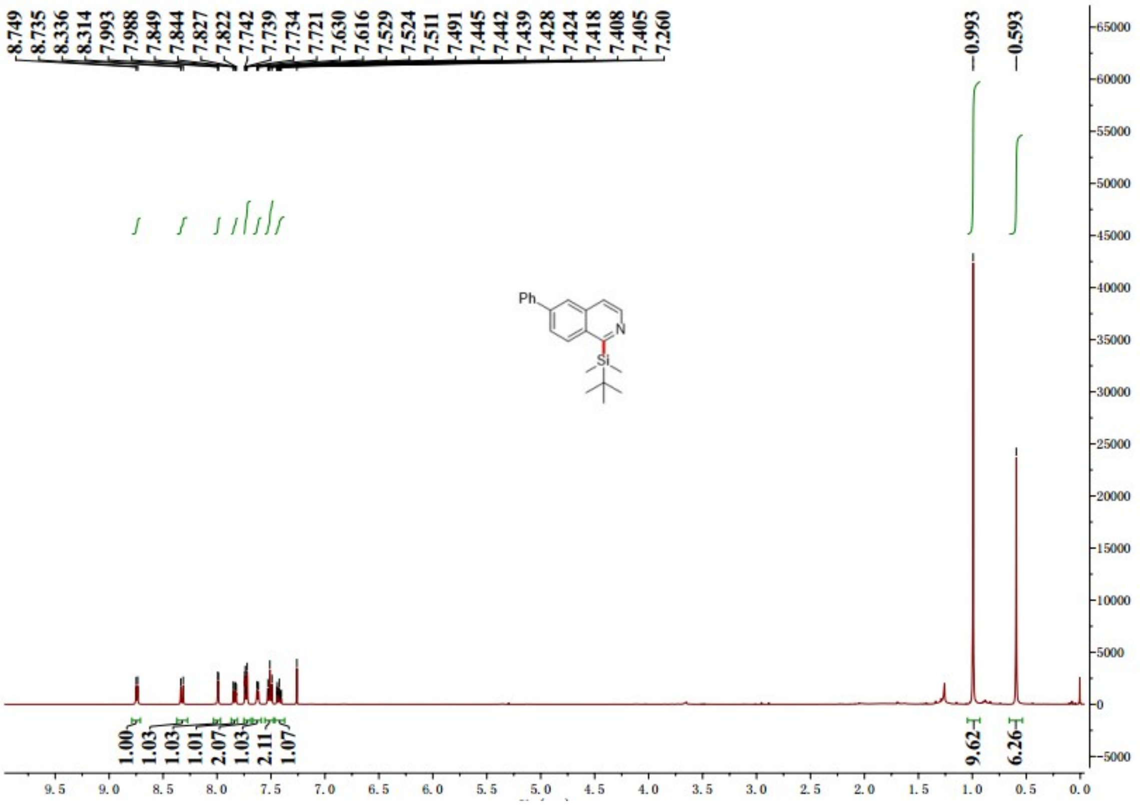

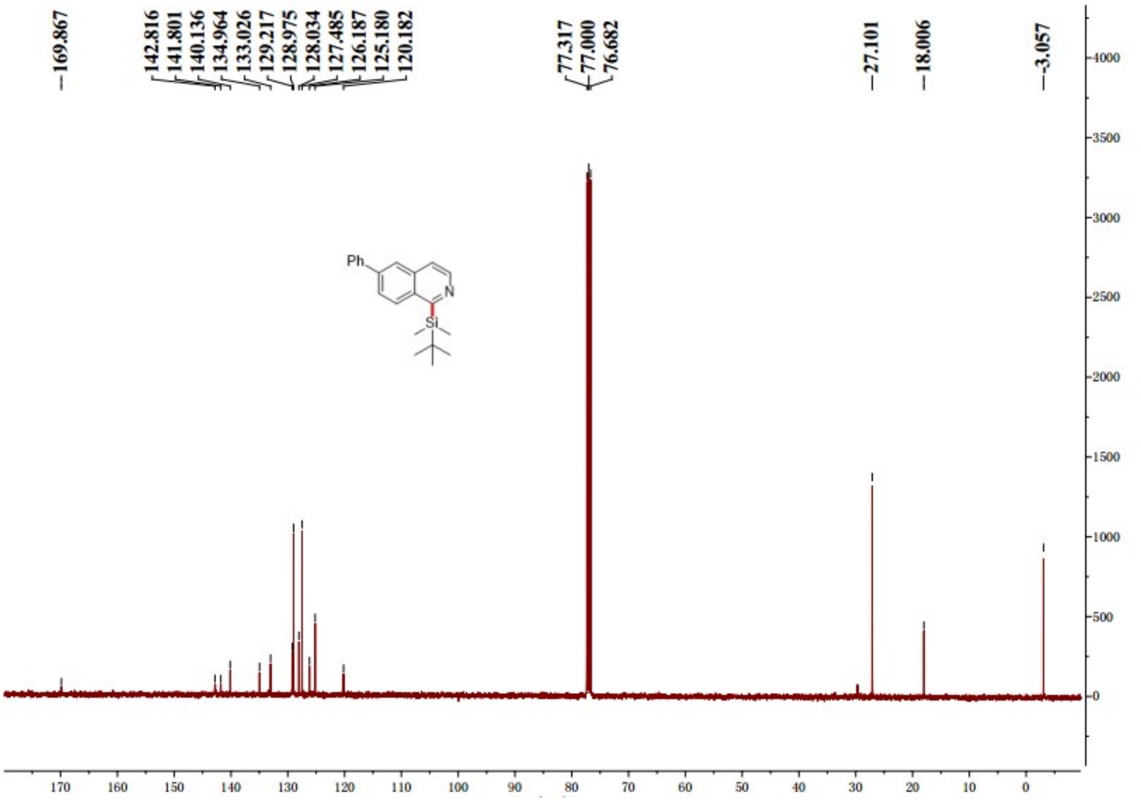

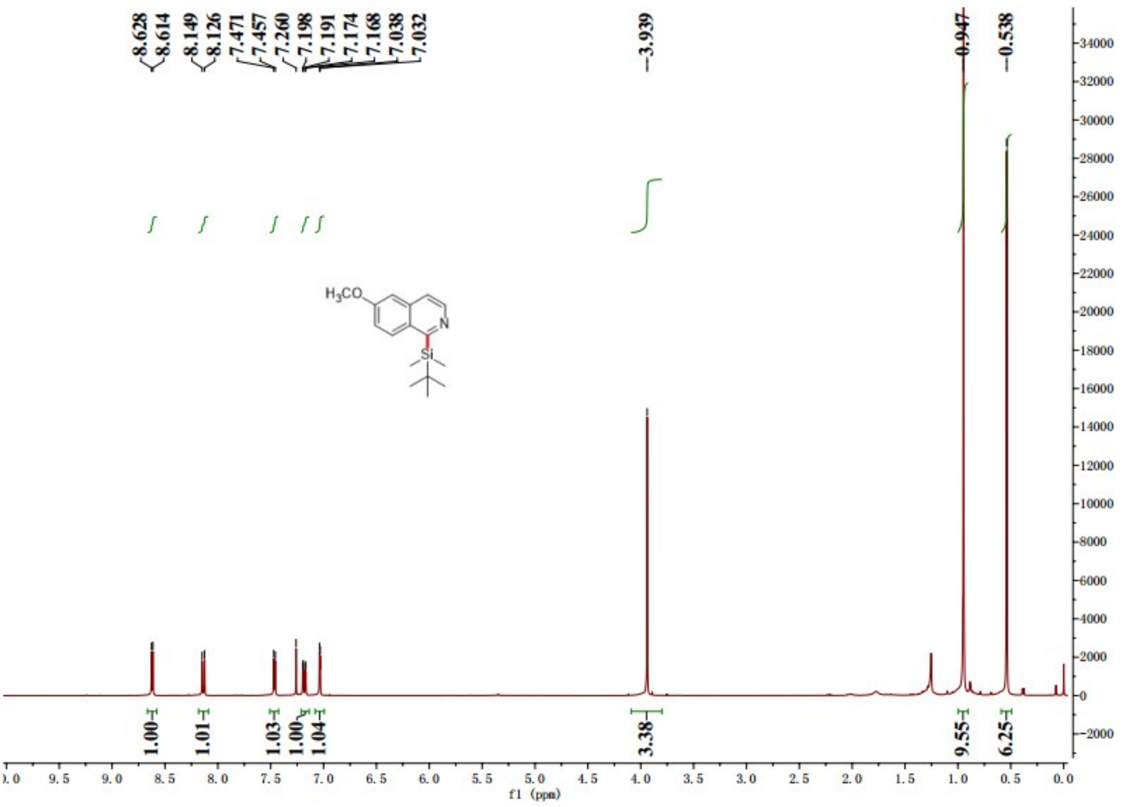

8.628
8.614
8.149
8.126
7.471
7.457
7.260
7.198
7.191
7.174
7.168
7.038
7.032
3.939
0.947
0.538
H_3CO
N
Si
1.00
1.01
1.03
1.00
1.04
3.38
9.55
6.25
f1 (ppm)

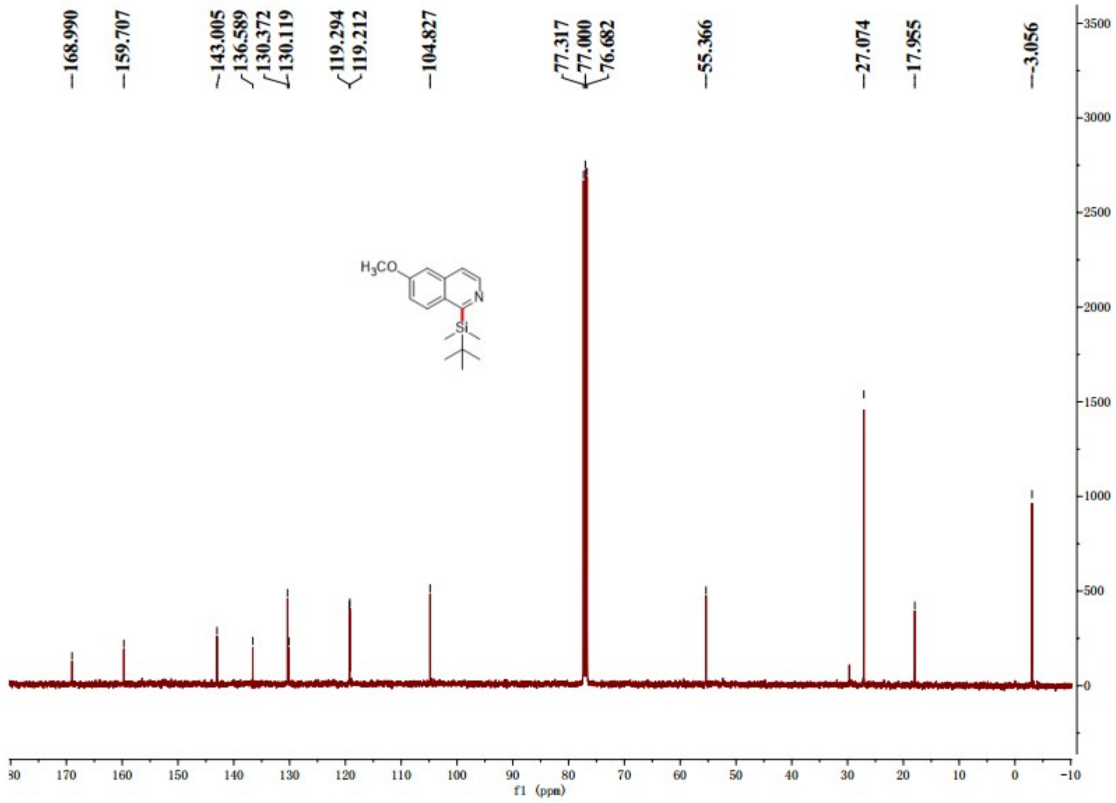

168.990
159.707
143.005
136.589
130.372
130.119
119.294
119.212
104.827
77.317
77.000
76.682
55.366
27.074
17.955
-3.056
H_3CO
N
Si
f1 (ppm)

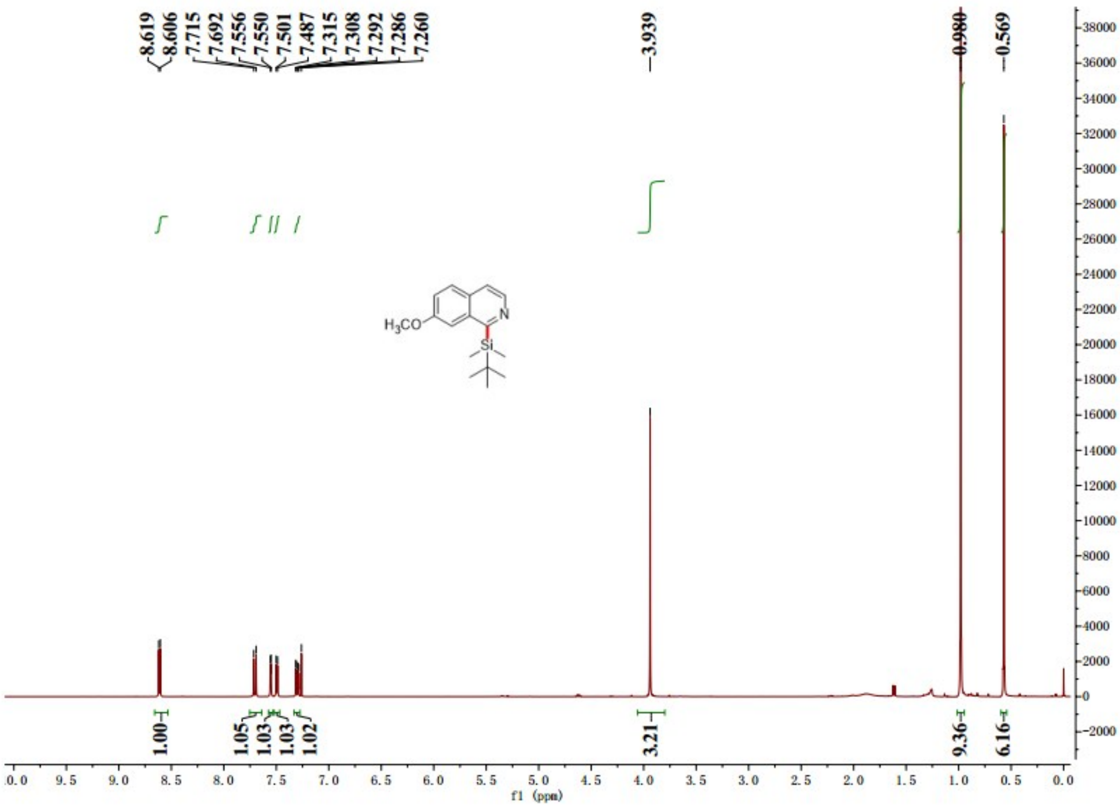
8.619
8.606
7.715
7.692
7.556
7.550
7.501
7.487
7.315
7.308
7.292
7.286
7.260
3.939
0.980
0.569
H3CO
N
Si
1.00
1.05
1.03
1.03
1.02
3.21
9.36
6.16
f1 (ppm)

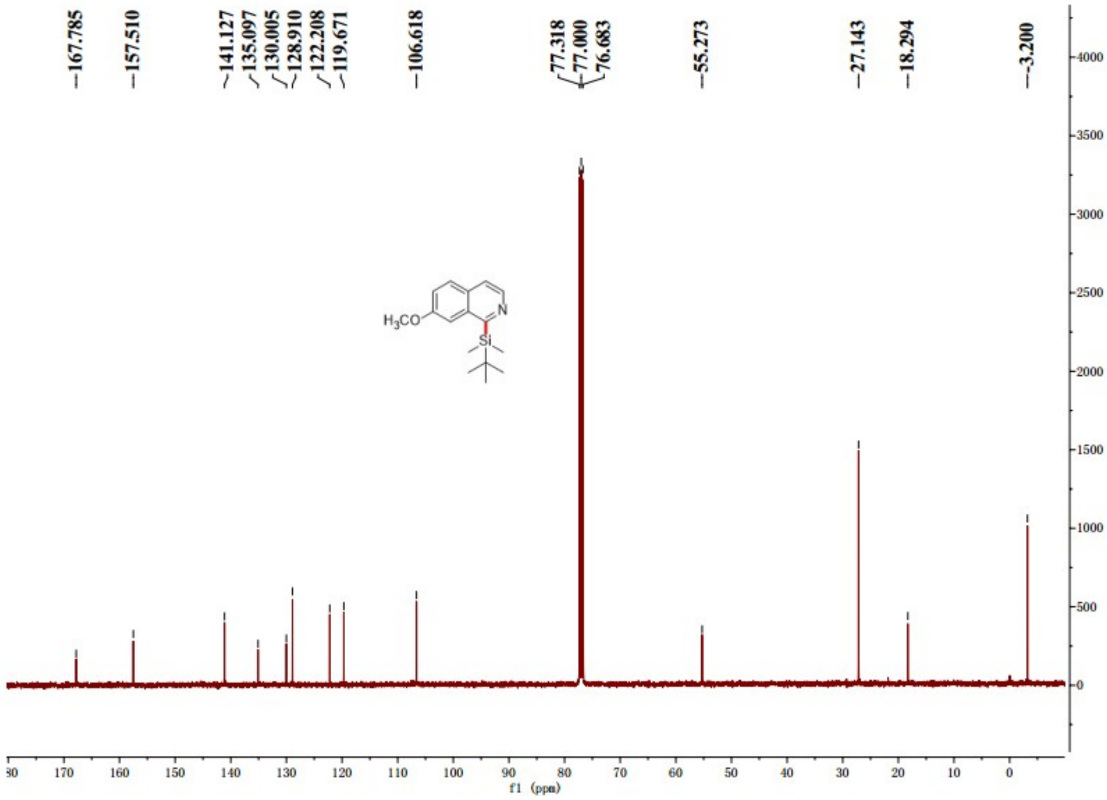
167.785
157.510
141.127
135.097
130.005
128.910
122.208
119.671
106.618
77.318
77.000
76.683
55.273
27.143
18.294
-3.200
H3CO
N
Si
f1 (ppm)

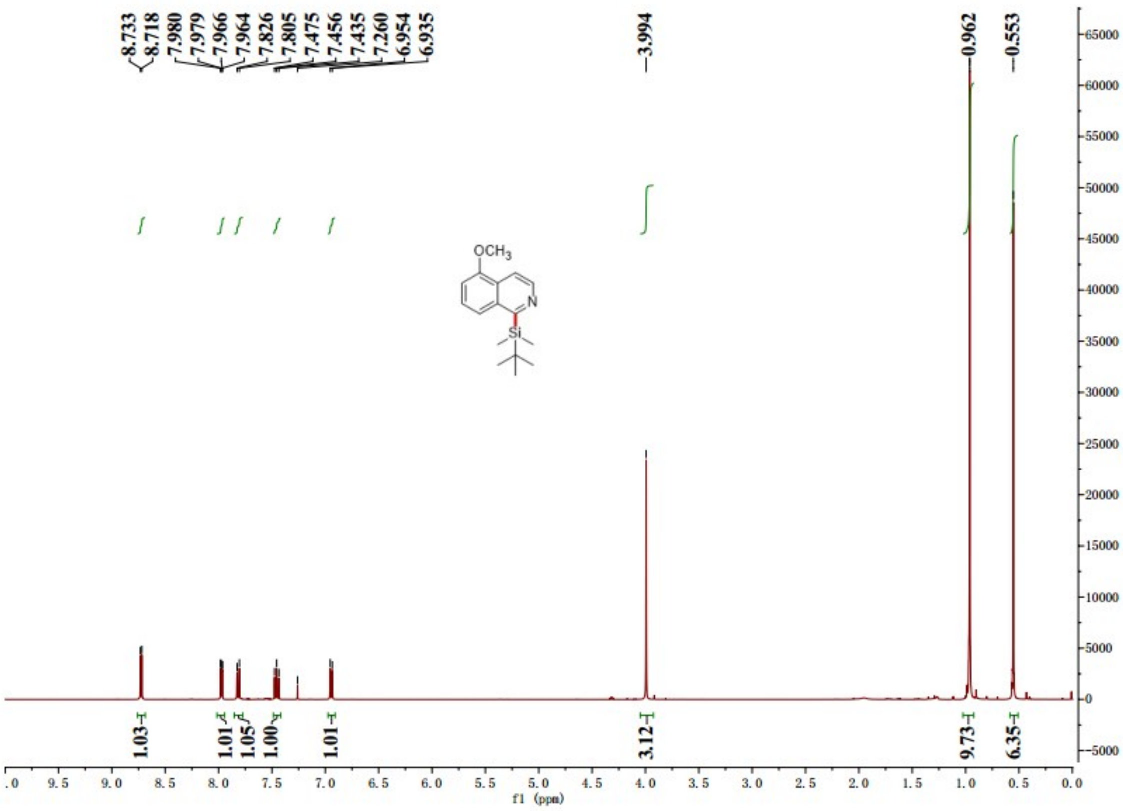

8.733
8.718
7.980
7.979
7.966
7.964
7.826
7.805
7.475
7.456
7.435
7.260
6.954
6.935
3.994
0.962
0.553
OCH3
N
Si
1.03
1.01
1.05
1.00
1.01
3.12
9.73
6.35
f1 (ppm)

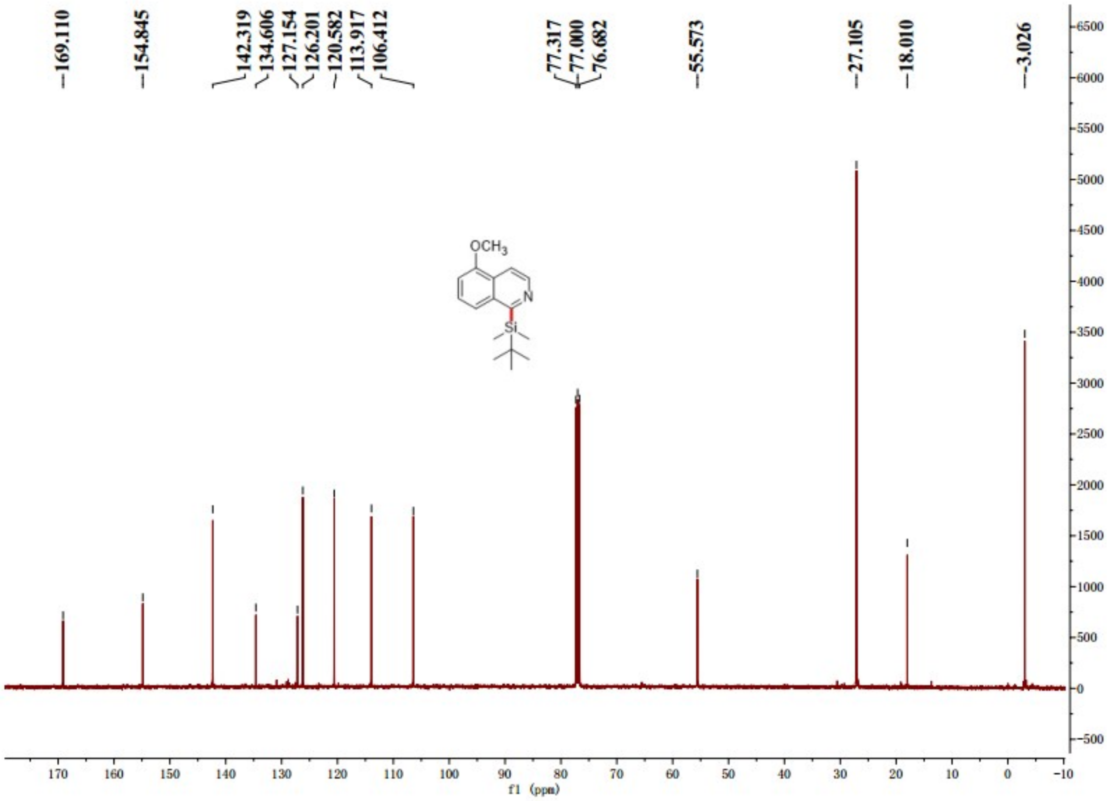

169.110
154.845
142.319
134.606
127.154
126.201
120.582
113.917
106.412
77.317
77.000
76.682
55.573
27.105
18.010
-3.026
OCH3
N
Si
f1 (ppm)

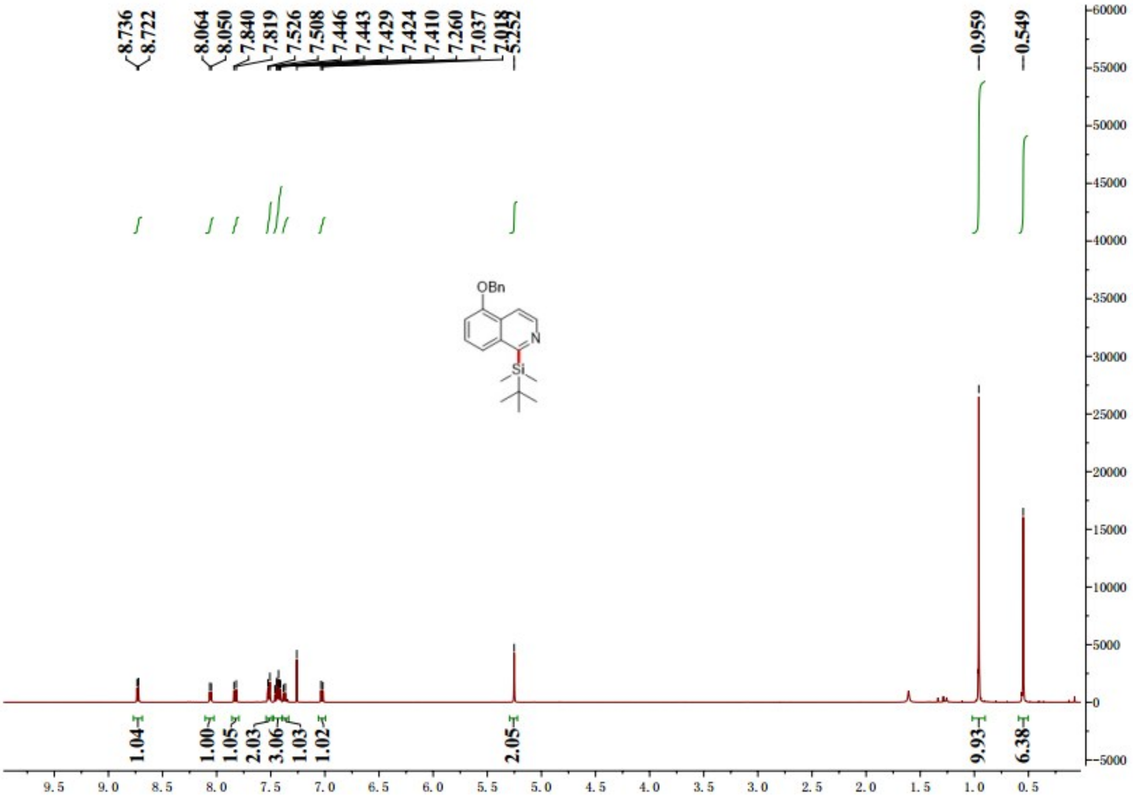
8.736
8.722
8.064
8.050
7.840
7.819
7.526
7.508
7.446
7.443
7.429
7.424
7.410
7.260
7.037
7.018
5.252
0.959
0.549
OBn
N
Si
1.04
1.00
1.05
2.03
3.06
1.03
1.02
2.05
9.93
6.38

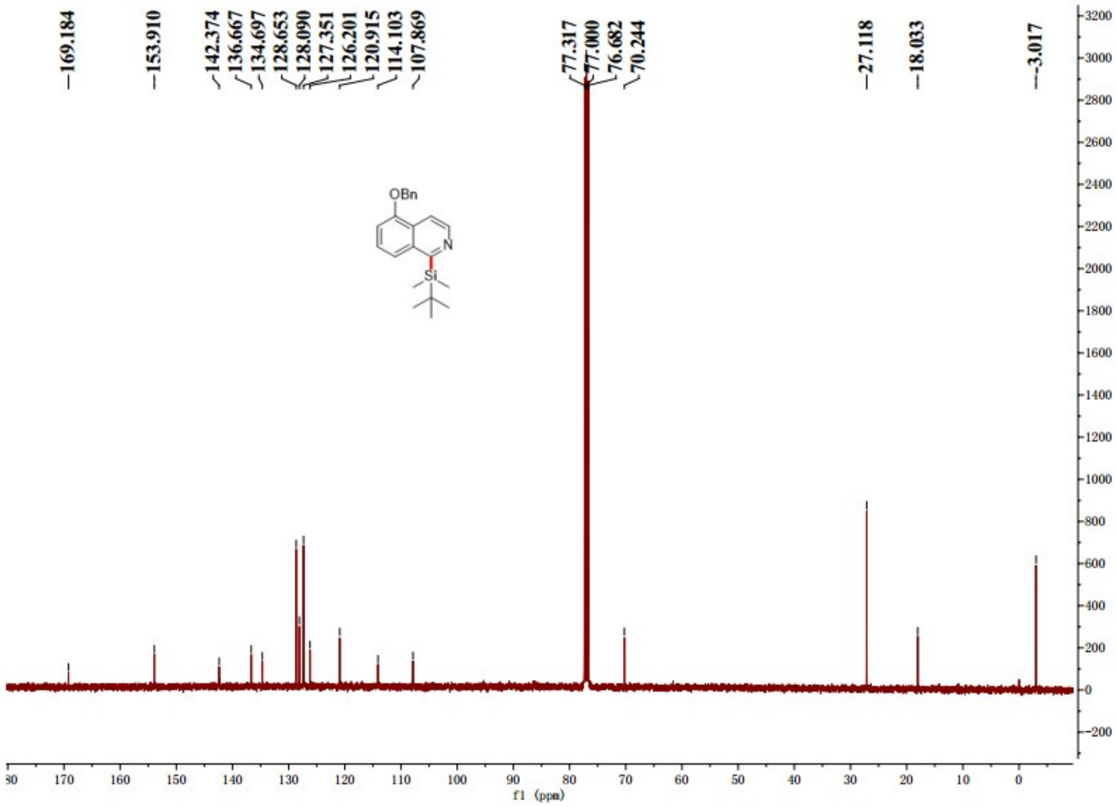
169.184
153.910
142.374
136.667
134.697
128.653
128.090
127.351
126.201
120.915
114.103
107.869
77.317
77.000
76.682
70.244
27.118
18.033
-3.017
OBn
N
Si
f1 (ppm)

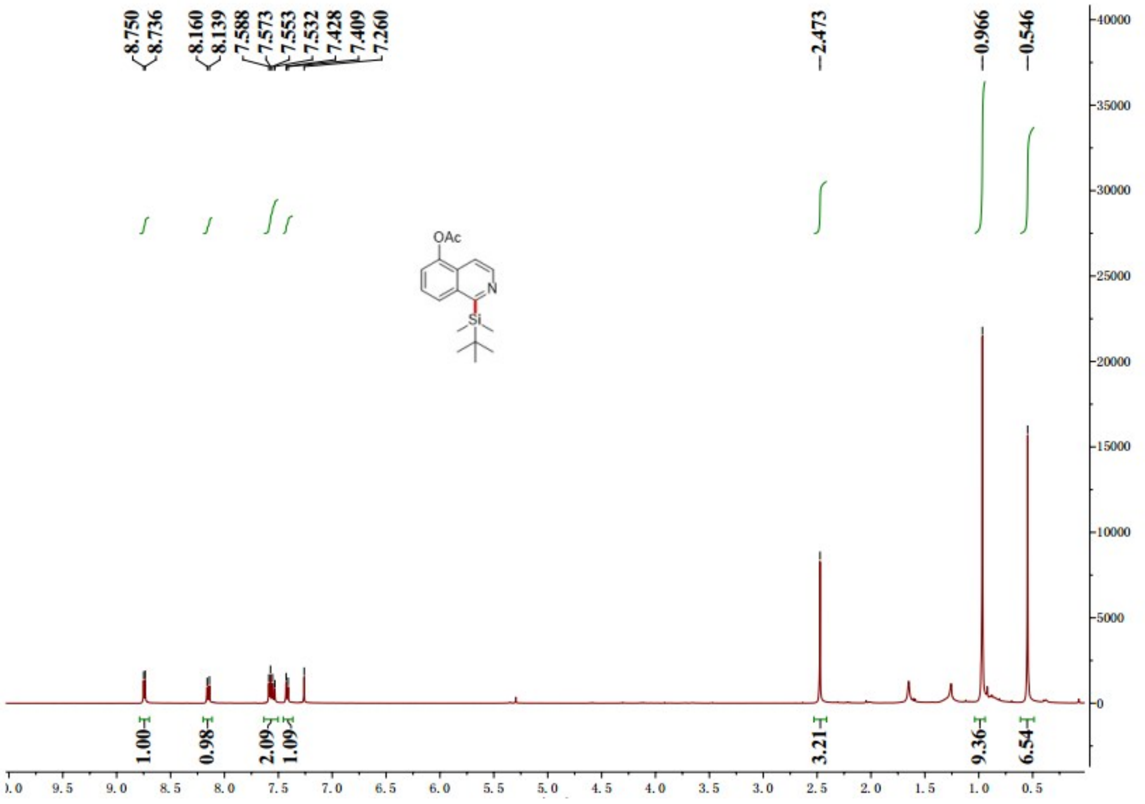
8.750
8.736
8.160
8.139
7.588
7.573
7.553
7.532
7.428
7.409
7.260
2.473
0.966
0.546
OAc
N
Si
1.00
0.98
2.09
1.09
3.21
9.36
6.54

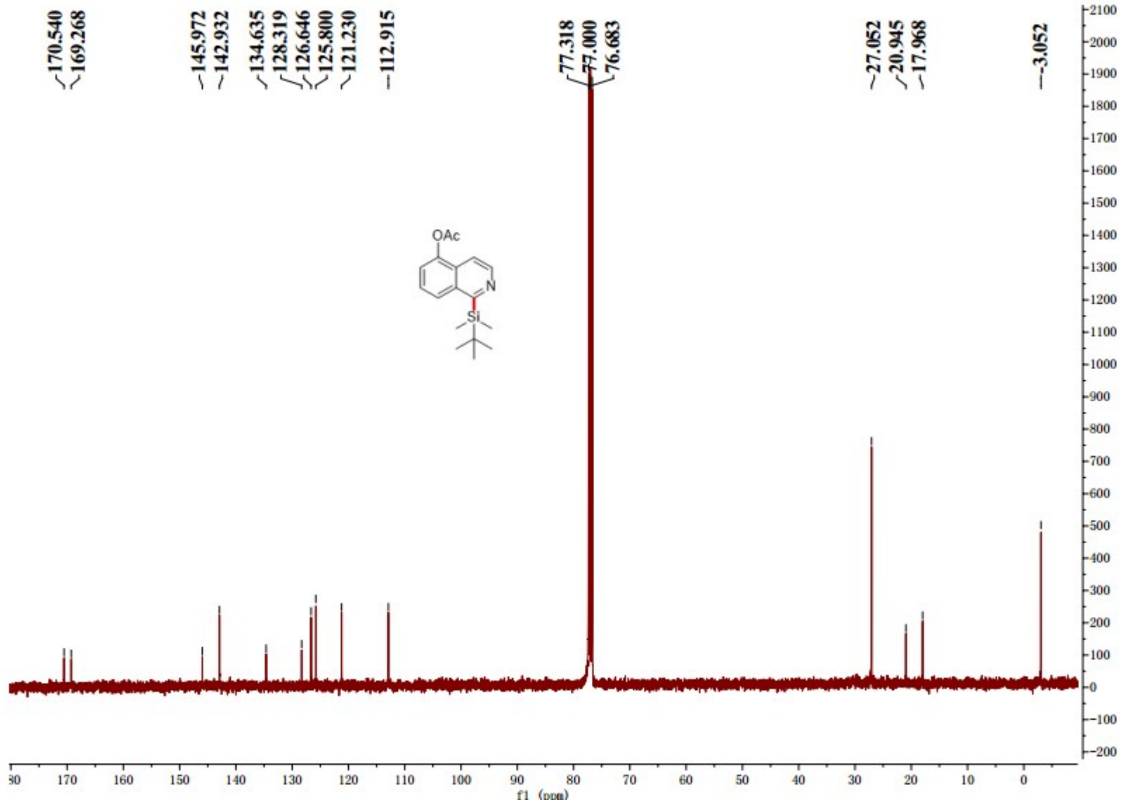
170.540
169.268
145.972
142.932
134.635
128.319
126.646
125.800
121.230
112.915
77.318
77.000
76.683
27.052
20.945
17.968
-3.052
OAc
N
Si
f1 (ppm)

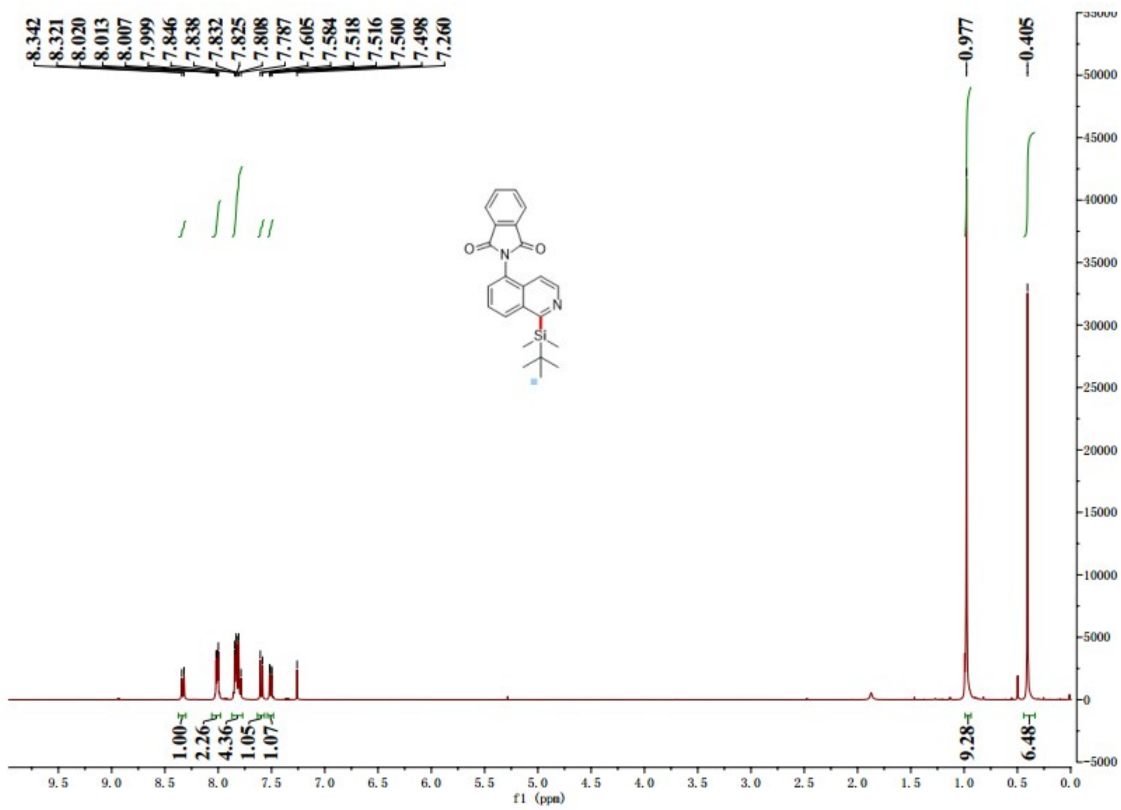
8.342
8.321
8.020
8.013
8.007
7.999
7.846
7.838
7.832
7.825
7.808
7.787
7.605
7.584
7.518
7.516
7.500
7.498
7.260
0.977
0.405
1.00
2.26
4.36
1.05
1.07
9.28
6.48
f1 (ppm)

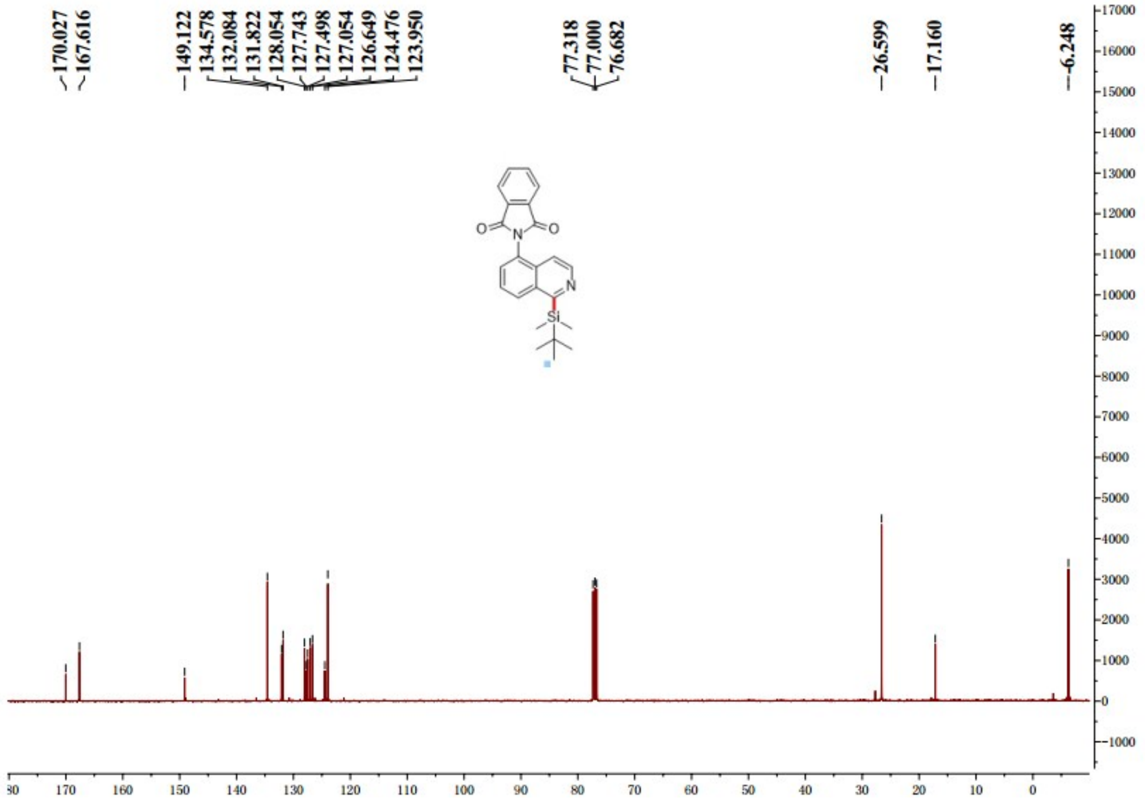
170.027
167.616
149.122
134.578
132.084
131.822
128.054
127.743
127.498
127.054
126.649
124.476
123.950
77.318
77.000
76.682
26.599
17.160
-6.248

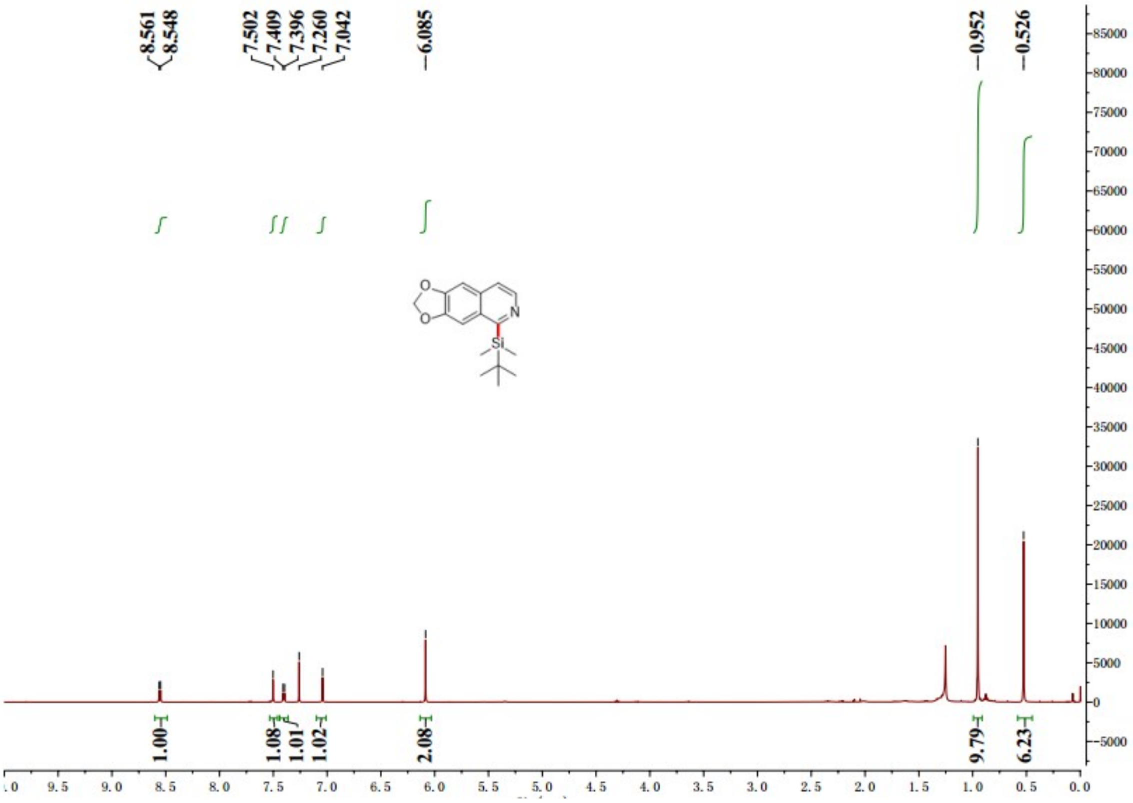

8.561
8.548
7.502
7.409
7.396
7.260
7.042
6.085
0.952
0.526
1.00
1.08
1.01
1.02
2.08
9.79
6.23
Si
N
O
O

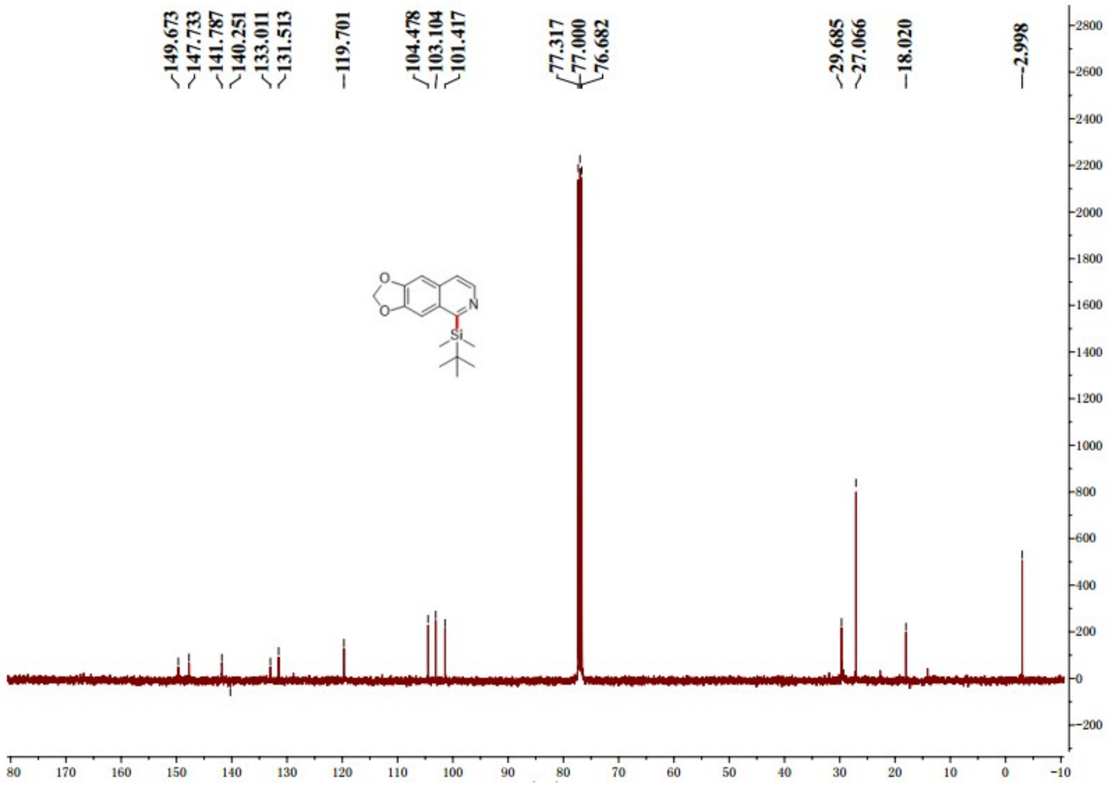

149.673
147.733
141.787
140.251
133.011
131.513
119.701
104.478
103.104
101.417
77.317
77.000
76.682
29.685
27.066
18.020
-2.998
Si
N
O
O

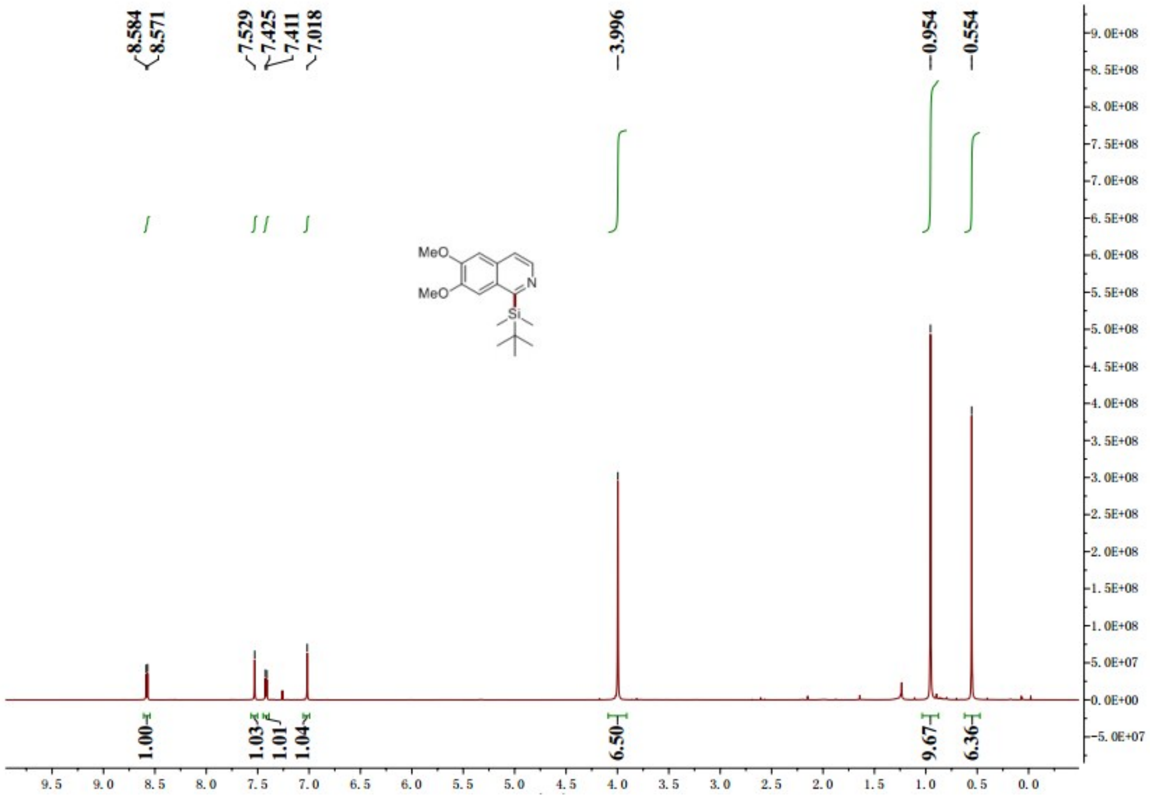
8.584
8.571
7.529
7.425
7.411
7.018
3.996
0.954
0.554
MeO
MeO
N
Si
1.00
1.03
1.01
1.04
6.50
9.67
6.36

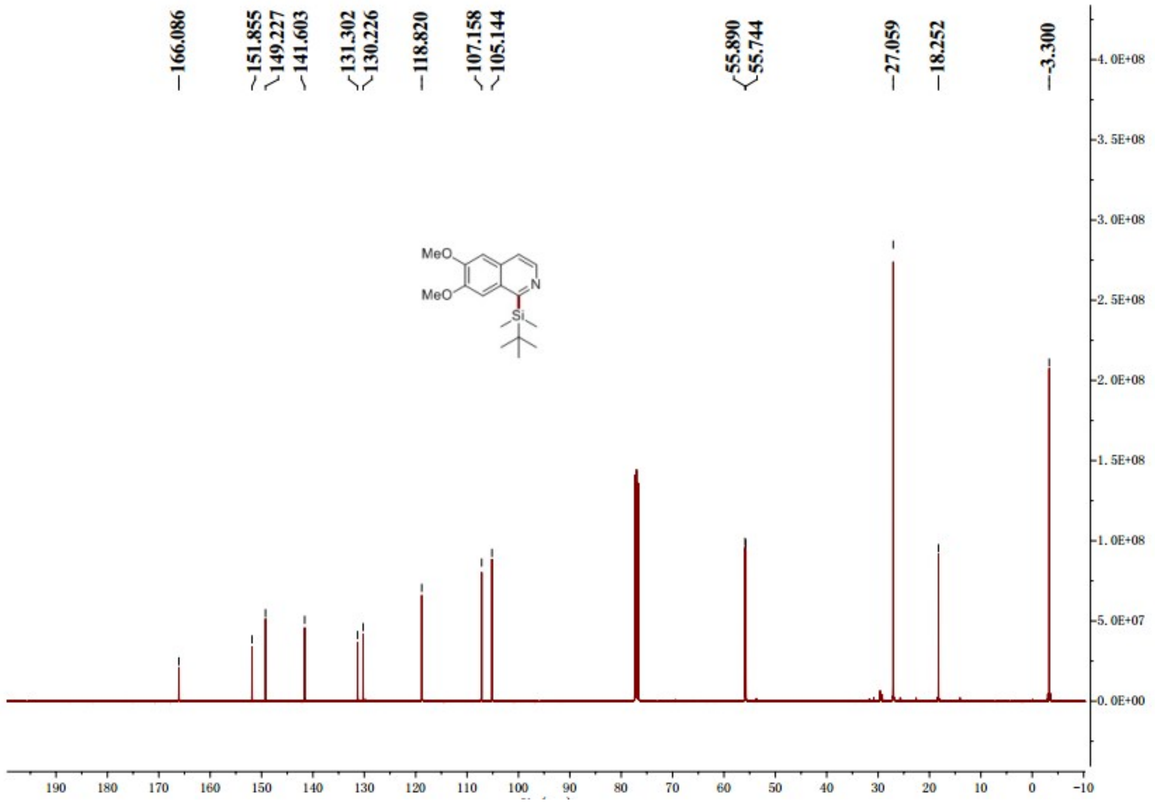
166.086
151.855
149.227
141.603
131.302
130.226
118.820
107.158
105.144
55.890
55.744
27.059
18.252
-3.300
MeO
MeO
N
Si

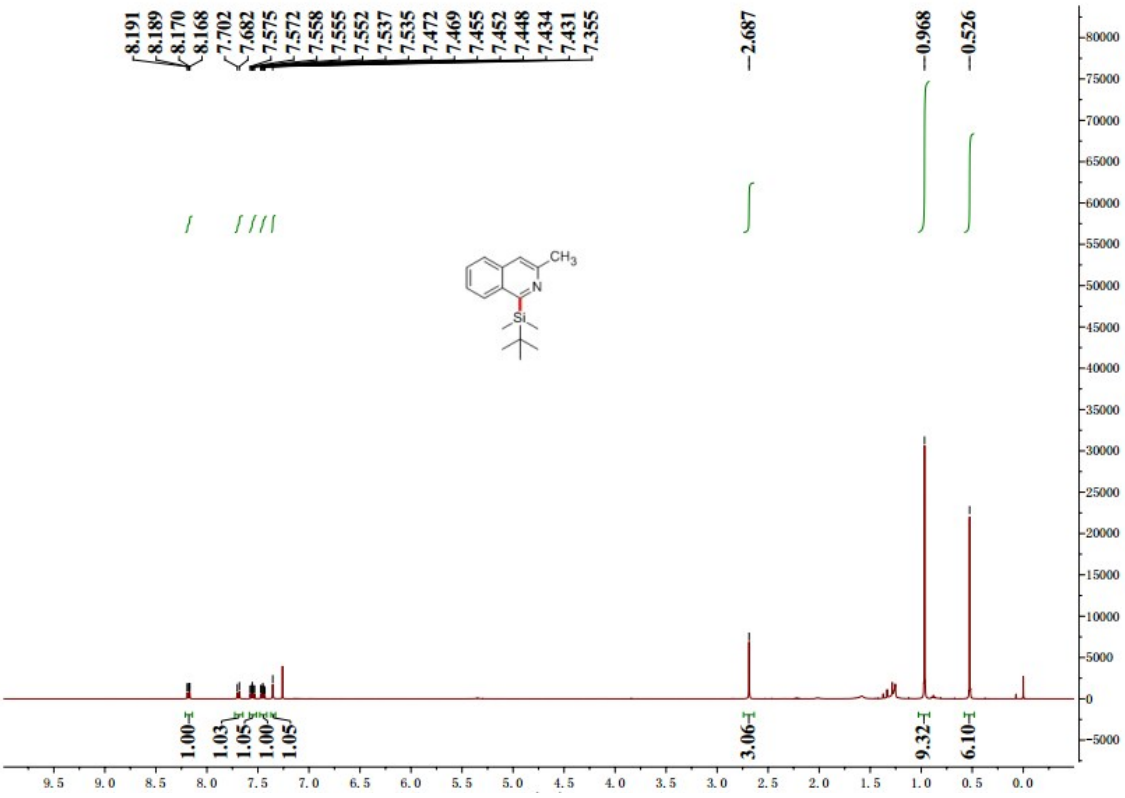
8.191 8.189 8.170 8.168 7.702 7.682 7.575 7.572 7.558 7.555 7.552 7.537 7.535 7.472 7.469 7.455 7.452 7.448 7.434 7.431 7.355
2.687
0.968
0.526
CH3
N
Si
1.00
1.03
1.05
1.00
1.05
3.06
9.32
6.10

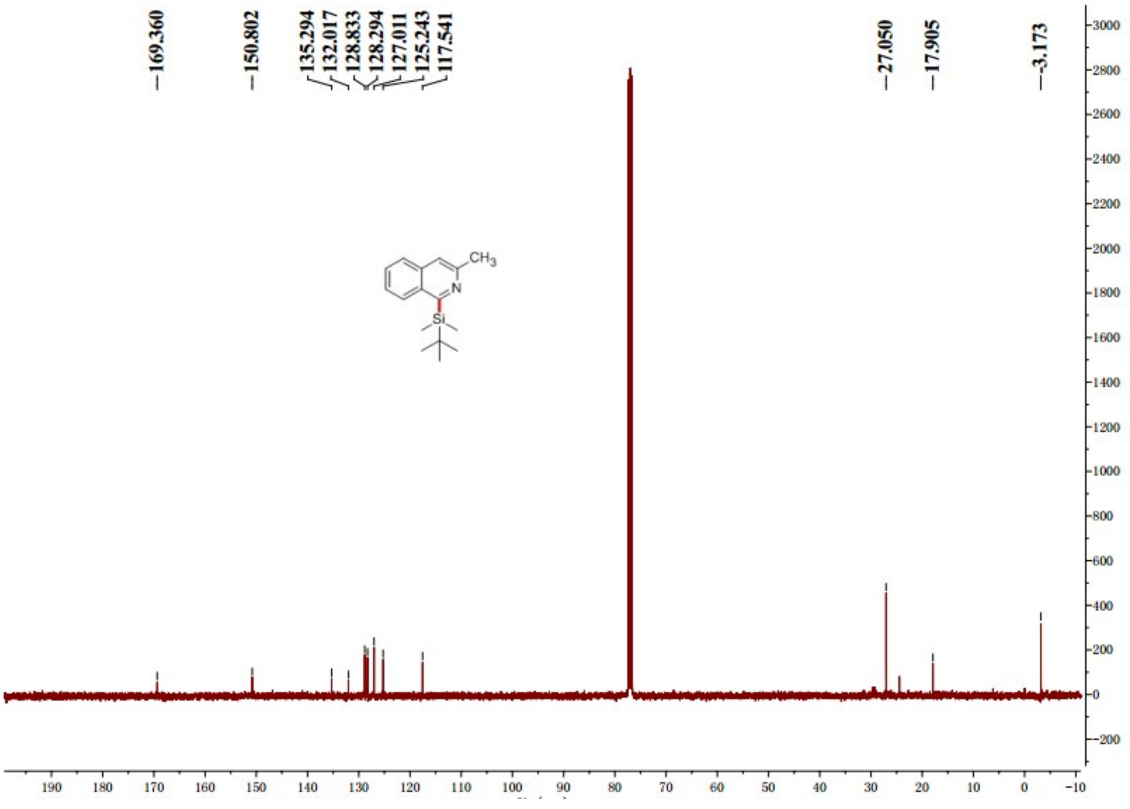
169.360
150.802
135.294 132.017 128.833 128.294 127.011 125.243 117.541
27.050
17.905
-3.173
CH3
N
Si

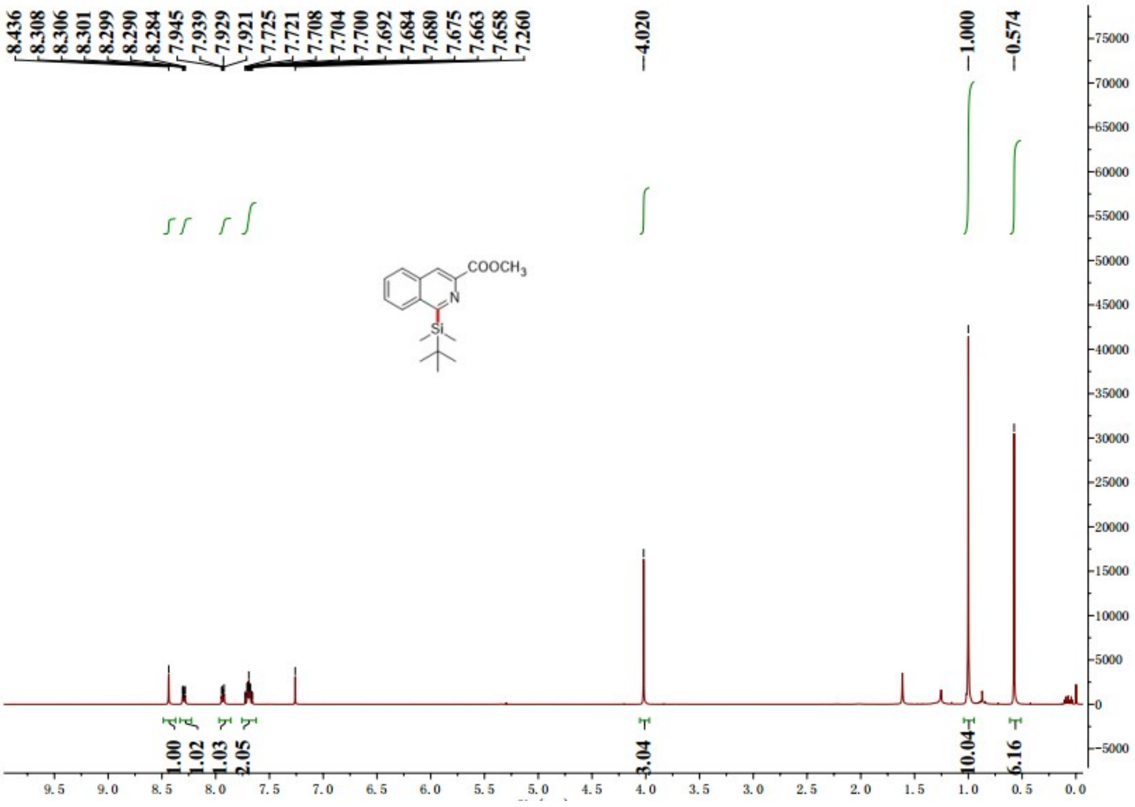
8.436
8.308
8.306
8.301
8.299
8.290
8.284
7.945
7.939
7.929
7.921
7.725
7.721
7.708
7.704
7.700
7.692
7.684
7.680
7.675
7.663
7.658
7.260
4.020
1.000
0.574
COOCH3
N
Si
1.00
1.02
1.03
2.05
3.04
10.04
6.16

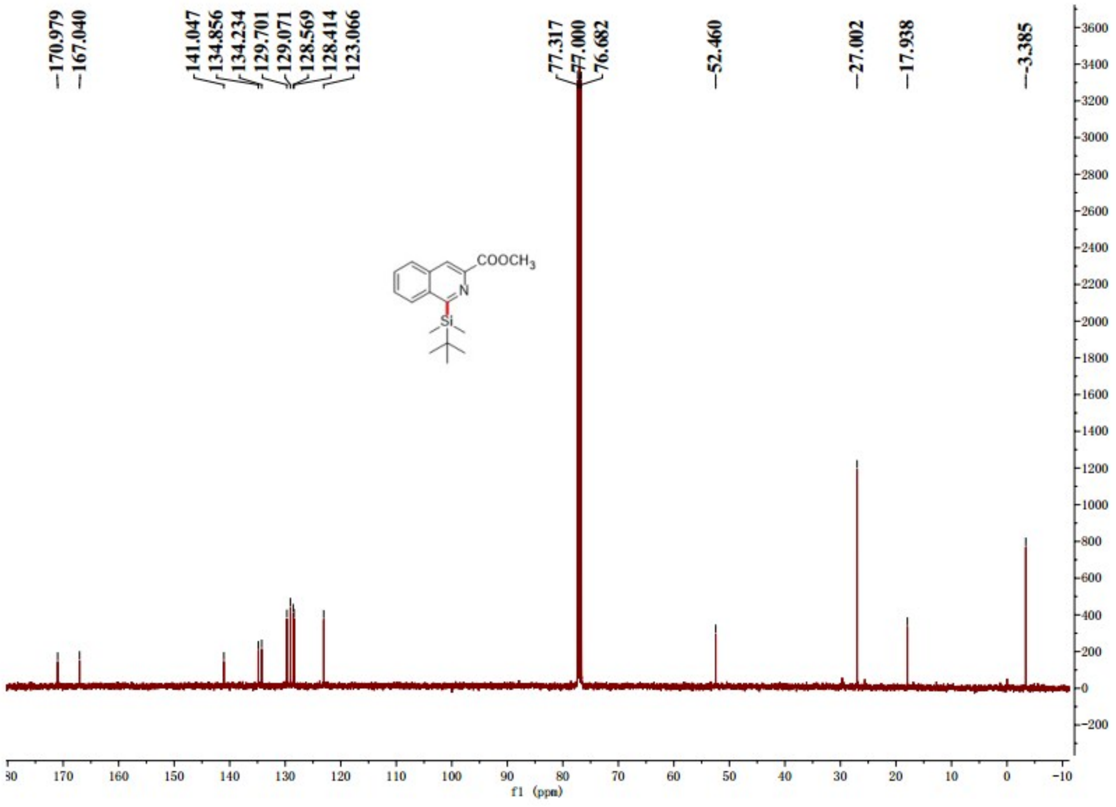
170.979
167.040
141.047
134.856
134.234
129.701
129.071
128.569
128.414
123.066
77.317
77.000
76.682
52.460
27.002
17.938
-3.385
COOCH3
N
Si
f1 (ppm)

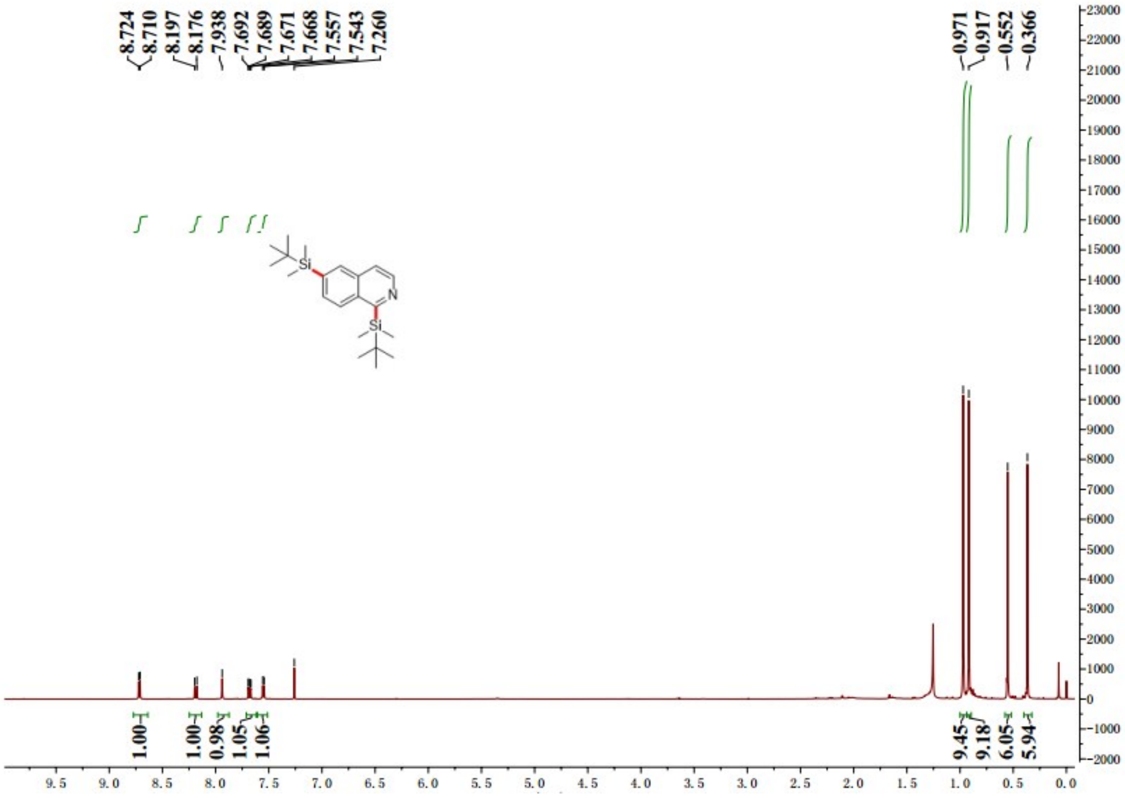
8.724
8.710
8.197
8.176
7.938
7.692
7.689
7.671
7.668
7.557
7.543
7.260
0.971
0.917
0.552
0.366
1.00
1.00
0.98
1.05
1.06
9.45
9.18
6.05
5.94
Si
Si
N

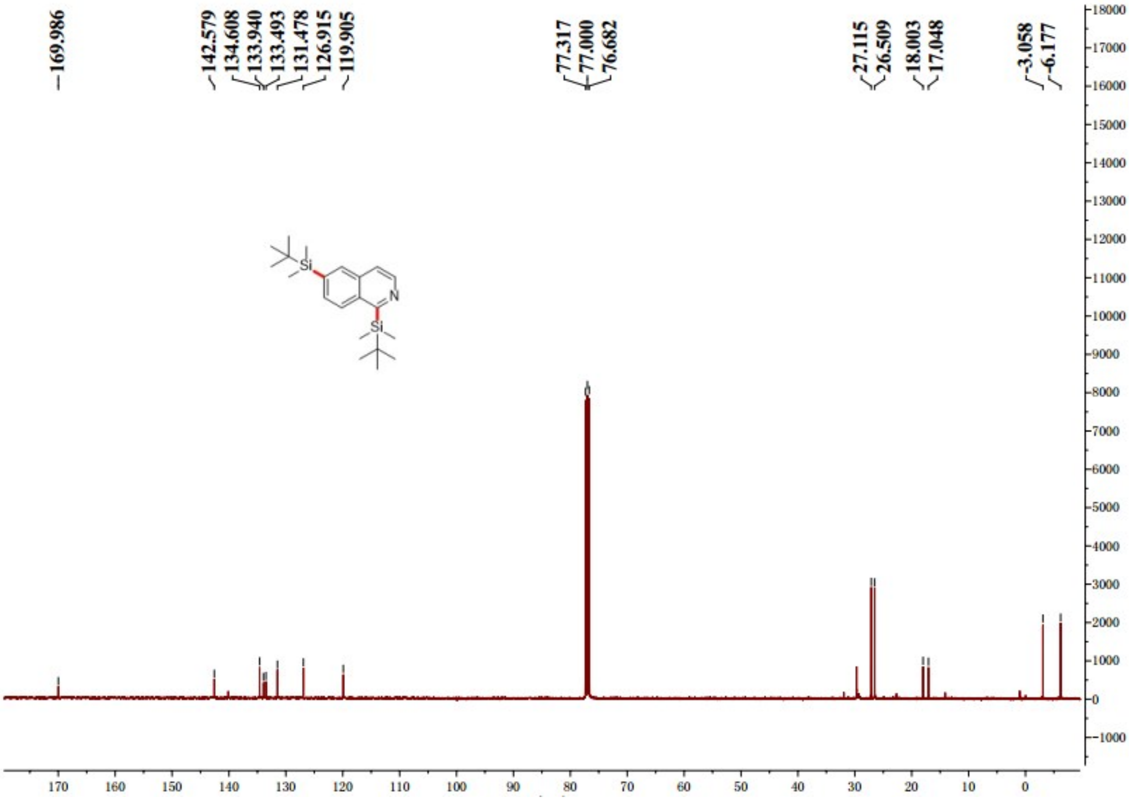
169.986
142.579
134.608
133.940
133.493
131.478
126.915
119.905
77.317
77.000
76.682
27.115
26.509
18.003
17.048
-3.058
-6.177
Si
Si
N

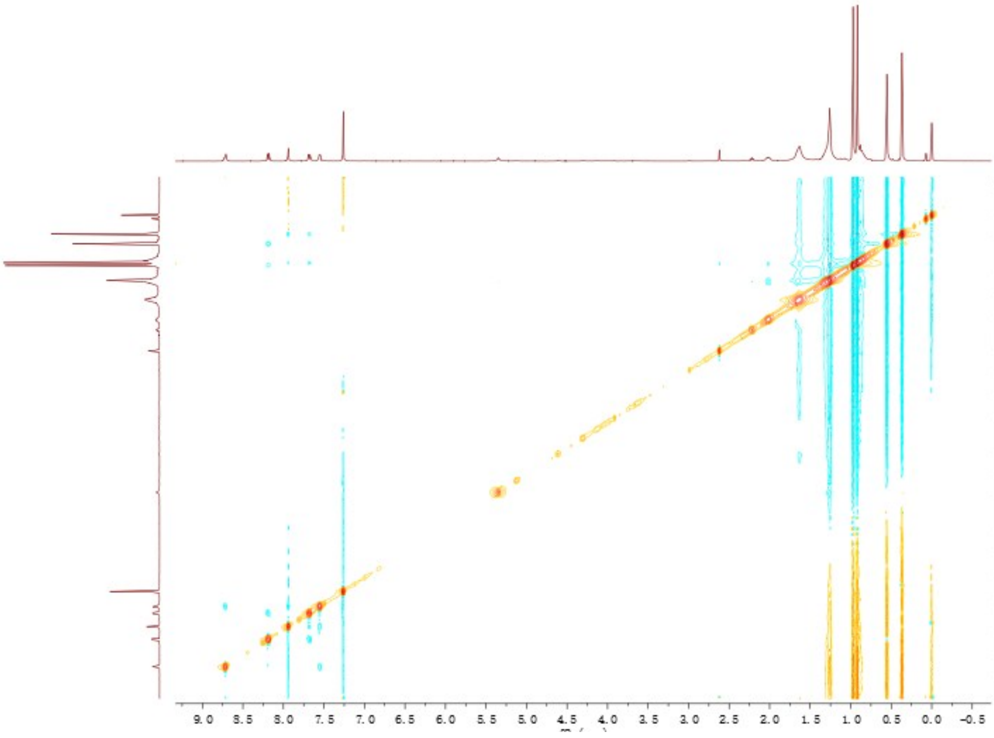

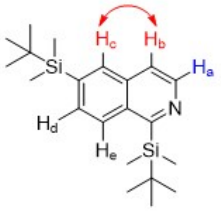

Si
N
Si

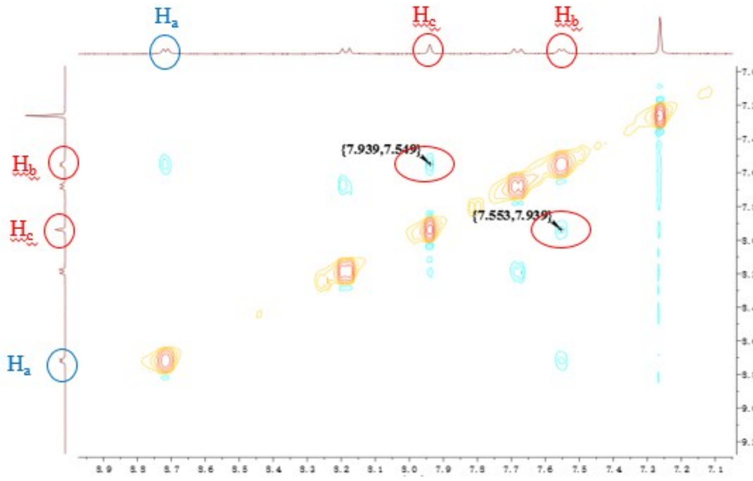

{7.939,7.549}
{7.553,7.939}

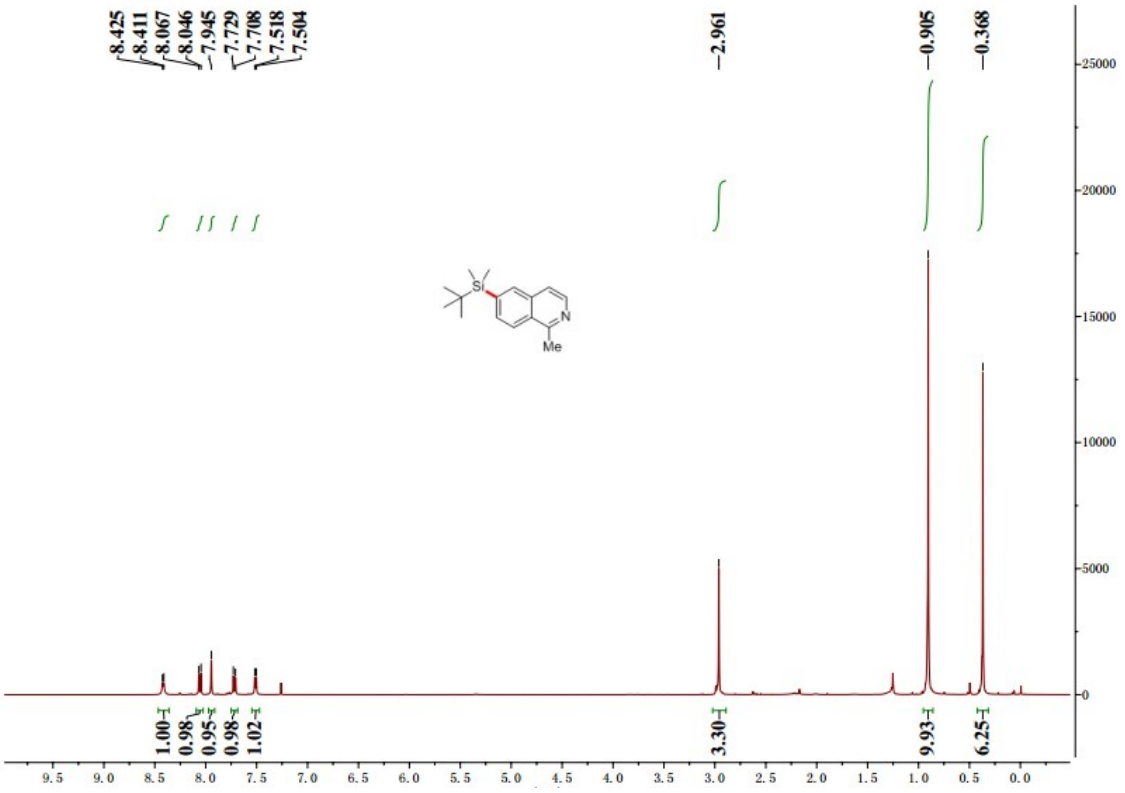
8.425
8.411
8.067
8.046
7.945
7.729
7.708
7.518
7.504
2.961
0.905
0.368
1.00
0.98
0.95
0.98
1.02
3.30
9.93
6.25
Si
N
Me

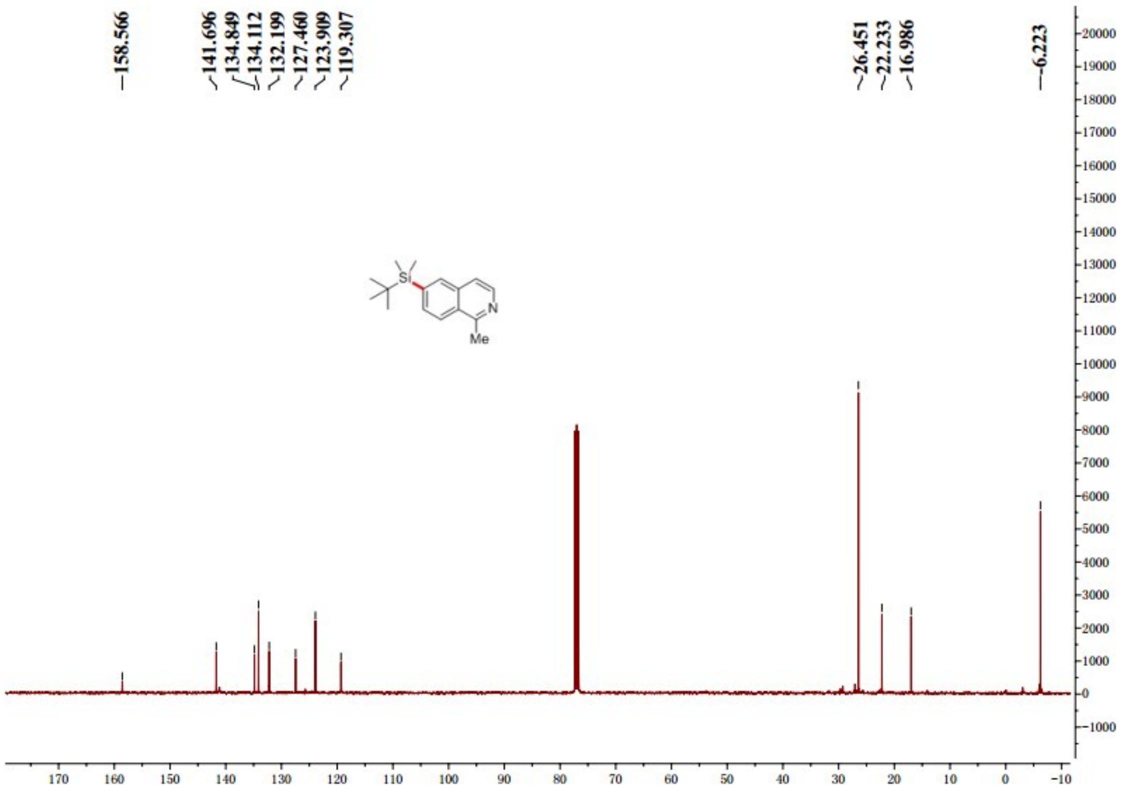
158.566
141.696
134.849
134.112
132.199
127.460
123.909
119.307
26.451
22.233
16.986
-6.223
Si
N
Me

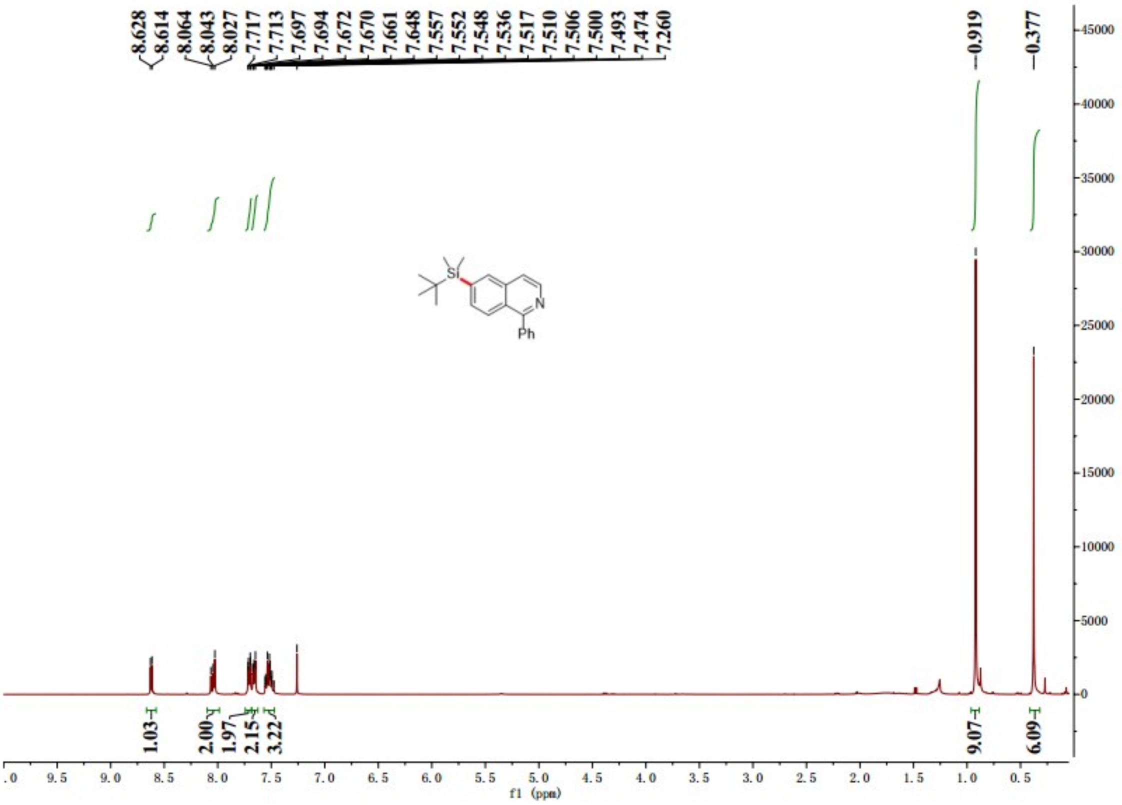
8.628
8.614
8.064
8.043
8.027
7.717
7.713
7.697
7.694
7.672
7.670
7.661
7.648
7.557
7.552
7.548
7.536
7.517
7.510
7.506
7.500
7.493
7.474
7.260
0.919
0.377
Si
N
Ph
1.03
2.00
1.97
2.15
3.22
9.07
6.09
f1 (ppm)

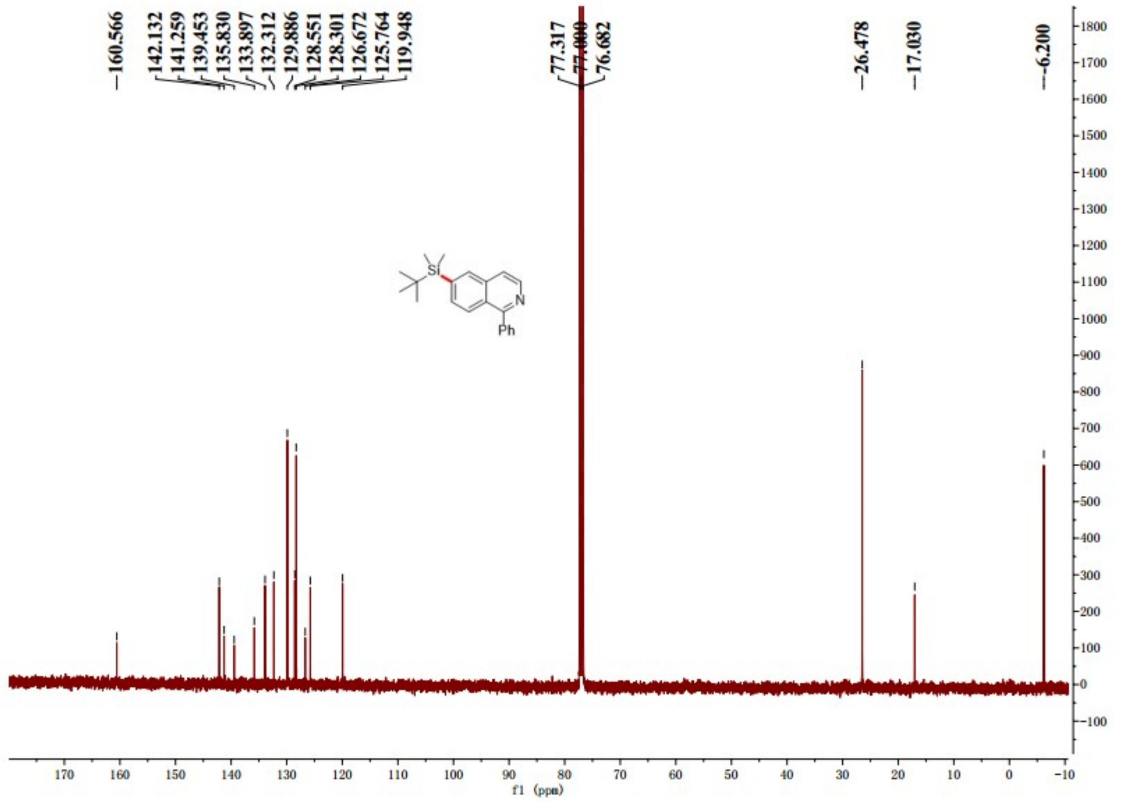
160.566
142.132
141.259
139.453
135.830
133.897
132.312
129.886
128.551
128.301
126.672
125.764
119.948
77.317
77.000
76.682
26.478
17.030
-6.200
Si
N
Ph
f1 (ppm)

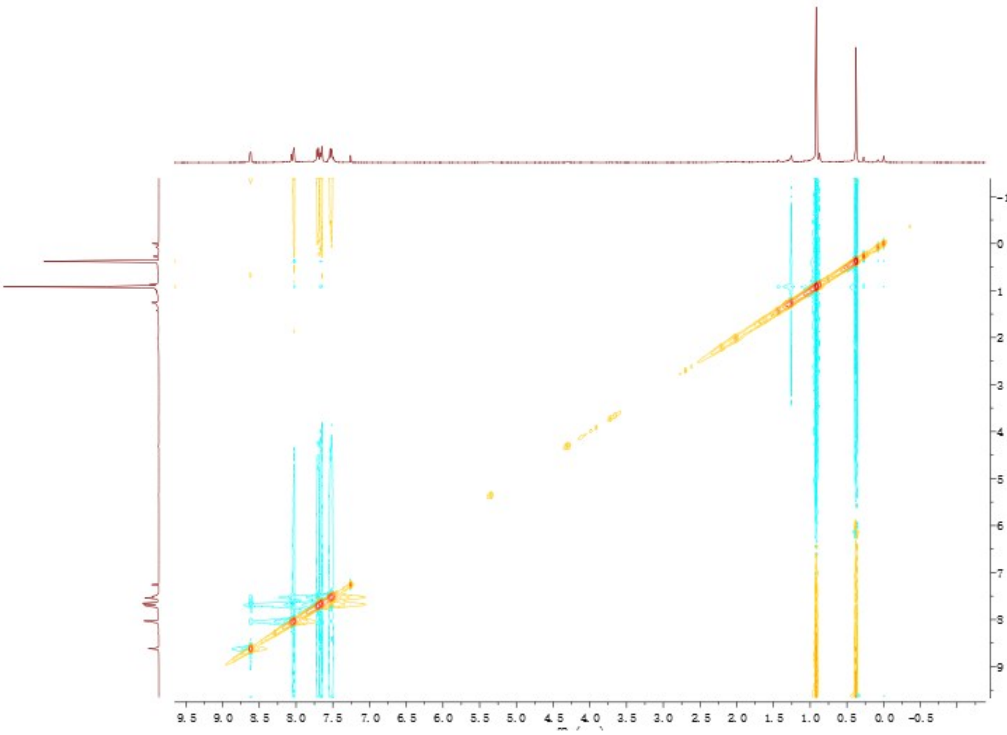

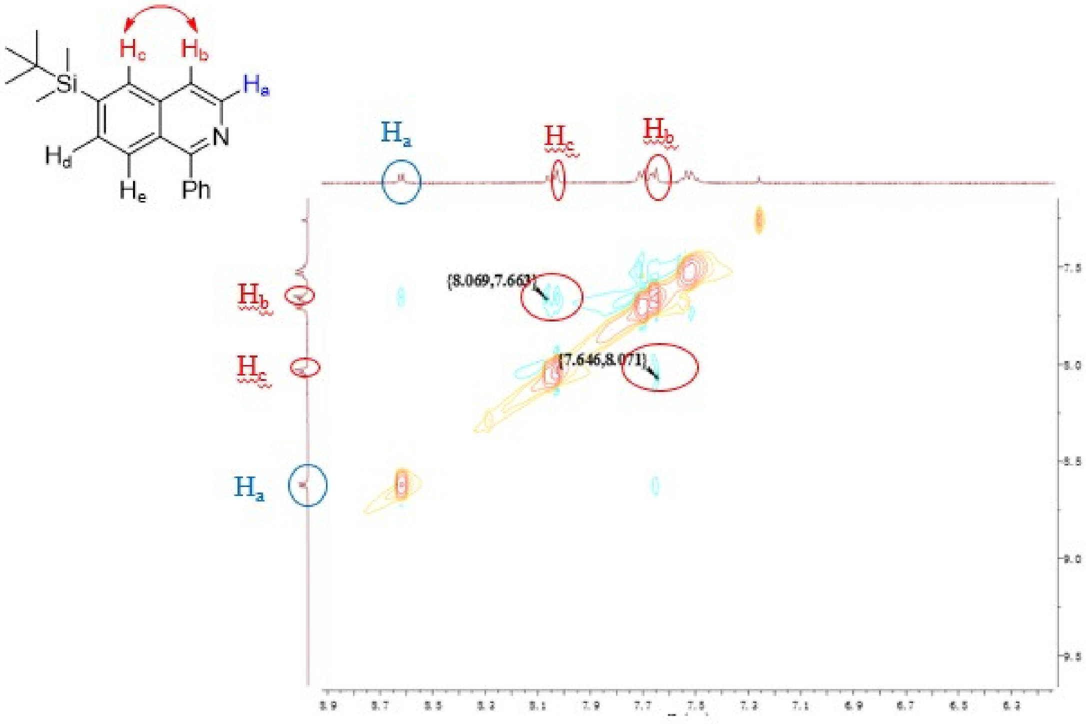
Si
Hc
Hb
Ha
N
Hd
He
Ph
Ha
Hc
Hb
(8.069,7.663)
(7.646,8.071)

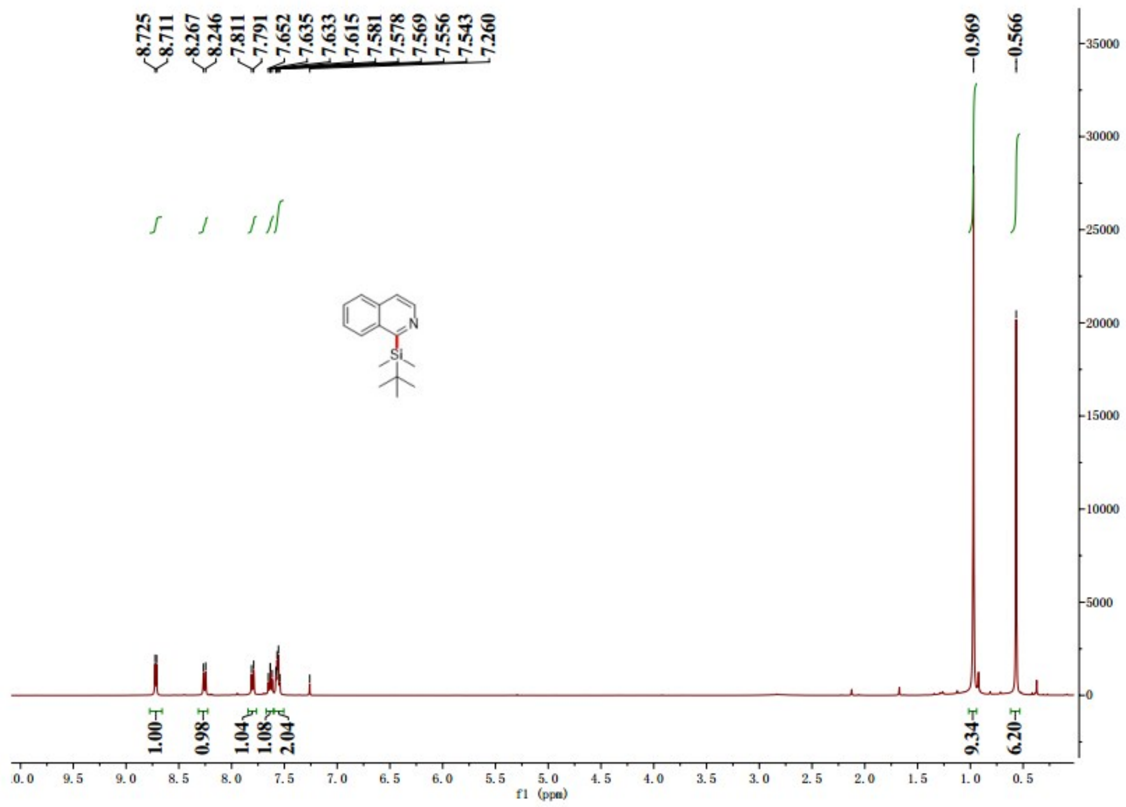
8.725
8.711
8.267
8.246
7.811
7.791
7.652
7.635
7.633
7.615
7.581
7.578
7.569
7.556
7.543
7.260
0.969
0.566
1.00
0.98
1.04
1.08
2.04
9.34
6.20
f1 (ppm)

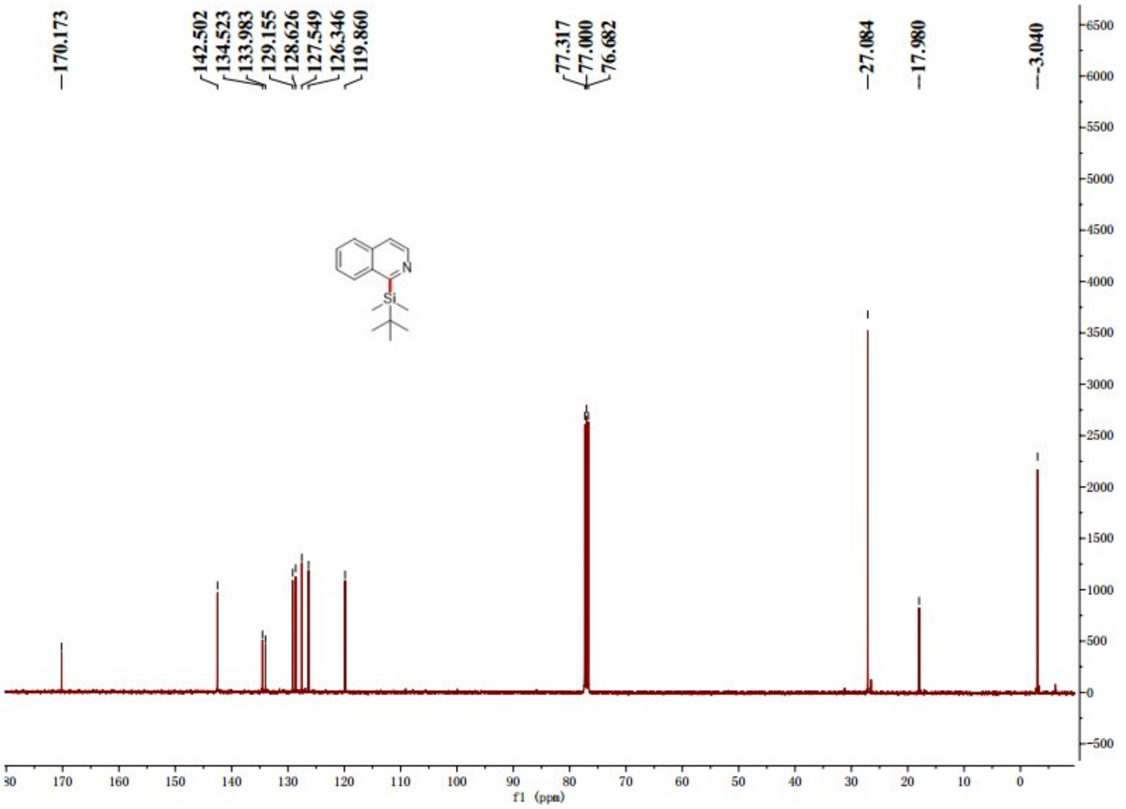
170.173
142.502
134.523
133.983
129.155
128.626
127.549
126.346
119.860
77.317
77.000
76.682
27.084
17.980
-3.040
f1 (ppm)

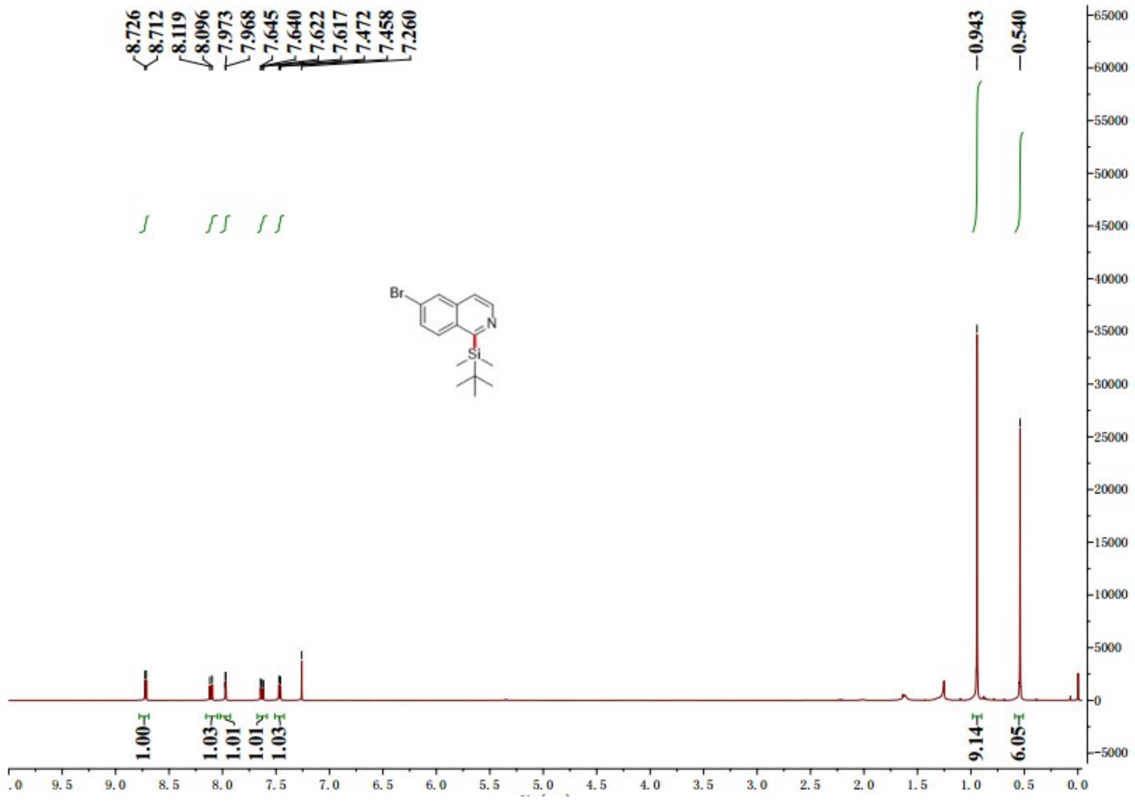
8.726
8.712
8.119
8.096
7.973
7.968
7.645
7.640
7.622
7.617
7.472
7.458
7.260
0.943
0.540
Br
N
Si
1.00
1.03
1.01
1.01
1.03
9.14
6.05

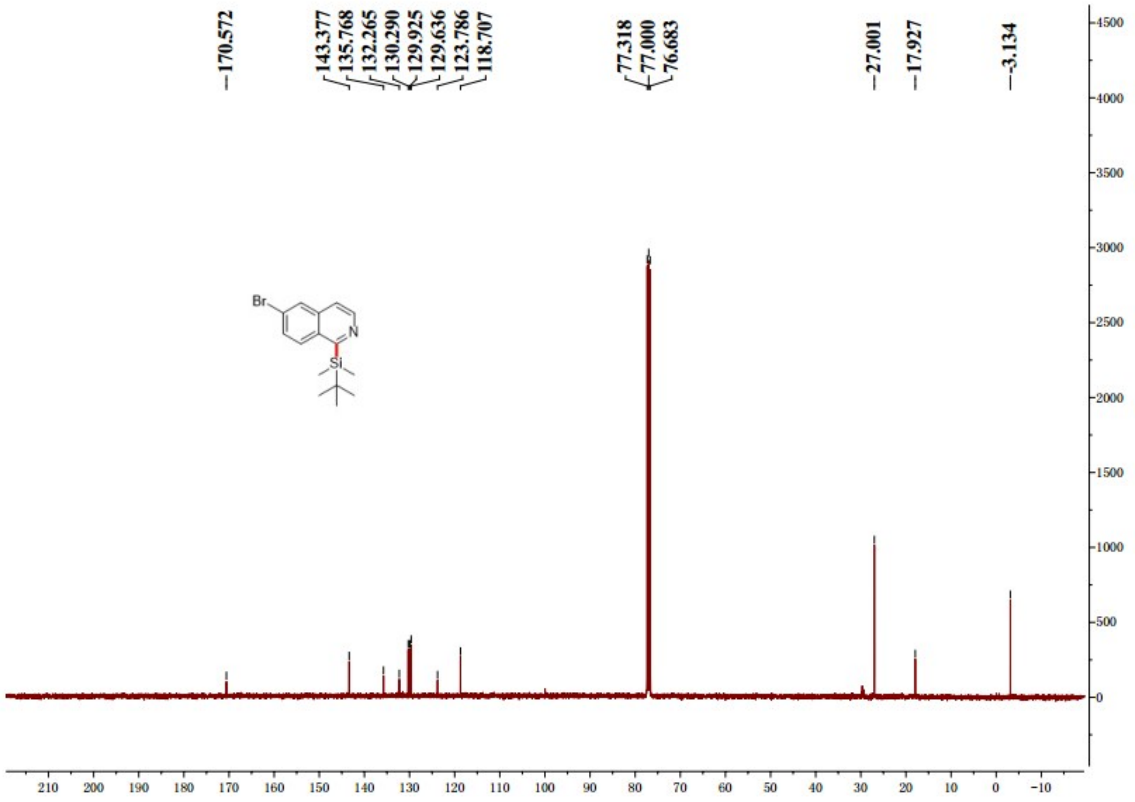
170.572
143.377
135.768
132.265
130.290
129.925
129.636
123.786
118.707
77.318
77.000
76.683
27.001
17.927
-3.134
Br
N
Si

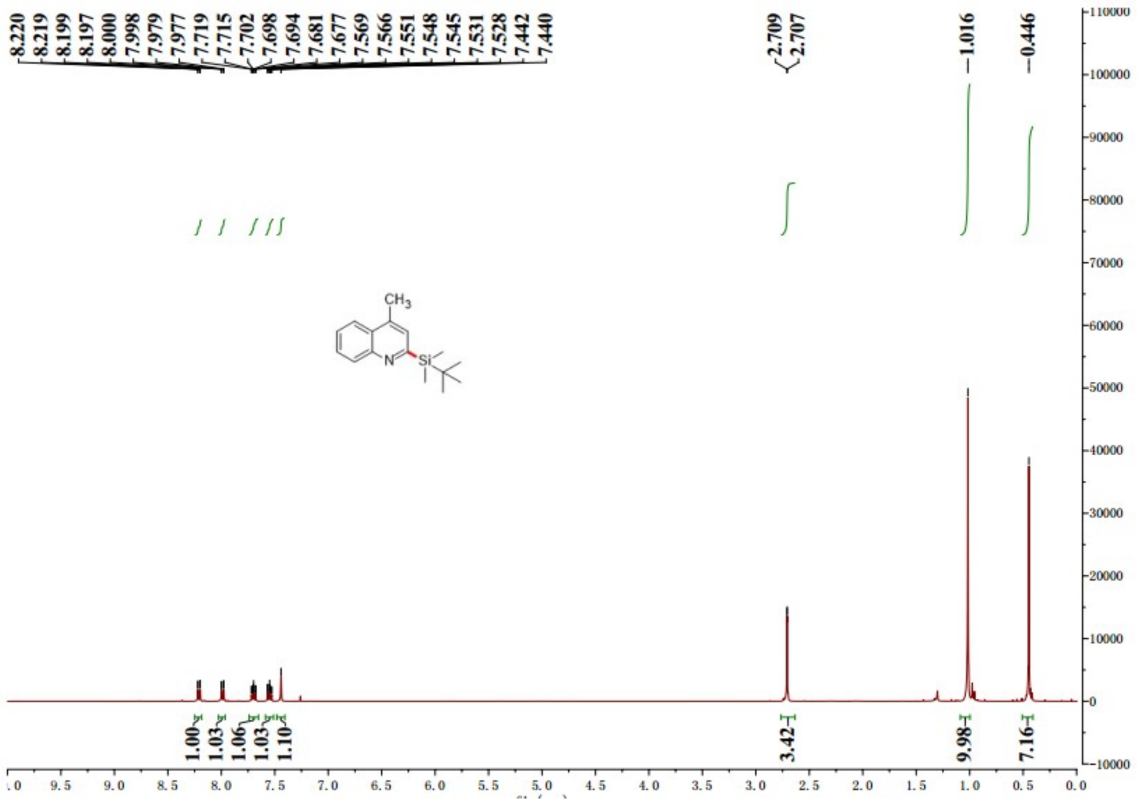
8.220
8.219
8.199
8.197
8.000
7.998
7.979
7.977
7.719
7.715
7.702
7.698
7.694
7.681
7.677
7.569
7.566
7.551
7.548
7.545
7.531
7.528
7.442
7.440
2.709
2.707
1.016
0.446
CH3
N
Si
1.00
1.03
1.06
1.03
1.10
3.42
9.98
7.16

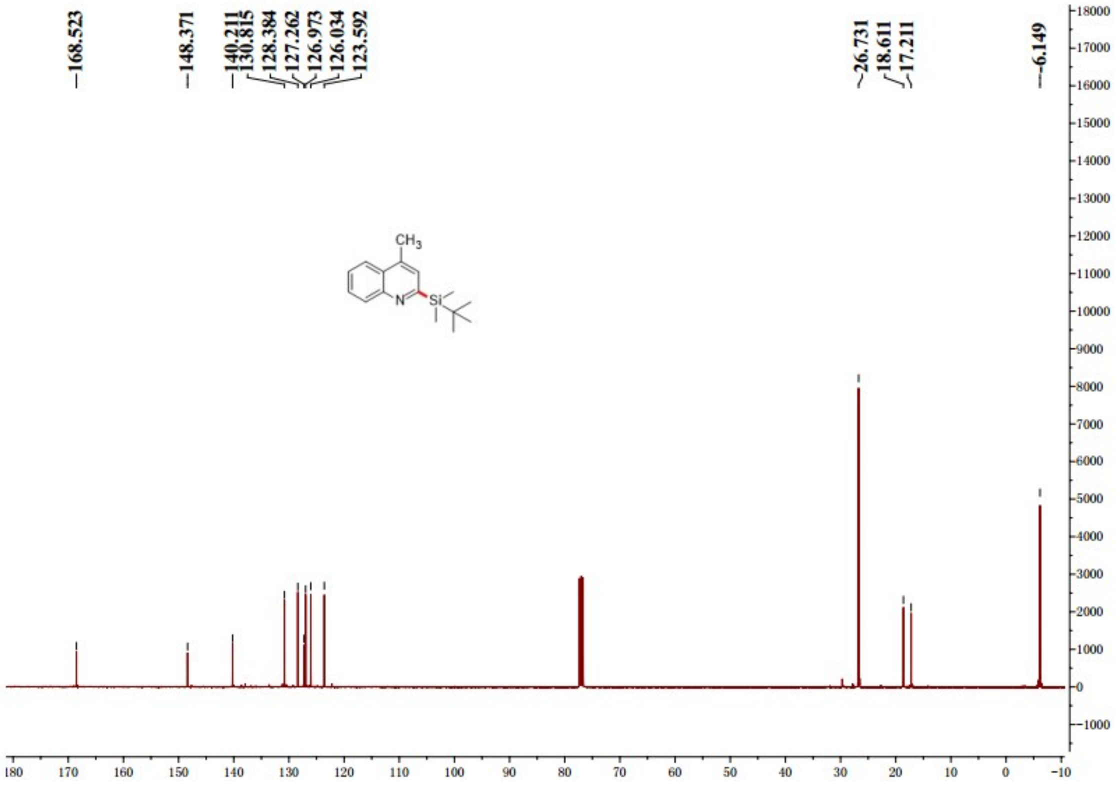
168.523
148.371
140.211
130.815
128.384
127.262
126.973
126.034
123.592
26.731
18.611
17.211
-6.149
CH3
N
Si

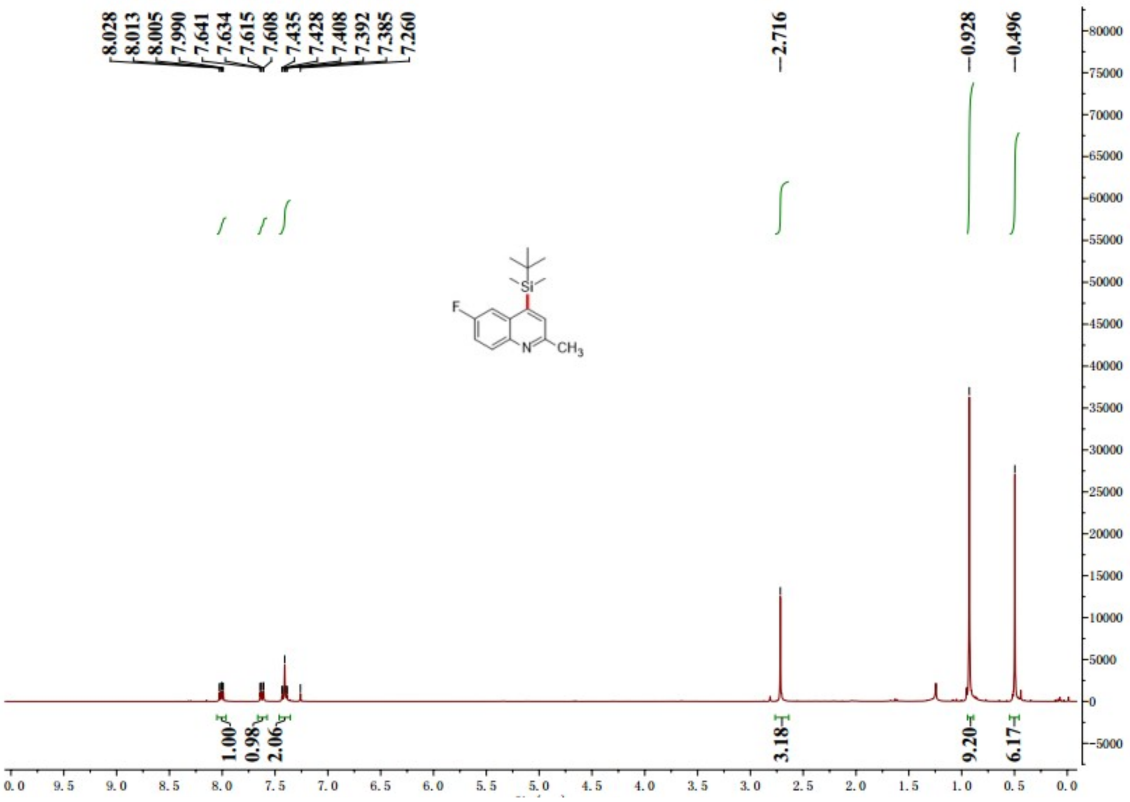
8.028 8.013 8.005 7.990 7.641 7.634 7.615 7.608 7.435 7.428 7.408 7.392 7.385 7.260
2.716
0.928
0.496
F
Si
N
CH3
1.00 0.98 2.06 3.18 9.20 6.17

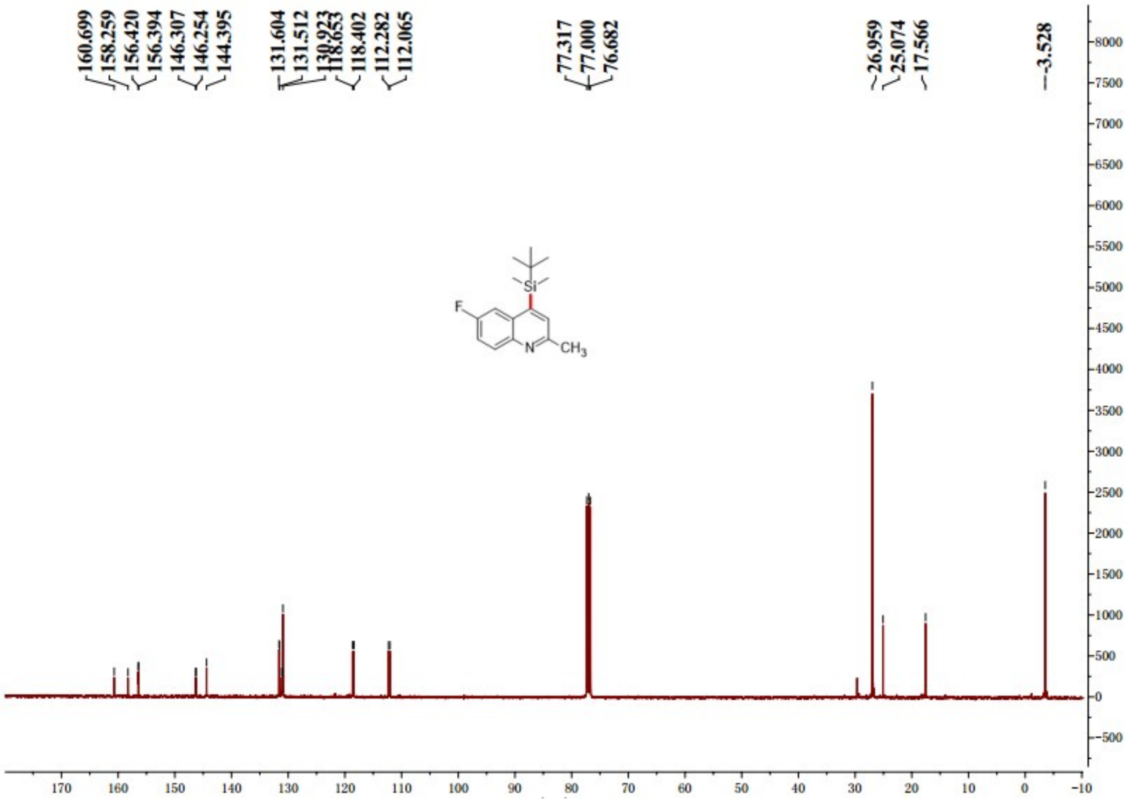
160.699 158.259 156.420 156.394 146.307 146.254 144.395
131.604 131.512 130.923 118.653 118.402 112.282 112.065
77.317 77.000 76.682
26.959 25.074 17.566
-3.528
F
Si
N
CH3

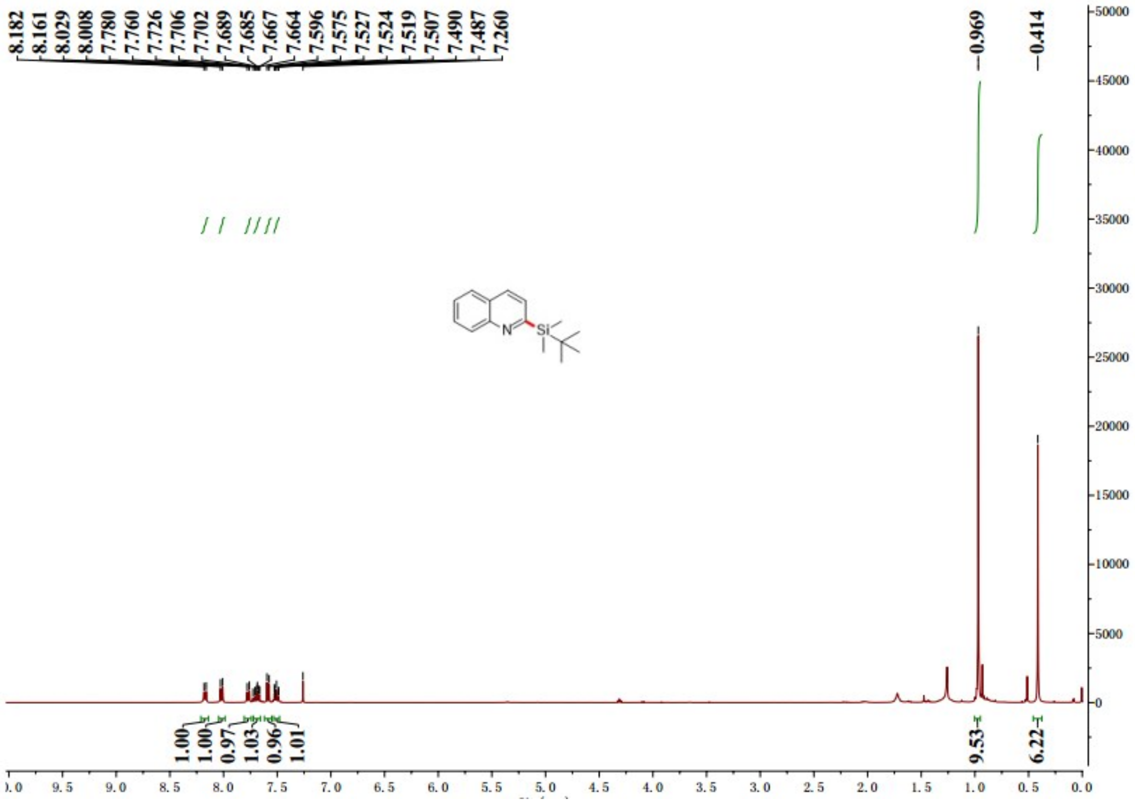
8.182
8.161
8.029
8.008
7.780
7.760
7.726
7.706
7.702
7.689
7.685
7.667
7.664
7.596
7.575
7.527
7.524
7.519
7.507
7.490
7.487
7.260
0.969
0.414
1.00
1.00
0.97
1.03
0.96
1.01
9.53
6.22
Si
N

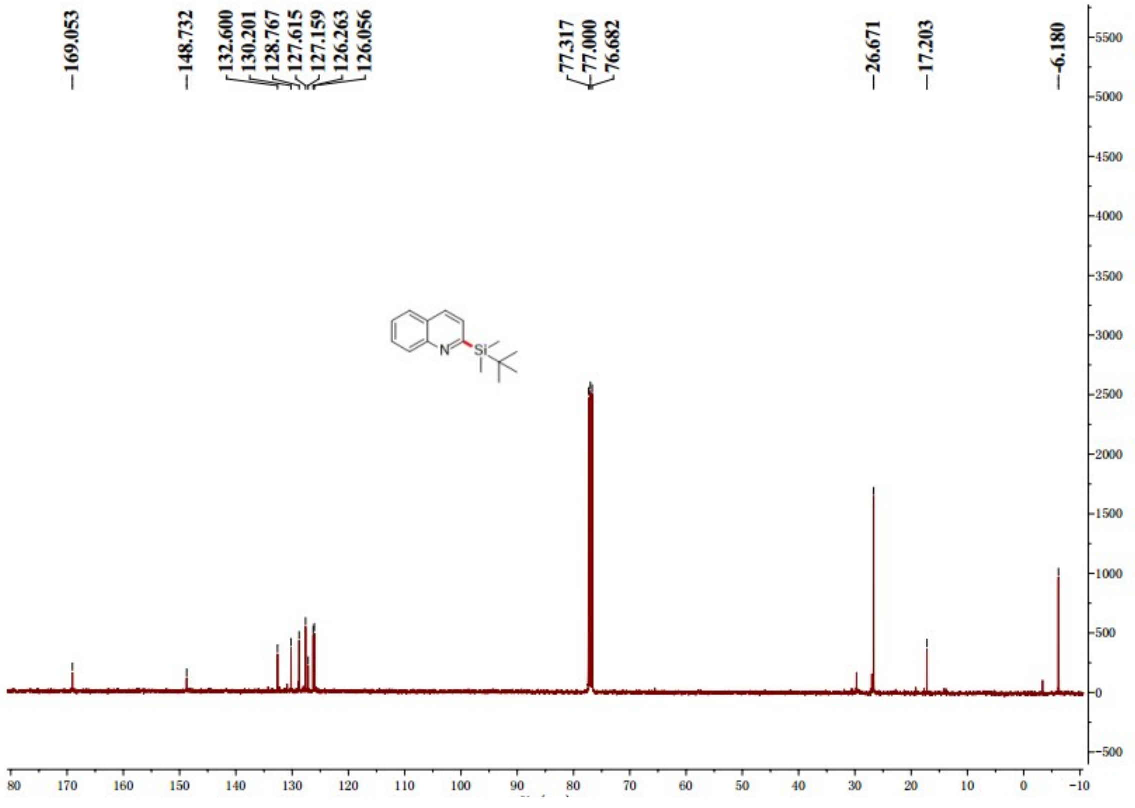
169.053
148.732
132.600
130.201
128.767
127.615
127.159
126.263
126.056
77.317
77.000
76.682
26.671
17.203
-6.180
Si
N

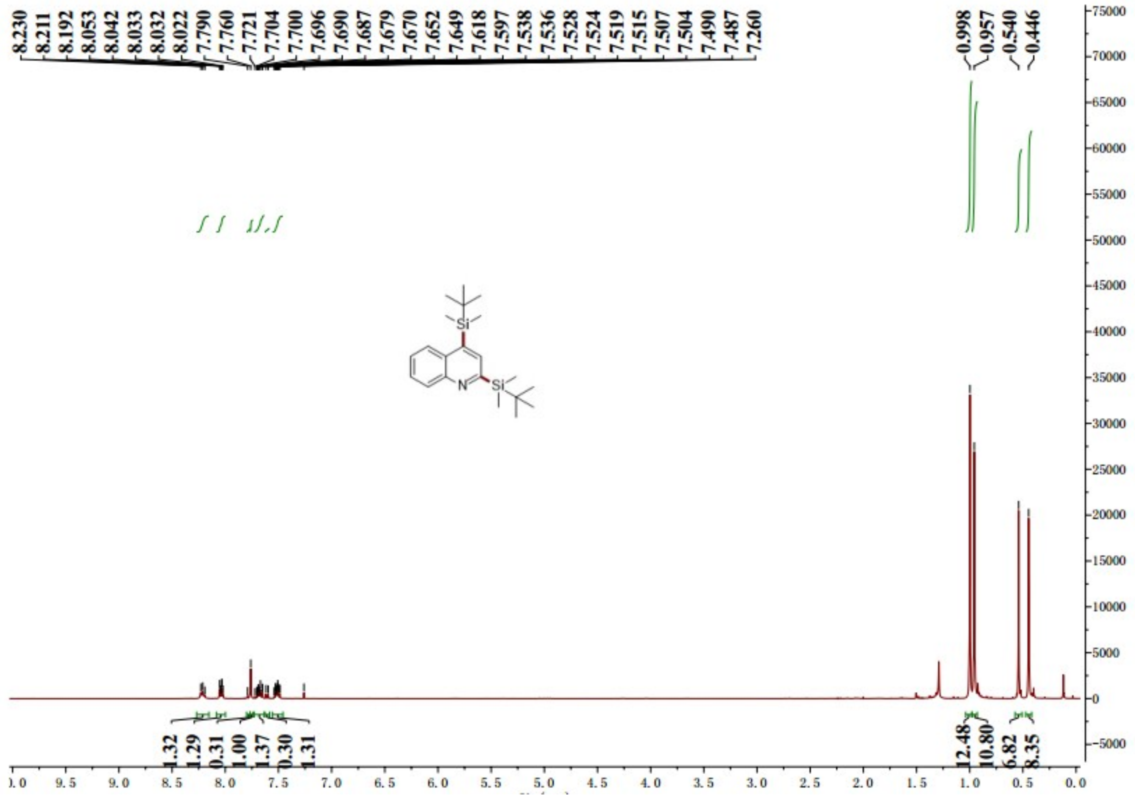

8.230 8.211 8.192 8.053 8.042 8.033 8.032 8.022 7.790 7.760 7.721 7.704 7.700 7.696 7.690 7.687 7.679 7.670 7.652 7.649 7.618 7.597 7.538 7.536 7.528 7.524 7.519 7.515 7.507 7.504 7.490 7.487 7.260
0.998 0.957 0.540 0.446
Si
N
Si
1.32 1.29 0.31 1.00 1.37 0.30 1.31
12.48 10.80 6.82 8.35

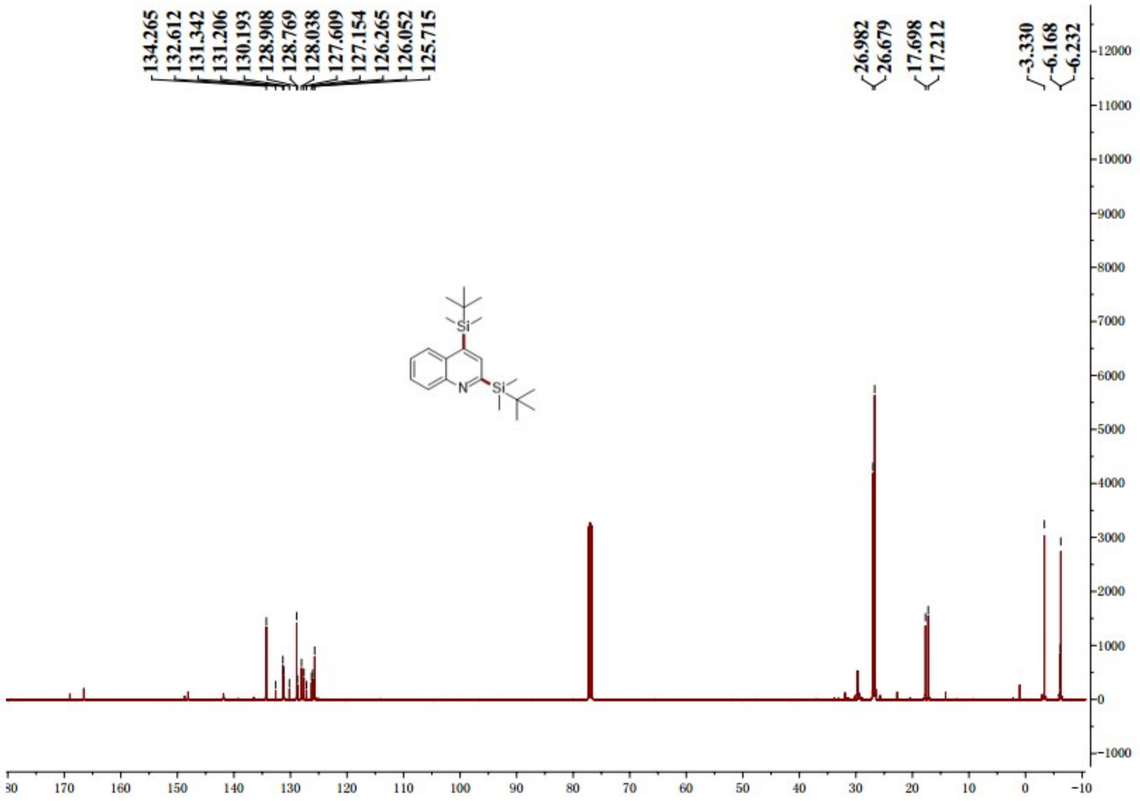

134.265 132.612 131.342 131.206 130.193 128.908 128.769 128.038 127.609 127.154 126.265 126.052 125.715
26.982 26.679 17.698 17.212
-3.330 -6.168 -6.232
Si
N
Si

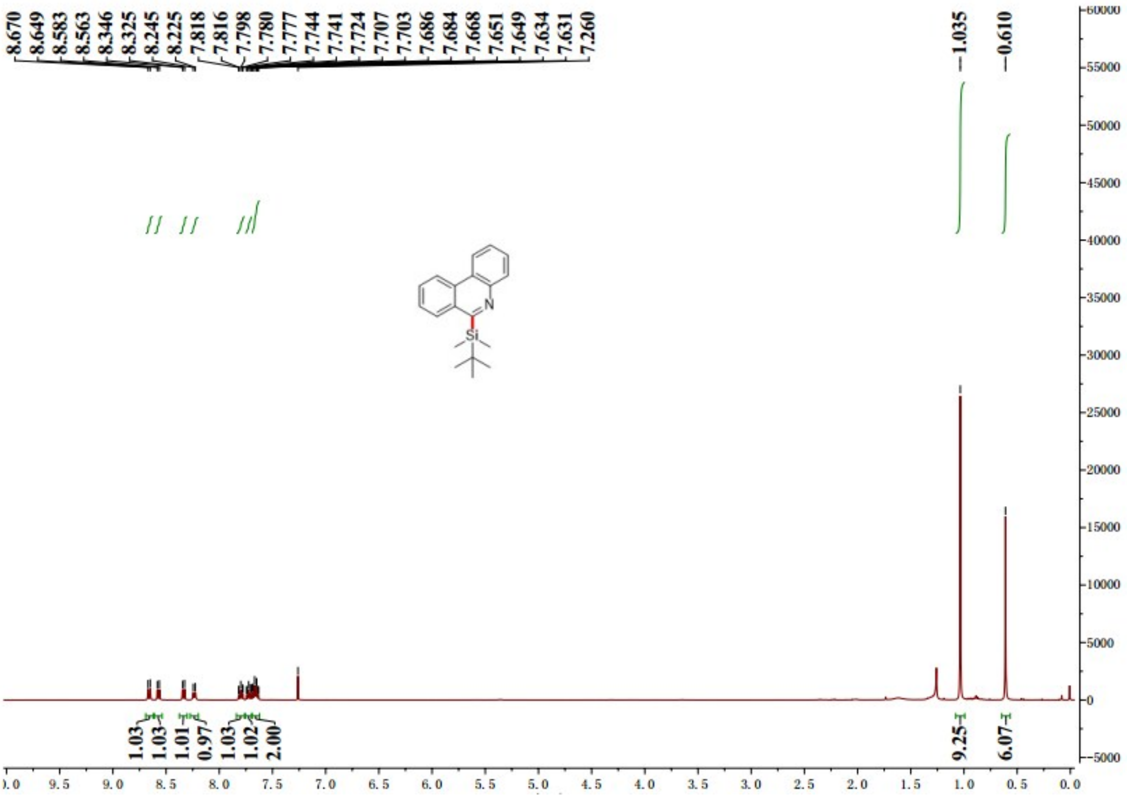
8.670
8.649
8.583
8.563
8.346
8.325
8.245
8.225
7.818
7.816
7.798
7.780
7.777
7.744
7.741
7.724
7.707
7.703
7.686
7.684
7.668
7.651
7.649
7.634
7.631
7.260
1.035
0.610
1.03
1.03
1.01
0.97
1.03
1.02
2.00
9.25
6.07
Si
N

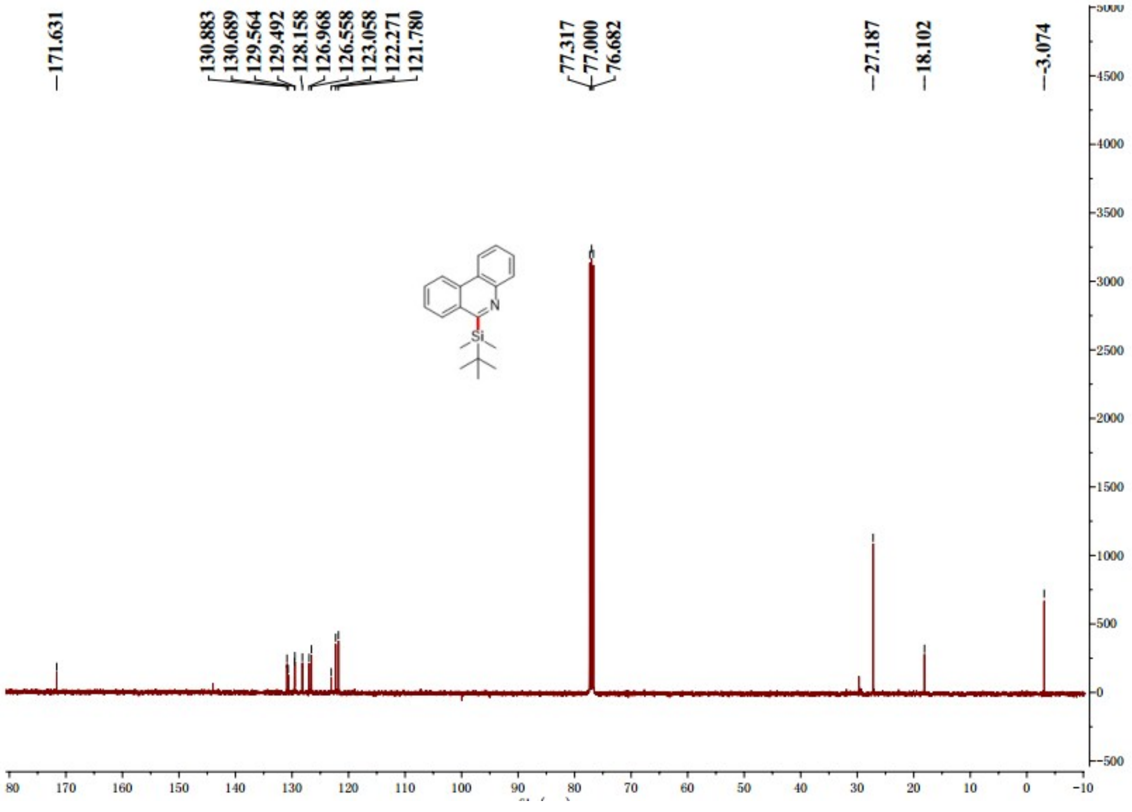
171.631
130.883
130.689
129.564
129.492
128.158
126.968
126.558
123.058
122.271
121.780
77.317
77.000
76.682
27.187
18.102
-3.074
Si
N

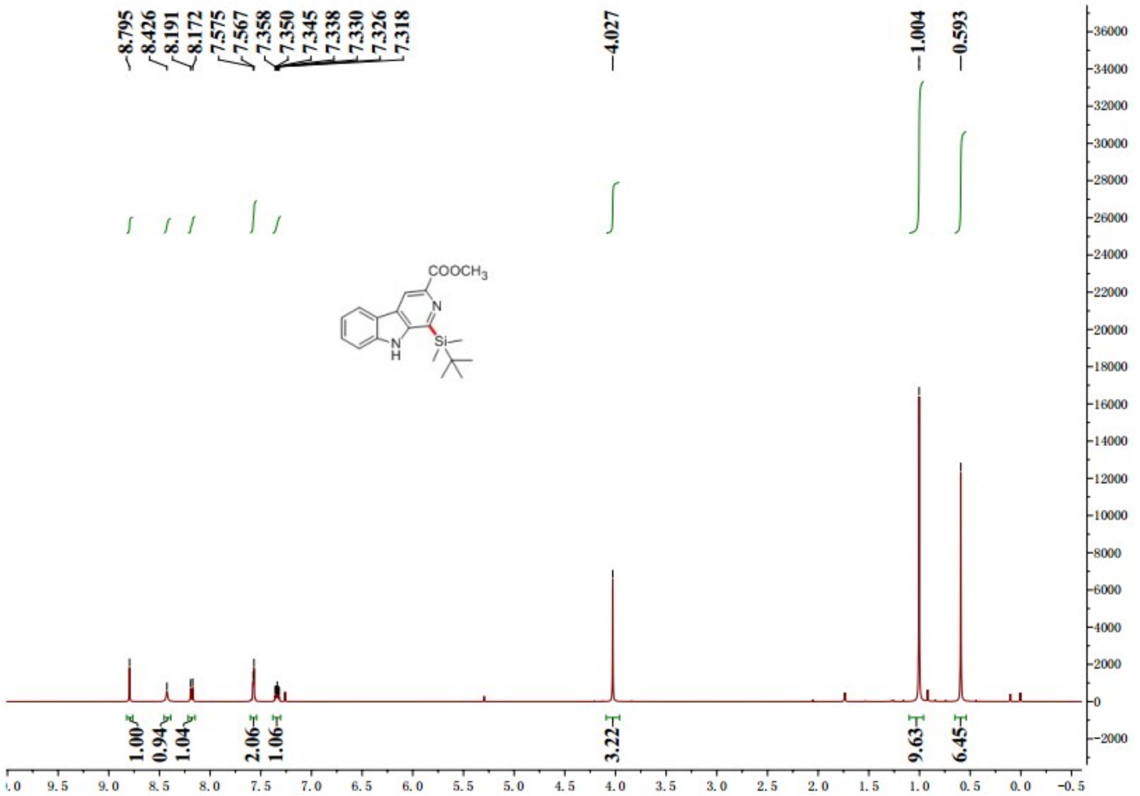
8.795
8.426
8.191
8.172
7.575
7.567
7.358
7.350
7.345
7.338
7.330
7.326
7.318
4.027
1.004
0.593
COOCH3
N
Si
H
1.00
0.94
1.04
2.06
1.06
3.22
9.63
6.45

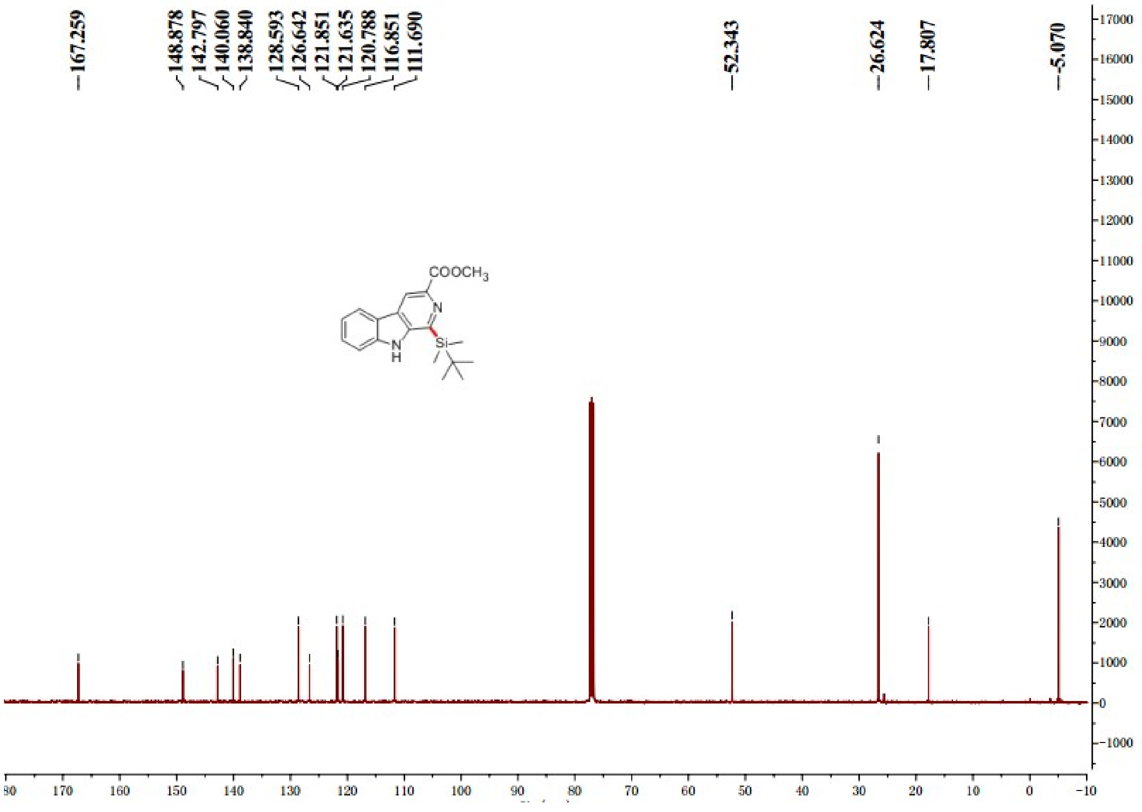
167.259
148.878
142.797
140.060
138.840
128.593
126.642
121.851
121.635
120.788
116.851
111.690
52.343
26.624
17.807
-5.070
COOCH3
N
Si
H

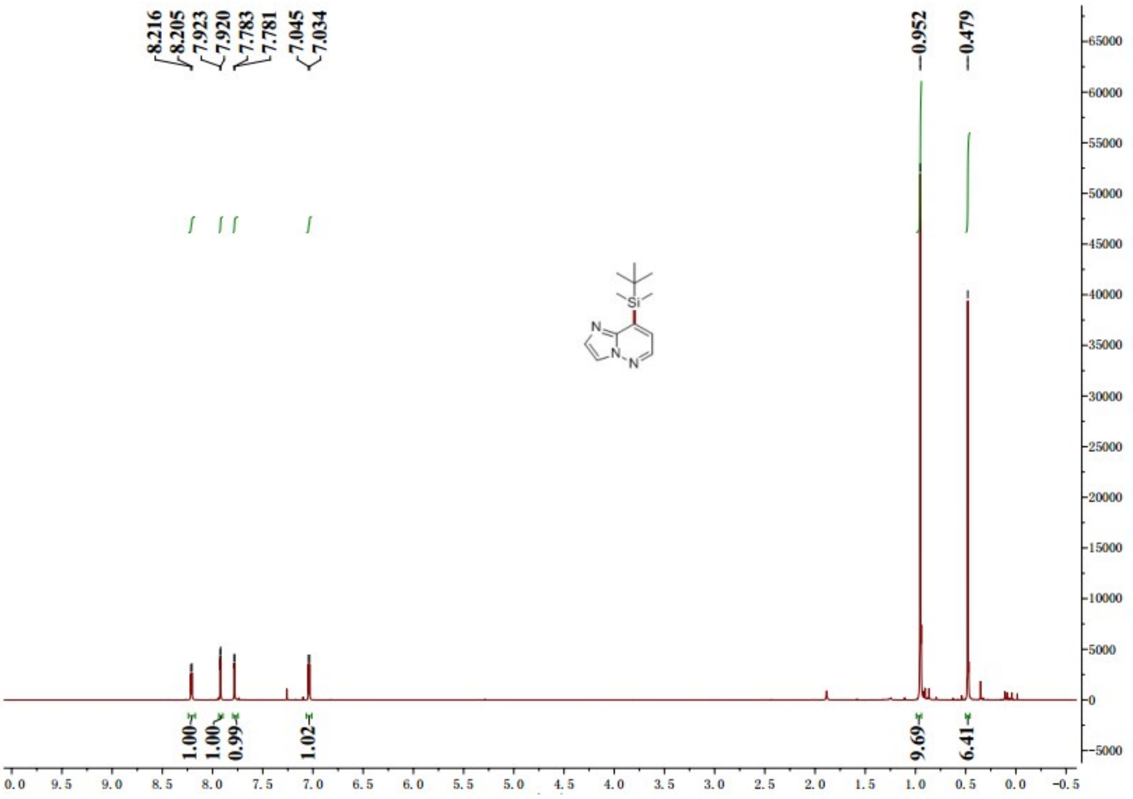
8.216
8.205
7.923
7.920
7.783
7.781
7.045
7.034
0.952
0.479
1.00
1.00
0.99
1.02
9.69
6.41
Si
N
N
N

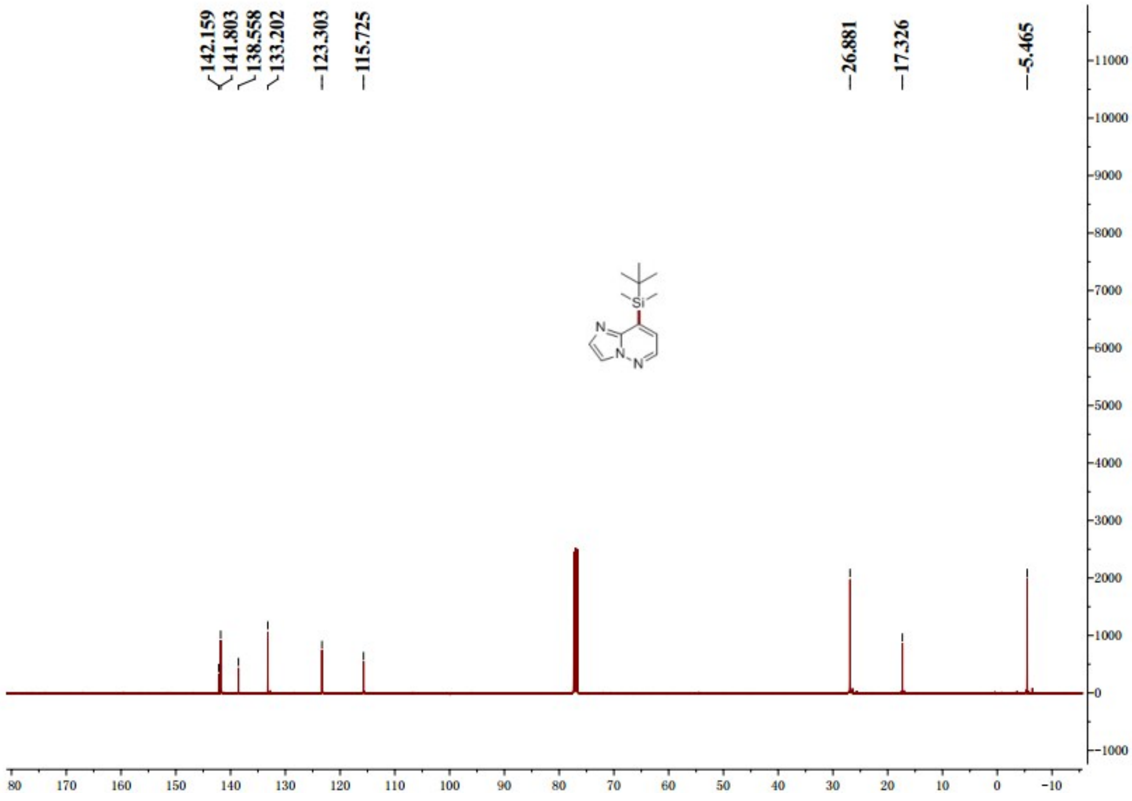
142.159
141.803
138.558
133.202
123.303
115.725
26.881
17.326
-5.465
Si
N
N
N

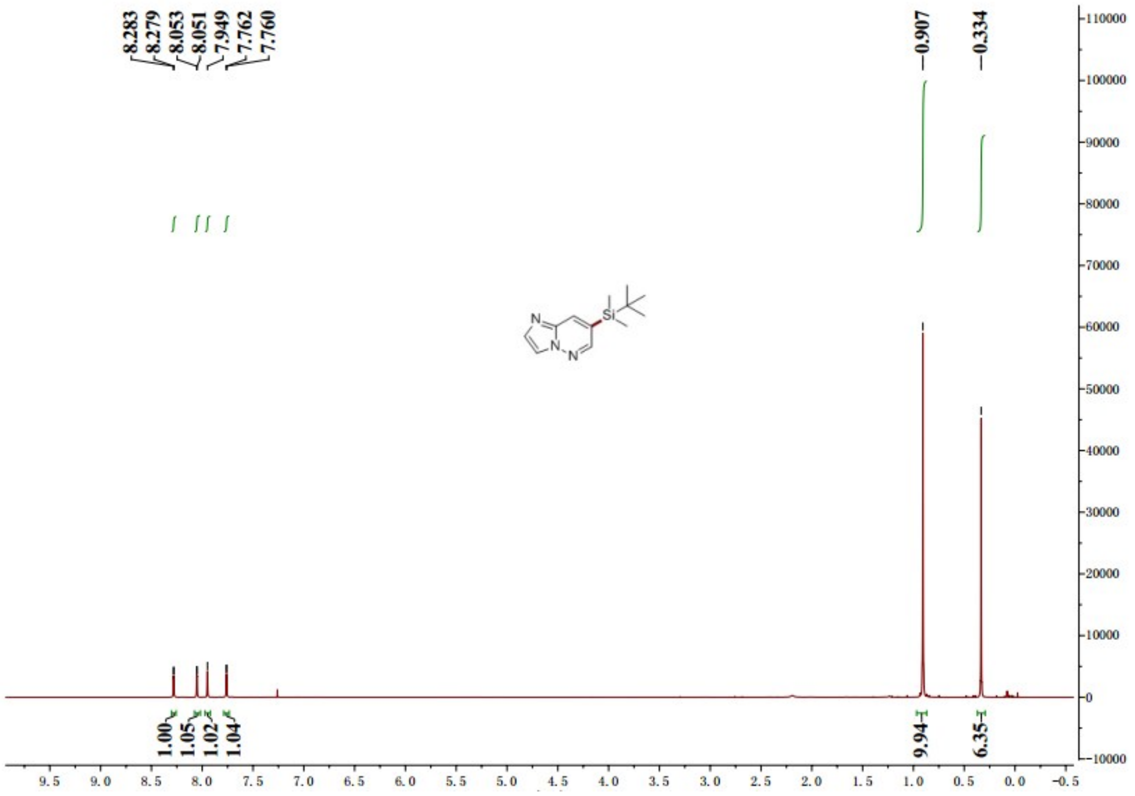

8.283
8.279
8.053
8.051
7.949
7.762
7.760
0.907
0.334
1.00
1.05
1.02
1.04
9.94
6.35

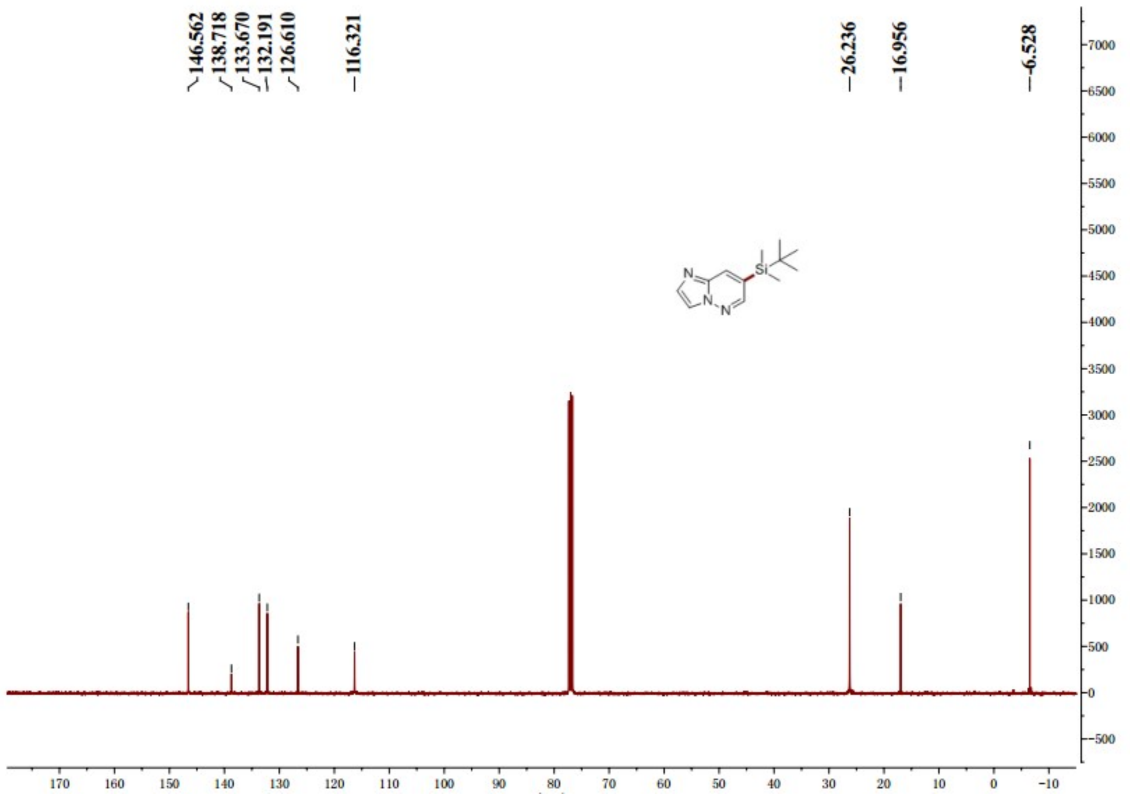

146.562
138.718
133.670
132.191
126.610
116.321
26.236
16.956
-6.528

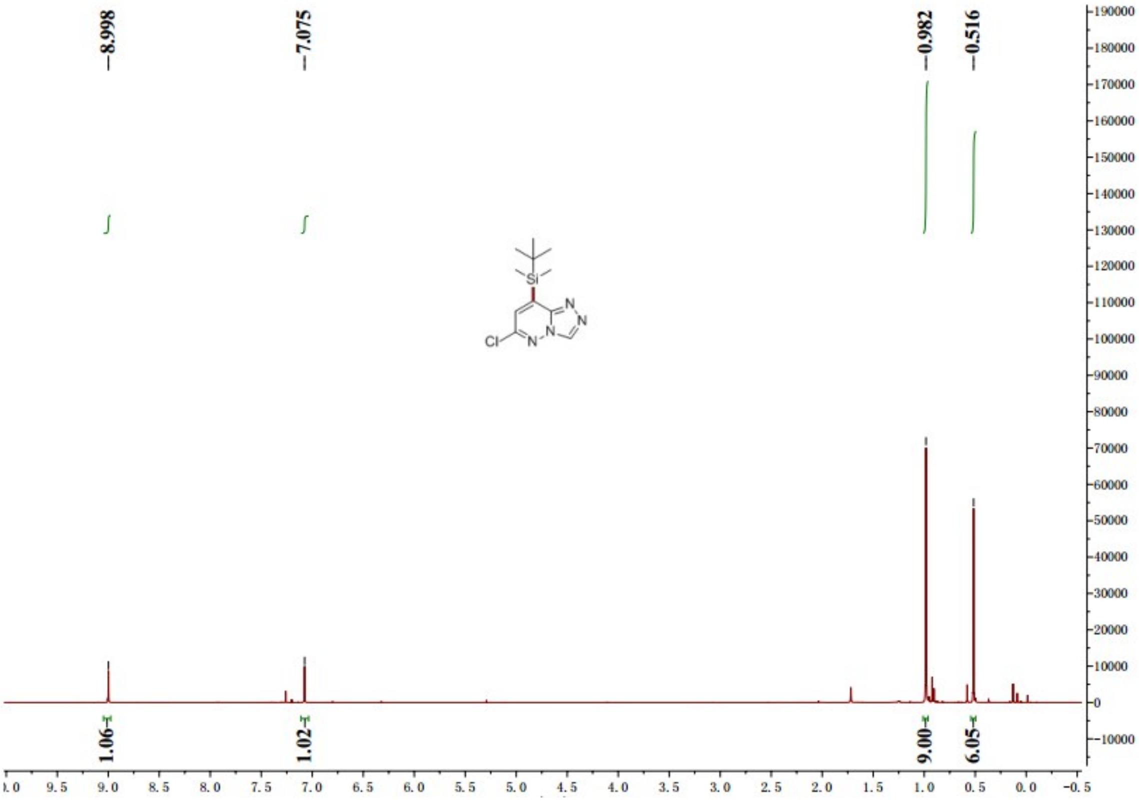
8.998
7.075
0.982
0.516
1.06
1.02
9.00
6.05
Si
N
N
N
N
Cl

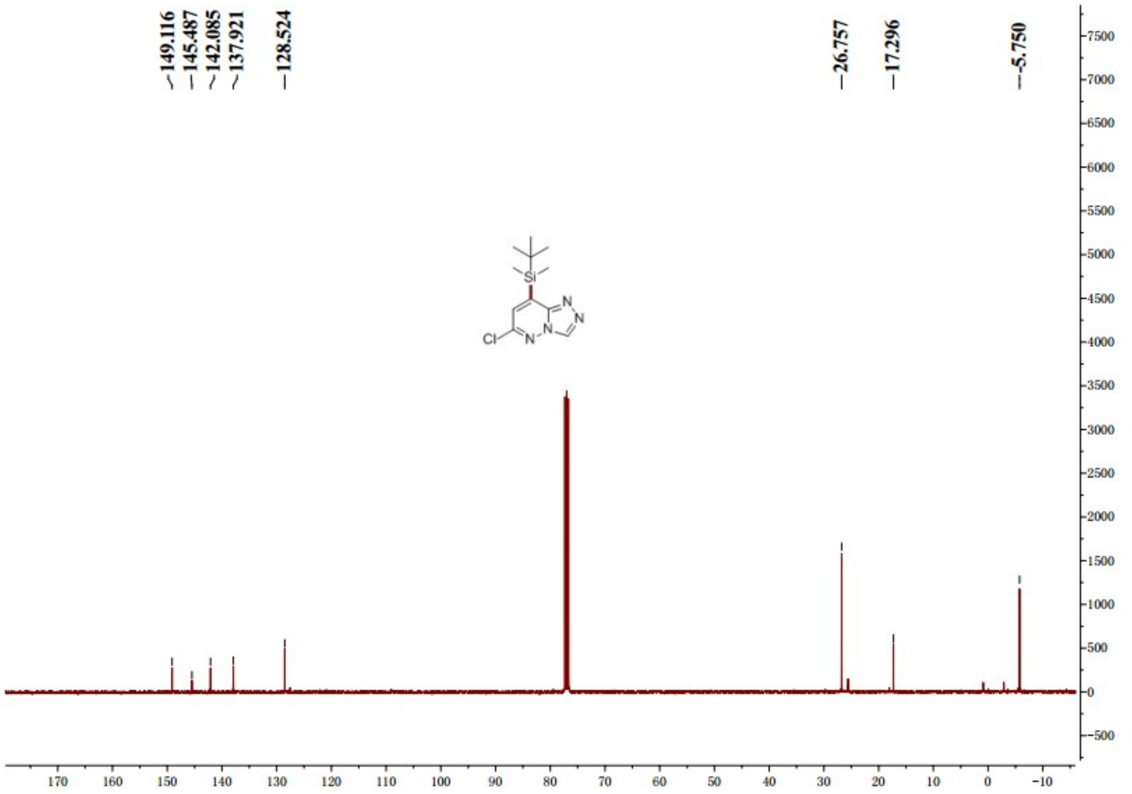
149.116
145.487
142.085
137.921
128.524
26.757
17.296
-5.750
Si
N
N
N
N
Cl

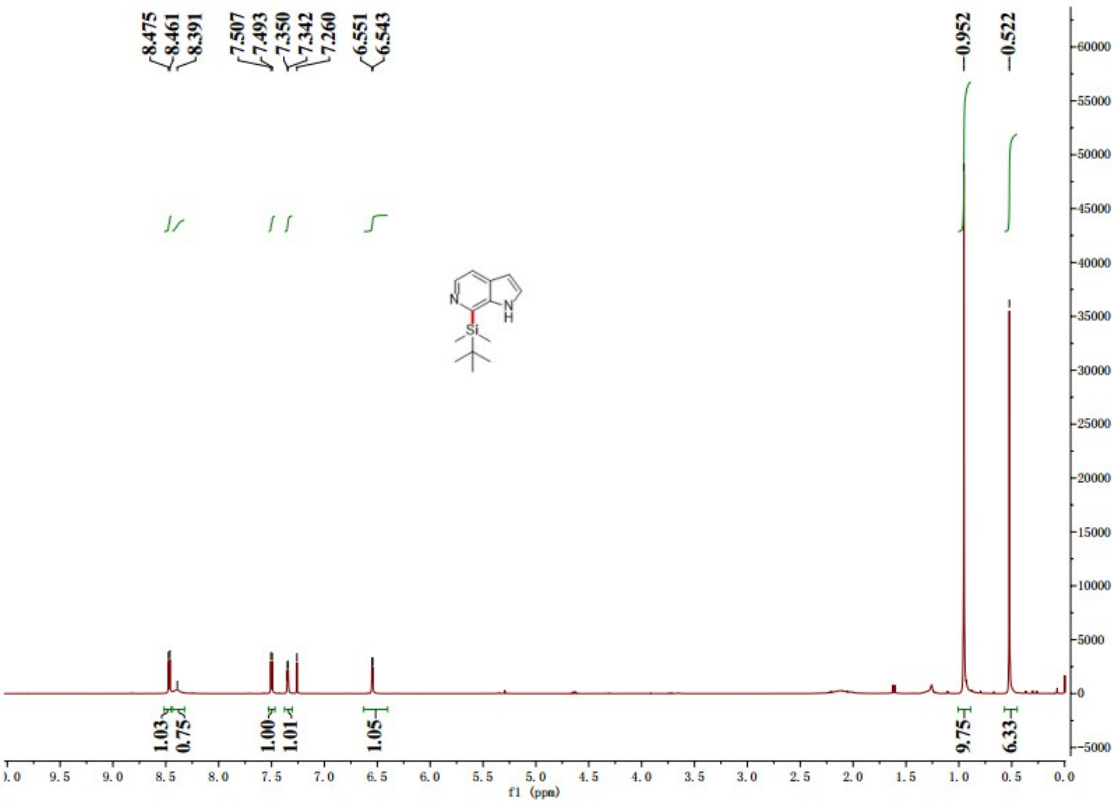
8.475
8.461
8.391
7.507
7.493
7.350
7.342
7.260
6.551
6.543
0.952
0.522
1.03
0.75
1.00
1.01
1.05
9.75
6.33
N
Si
H
f1 (ppm)

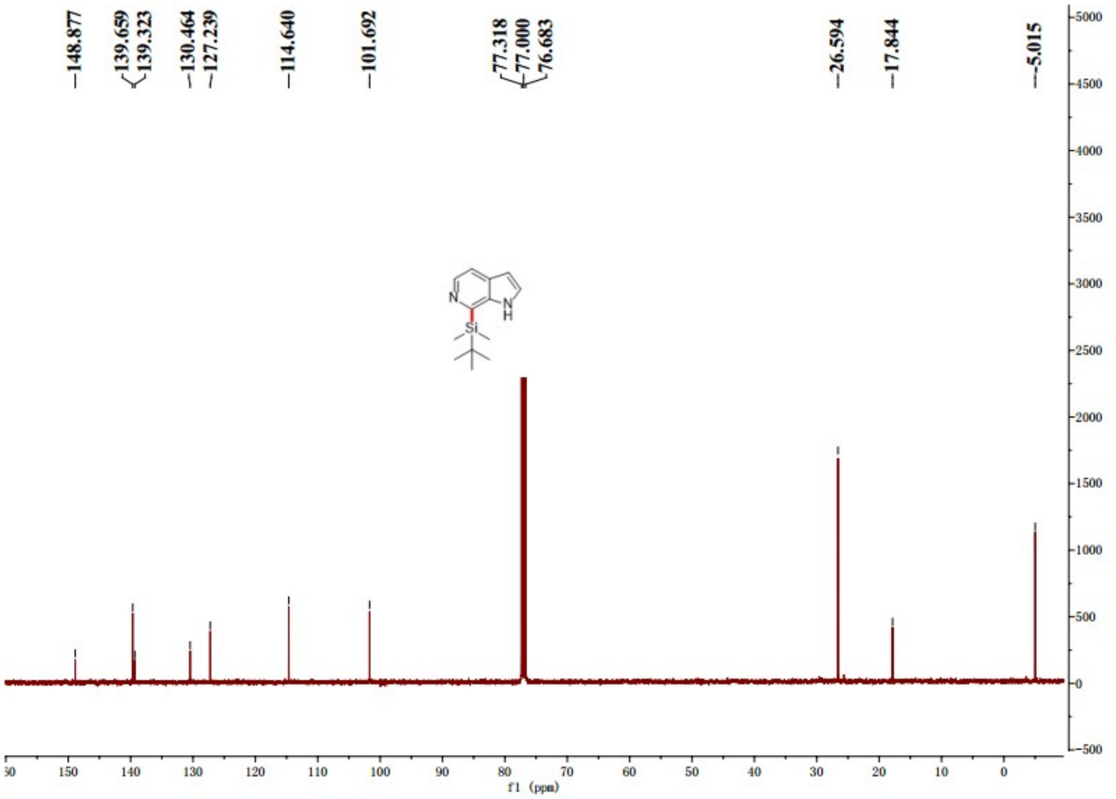
148.877
139.659
139.323
130.464
127.239
114.640
101.692
77.318
77.000
76.683
26.594
17.844
-5.015
N
Si
H
f1 (ppm)

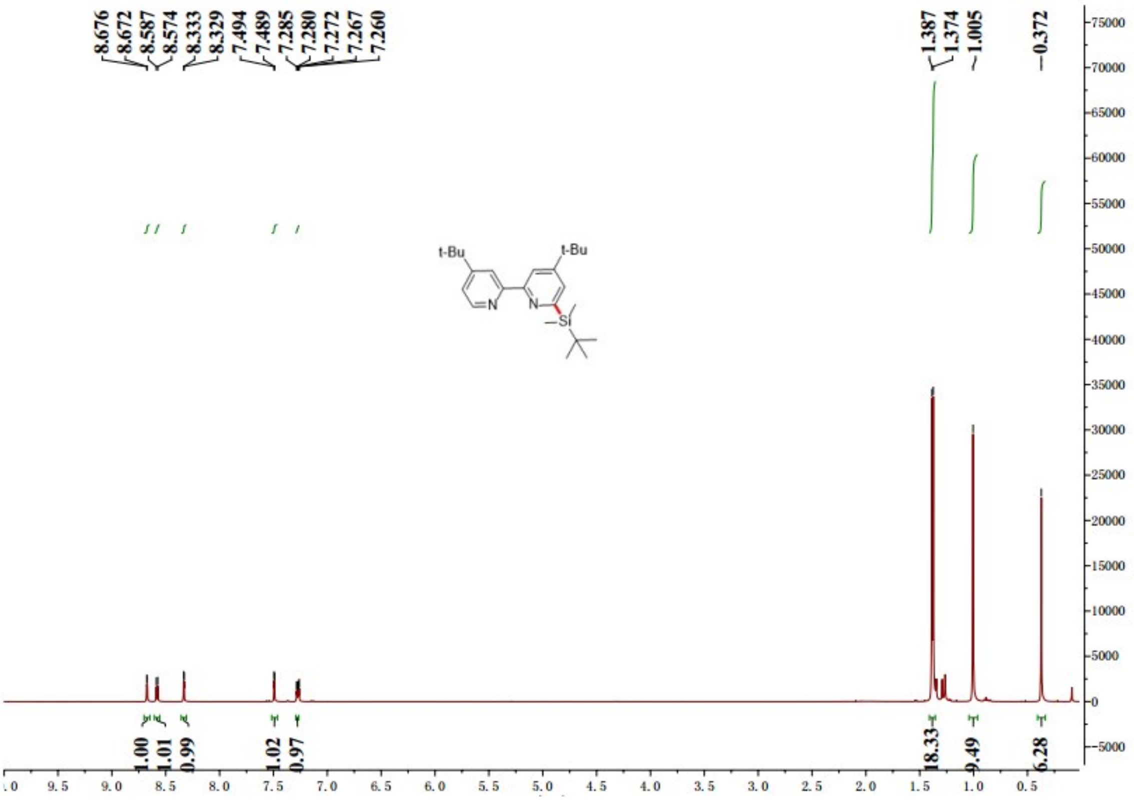
8.676
8.672
8.587
8.574
8.333
8.329
7.494
7.489
7.285
7.280
7.272
7.267
7.260
1.387
1.374
1.005
0.372
t-Bu
t-Bu
N
N
Si
1.00
1.01
0.99
1.02
0.97
18.33
9.49
6.28

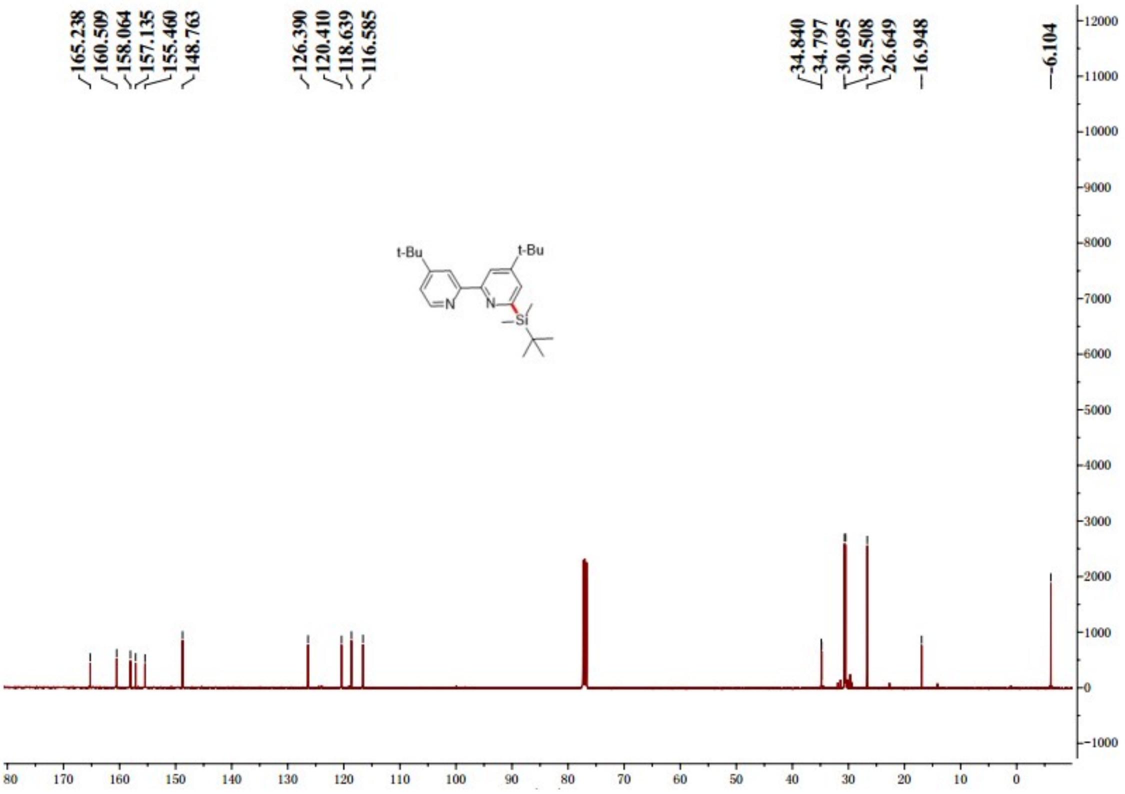
165.238
160.509
158.064
157.135
155.460
148.763
126.390
120.410
118.639
116.585
34.840
34.797
30.695
30.508
26.649
16.948
-6.104
t-Bu
t-Bu
N
N
Si

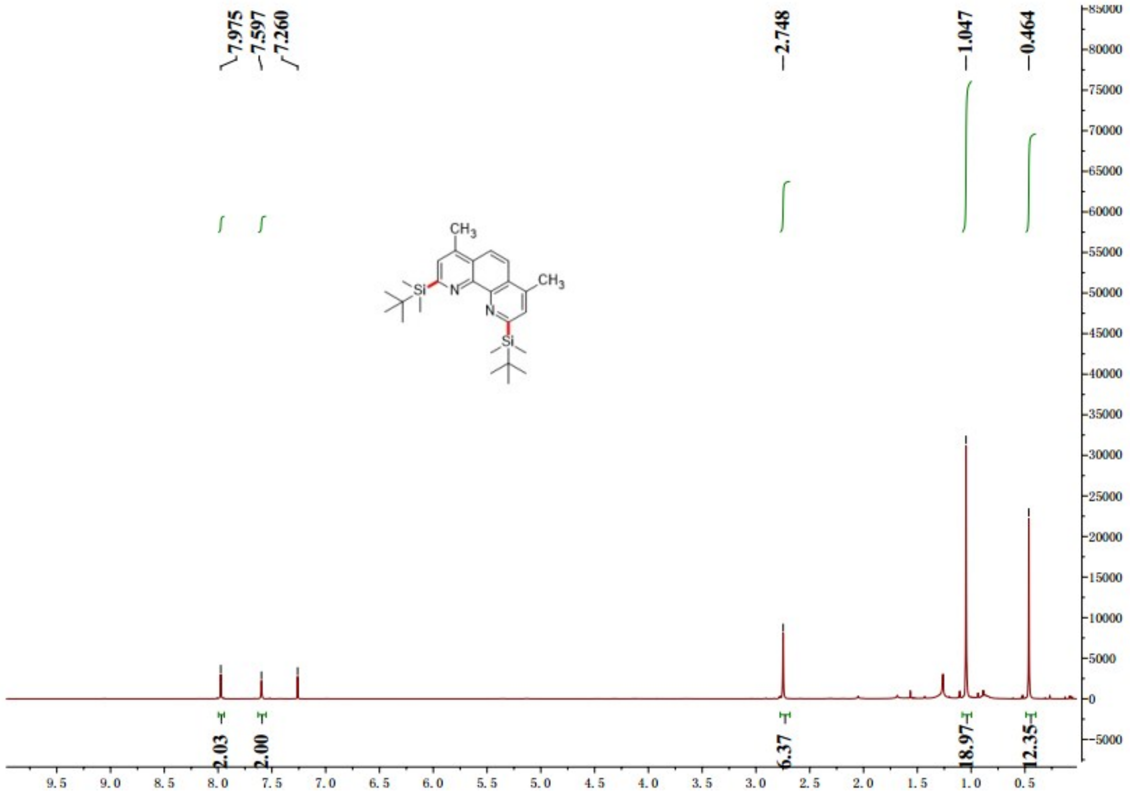
7.975
7.597
7.260
2.748
1.047
0.464
CH3
Si
N
CH3
N
Si
2.03
2.00
6.37
18.97
12.35

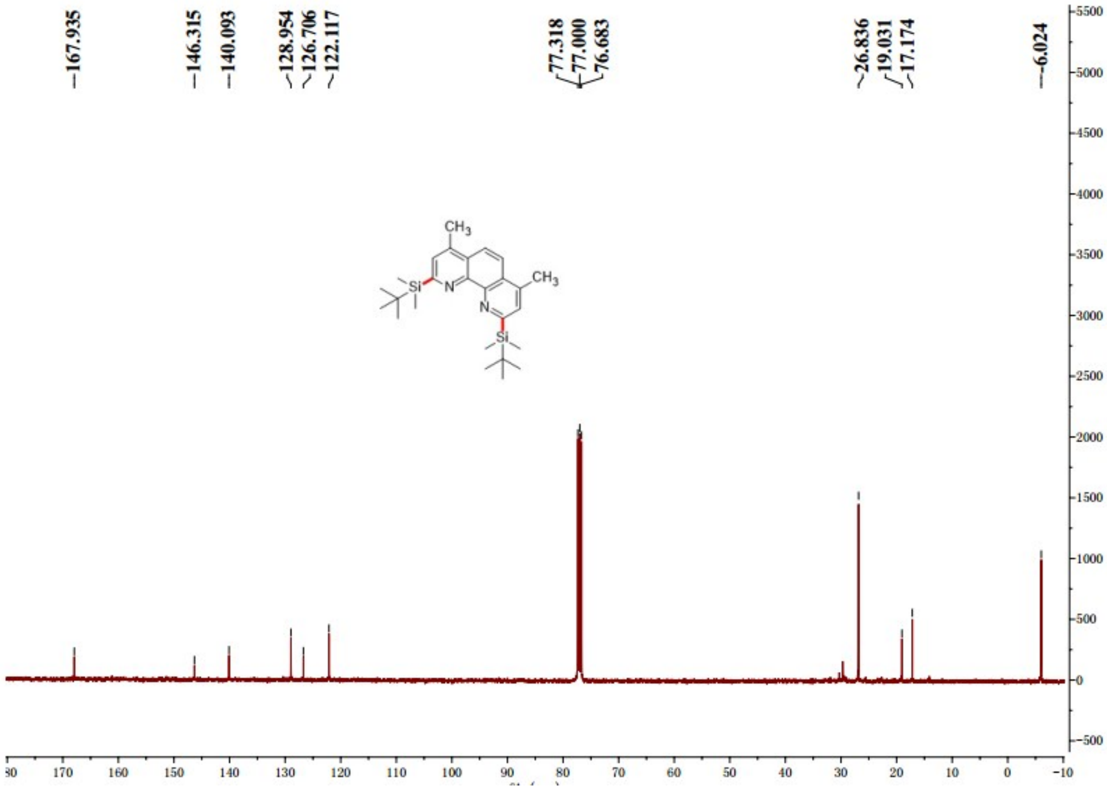
167.935
146.315
140.093
128.954
126.706
122.117
77.318
77.000
76.683
26.836
19.031
17.174
-6.024
CH3
Si
N
CH3
N
Si

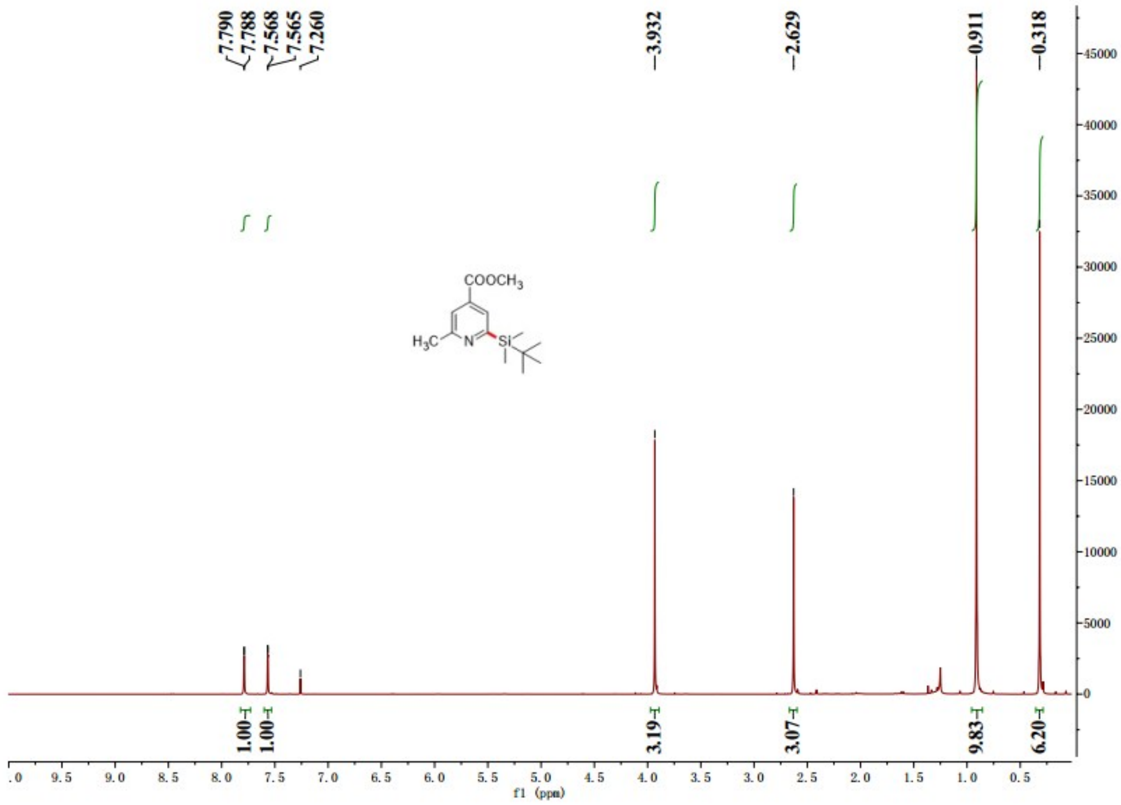
7.790
7.788
7.568
7.565
7.260
3.932
2.629
0.911
0.318
COOCH3
H3C
N
Si
1.00
1.00
3.19
3.07
9.83
6.20
f1 (ppm)

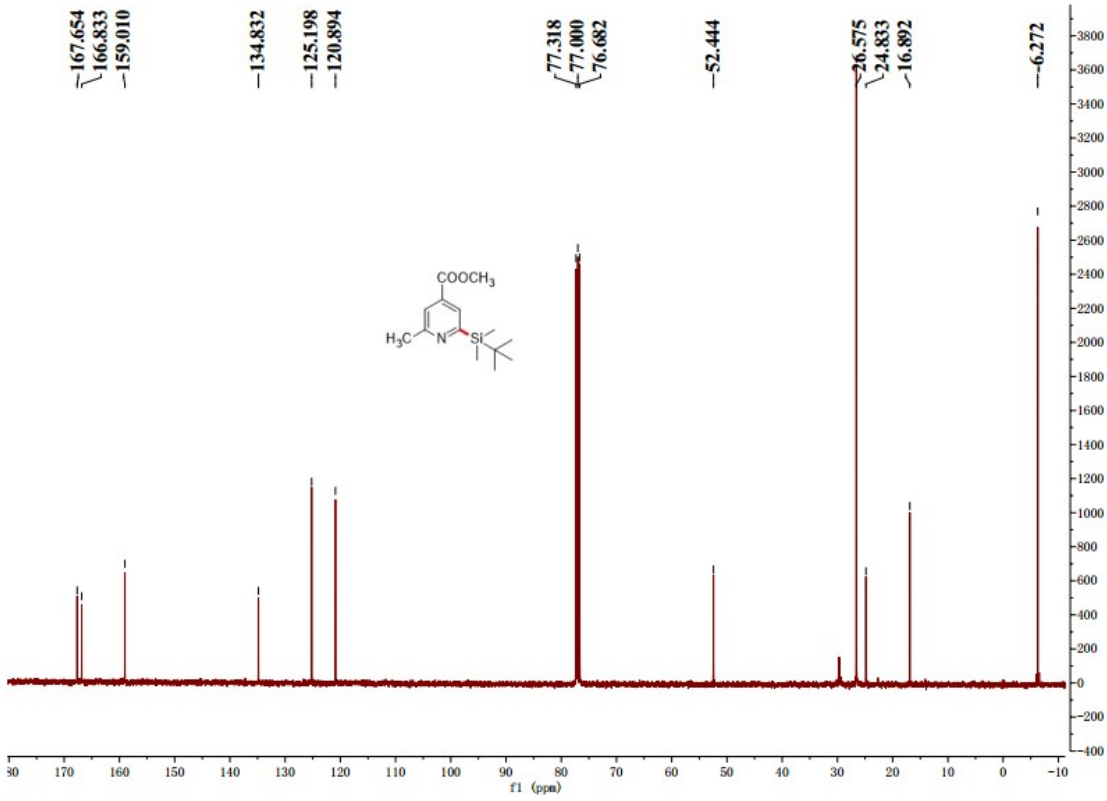
167.654
166.833
159.010
134.832
125.198
120.894
77.318
77.000
76.682
52.444
26.575
24.833
16.892
-6.272
COOCH3
H3C
N
Si
f1 (ppm)

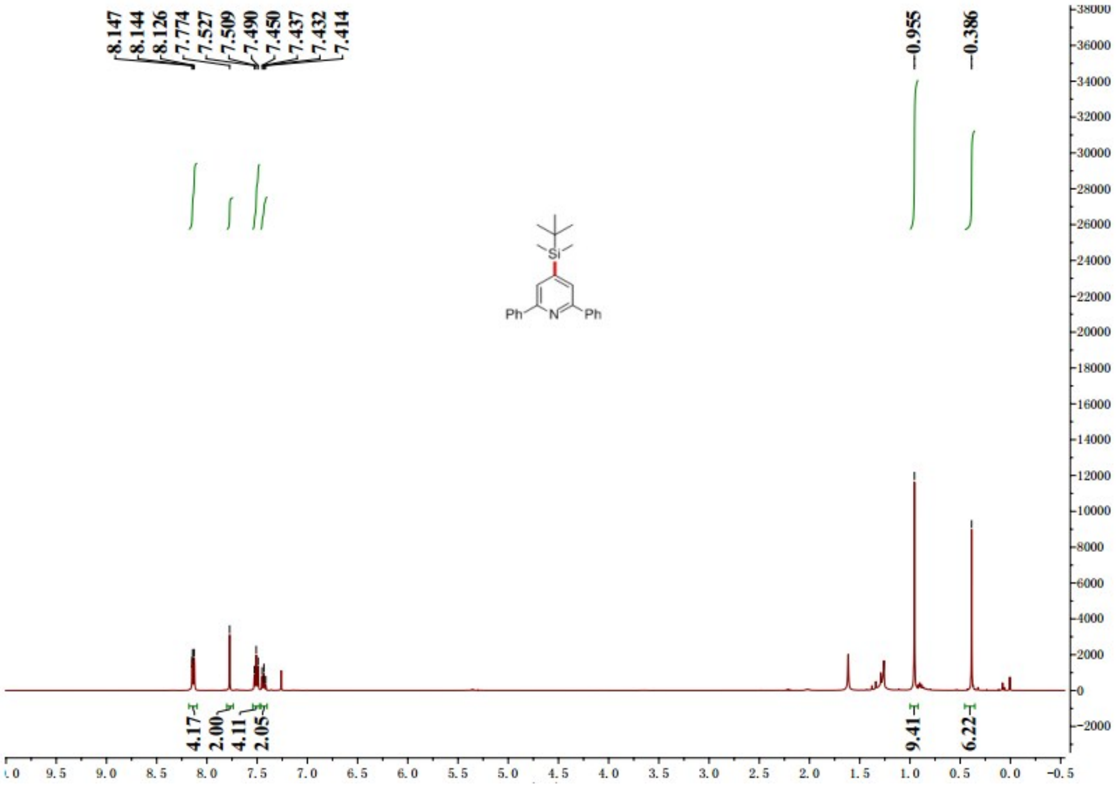
8.147
8.144
8.126
7.774
7.527
7.509
7.490
7.450
7.437
7.432
7.414
0.955
0.386
Si
Ph
N
Ph
4.17
2.00
4.11
2.05
9.41
6.22

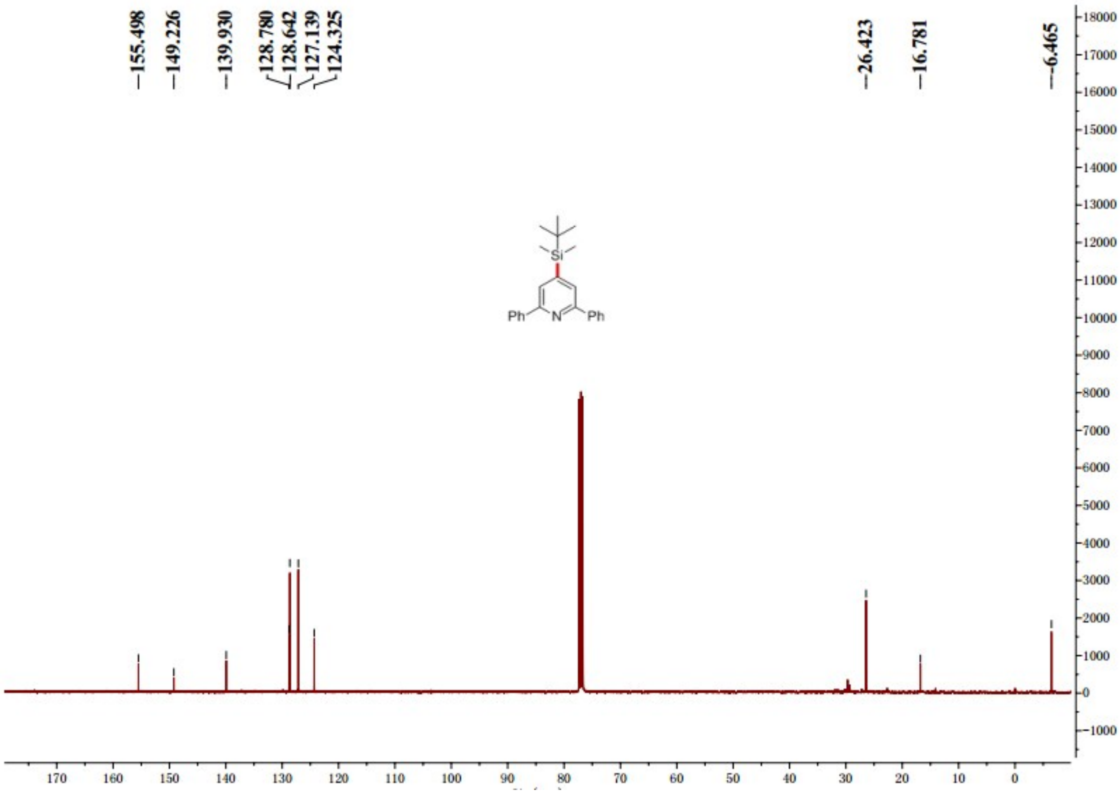
155.498
149.226
139.930
128.780
128.642
127.139
124.325
26.423
16.781
-6.465
Si
Ph
N
Ph

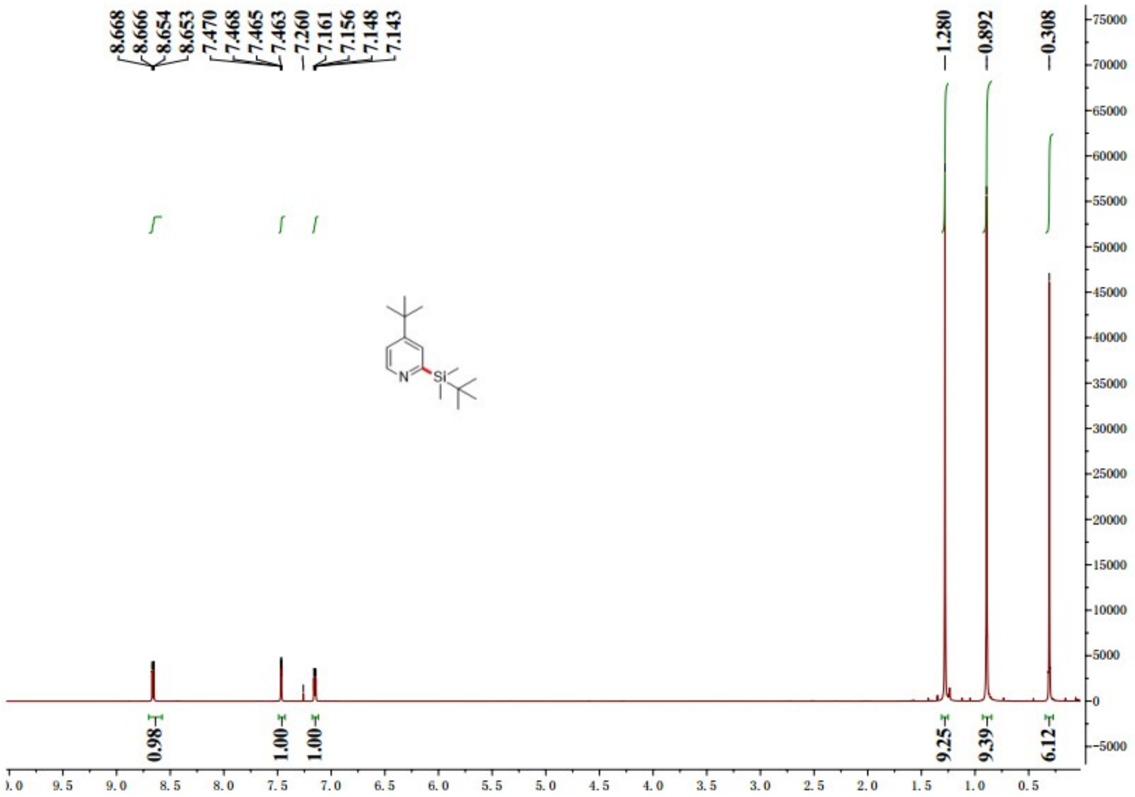
8.668
8.666
8.654
8.653
7.470
7.468
7.465
7.463
7.260
7.161
7.156
7.148
7.143
1.280
0.892
0.308
N
Si
0.98
1.00
1.00
9.25
9.39
6.12

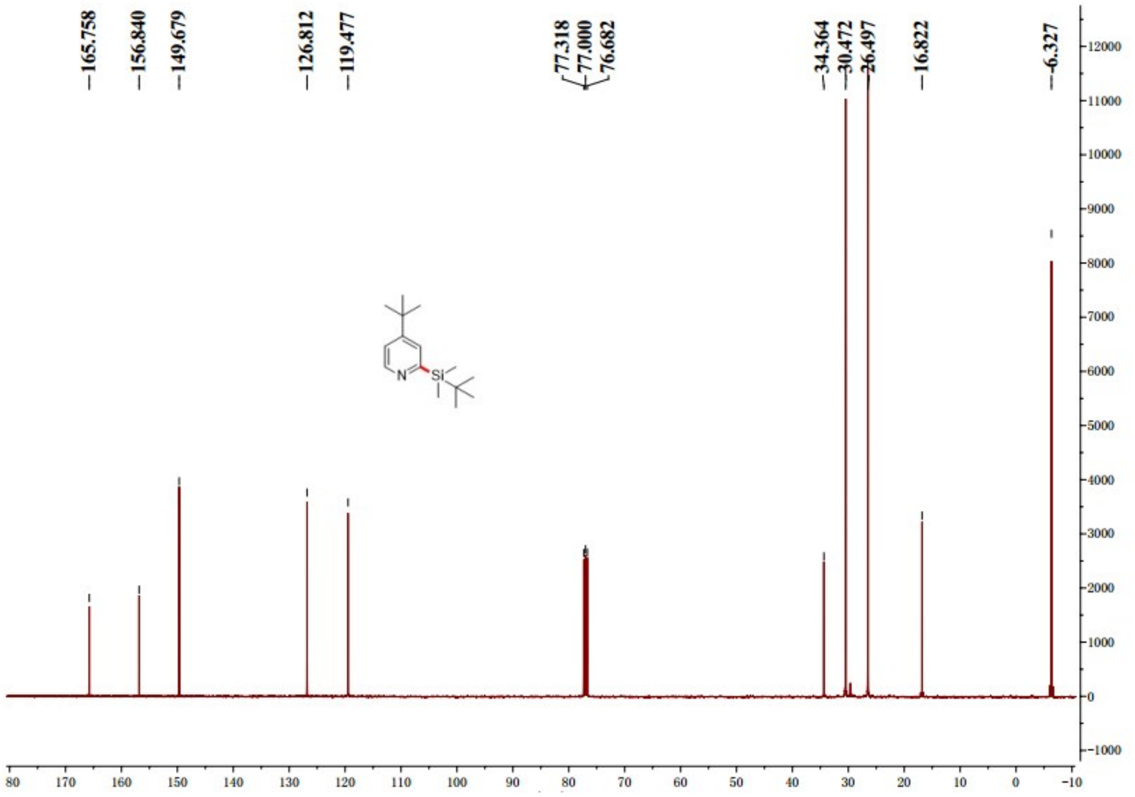
165.758
156.840
149.679
126.812
119.477
77.318
77.000
76.682
34.364
30.472
26.497
16.822
-6.327
N
Si

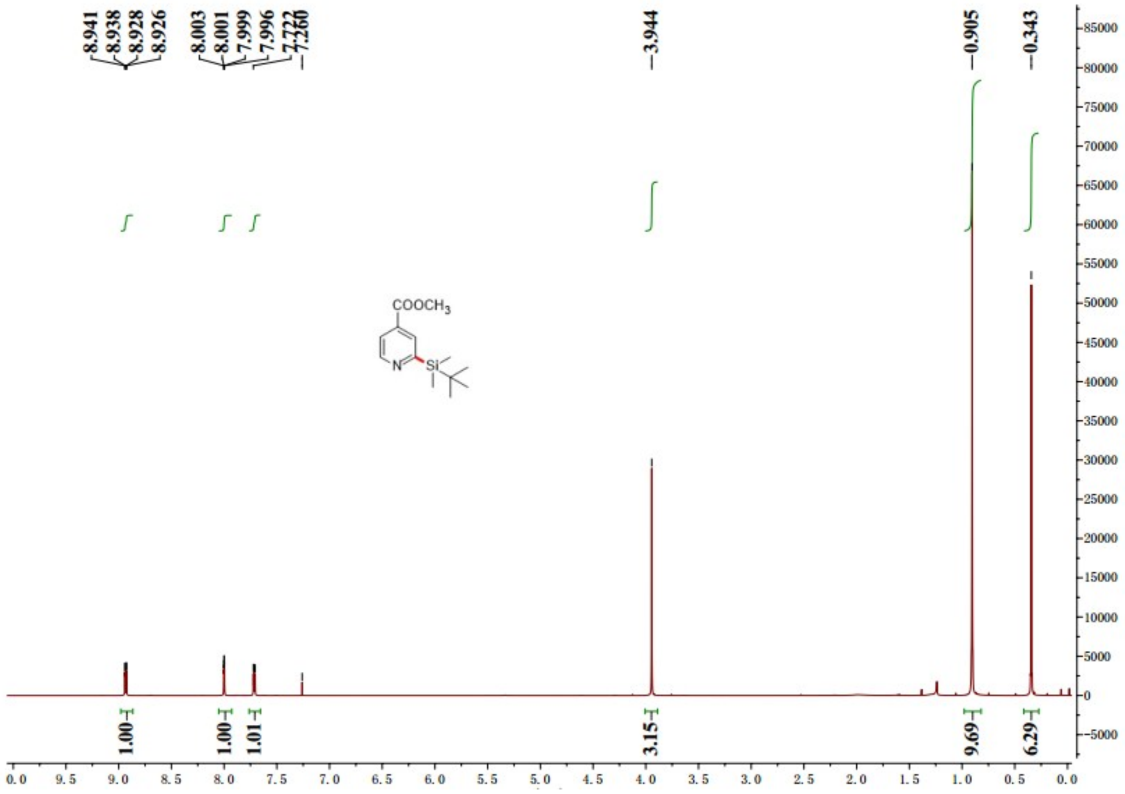
8.941
8.938
8.928
8.926
8.003
8.001
7.999
7.996
7.722
7.260
3.944
0.905
0.343
COOCH3
N
Si
1.00
1.00
1.01
3.15
9.69
6.29

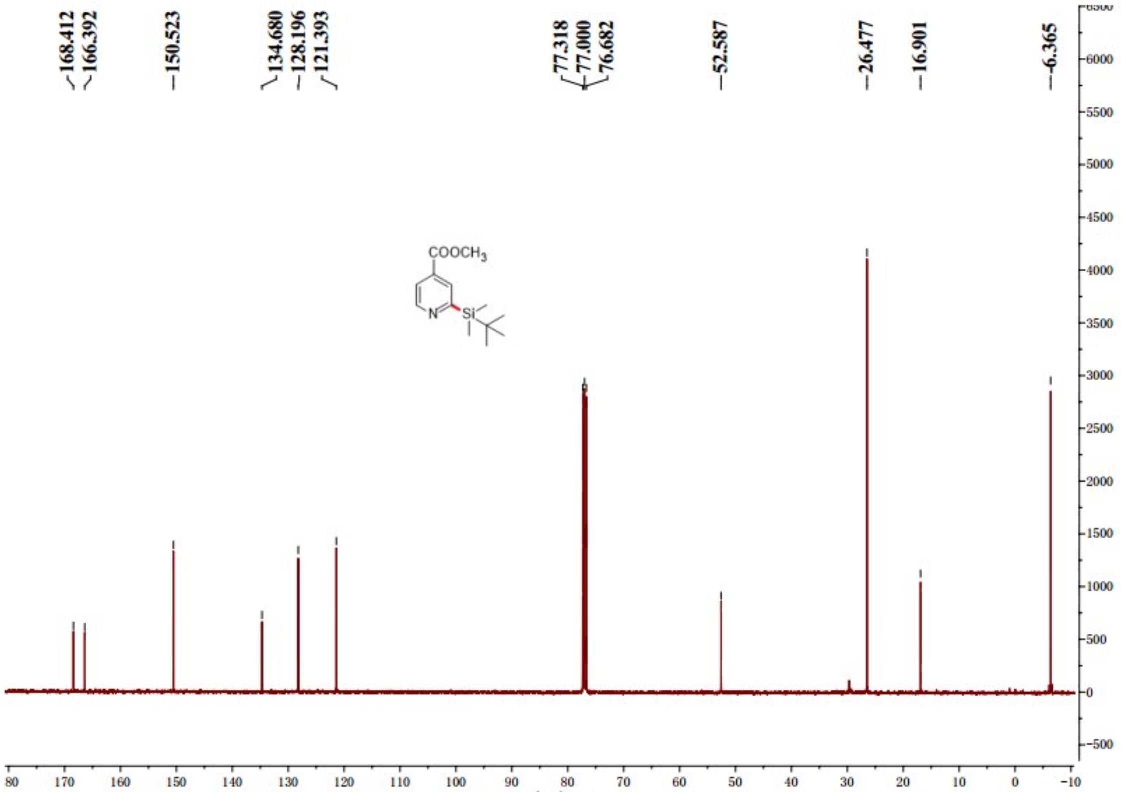
168.412
166.392
150.523
134.680
128.196
121.393
77.318
77.000
76.682
52.587
26.477
16.901
-6.365
COOCH3
N
Si

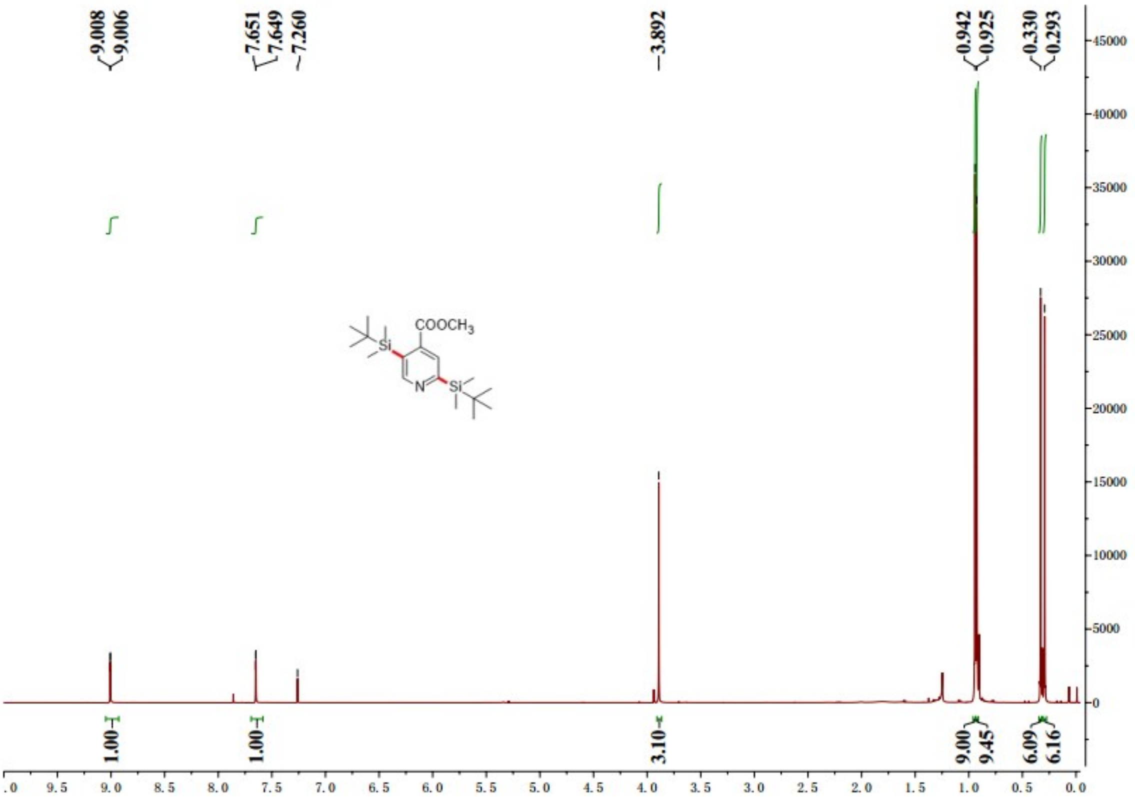
9.008
9.006
7.651
7.649
7.260
3.892
0.942
0.925
0.330
0.293
COOCH3
Si
N
Si
1.00
1.00
3.10
9.00
9.45
6.09
6.16

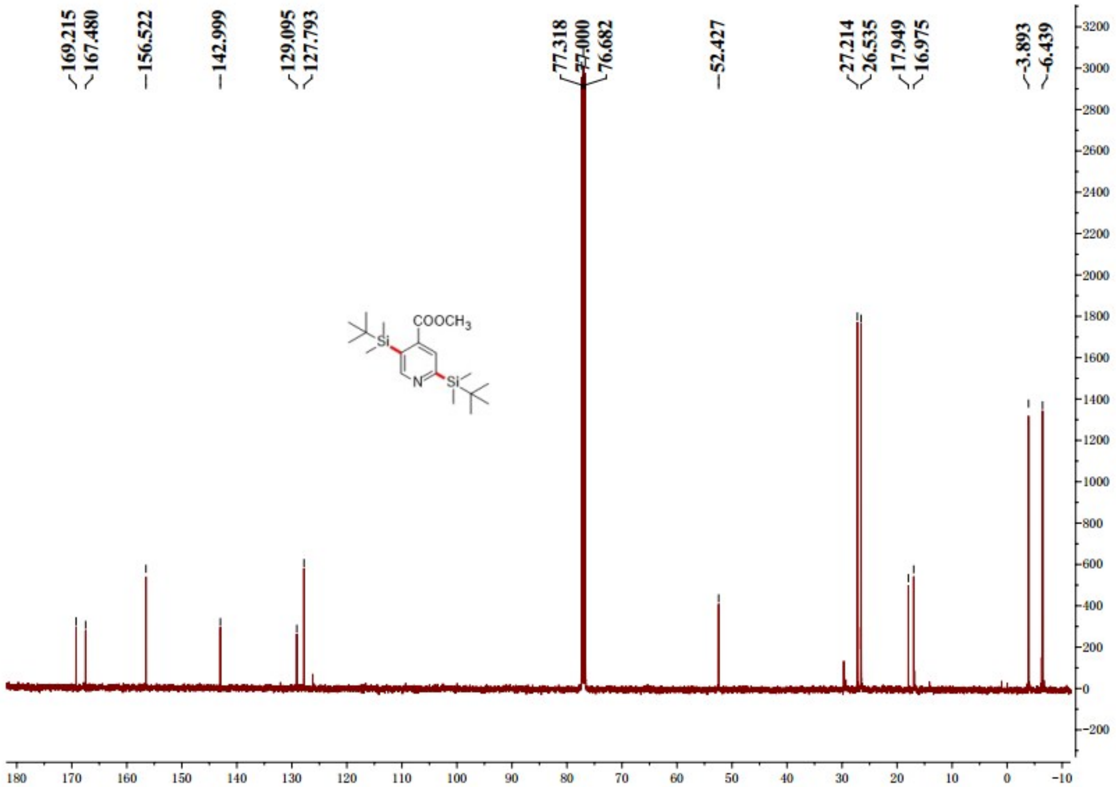
169.215
167.480
156.522
142.999
129.095
127.793
77.318
77.000
76.682
52.427
27.214
26.535
17.949
16.975
-3.893
-6.439
COOCH3
Si
N
Si

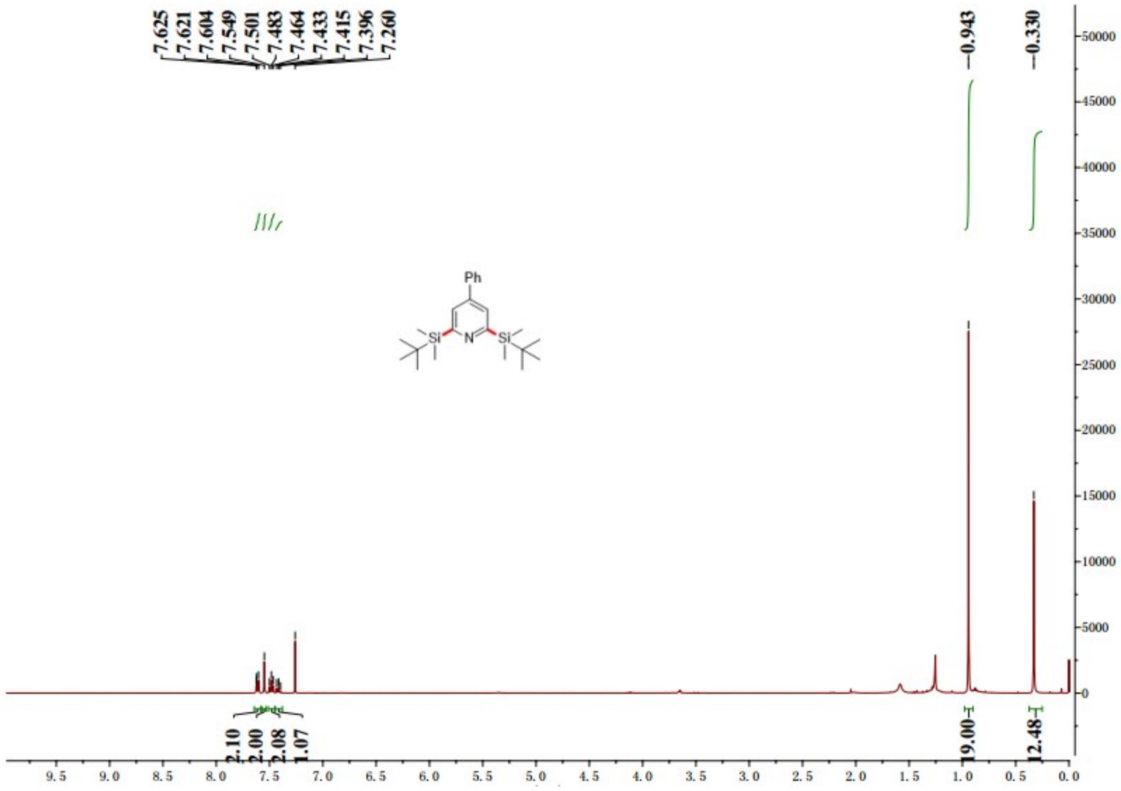
7.625
7.621
7.604
7.549
7.501
7.483
7.464
7.433
7.415
7.396
7.260
0.943
0.330
Ph
Si
N
Si
2.10
2.00
2.08
1.07
19.00
12.48

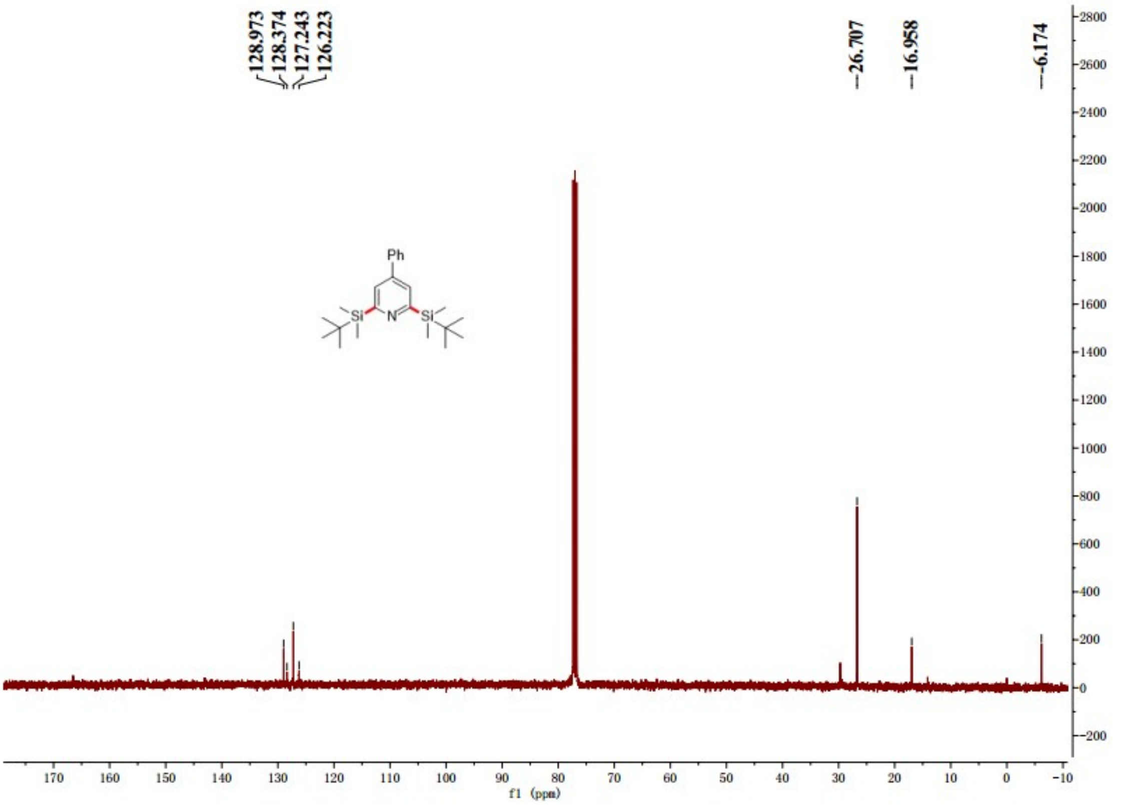
128.973
128.374
127.243
126.223
26.707
16.958
-6.174
Ph
Si
N
Si
f1 (ppm)

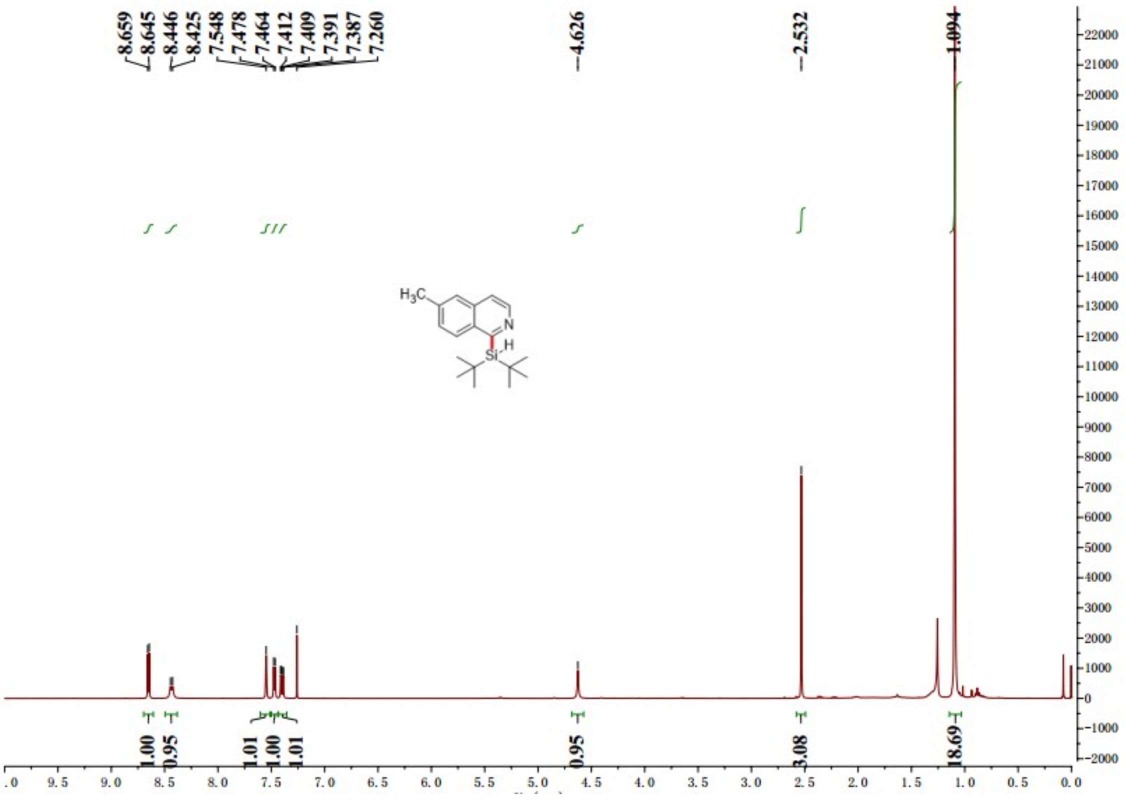
8.659
8.645
8.446
8.425
7.548
7.478
7.464
7.412
7.409
7.391
7.387
7.260
4.626
2.532
1.094
1.00
0.95
1.01
1.00
1.01
0.95
3.08
18.69
H3C
N
H
Si

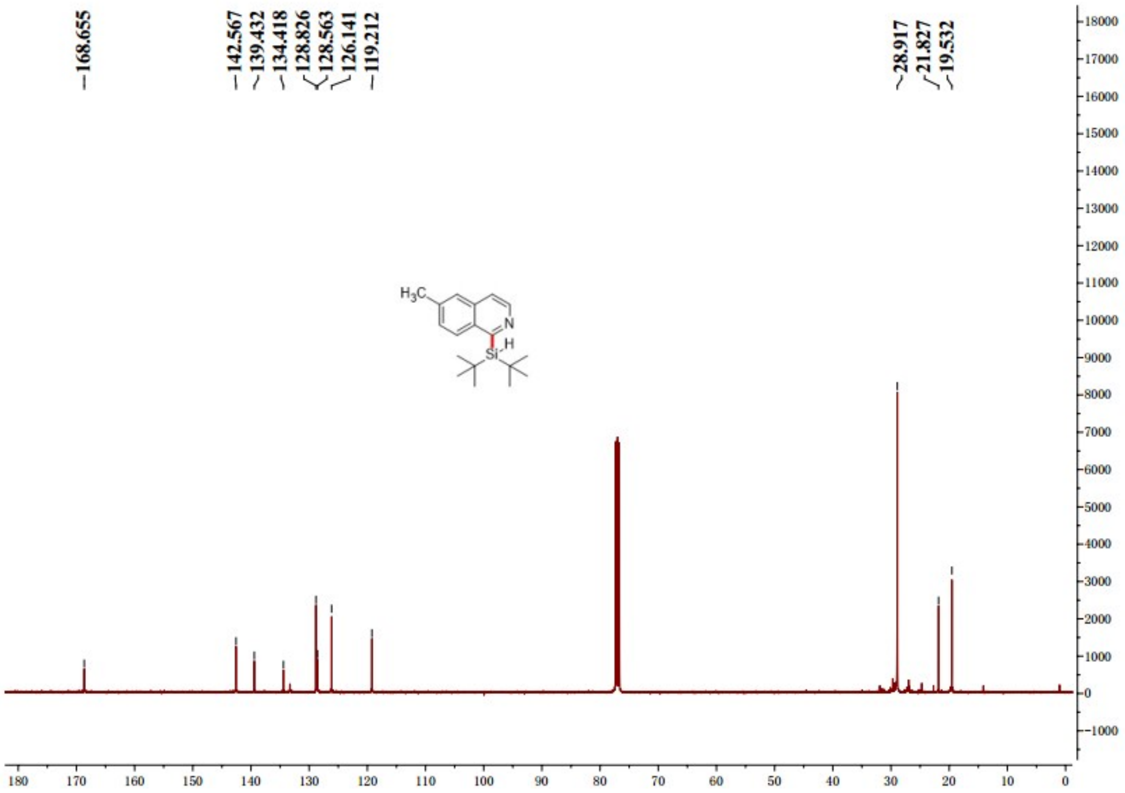
168.655
142.567
139.432
134.418
128.826
128.563
126.141
119.212
28.917
21.827
19.532
H3C
N
H
Si

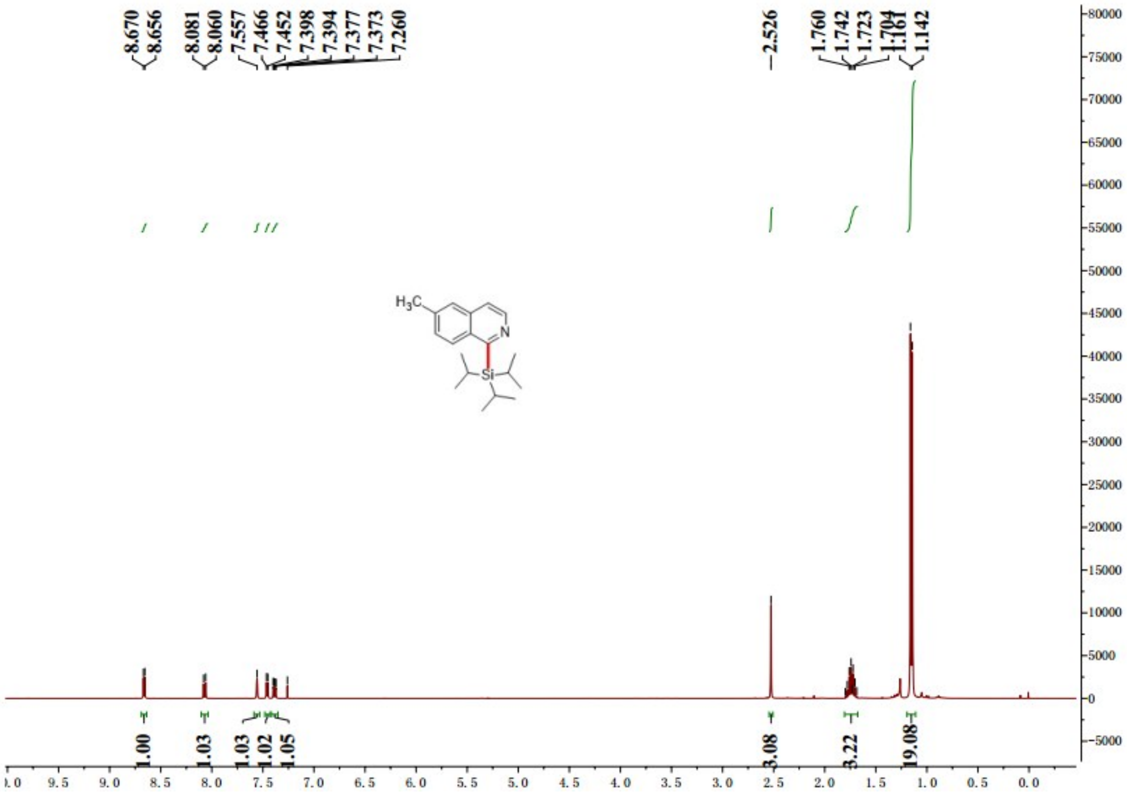

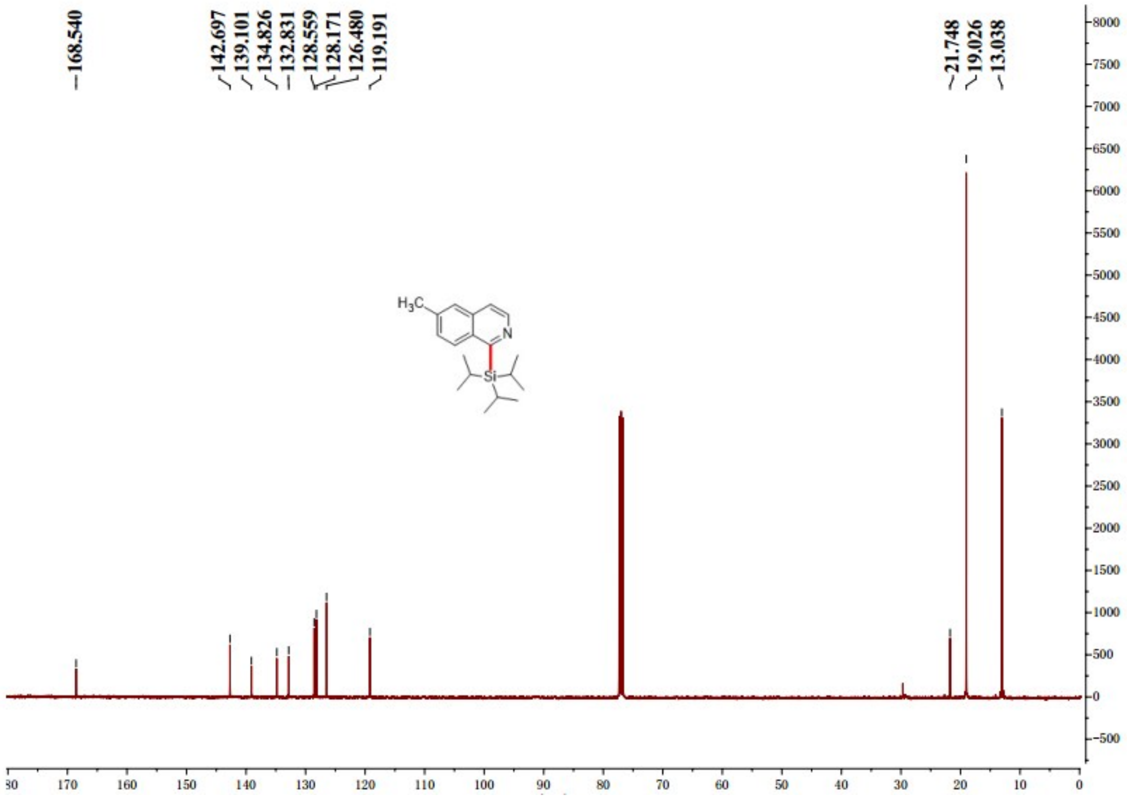

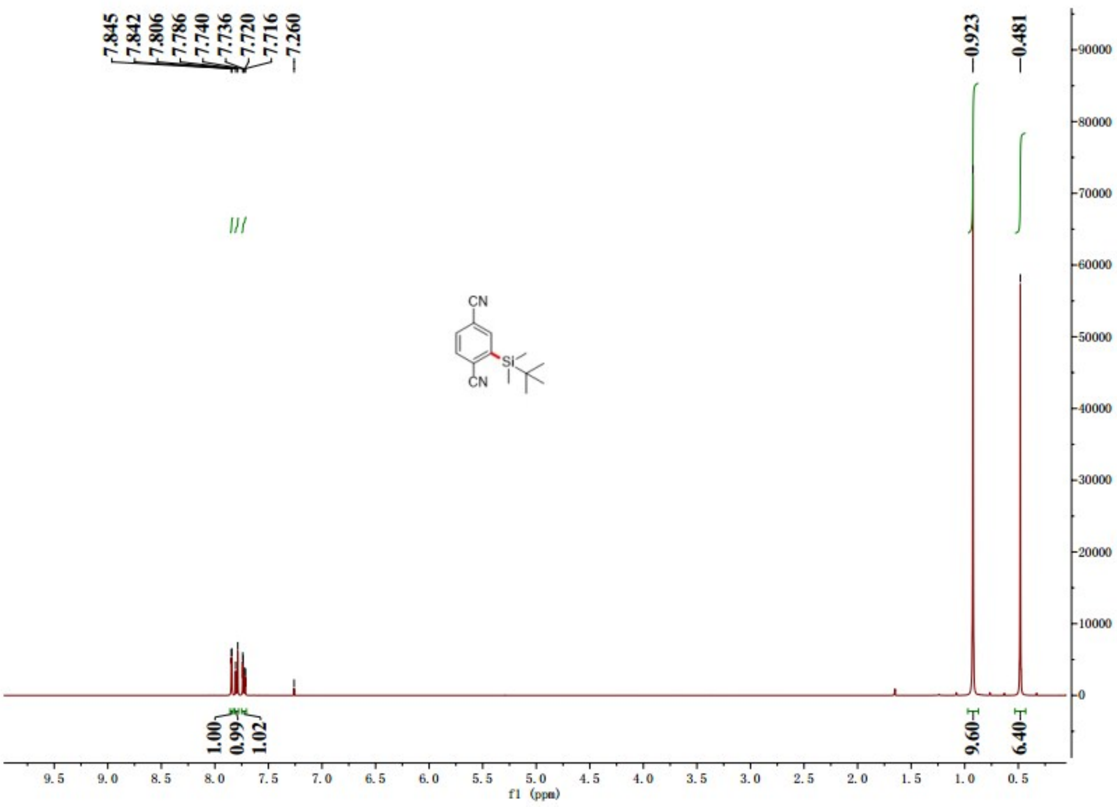
7.845
7.842
7.806
7.786
7.740
7.736
7.720
7.716
7.260
0.923
0.481
CN
Si
CN
1.00
0.99
1.02
9.60
6.40
f1 (ppm)

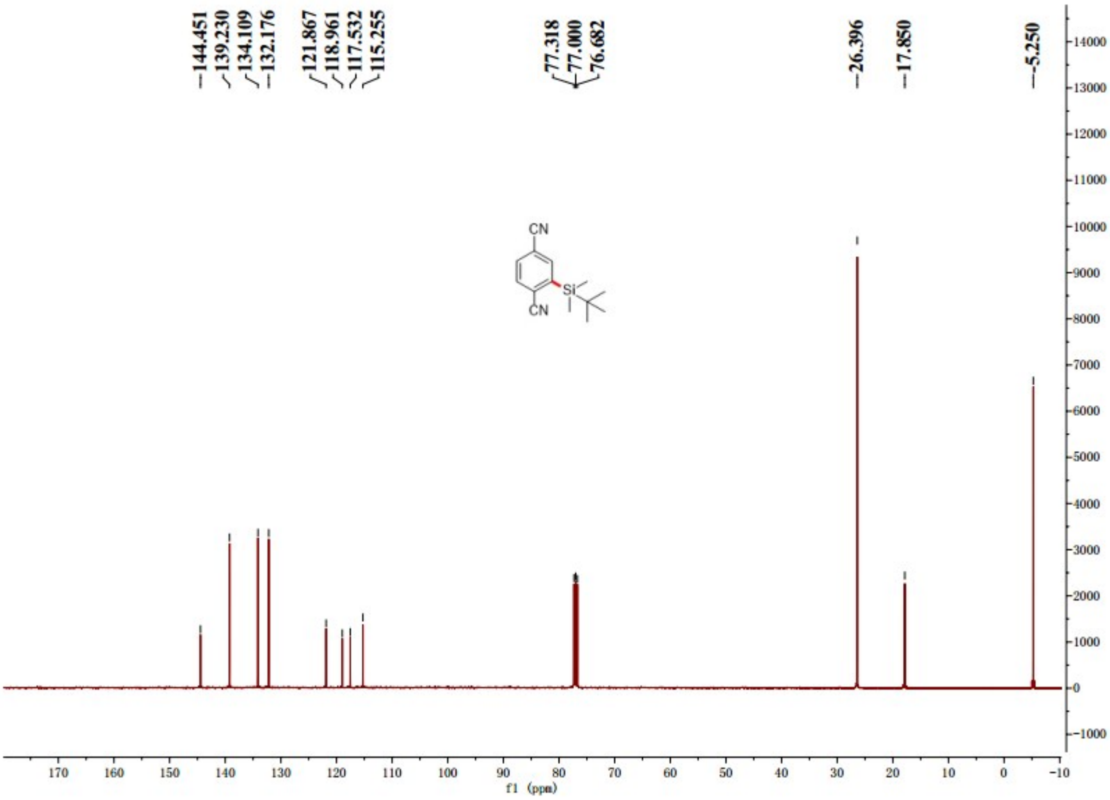
144.451
139.230
134.109
132.176
121.867
118.961
117.532
115.255
77.318
77.000
76.682
26.396
17.850
-5.250
CN
Si
CN
f1 (ppm)

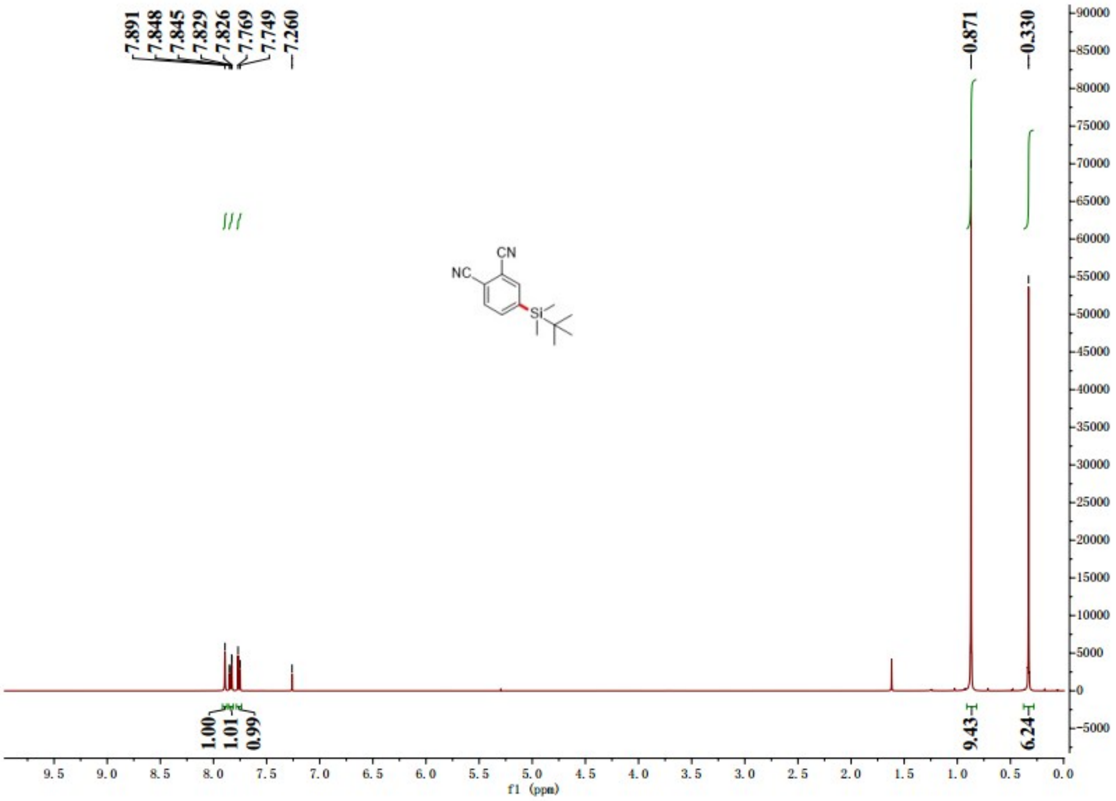
7.891
7.848
7.845
7.829
7.826
7.769
7.749
7.260
0.871
0.330
NC
CN
Si
1.00
1.01
0.99
9.43
6.24
f1 (ppm)

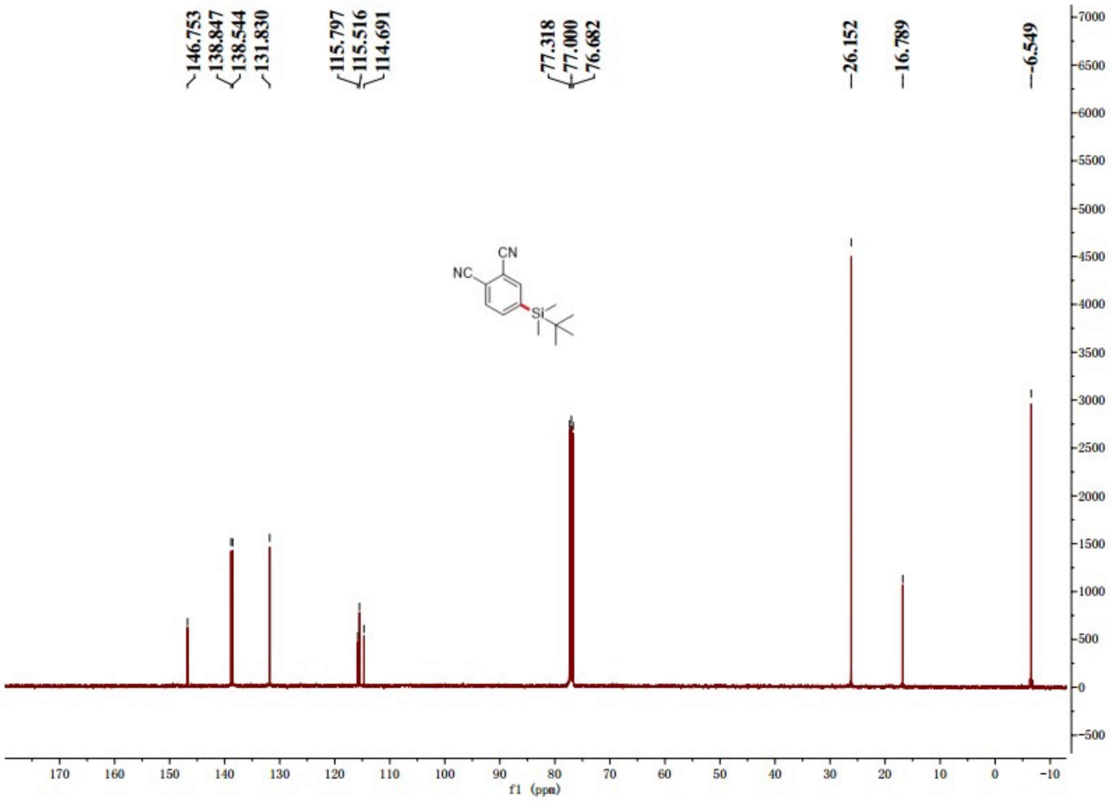
146.753
138.847
138.544
131.830
115.797
115.516
114.691
77.318
77.000
76.682
26.152
16.789
-6.549
NC
CN
Si
f1 (ppm)

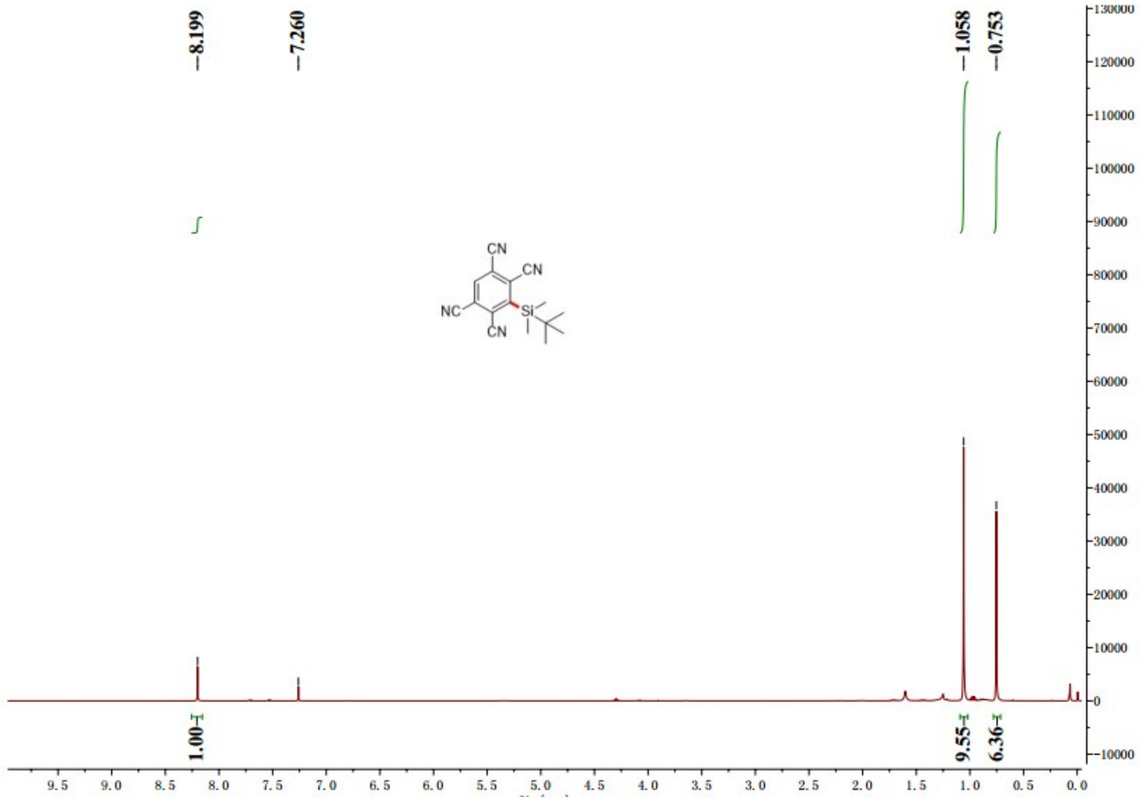

8.199
7.260
1.058
0.753
CN
CN
NC
Si
CN
1.00
9.55
6.36

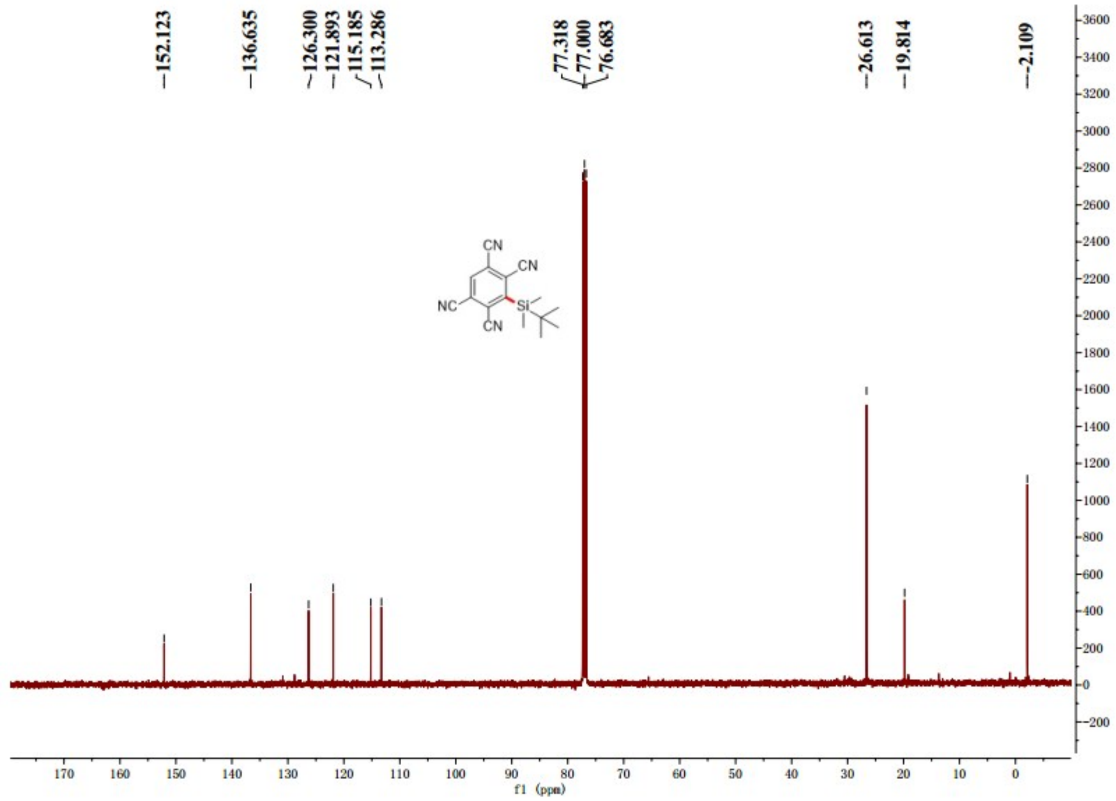

152.123
136.635
126.300
121.893
115.185
113.286
77.318
77.000
76.683
26.613
19.814
-2.109
CN
CN
NC
Si
CN
f1 (ppm)

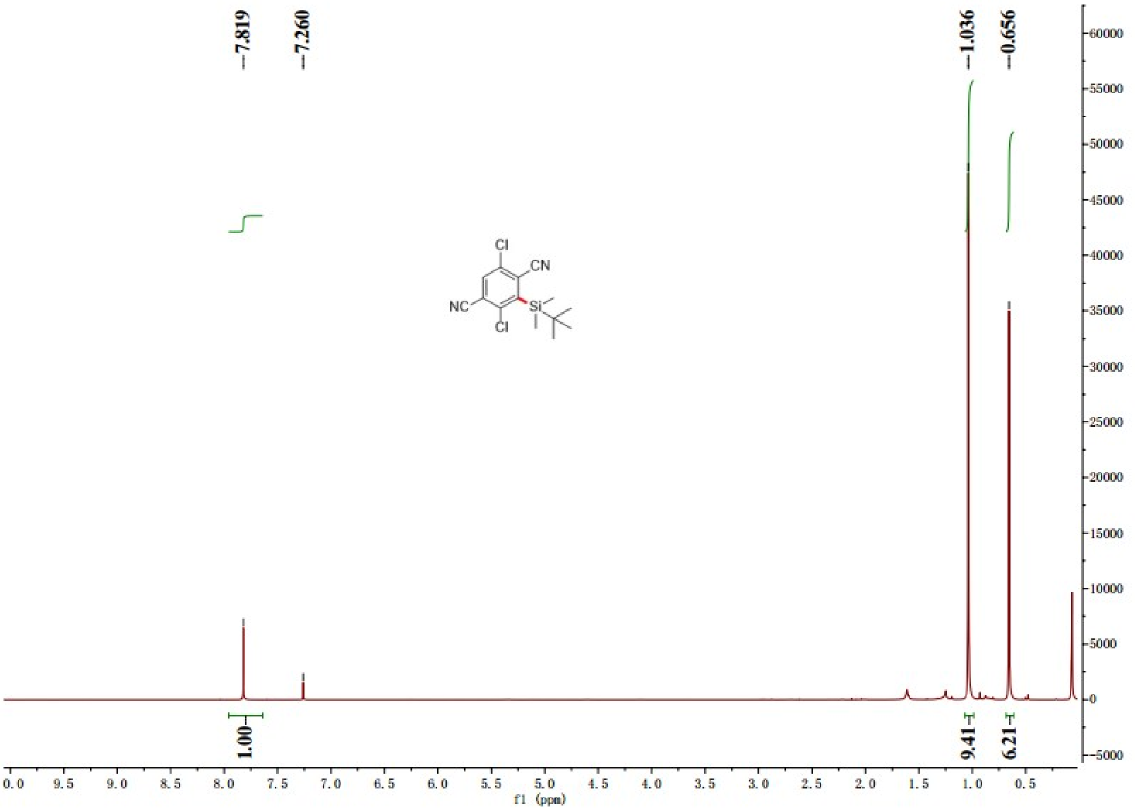
7.819
7.260
1.036
0.656
Cl
CN
NC
Si
Cl
1.00
9.41
6.21
f1 (ppm)

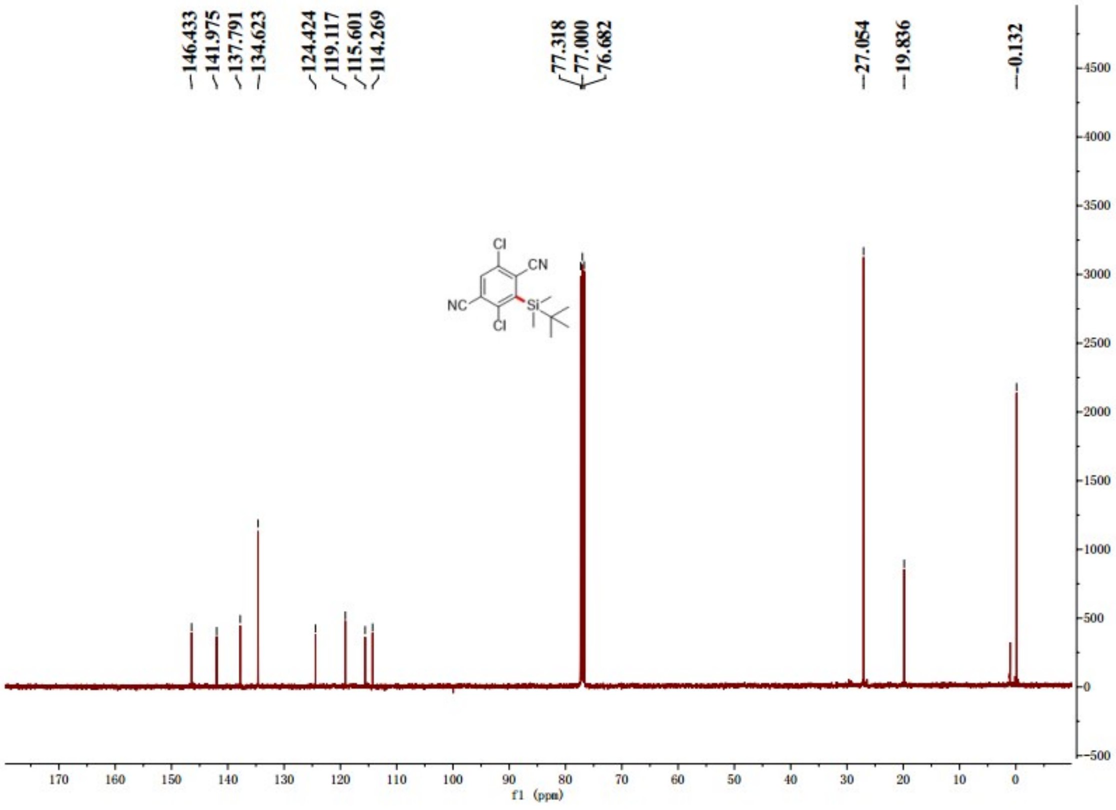
146.433
141.975
137.791
134.623
124.424
119.117
115.601
114.269
77.318
77.000
76.682
27.054
19.836
-0.132
Cl
CN
NC
Si
Cl
f1 (ppm)

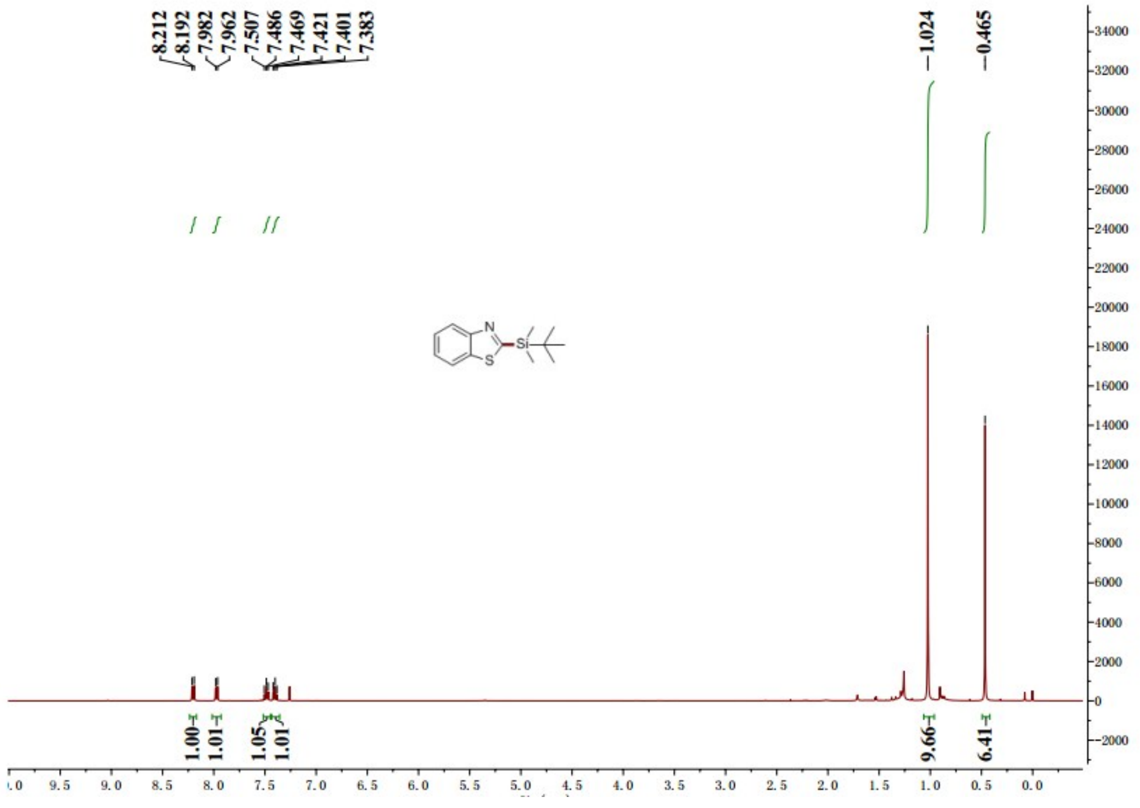
8.212
8.192
7.982
7.962
7.507
7.486
7.469
7.421
7.401
7.383
1.024
0.465
1.00
1.01
1.05
1.01
9.66
6.41
N
S
Si

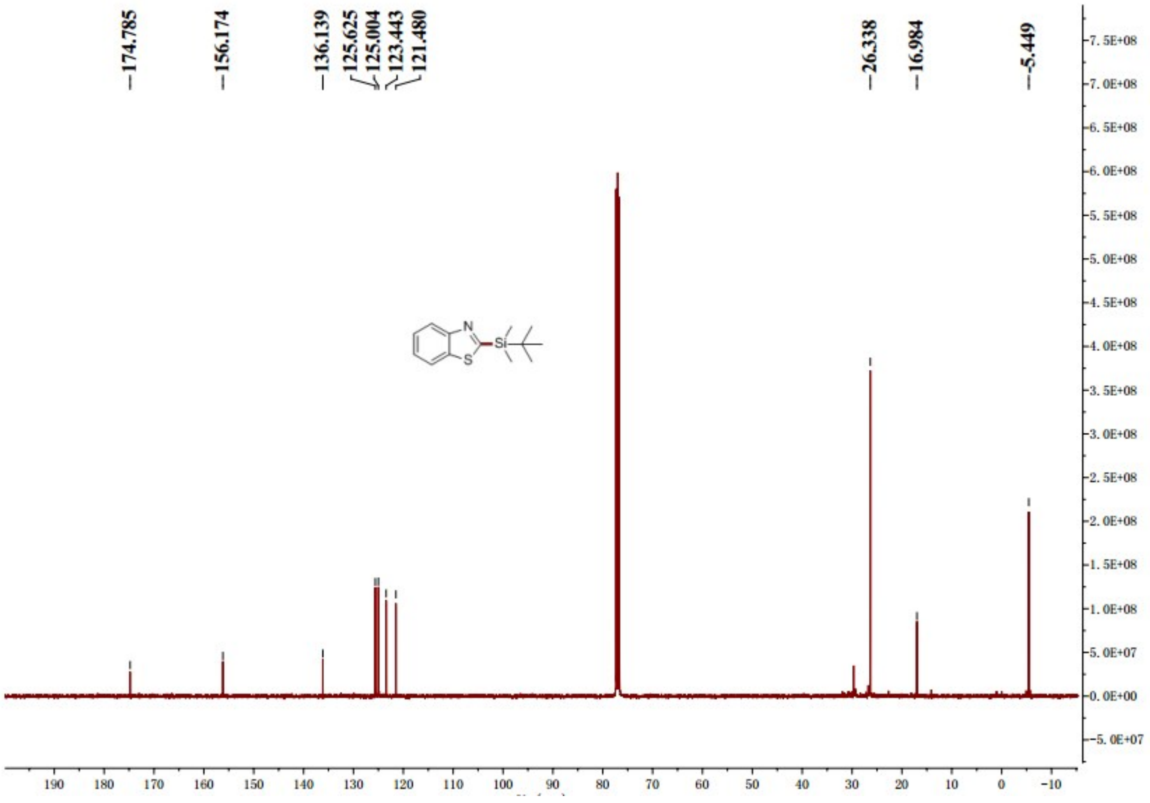
174.785
156.174
136.139
125.625
125.004
123.443
121.480
26.338
16.984
-5.449
N
S
Si

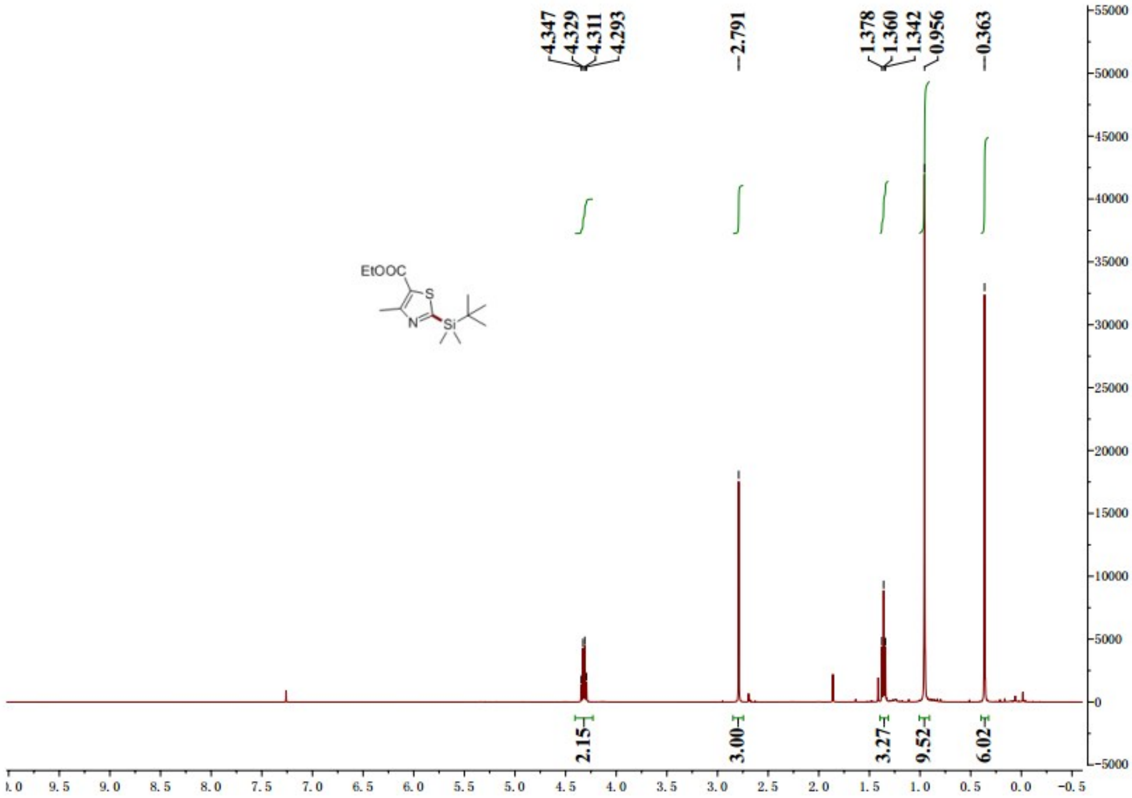
4.347
4.329
4.311
4.293
2.791
1.378
1.360
1.342
0.956
0.363
2.15
3.00
3.27
9.52
6.02
EtOOC
S
N
Si

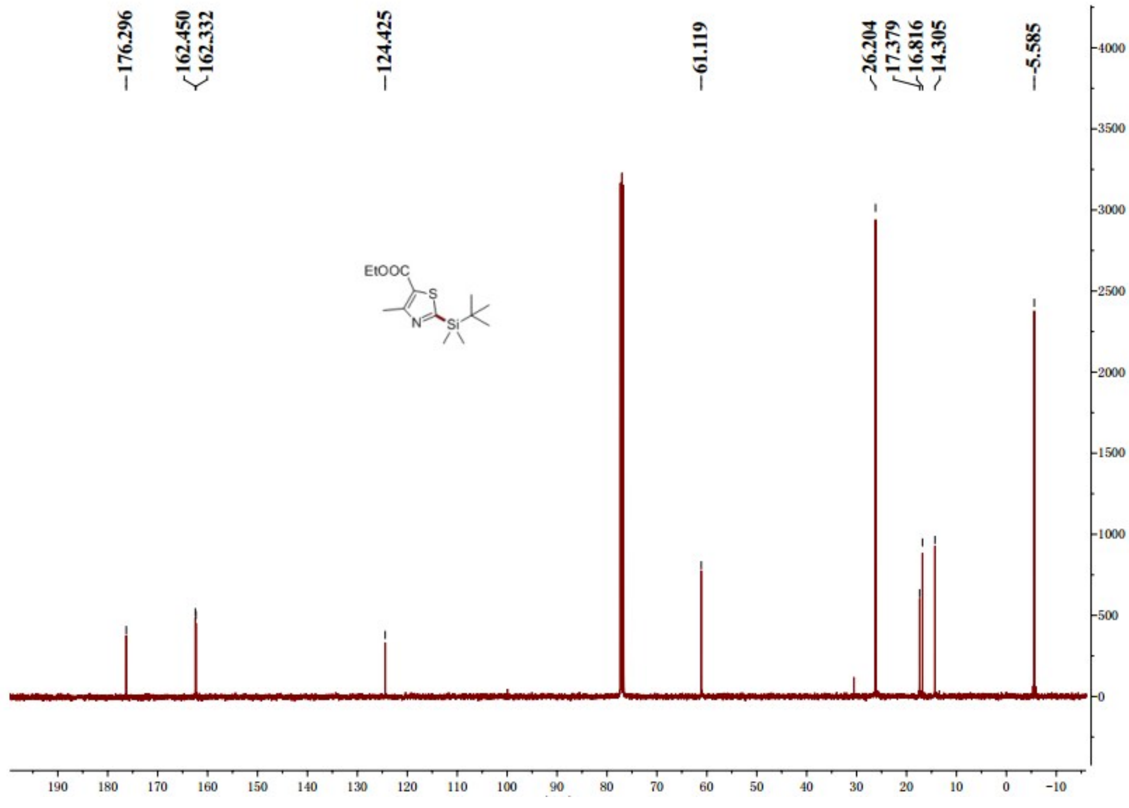
176.296
162.450
162.332
124.425
61.119
26.204
17.379
16.816
14.305
-5.585
EtOOC
S
N
Si

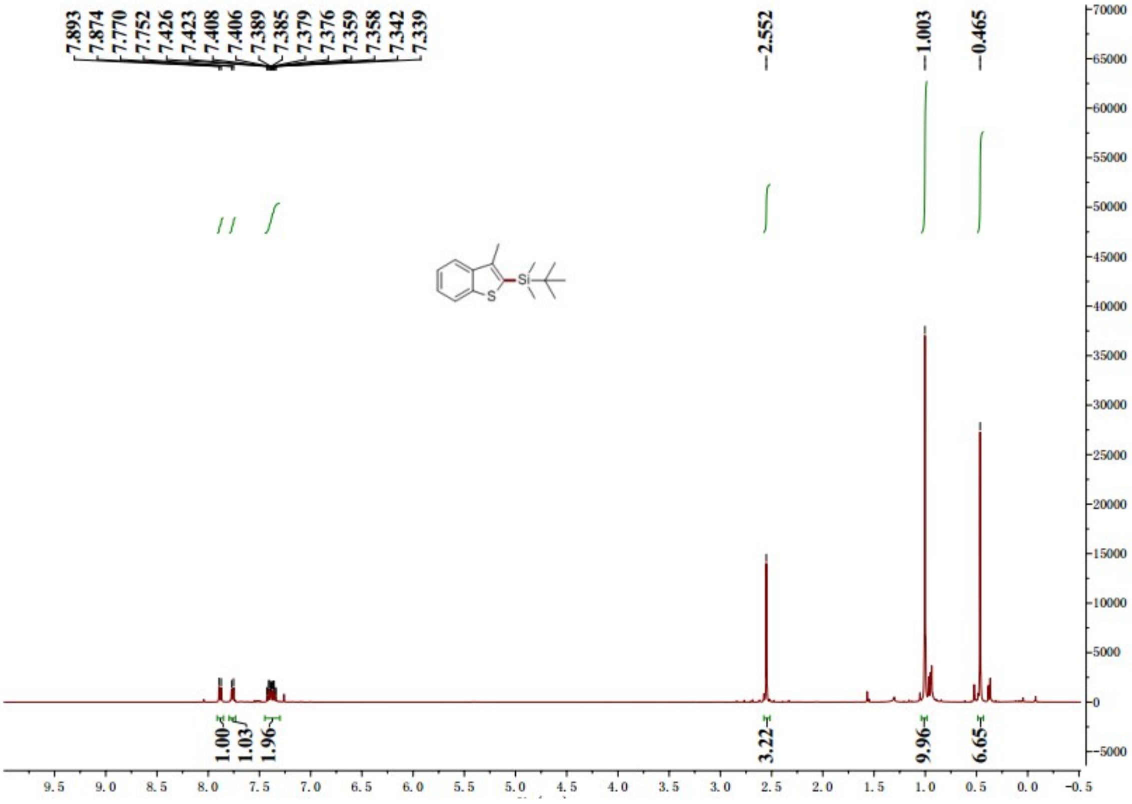
7.893
7.874
7.770
7.752
7.426
7.423
7.408
7.406
7.389
7.385
7.379
7.376
7.359
7.358
7.342
7.339
2.552
1.003
0.465
1.00
1.03
1.96
3.22
9.96
6.65

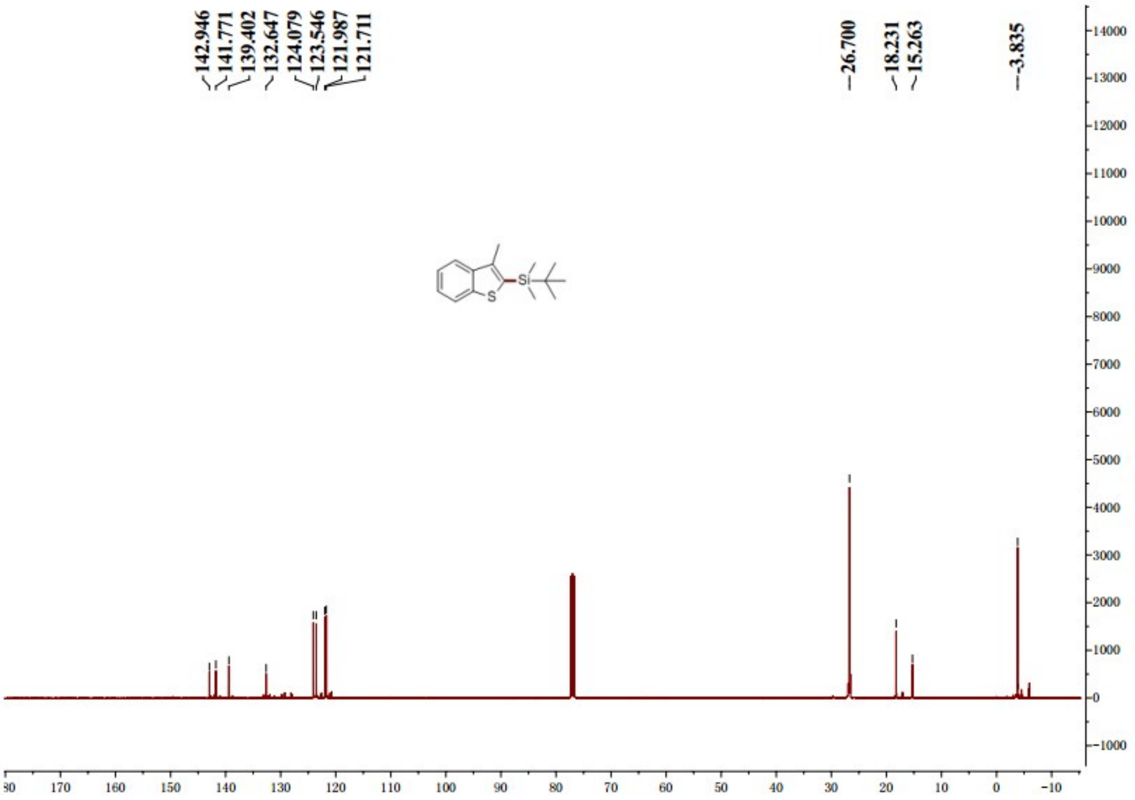
142.946
141.771
139.402
132.647
124.079
123.546
121.987
121.711
26.700
18.231
15.263
-3.835

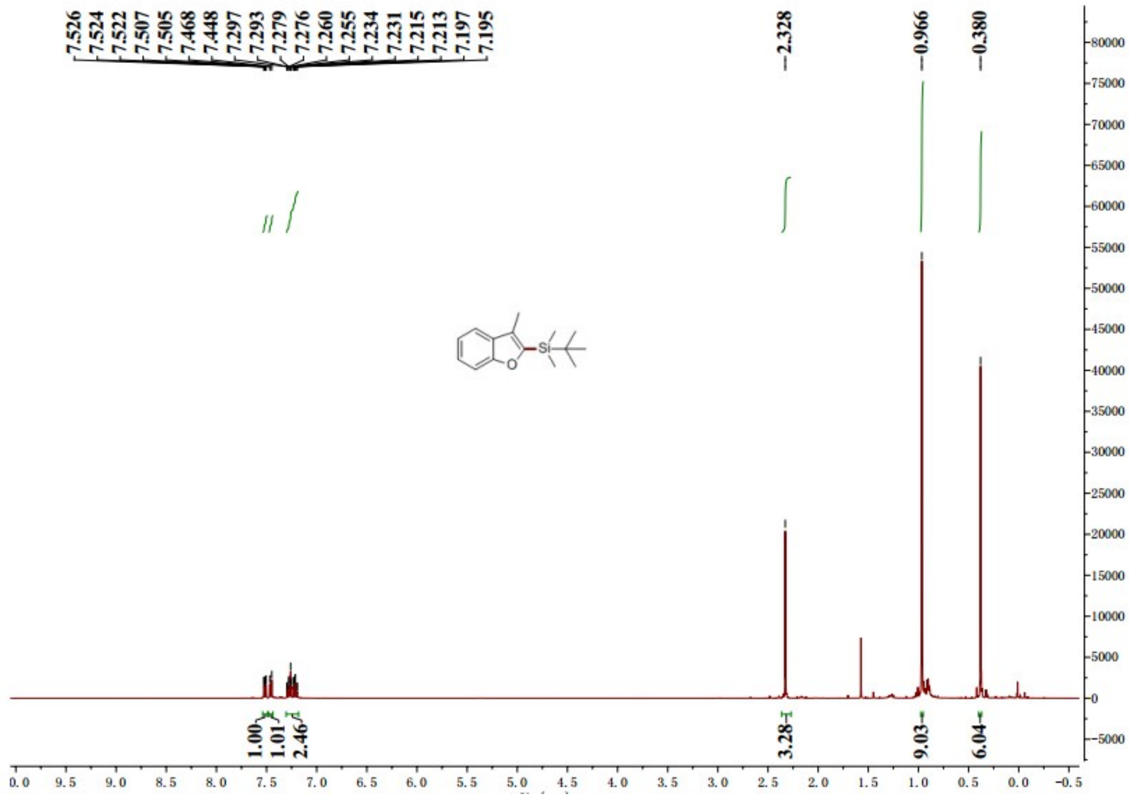
7.526
7.524
7.522
7.507
7.505
7.468
7.448
7.297
7.293
7.279
7.276
7.260
7.255
7.234
7.231
7.215
7.213
7.197
7.195
2.328
0.966
0.380
1.00
1.01
2.46
3.28
9.03
6.04
Si
O

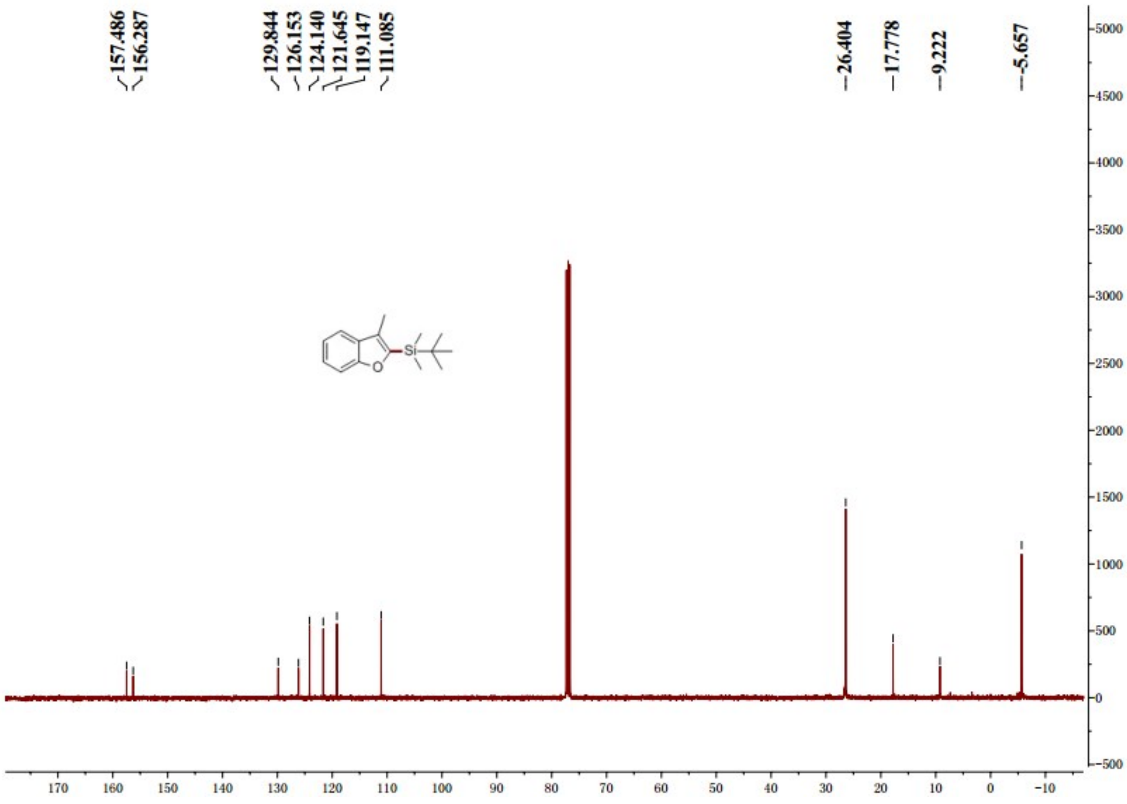
157.486
156.287
129.844
126.153
124.140
121.645
119.147
111.085
26.404
17.778
9.222
-5.657
Si
O

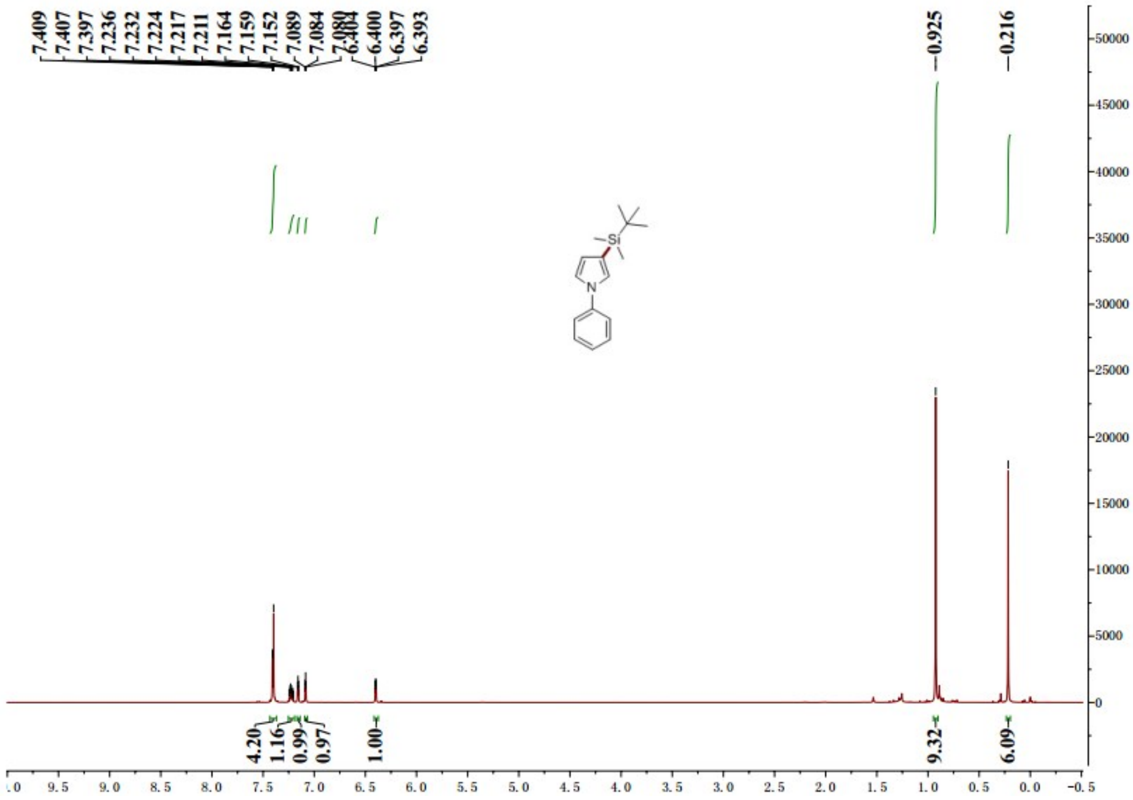
7.409
7.407
7.397
7.236
7.232
7.224
7.217
7.211
7.164
7.159
7.152
7.089
7.084
6.400
6.397
6.393
0.925
0.216
Si
N
4.20
1.16
0.99
0.97
1.00
9.32
6.09

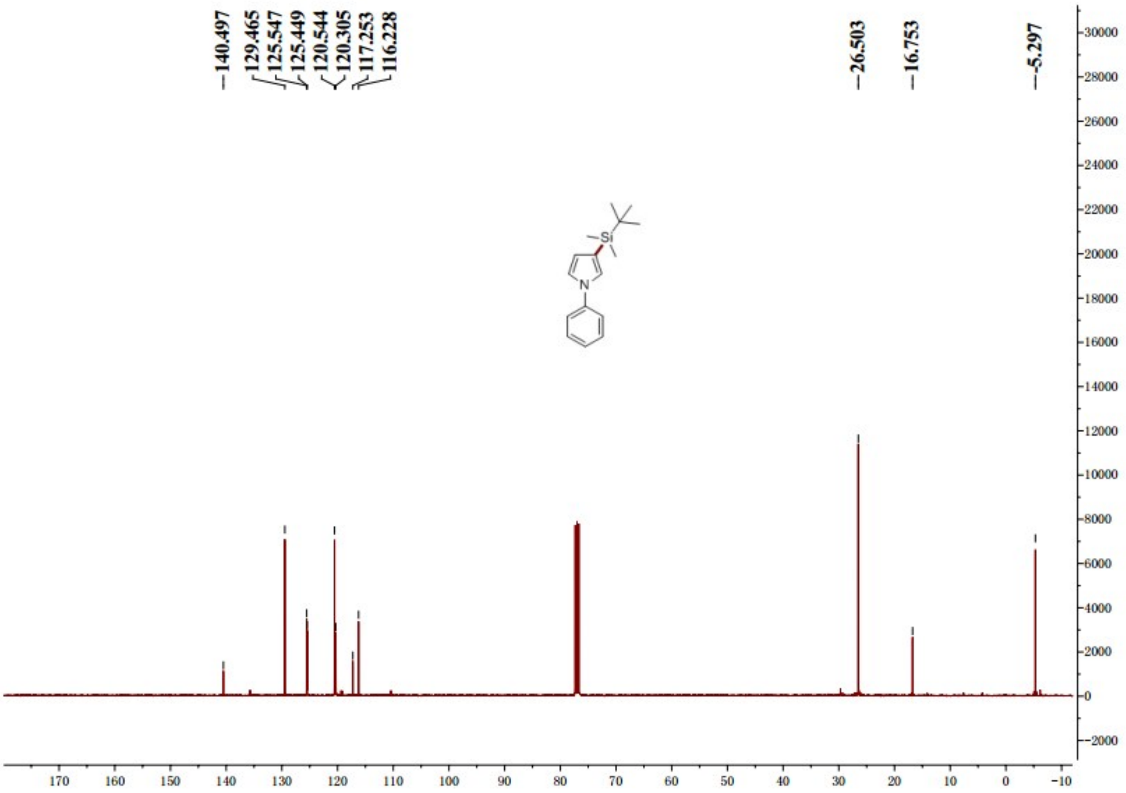
140.497
129.465
125.547
125.449
120.544
120.305
117.253
116.228
26.503
16.753
-5.297
Si
N

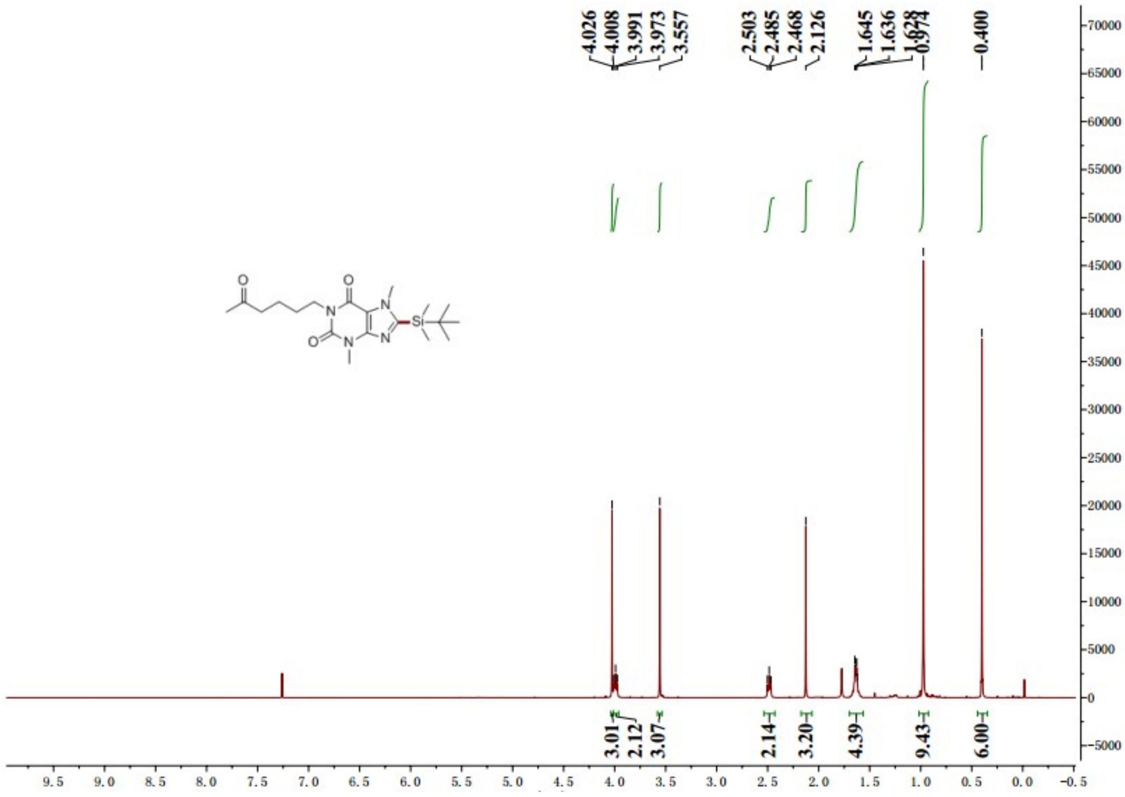
4.026
4.008
3.991
3.973
3.557
2.503
2.485
2.468
2.126
1.645
1.636
1.628
0.974
0.400
3.01
2.12
3.07
2.14
3.20
4.39
9.43
6.00

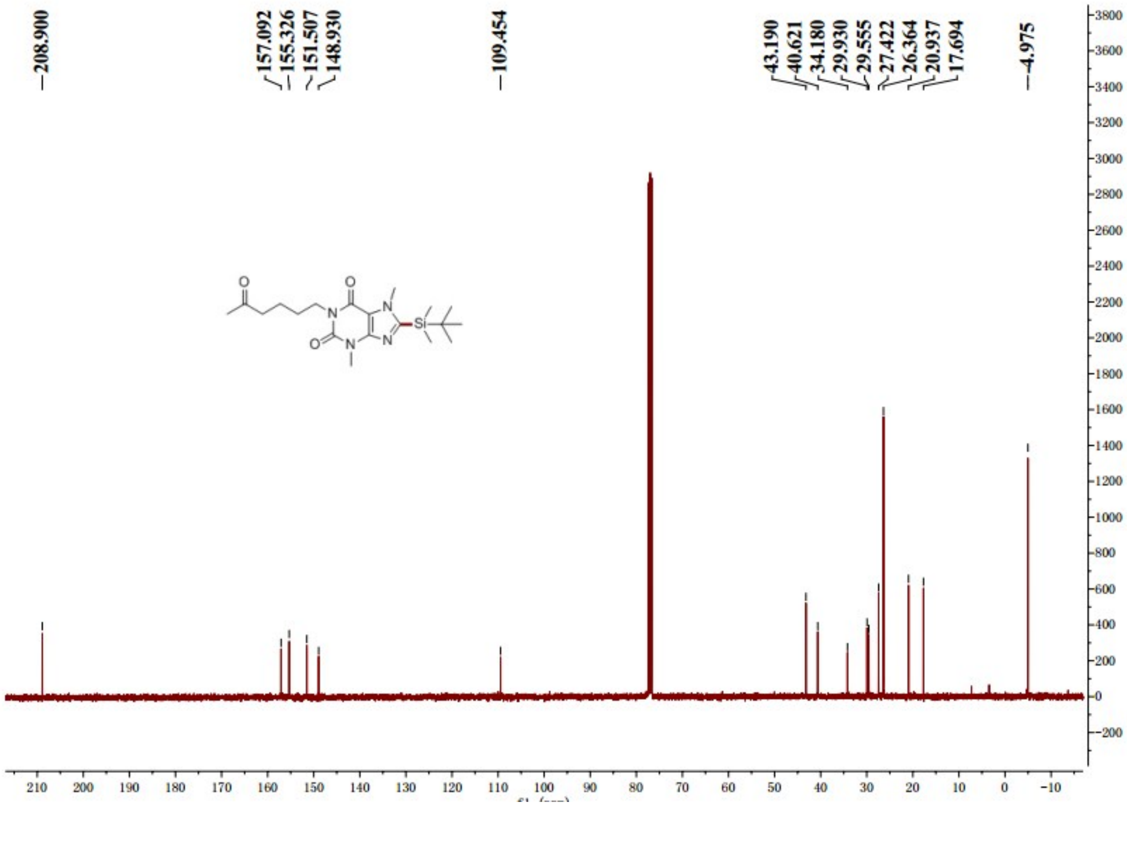
208.900
157.092
155.326
151.507
148.930
109.454
43.190
40.621
34.180
29.930
29.555
27.422
26.364
20.937
17.694
-4.975

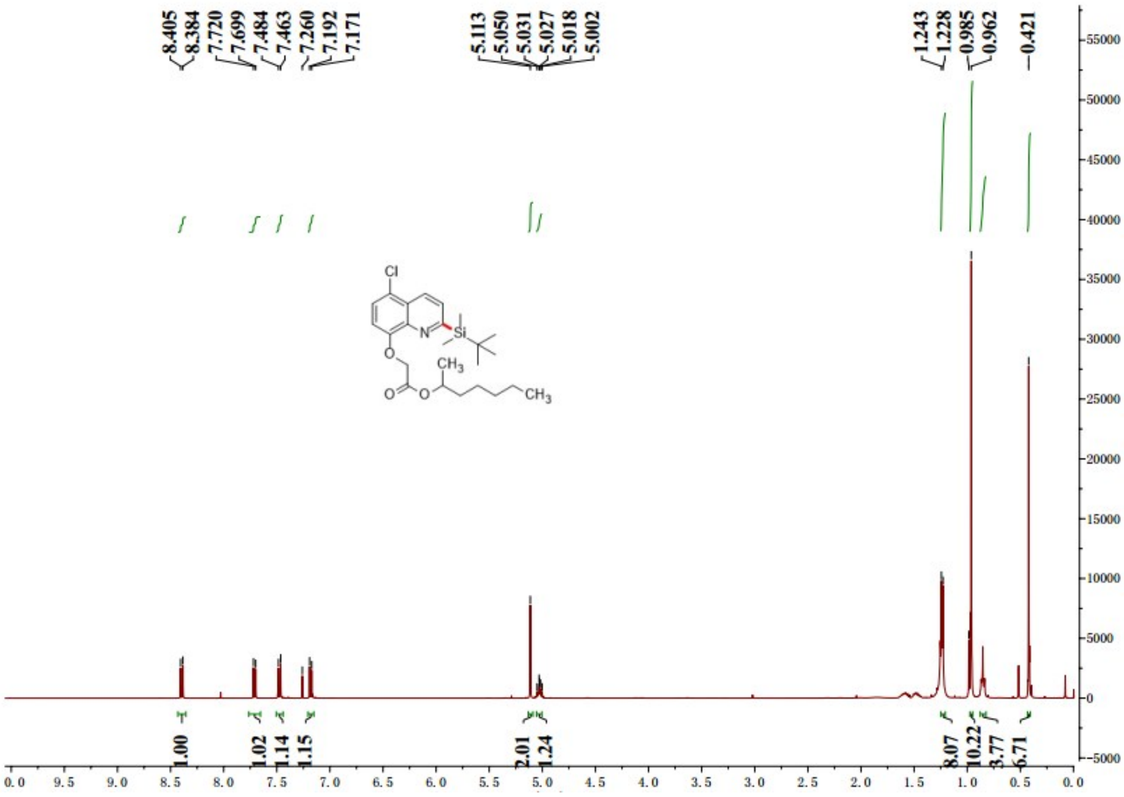
8.405
8.384
7.720
7.699
7.484
7.463
7.260
7.192
7.171
5.113
5.050
5.031
5.027
5.018
5.002
1.243
1.228
0.985
0.962
0.421
Cl
N
Si
O
CH3
CH3
1.00
1.02
1.14
1.15
2.01
1.24
8.07
10.22
3.77
6.71

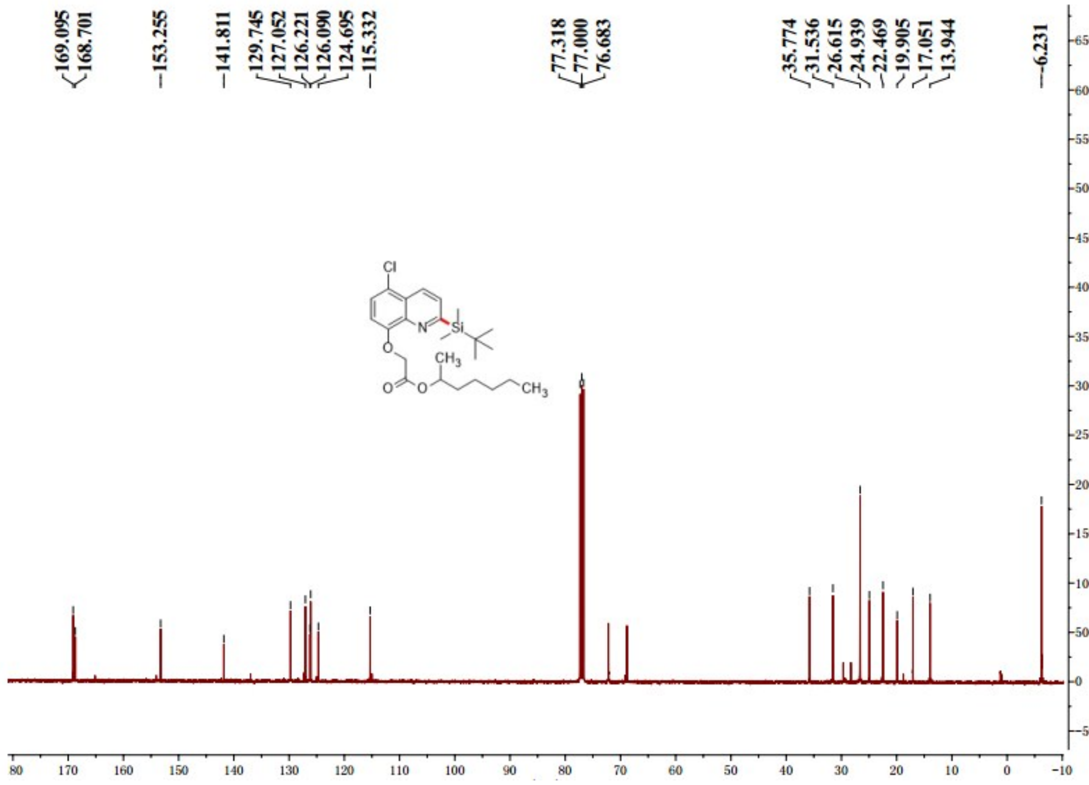
169.095
168.701
153.255
141.811
129.745
127.052
126.221
126.090
124.695
115.332
77.318
77.000
76.683
35.774
31.536
26.615
24.939
22.469
19.905
17.051
13.944
-6.231
Cl
N
Si
O
CH3
CH3

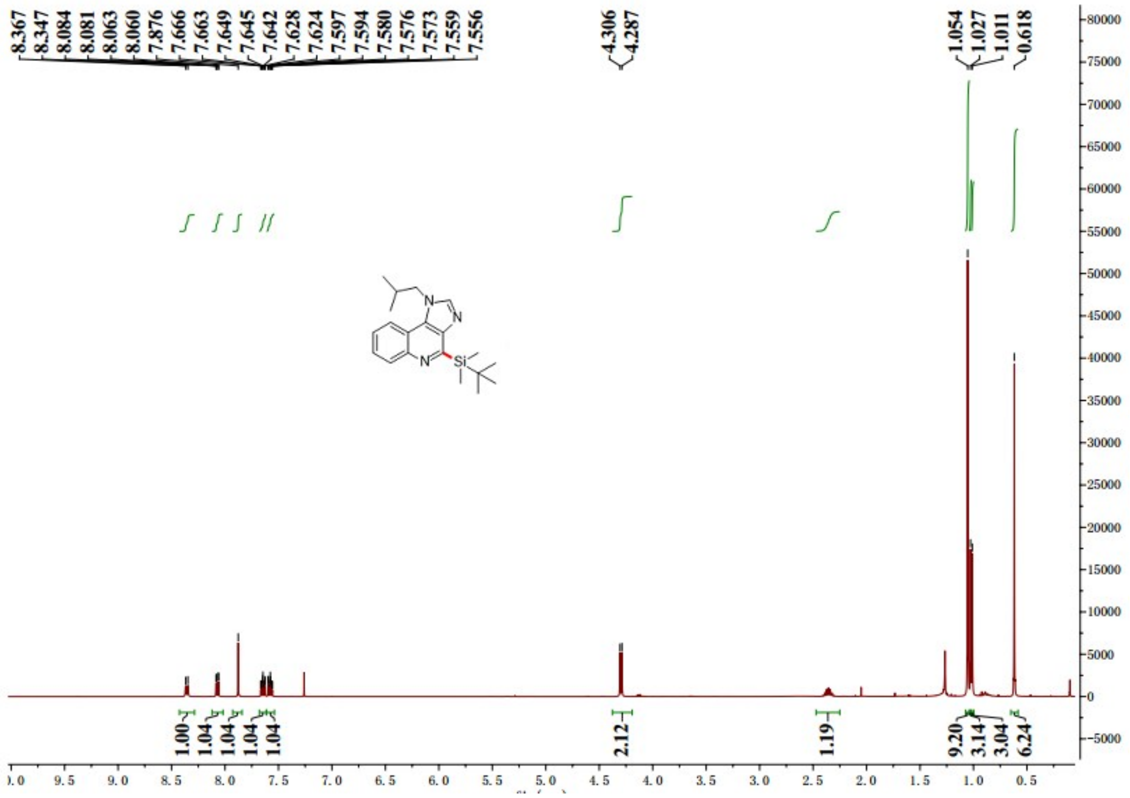
8.367
8.347
8.084
8.081
8.063
8.060
7.876
7.666
7.663
7.649
7.645
7.642
7.628
7.624
7.597
7.594
7.580
7.576
7.573
7.559
7.556
4.306
4.287
1.054
1.027
1.011
0.618
1.00
1.04
1.04
1.04
1.04
2.12
1.19
9.20
3.14
3.04
6.24
Si
N

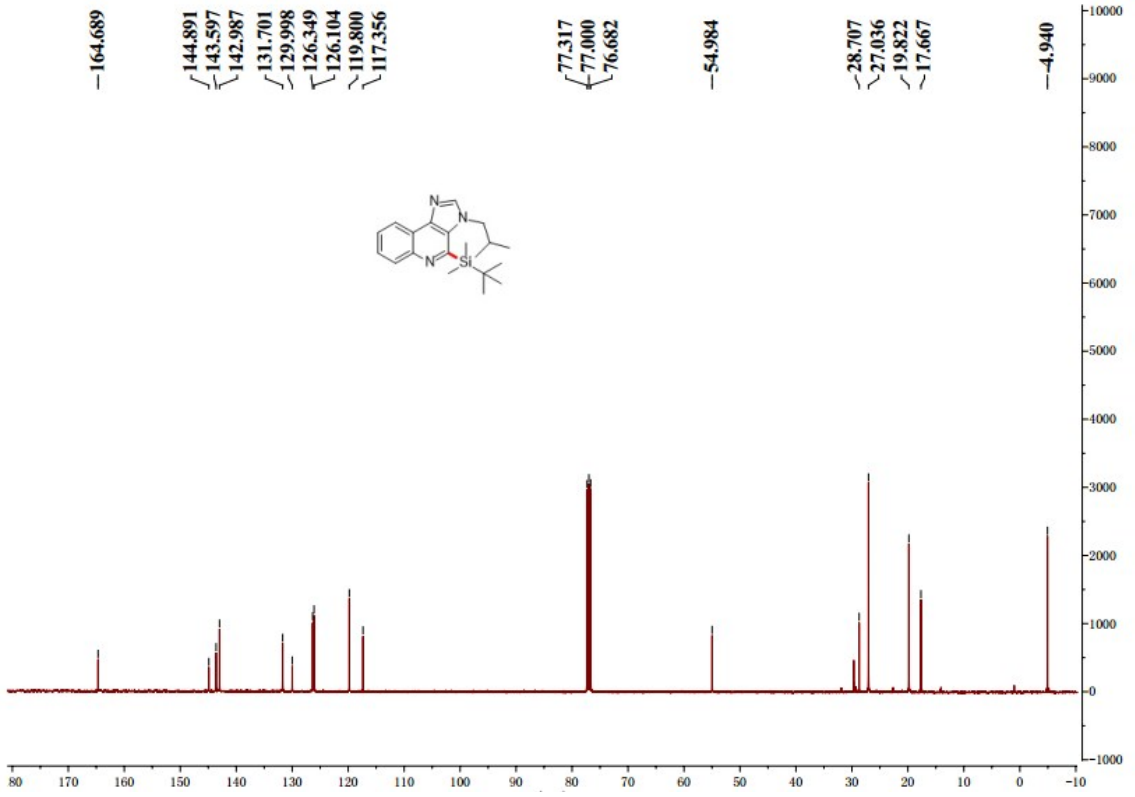
164.689
144.891
143.597
142.987
131.701
129.998
126.349
126.104
119.800
117.356
77.317
77.000
76.682
54.984
28.707
27.036
19.822
17.667
-4.940
Si
N

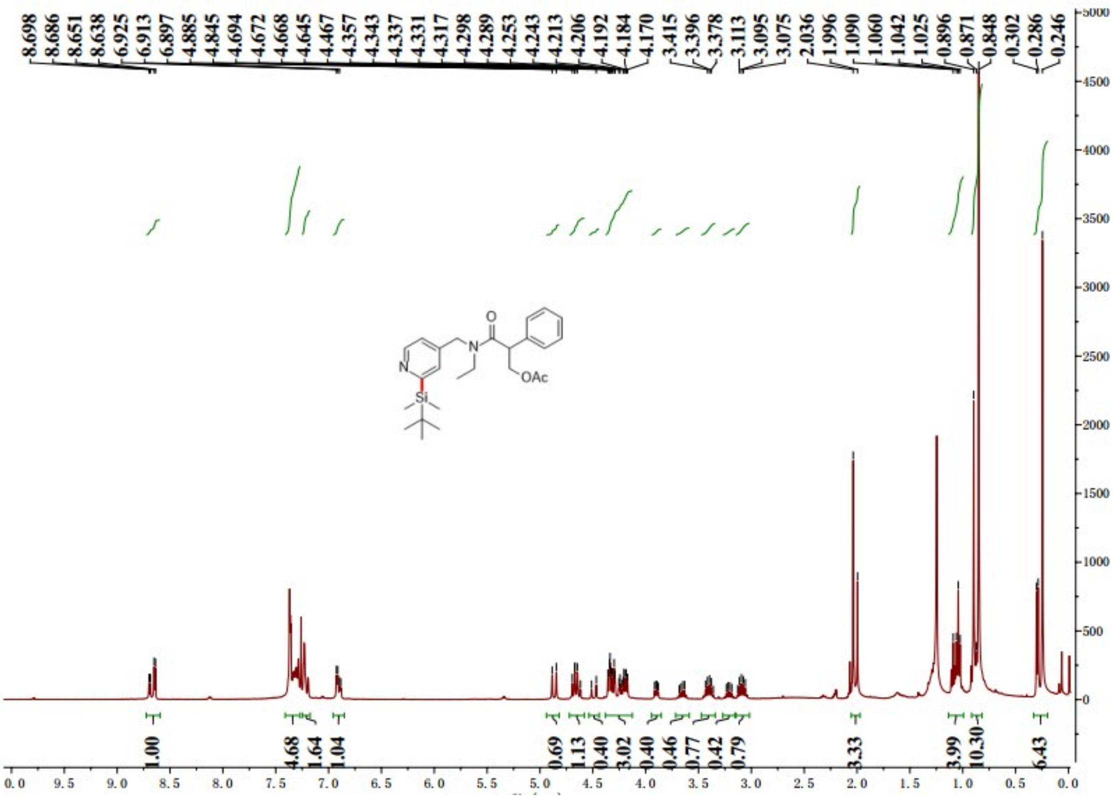

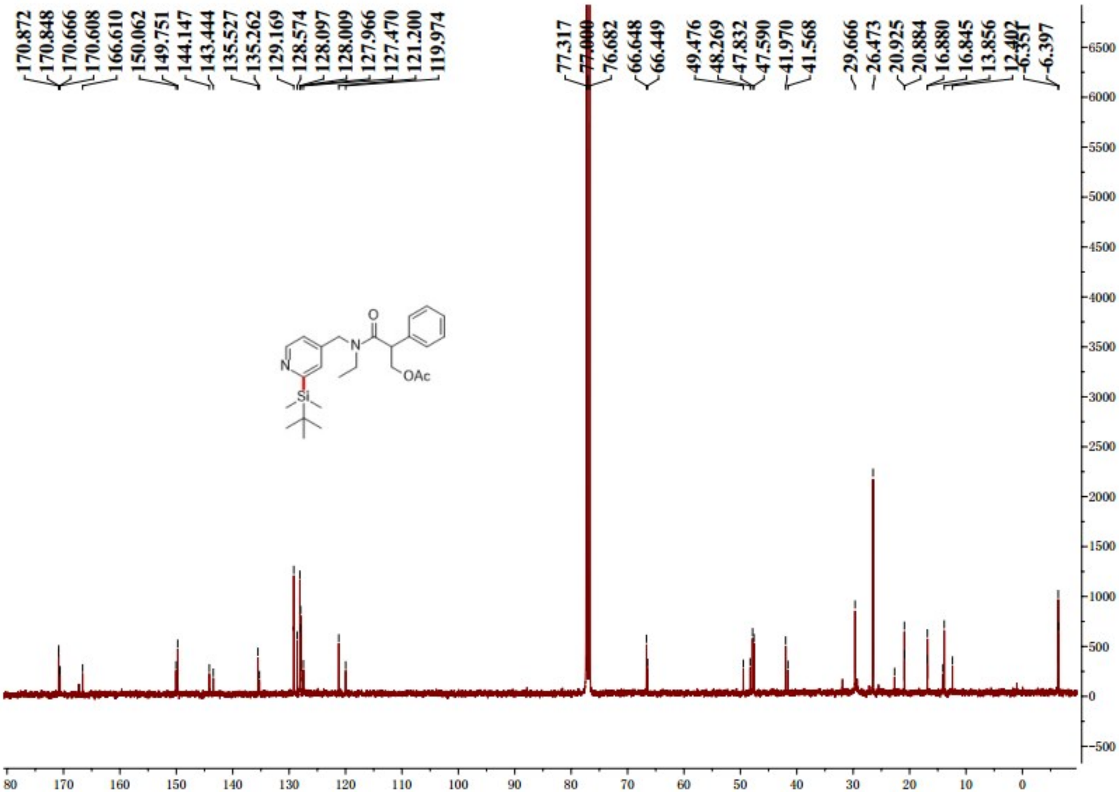

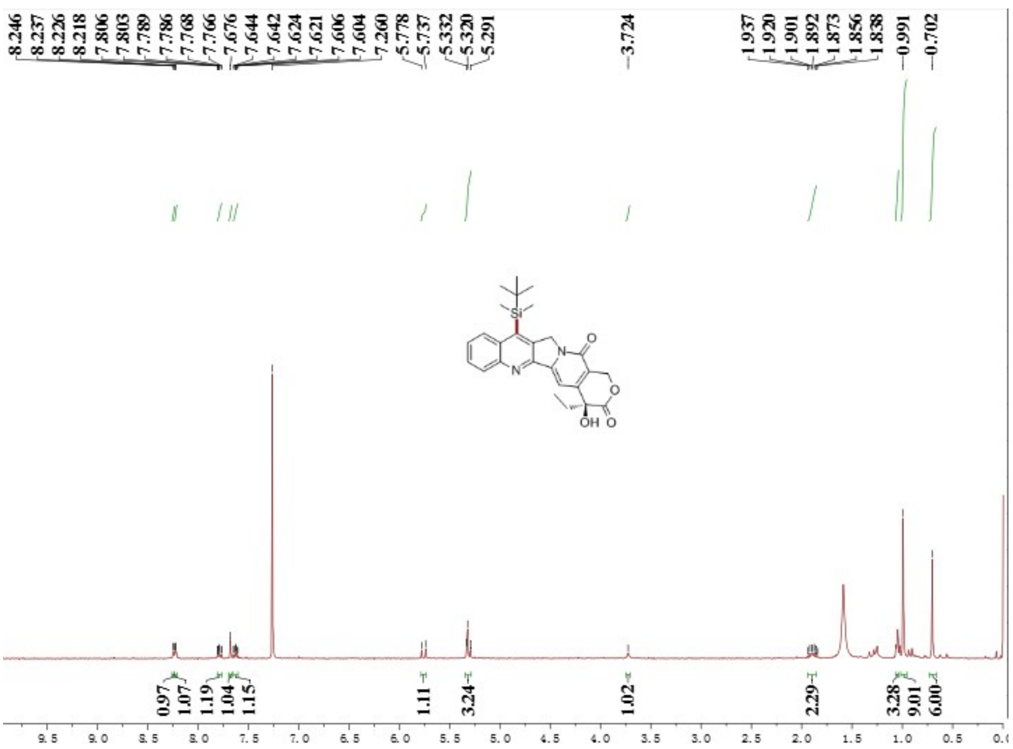
8.246 8.237 8.226 8.218 7.806 7.803 7.789 7.786 7.768 7.766 7.676 7.644 7.642 7.624 7.621 7.606 7.604 7.260 5.778 5.737 5.332 5.320 5.291 3.724 1.937 1.920 1.901 1.892 1.873 1.856 1.838 0.991 0.702
0.97 1.07 1.19 1.04 1.15 1.11 3.24 1.02 2.29 3.28 9.01 6.00
9.5 9.0 8.5 8.0 7.5 7.0 6.5 6.0 5.5 5.0 4.5 4.0 3.5 3.0 2.5 2.0 1.5 1.0 0.5
Si
N
O
OH

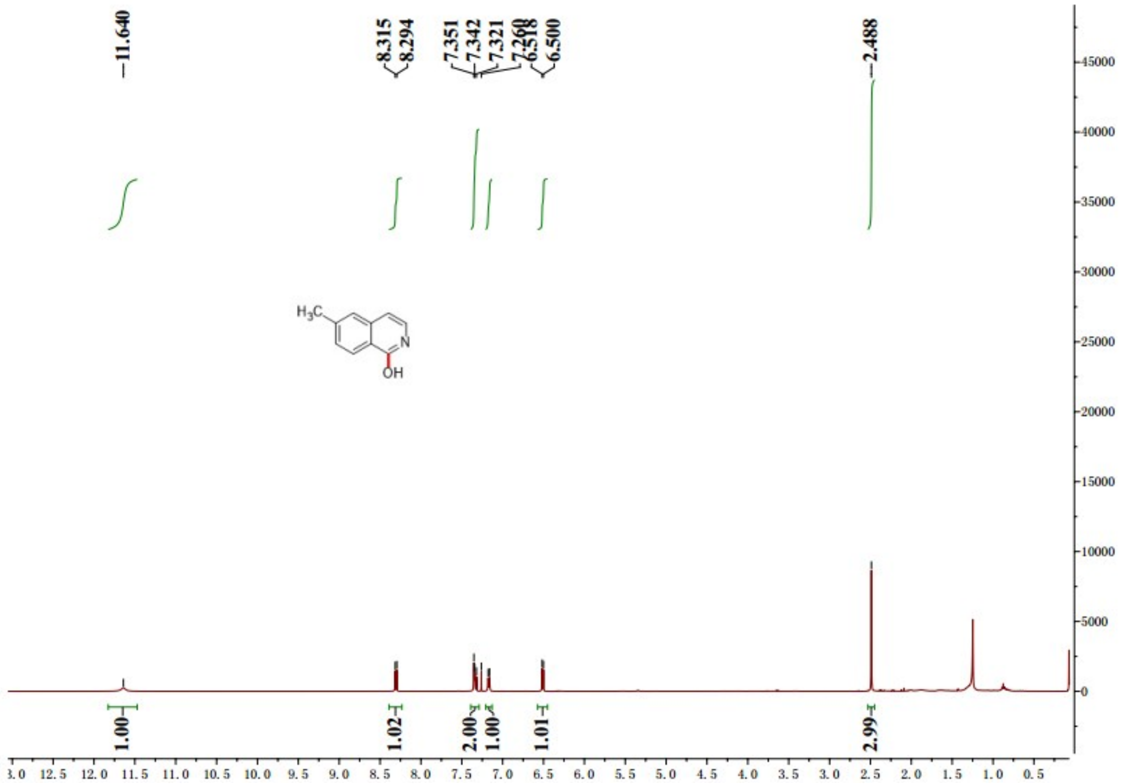
11.640
8.315
8.294
7.351
7.342
7.321
7.260
6.518
6.500
2.488
1.00
1.02
2.00
1.00
1.01
2.99
H3C
N
OH

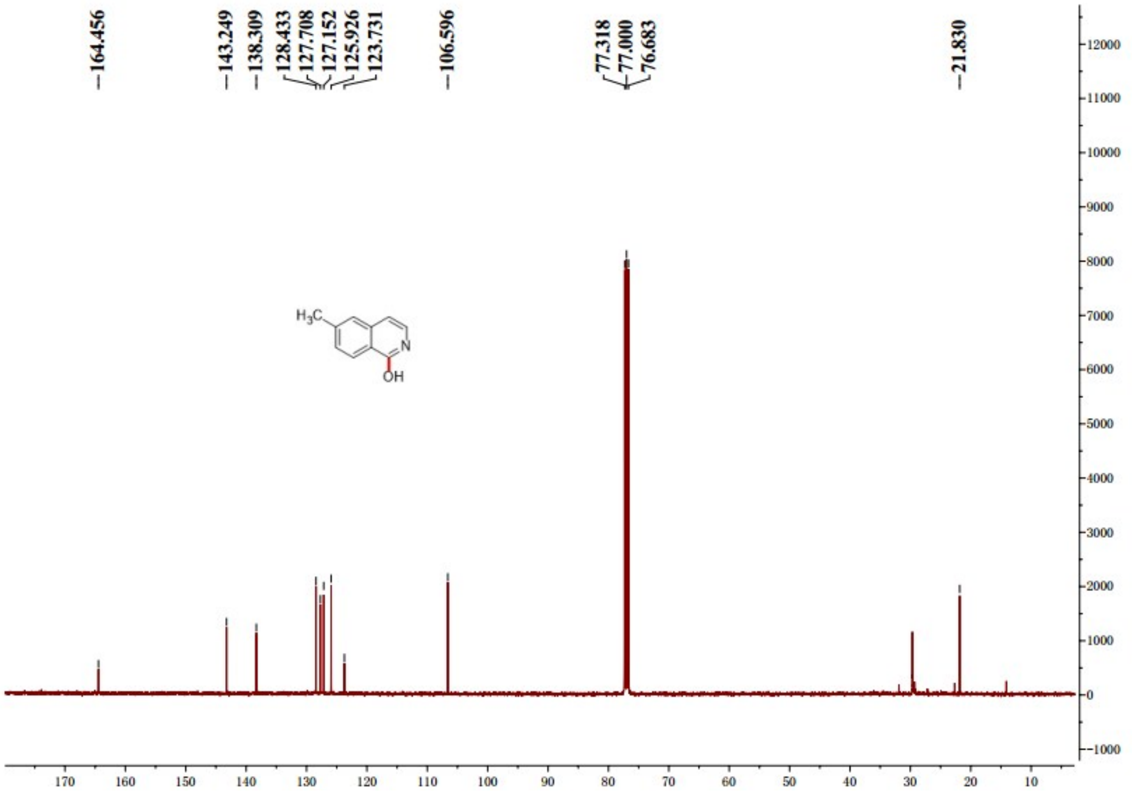
164.456
143.249
138.309
128.433
127.708
127.152
125.926
123.731
106.596
77.318
77.000
76.683
21.830
H3C
N
OH

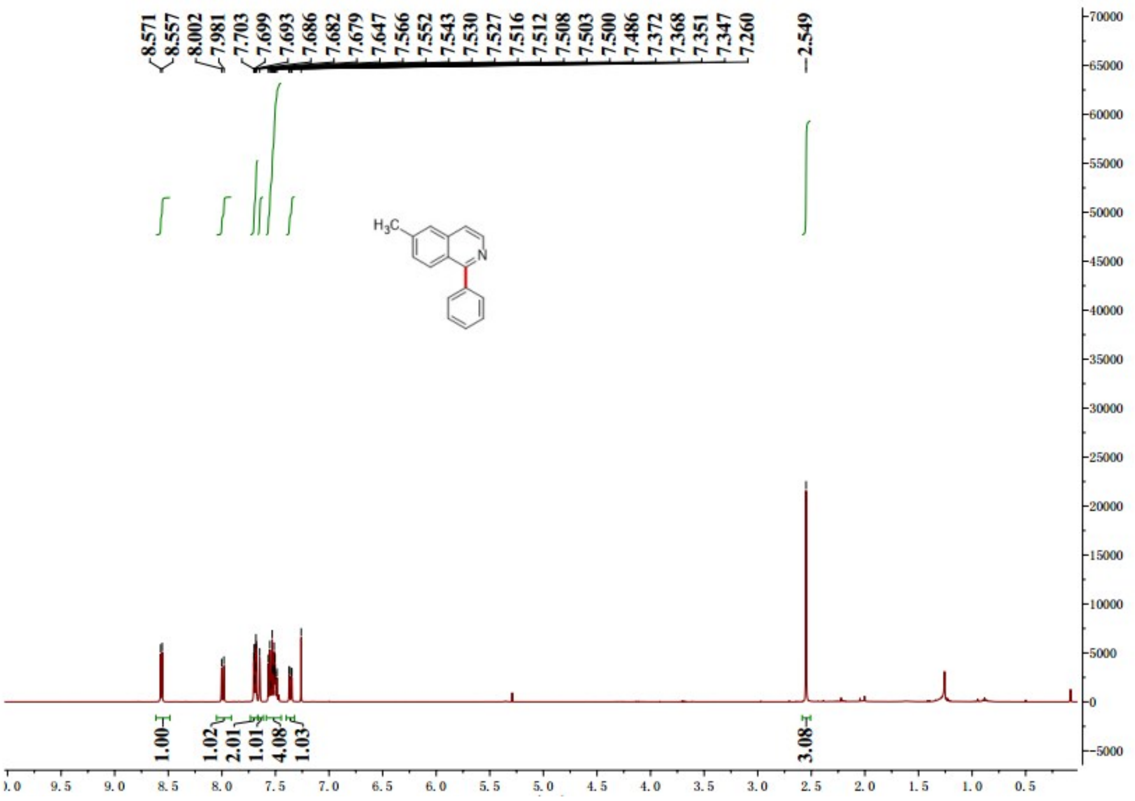
8.571
8.557
8.002
7.981
7.703
7.699
7.693
7.686
7.682
7.679
7.647
7.566
7.552
7.543
7.530
7.527
7.516
7.512
7.508
7.503
7.500
7.486
7.372
7.368
7.351
7.347
7.260
2.549
H3C
N
1.00
1.02
2.01
1.01
4.08
1.03
3.08

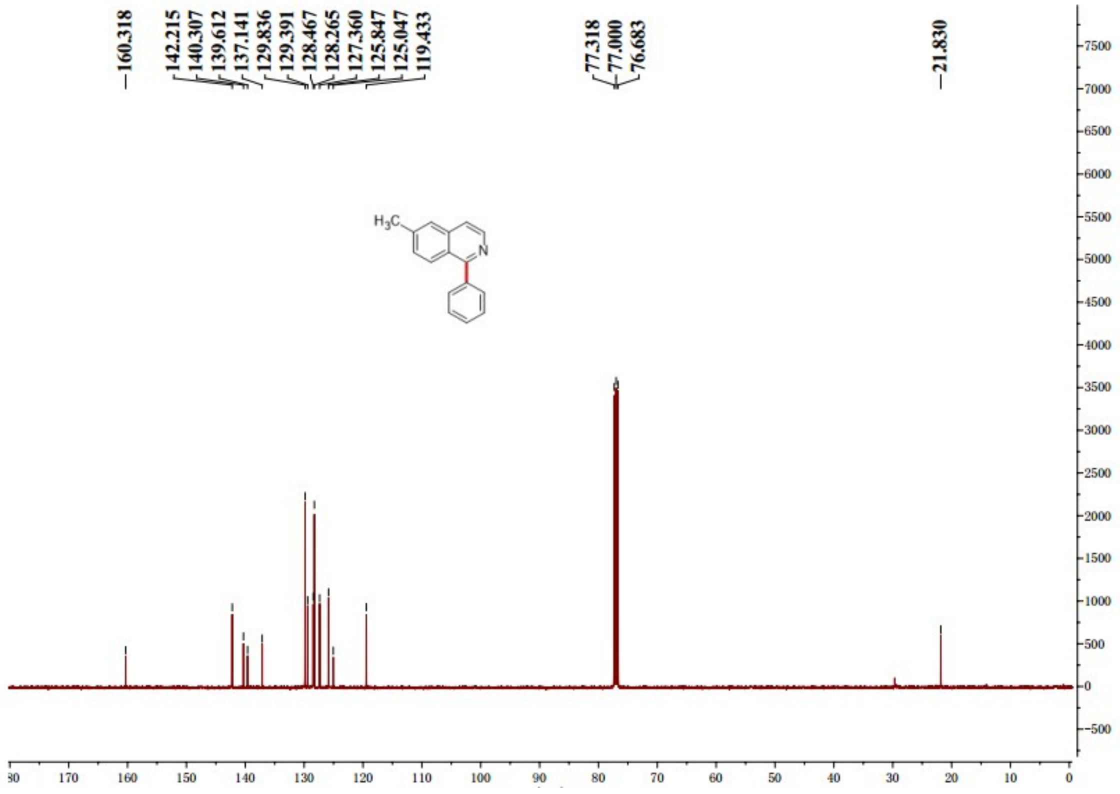
160.318
142.215
140.307
139.612
137.141
129.836
129.391
128.467
128.265
127.360
125.847
125.047
119.433
77.318
77.000
76.683
21.830
H3C
N

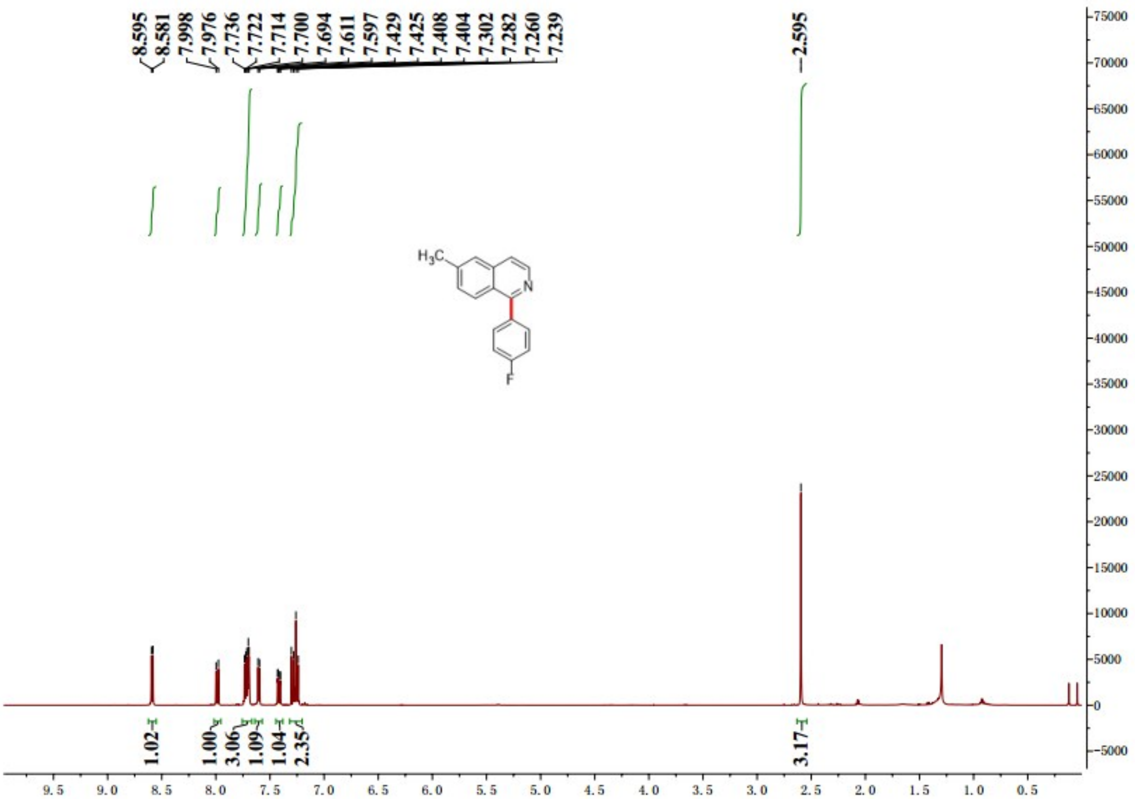
8.595
8.581
7.998
7.976
7.736
7.722
7.714
7.700
7.694
7.611
7.597
7.429
7.425
7.408
7.404
7.302
7.282
7.260
7.239
2.595
1.02
1.00
3.06
1.09
1.04
2.35
3.17
H3C
N
F

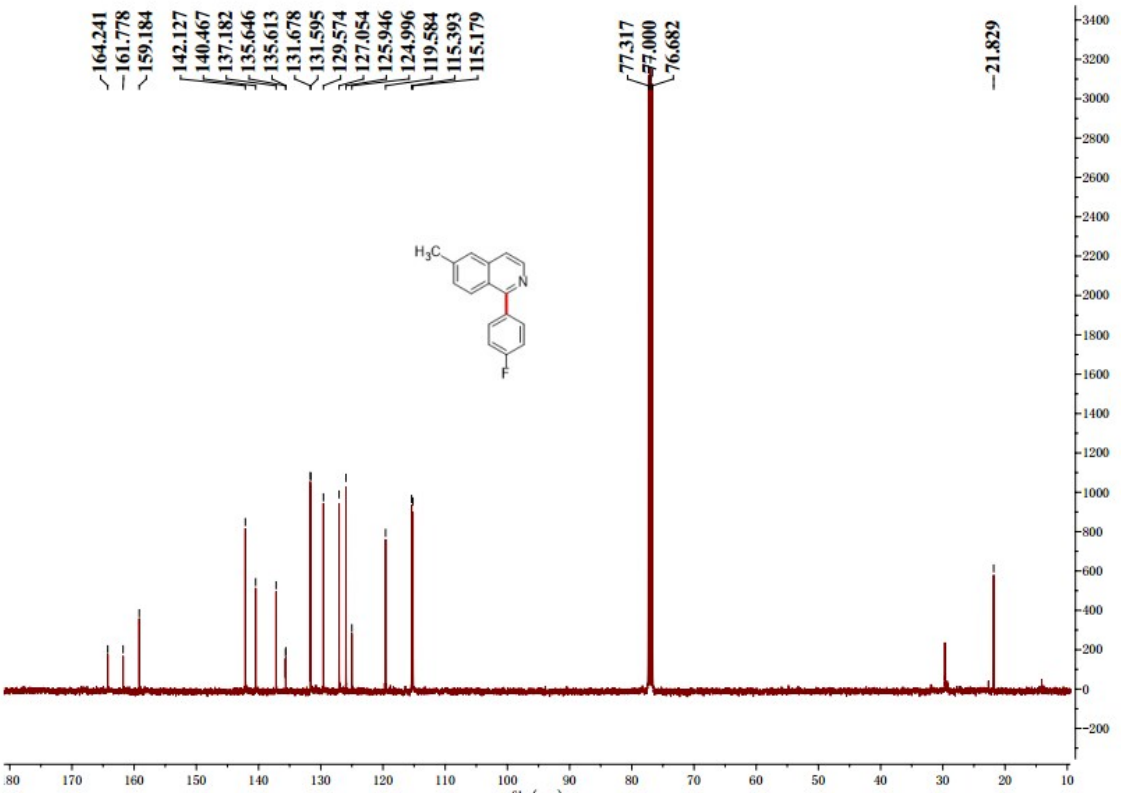
164.241
161.778
159.184
142.127
140.467
137.182
135.646
135.613
131.678
131.595
129.574
127.054
125.946
124.996
119.584
115.393
115.179
77.317
77.000
76.682
21.829
H3C
N
F

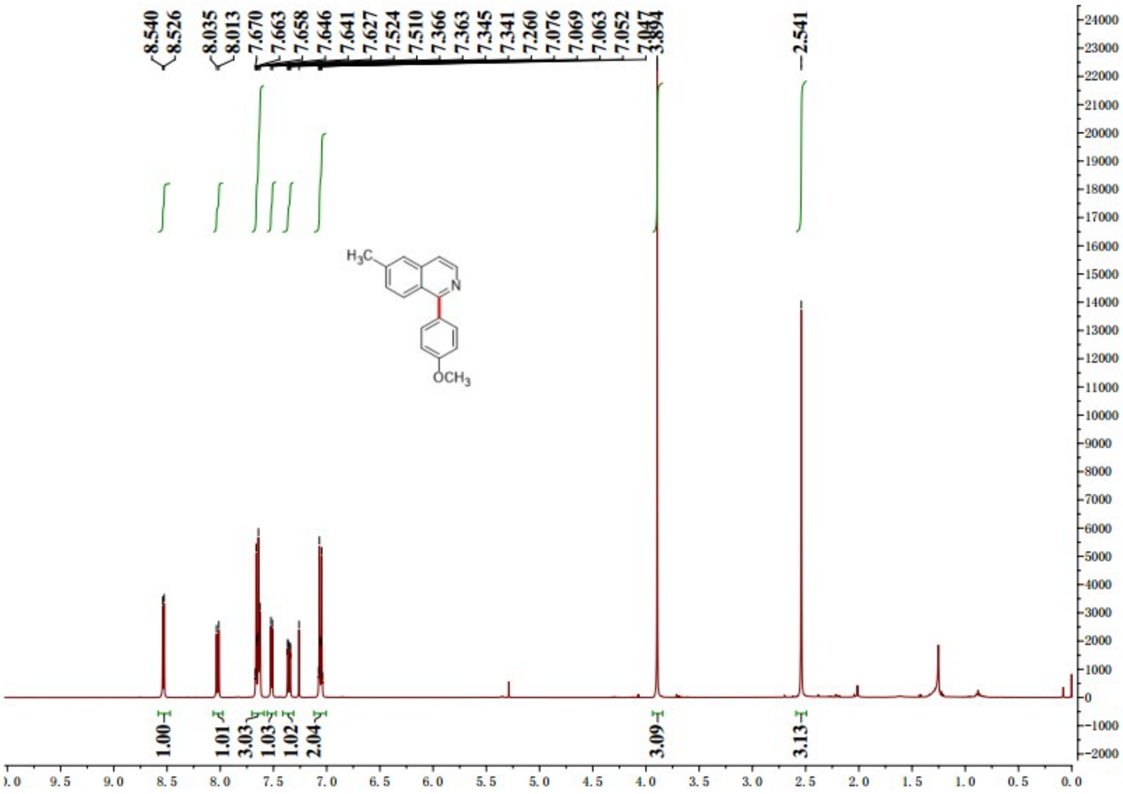
8.540
8.526
8.035
8.013
7.670
7.663
7.658
7.646
7.641
7.627
7.524
7.510
7.366
7.363
7.345
7.341
7.260
7.076
7.069
7.063
7.052
7.047
3.894
2.541
1.00
1.01
3.03
1.03
1.02
2.04
3.09
3.13
H3C
N
OCH3

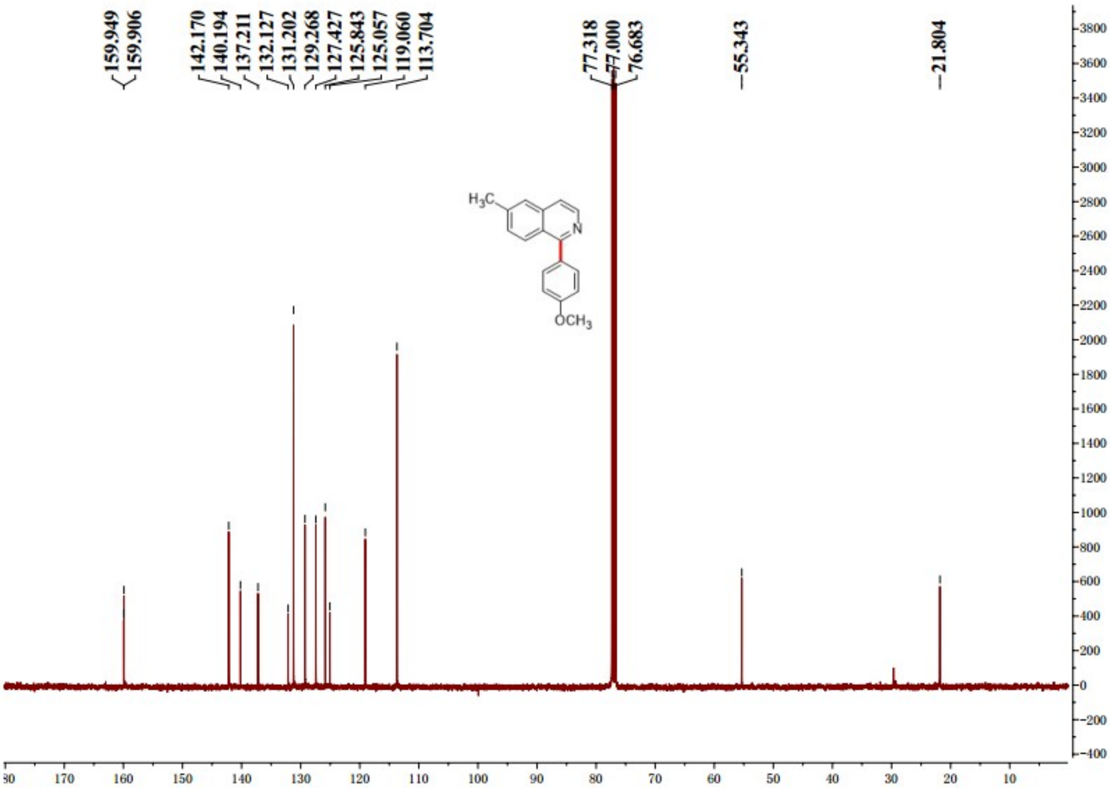
159.949
159.906
142.170
140.194
137.211
132.127
131.202
129.268
127.427
125.843
125.057
119.060
113.704
77.318
77.000
76.683
55.343
21.804
H3C
N
OCH3

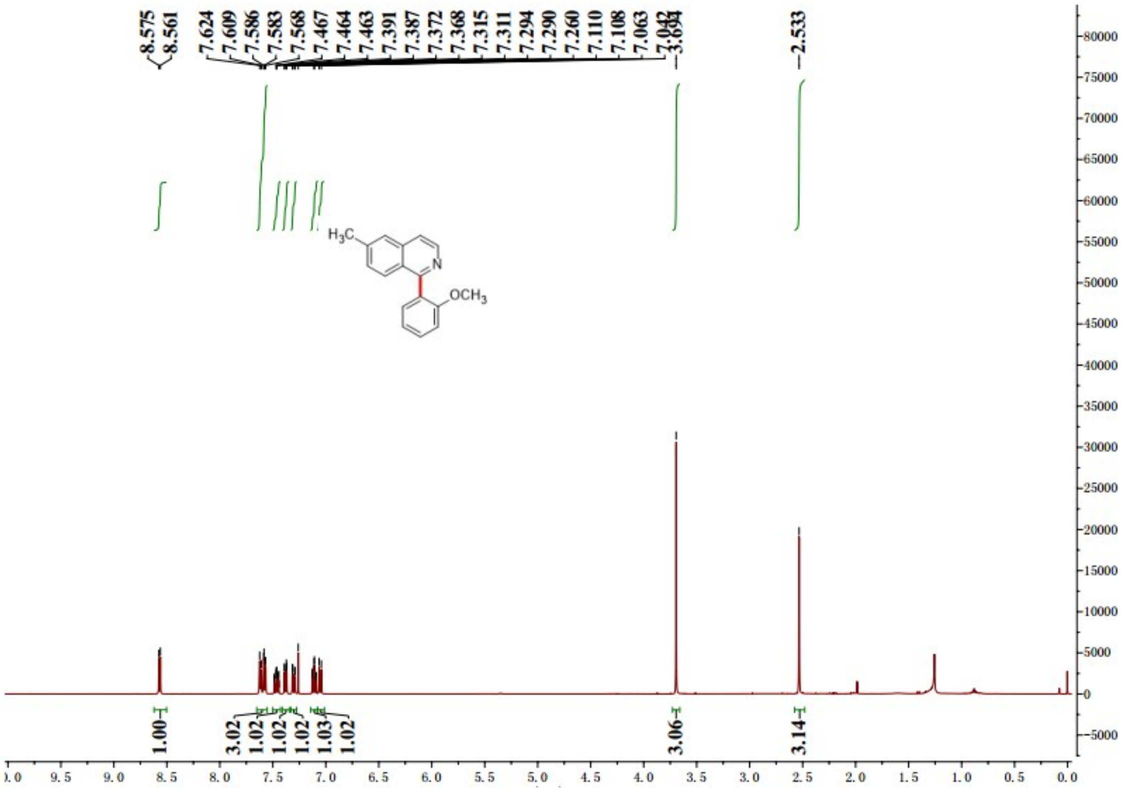

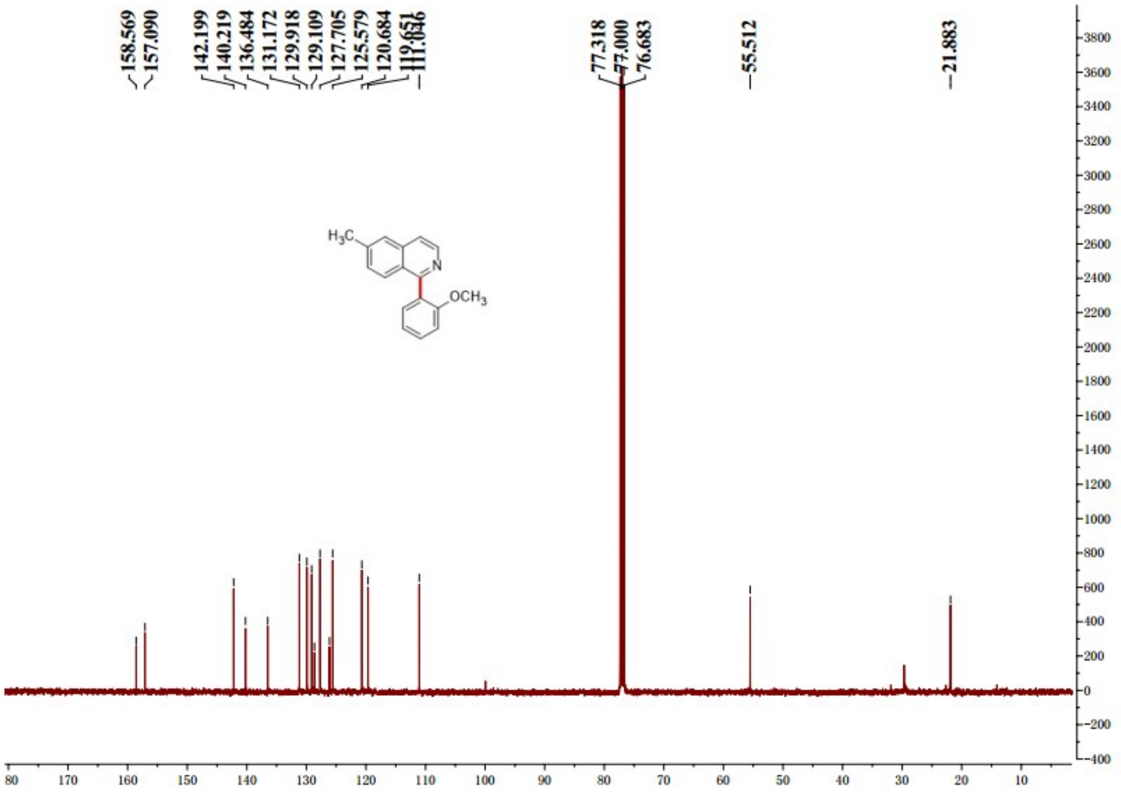

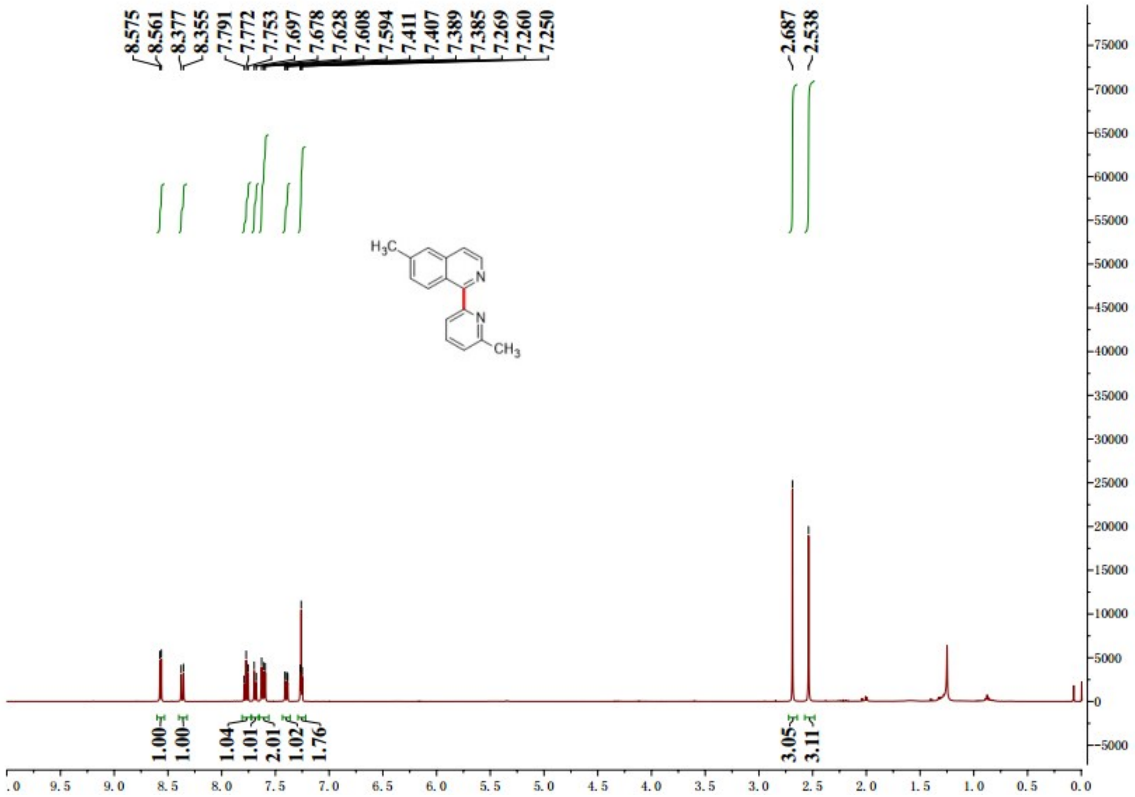
8.575
8.561
8.377
8.355
7.791
7.772
7.753
7.697
7.678
7.628
7.608
7.594
7.411
7.407
7.389
7.385
7.269
7.260
7.250
2.687
2.538
1.00
1.00
1.04
1.01
2.01
1.02
1.76
3.05
3.11

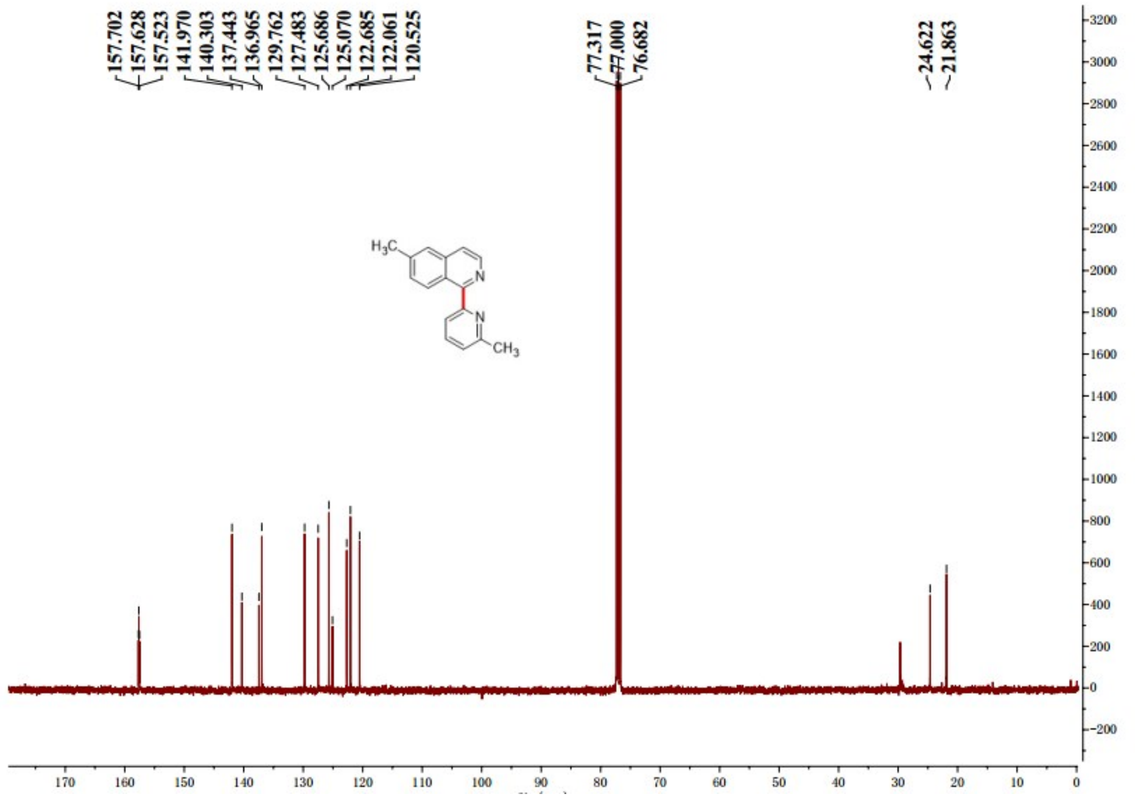
157.702
157.628
157.523
141.970
140.303
137.443
136.965
129.762
127.483
125.686
125.070
122.685
122.061
120.525
77.317
77.000
76.682
24.622
21.863

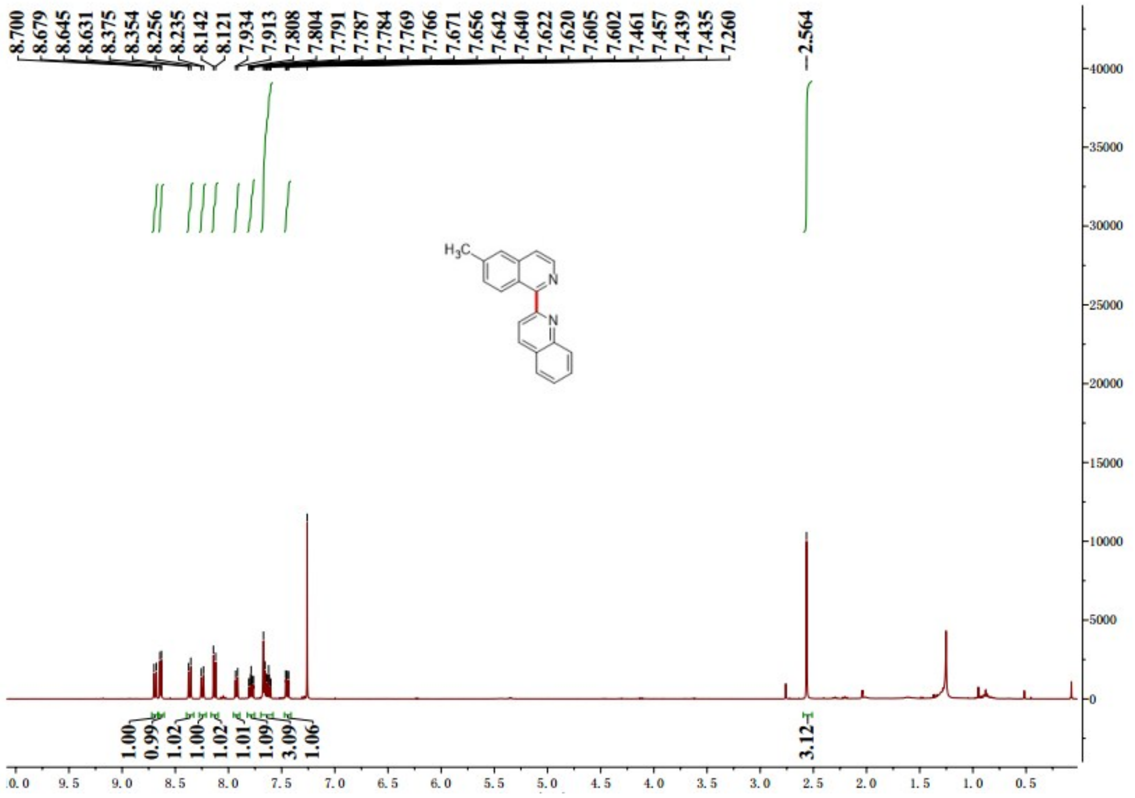

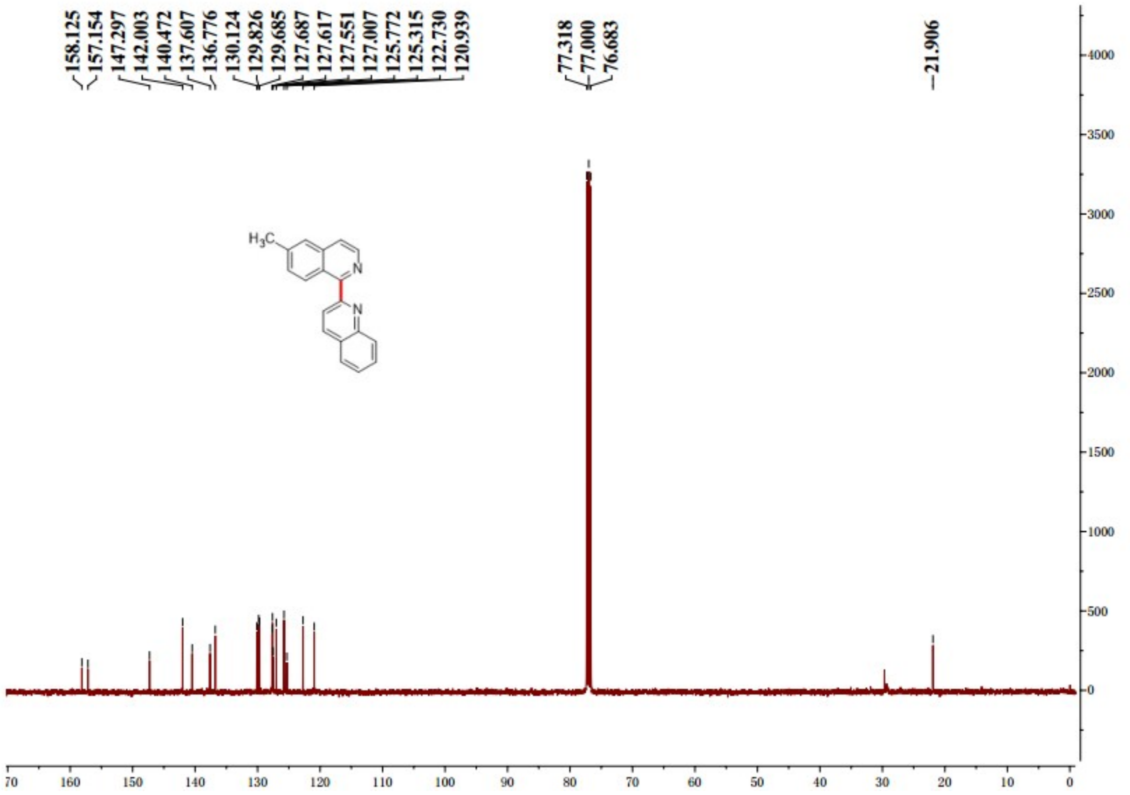

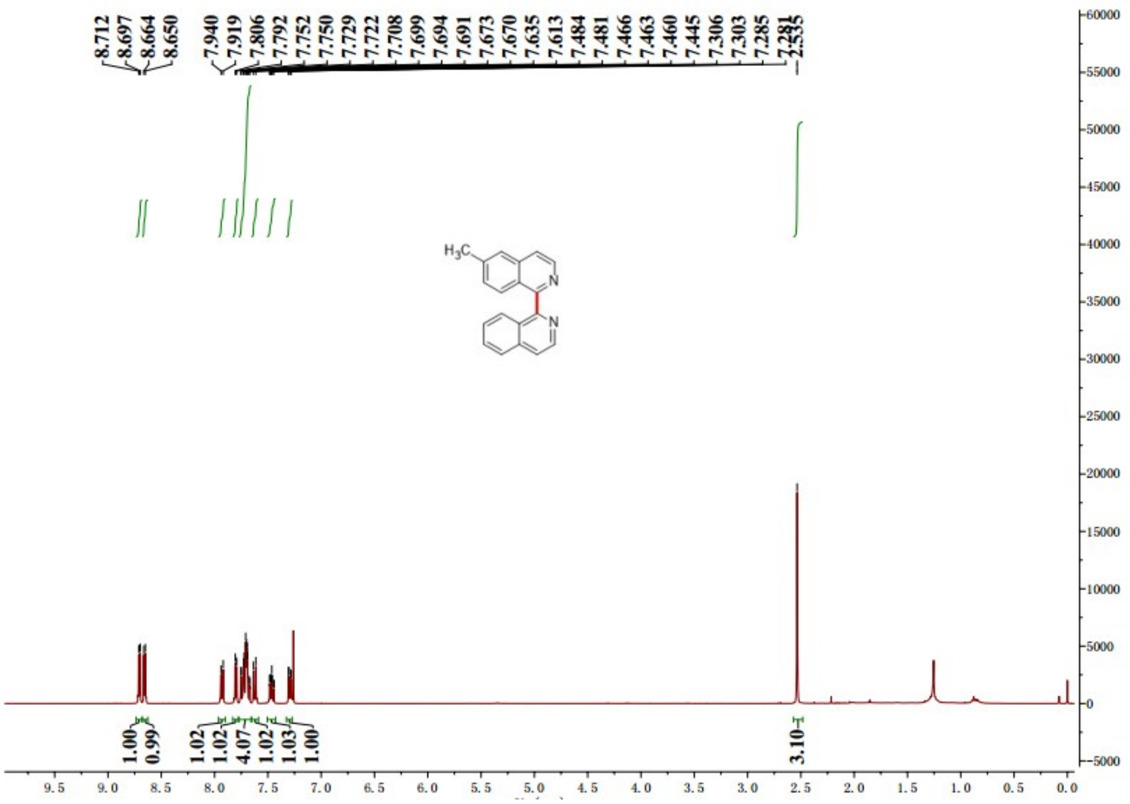
8.712
8.697
8.664
8.650
7.940
7.919
7.806
7.792
7.752
7.750
7.729
7.722
7.708
7.699
7.694
7.691
7.673
7.670
7.635
7.613
7.484
7.481
7.466
7.463
7.460
7.445
7.306
7.303
7.285
7.281
2.535
1.00
0.99
1.02
1.02
4.07
1.02
1.03
1.00
3.10

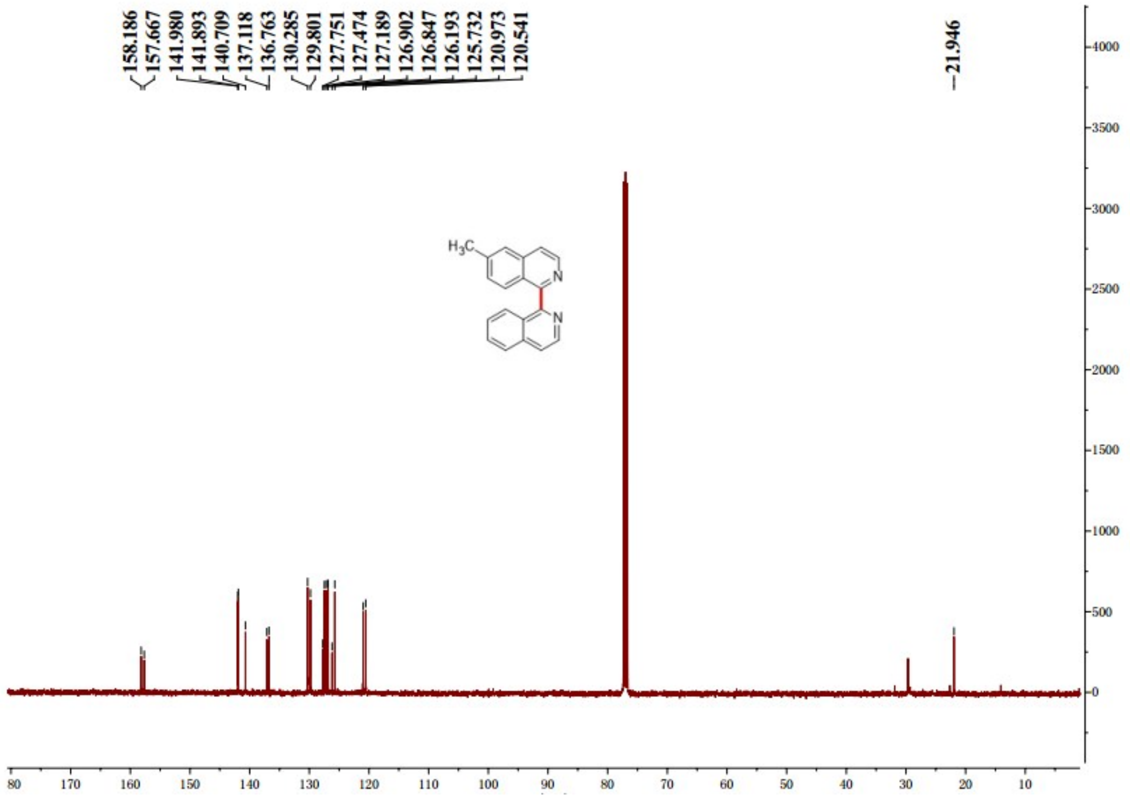
158.186
157.667
141.980
141.893
140.709
137.118
136.763
130.285
129.801
127.751
127.474
127.189
126.902
126.847
126.193
125.732
120.973
120.541
21.946

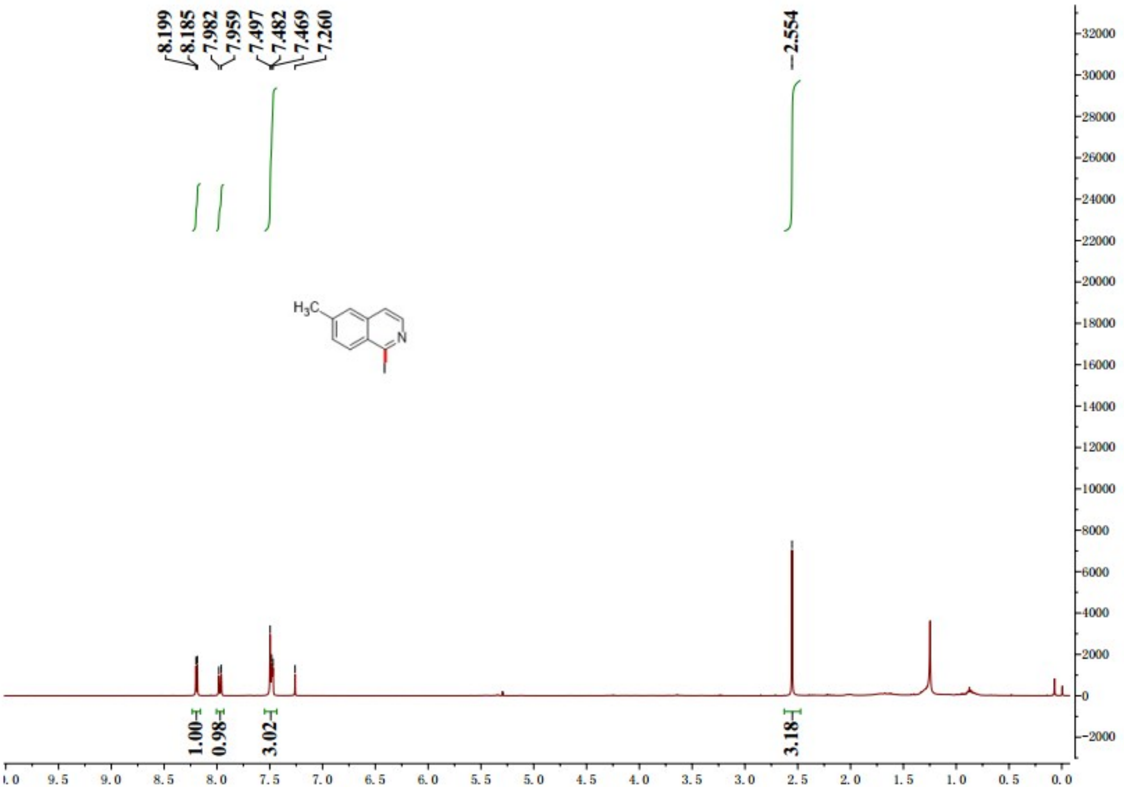
8.199
8.185
7.982
7.959
7.497
7.482
7.469
7.260
2.554
1.00
0.98
3.02
3.18
H3C
N

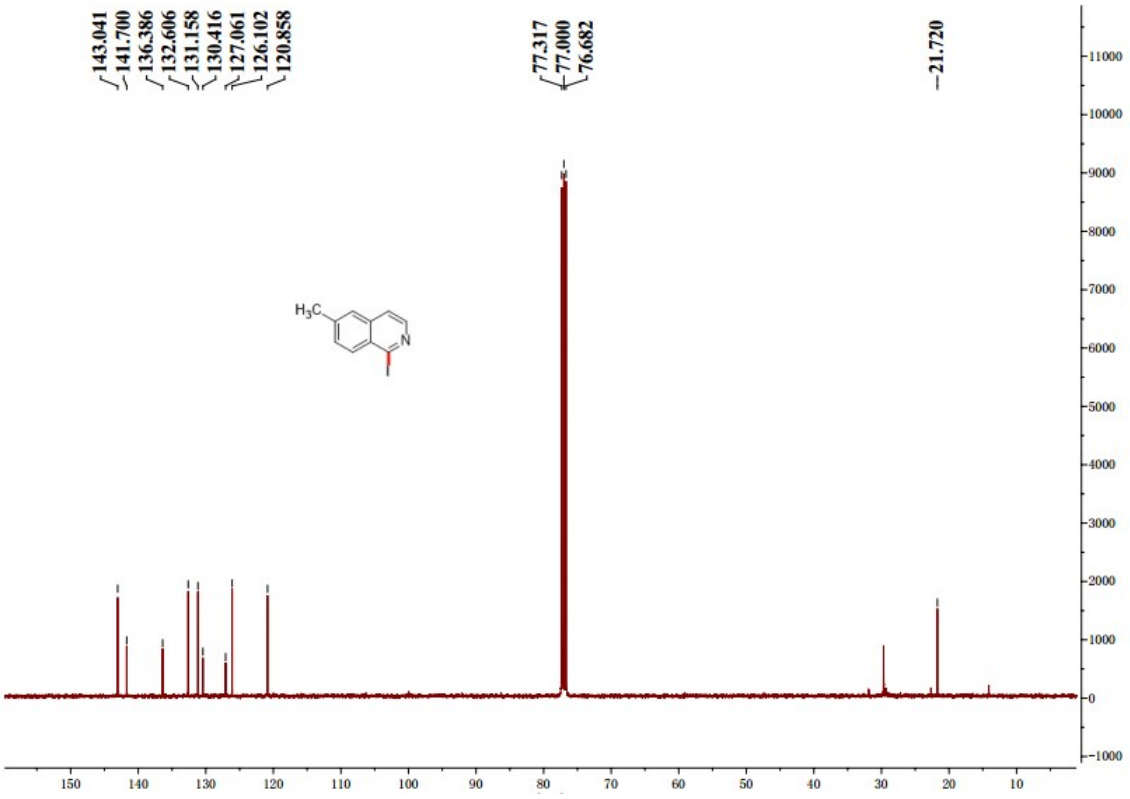
143.041
141.700
136.386
132.606
131.158
130.416
127.061
126.102
120.858
77.317
77.000
76.682
21.720
H3C
N

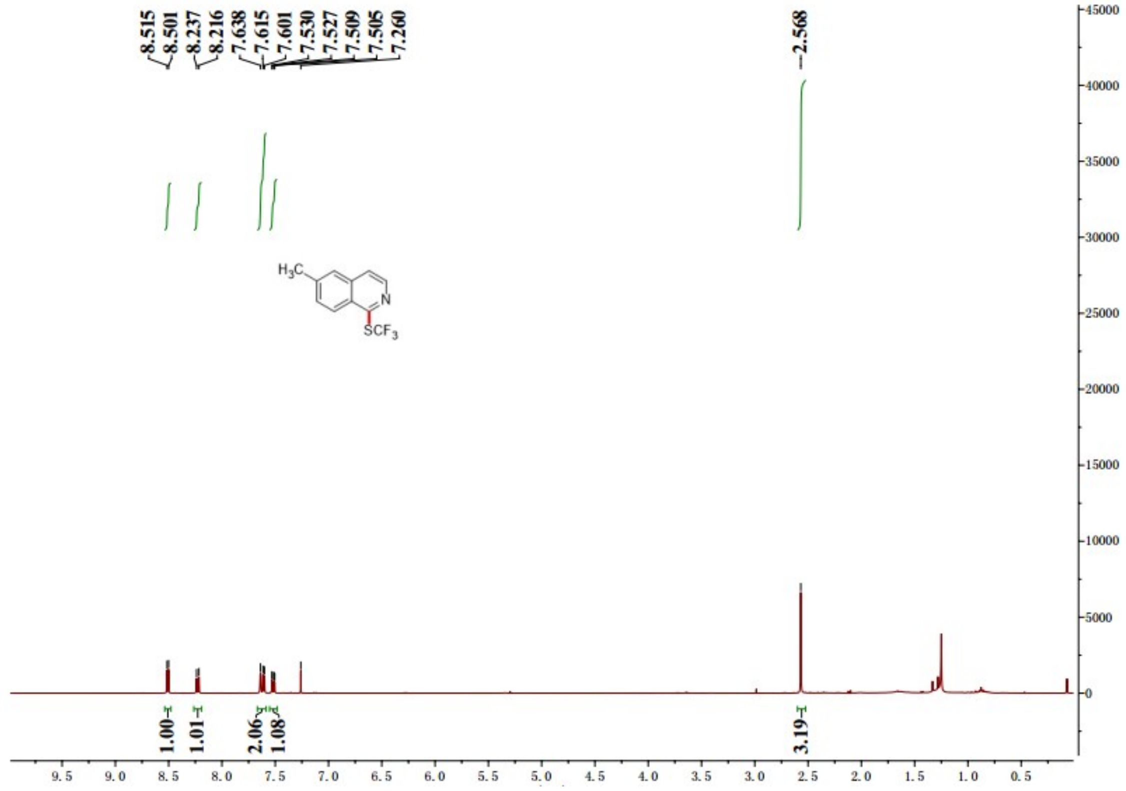
8.515
8.501
8.237
8.216
7.638
7.615
7.601
7.530
7.527
7.509
7.505
7.260
2.568
1.00
1.01
2.06
1.08
3.19

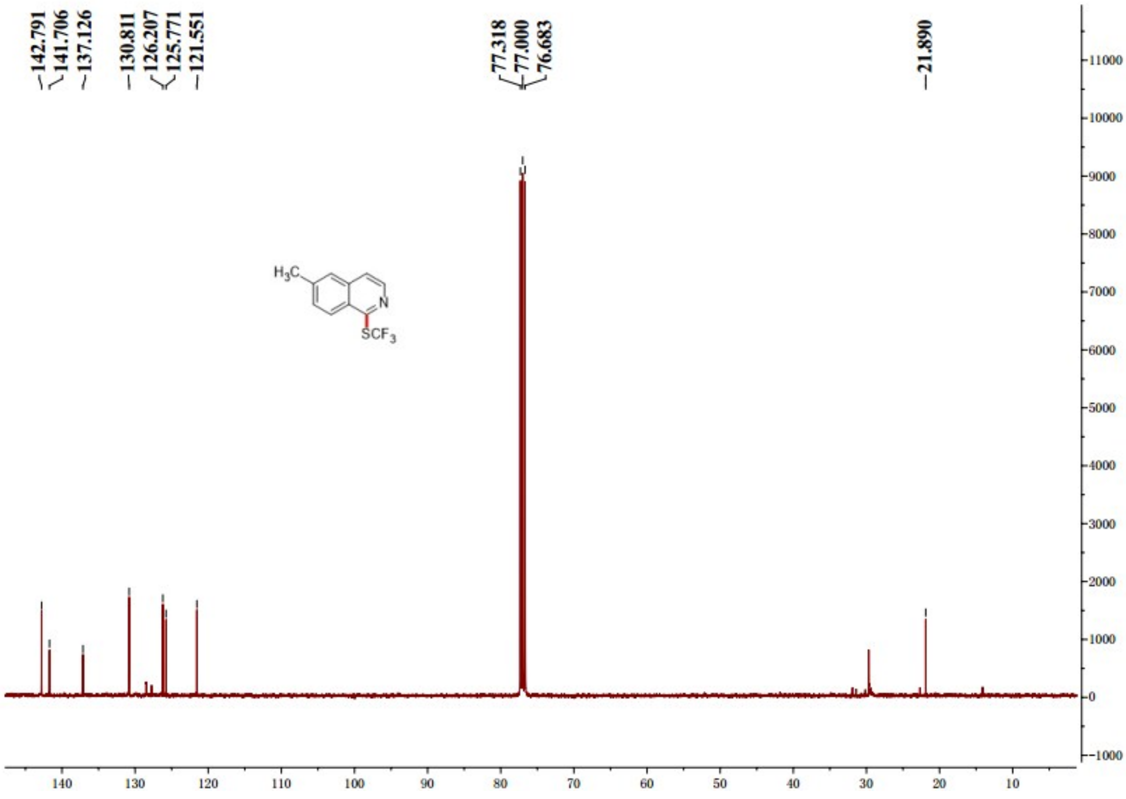
142.791
141.706
137.126
130.811
126.207
125.771
121.551
77.318
77.000
76.683
21.890

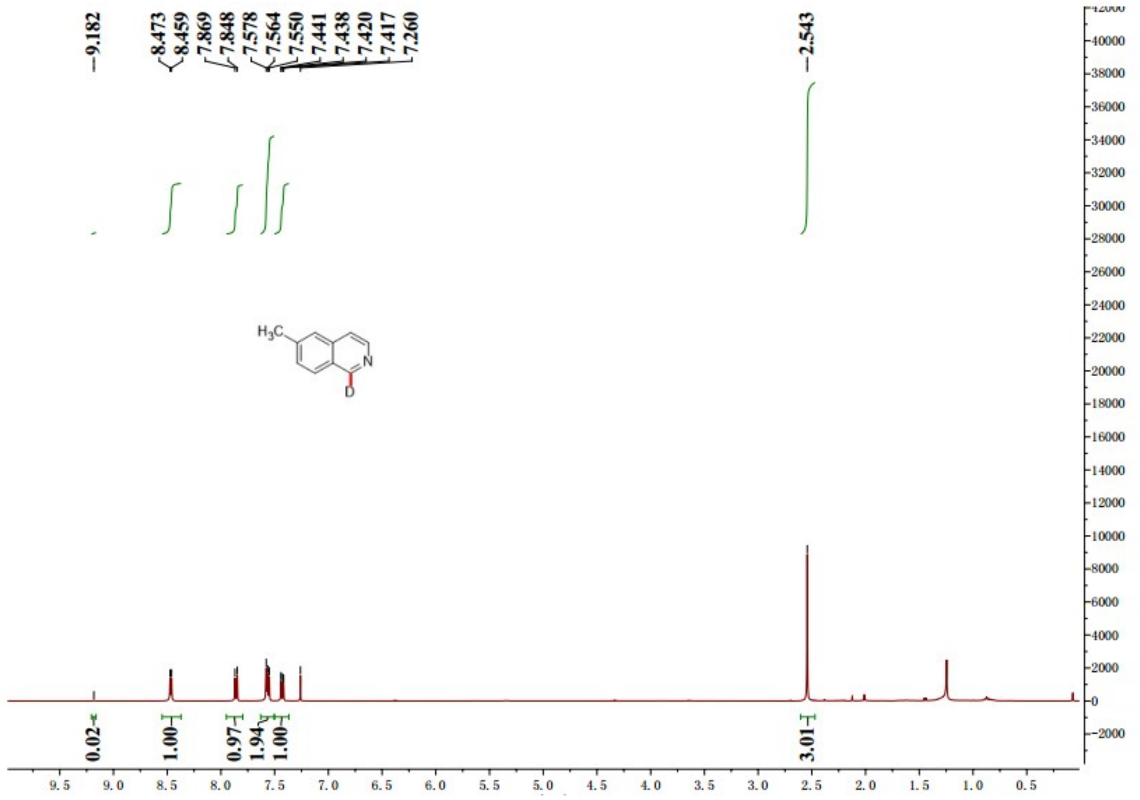
9.182
8.473
8.459
7.869
7.848
7.578
7.564
7.550
7.441
7.438
7.420
7.417
7.260
2.543
H3C
N
D
0.02
1.00
0.97
1.94
1.00
3.01

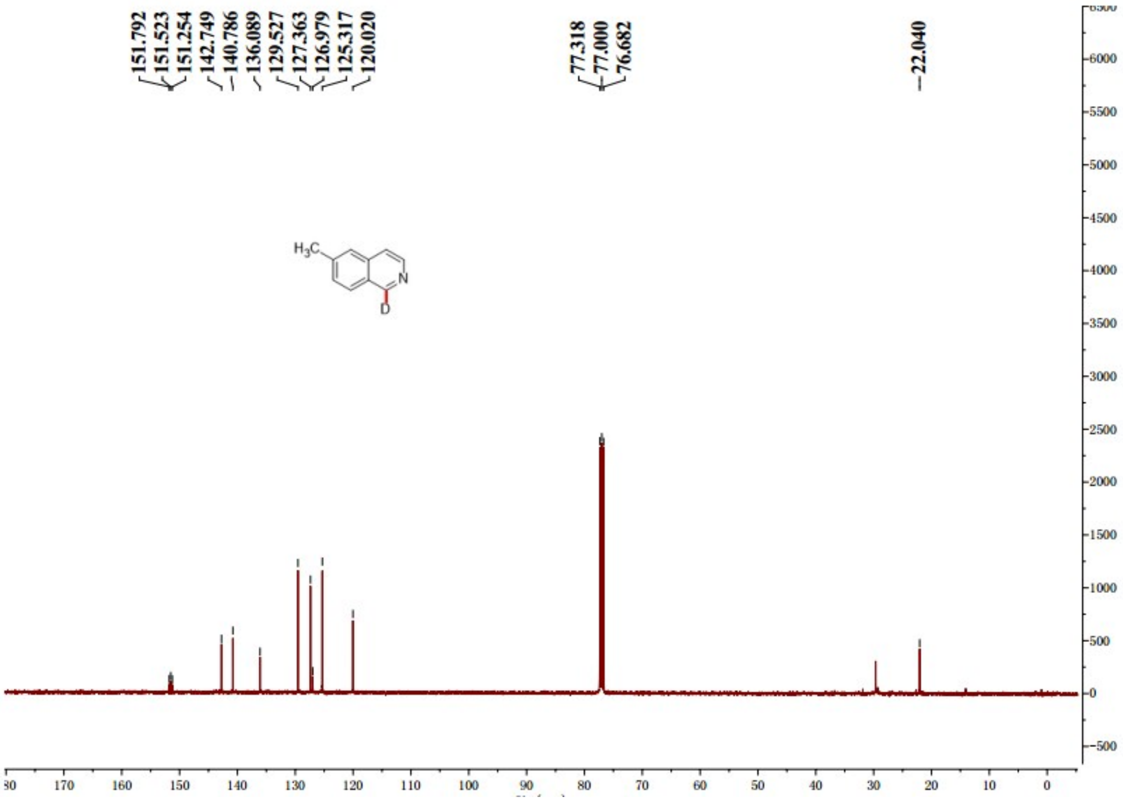
151.792
151.523
151.254
142.749
140.786
136.089
129.527
127.363
126.979
125.317
120.020
77.318
77.000
76.682
22.040
H3C
N
D

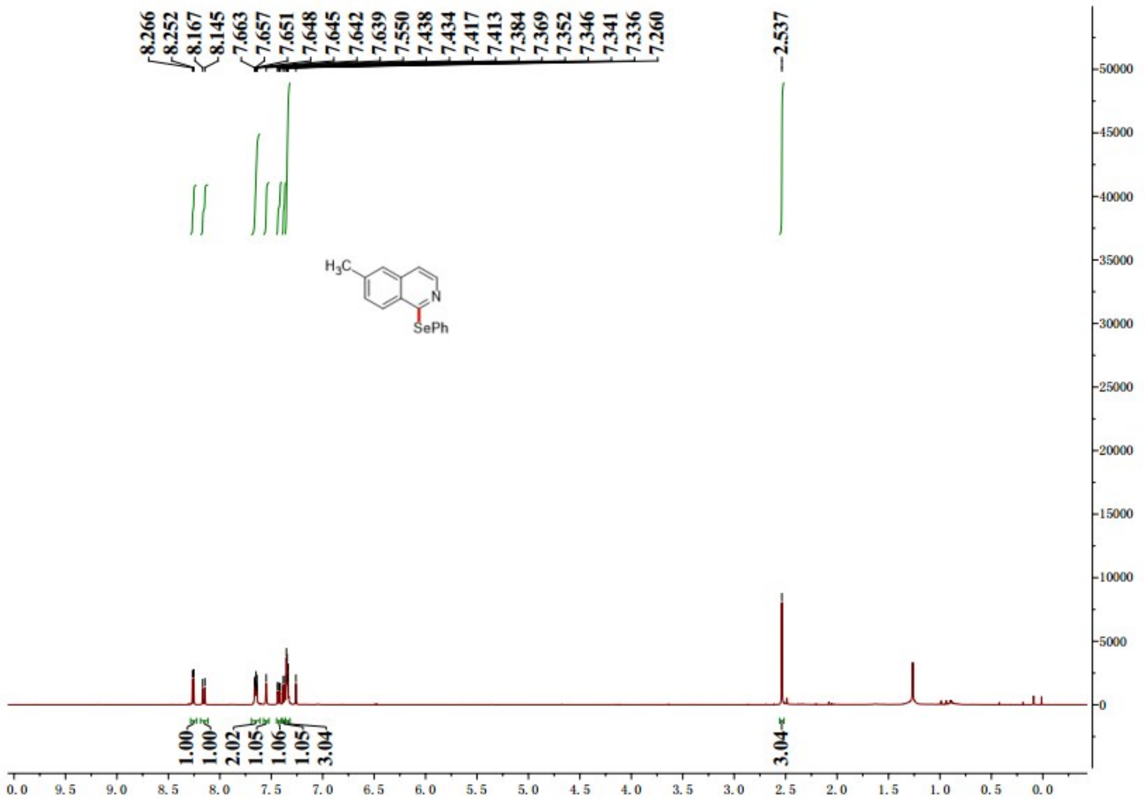

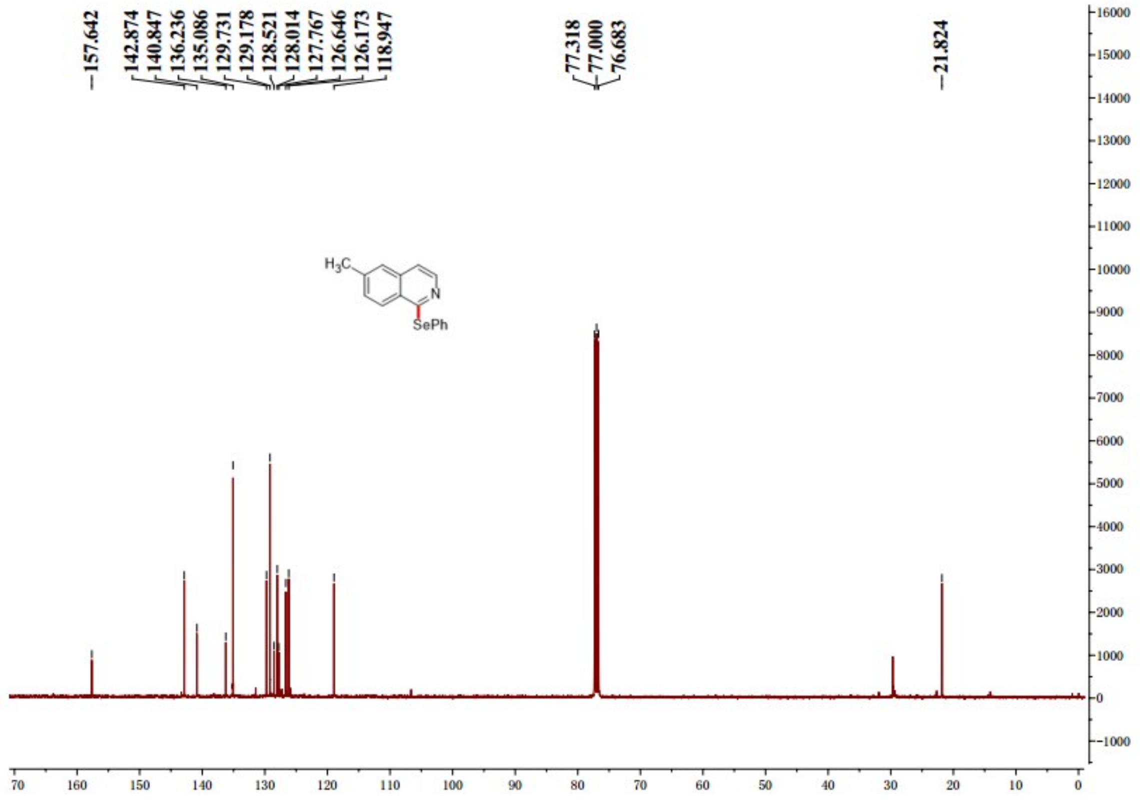

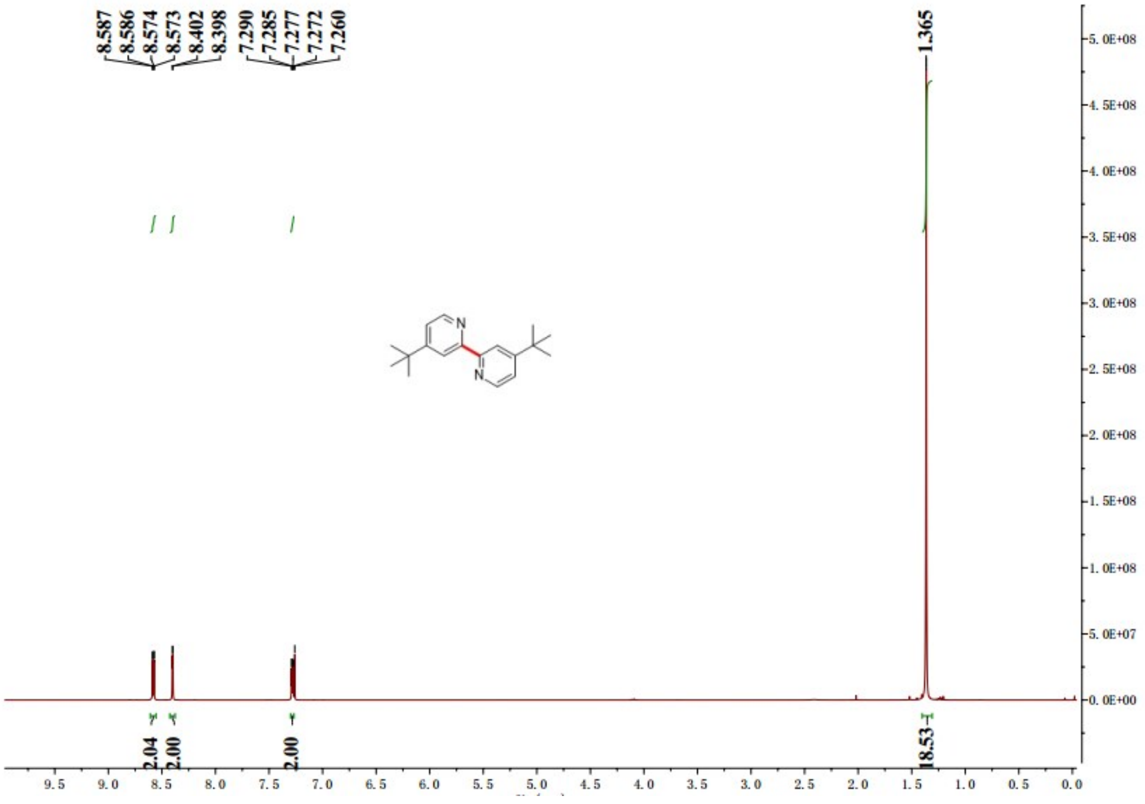
8.587
8.586
8.574
8.573
8.402
8.398
7.290
7.285
7.277
7.272
7.260
1.365
2.04
2.00
2.00
18.53
N
N

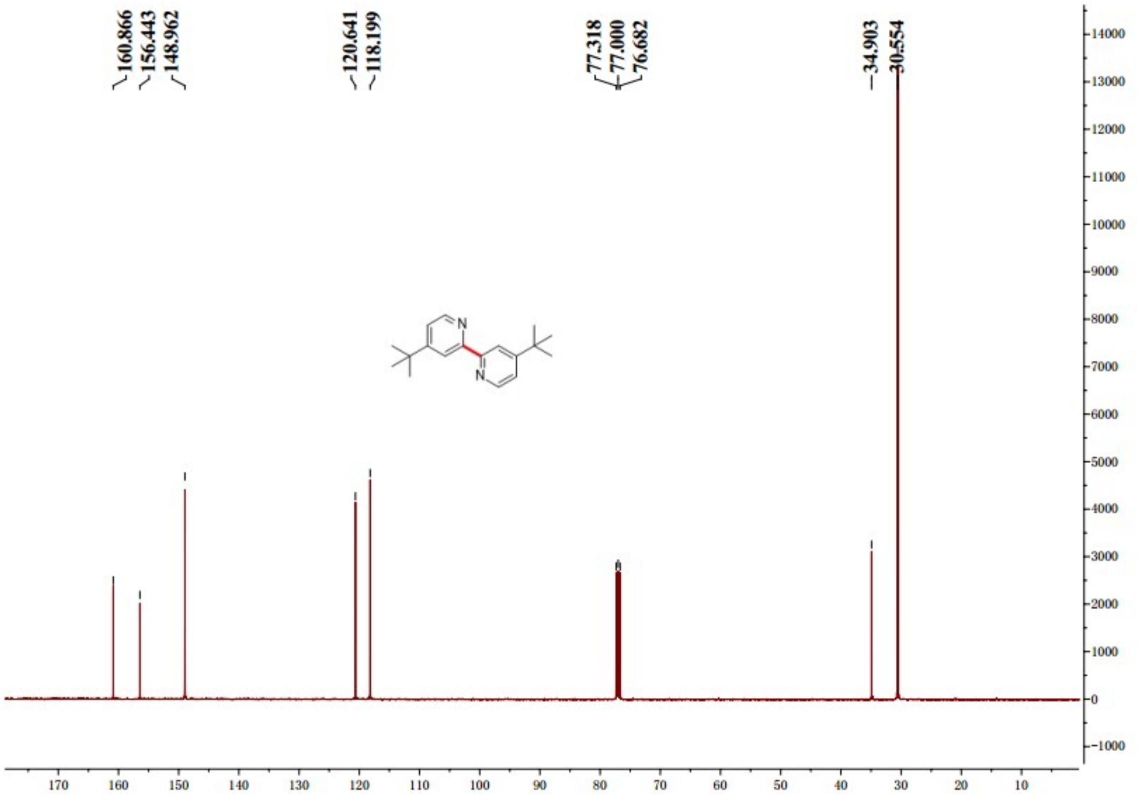
160.866
156.443
148.962
120.641
118.199
77.318
77.000
76.682
34.903
30.554
N
N

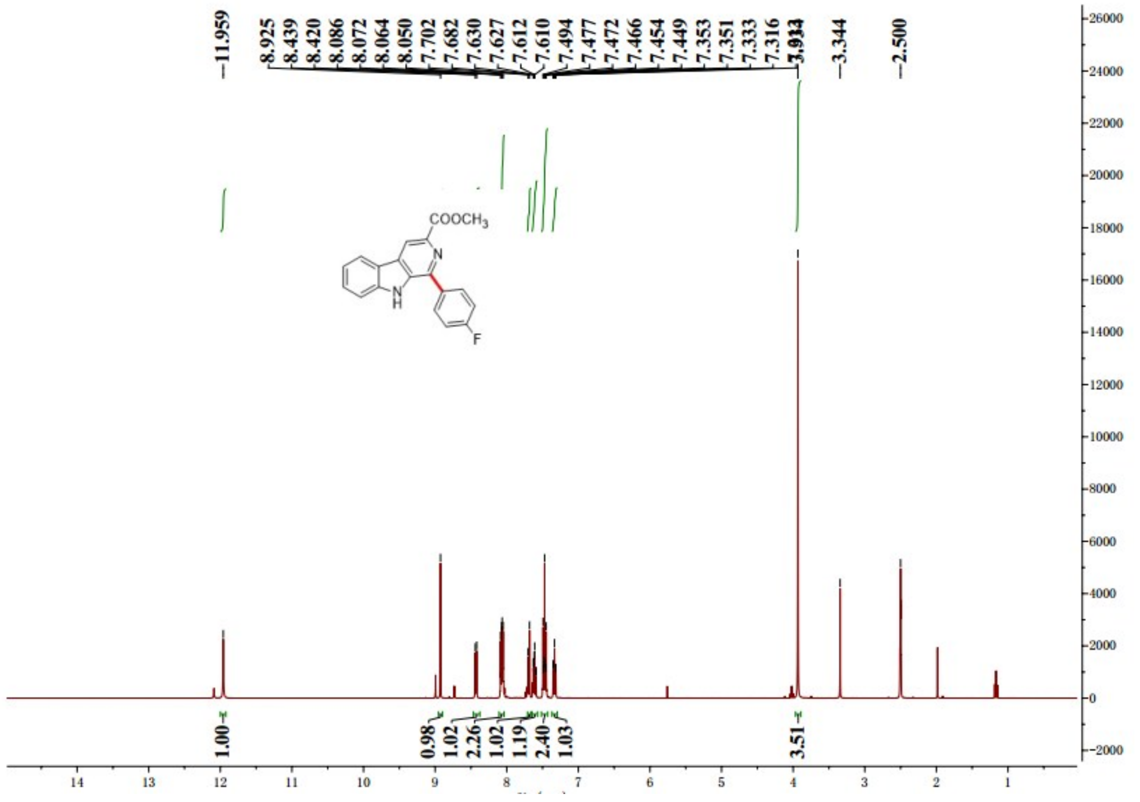
11.959
8.925
8.439
8.420
8.086
8.072
8.064
8.050
7.702
7.682
7.630
7.627
7.612
7.610
7.494
7.477
7.472
7.466
7.454
7.449
7.353
7.351
7.333
7.316
3.344
2.500
COOCH3
N
H
F
1.00
0.98
1.02
2.26
1.02
1.19
2.40
1.03
3.51

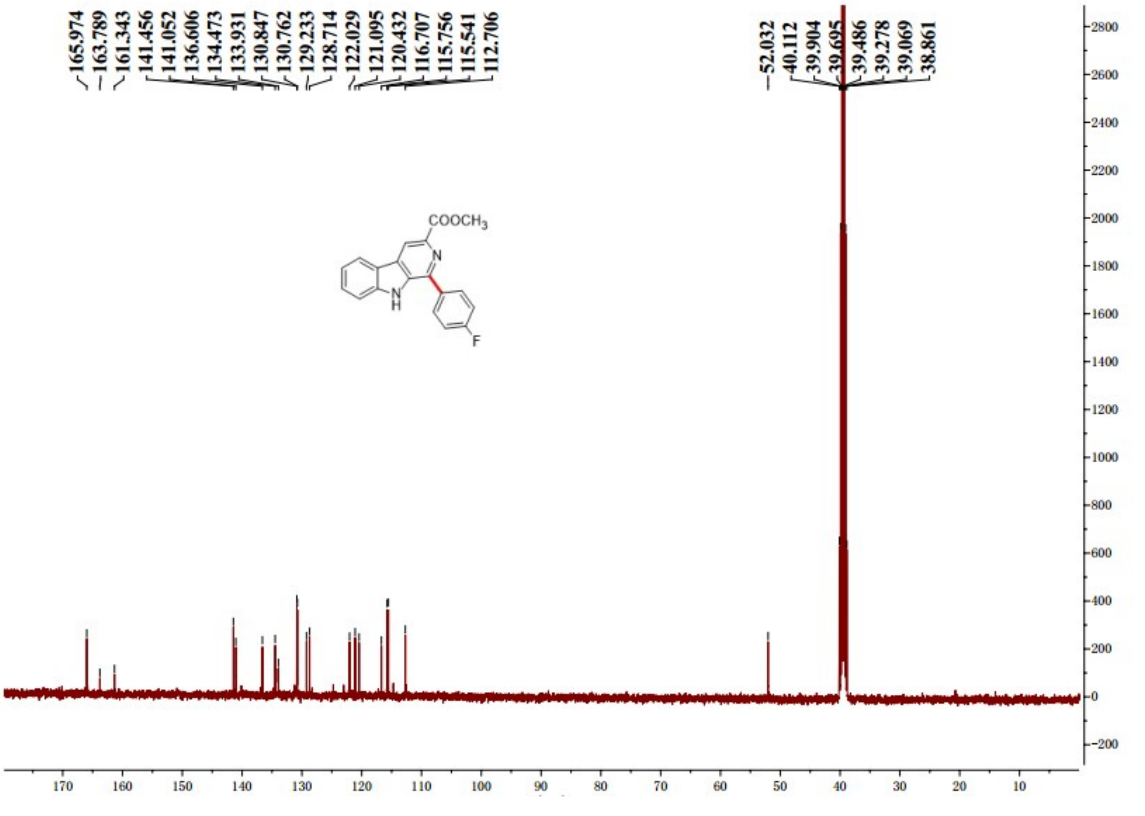
165.974
163.789
161.343
141.456
141.052
136.606
134.473
133.931
130.847
130.762
129.233
128.714
122.029
121.095
120.432
116.707
115.756
115.541
112.706
52.032
40.112
39.904
39.695
39.486
39.278
39.069
38.861
COOCH3
N
H
F

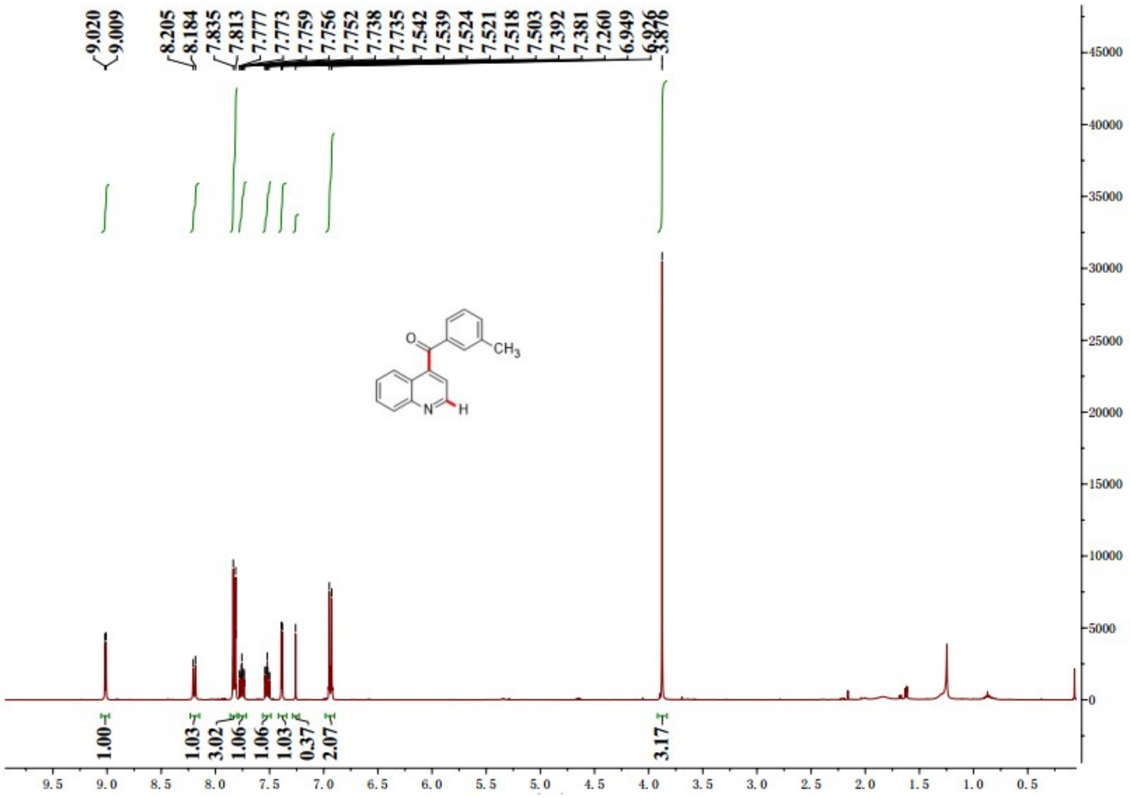

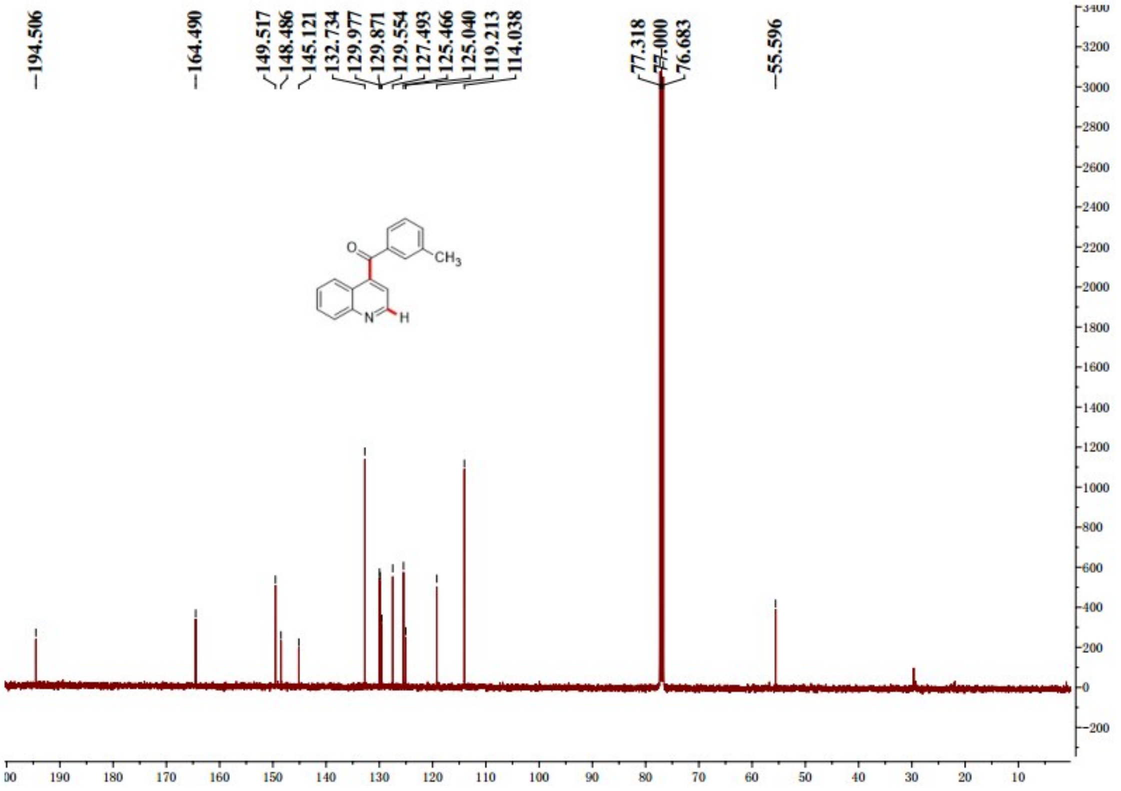

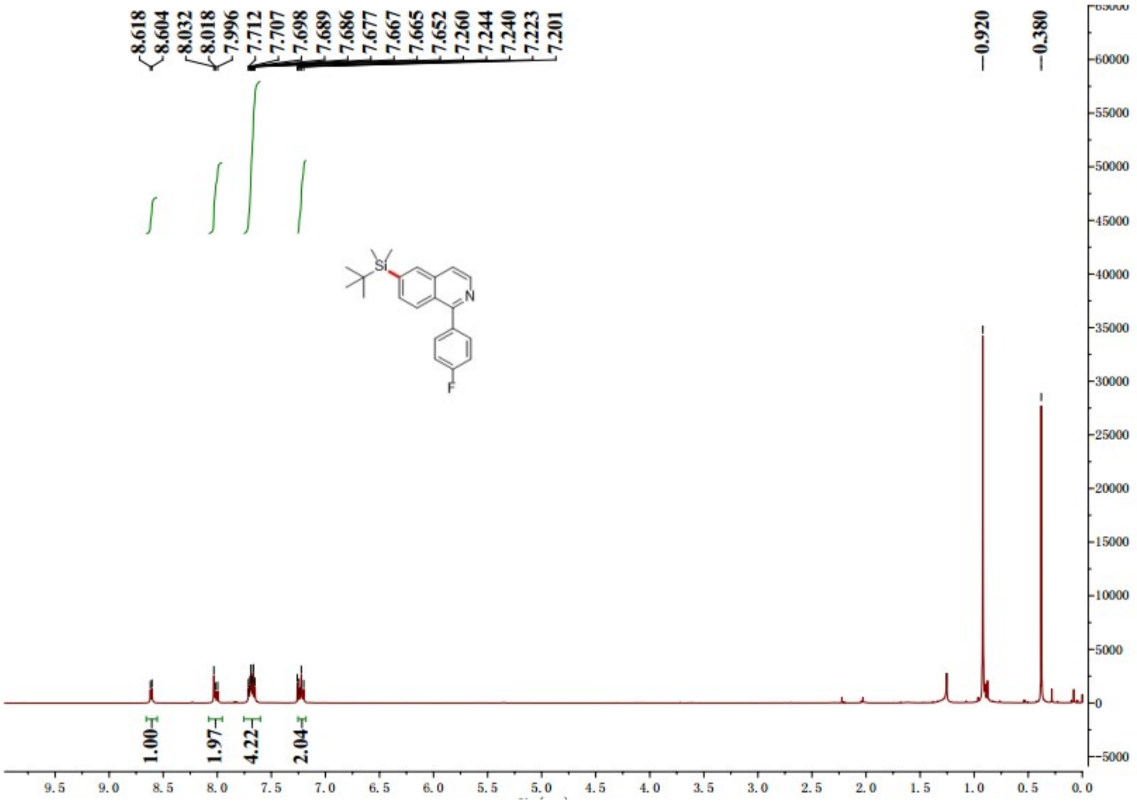
8.618
8.604
8.032
8.018
7.996
7.712
7.707
7.698
7.689
7.686
7.677
7.667
7.665
7.652
7.260
7.244
7.240
7.223
7.201
0.920
0.380
1.00
1.97
4.22
2.04
Si
N
F

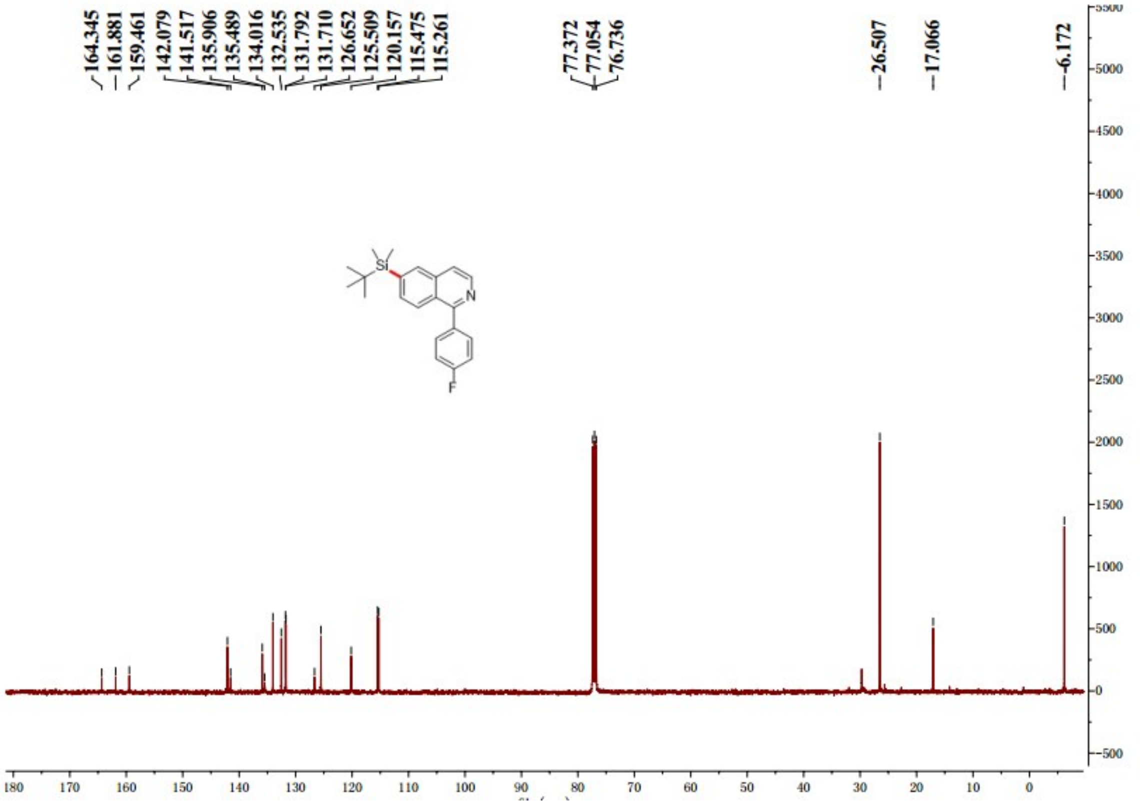
164.345
161.881
159.461
142.079
141.517
135.906
135.489
134.016
132.535
131.792
131.710
126.652
125.509
120.157
115.475
115.261
77.372
77.054
76.736
26.507
17.066
-6.172
Si
N
F

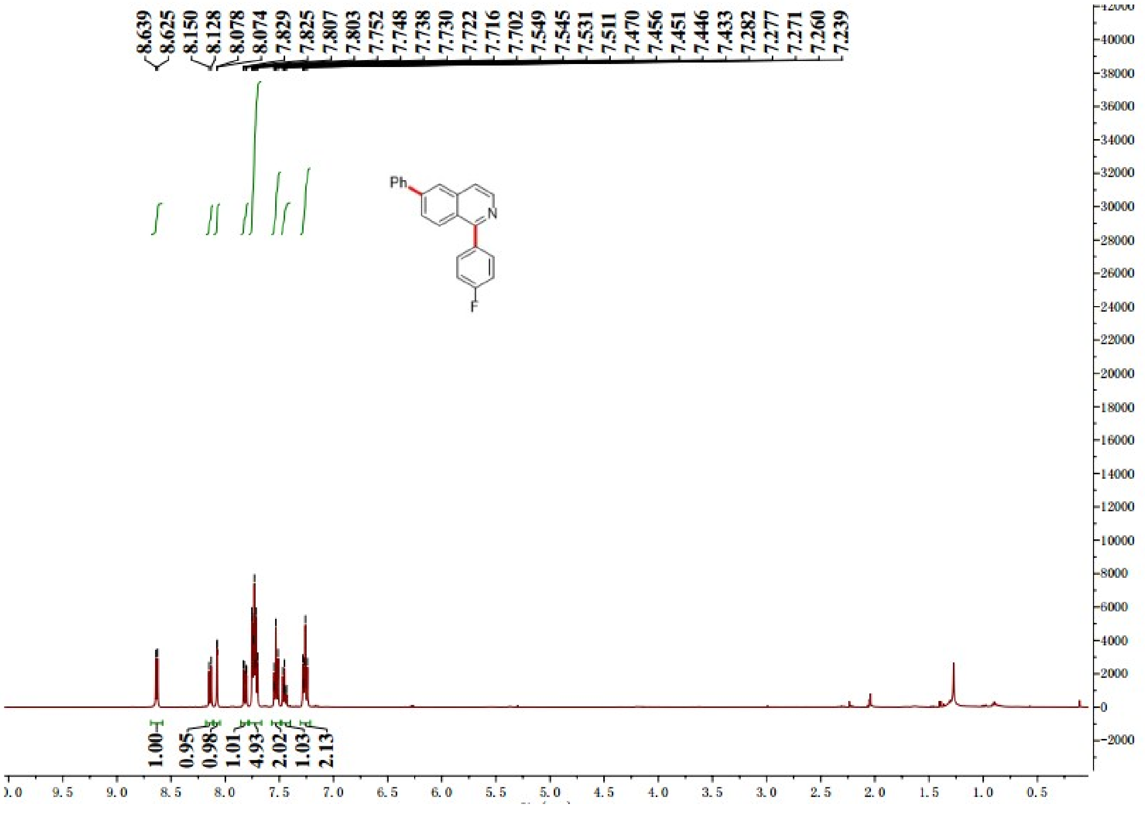
8.639
8.625
8.150
8.128
8.078
8.074
7.829
7.825
7.807
7.803
7.752
7.748
7.738
7.730
7.722
7.716
7.702
7.549
7.545
7.531
7.511
7.470
7.456
7.451
7.446
7.433
7.282
7.277
7.271
7.260
7.239
Ph
N
F
1.00
0.95
0.98
1.01
4.93
2.02
1.03
2.13

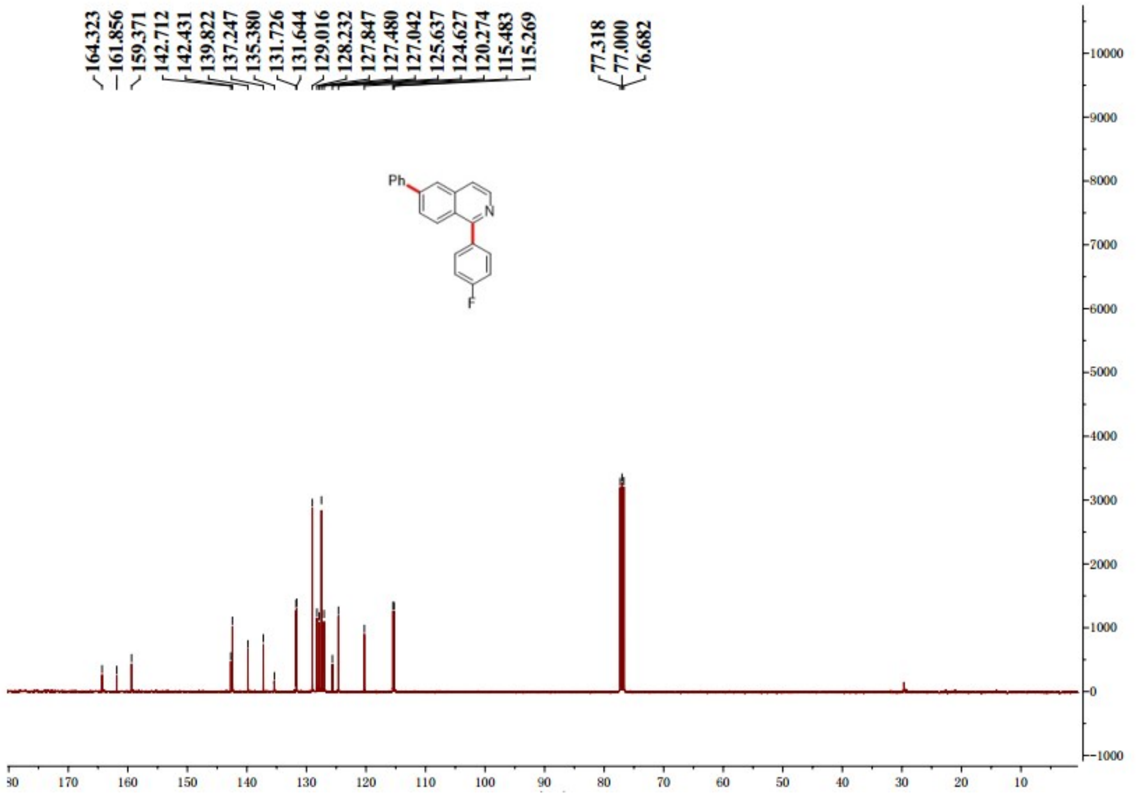
164.323
161.856
159.371
142.712
142.431
139.822
137.247
135.380
131.726
131.644
129.016
128.232
127.847
127.480
127.042
125.637
124.627
120.274
115.483
115.269
77.318
77.000
76.682
Ph
N
F

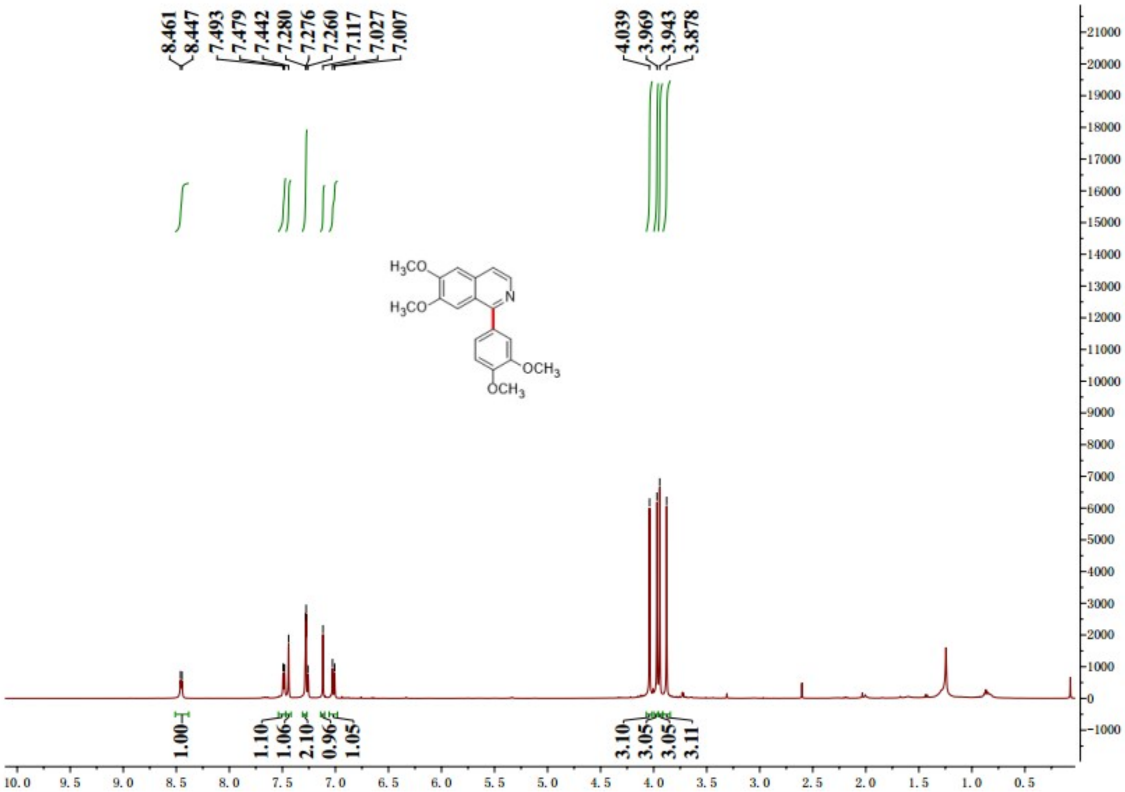
8.461
8.447
7.493
7.479
7.442
7.280
7.276
7.260
7.117
7.027
7.007
4.039
3.969
3.943
3.878
H3CO
H3CO
N
OCH3
OCH3
1.00
1.10
1.06
2.10
0.96
1.05
3.10
3.05
3.05
3.11

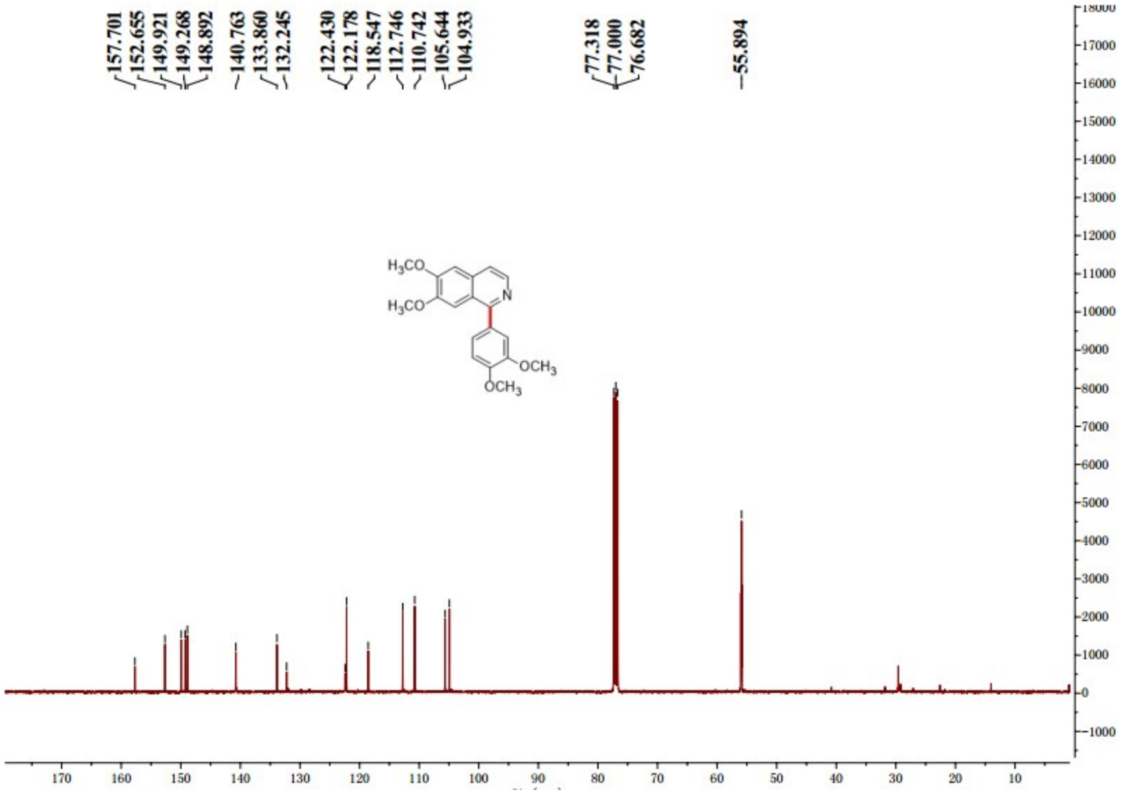
157.701
152.655
149.921
149.268
148.892
140.763
133.860
132.245
122.430
122.178
118.547
112.746
110.742
105.644
104.933
77.318
77.000
76.682
55.894
H3CO
H3CO
N
OCH3
OCH3

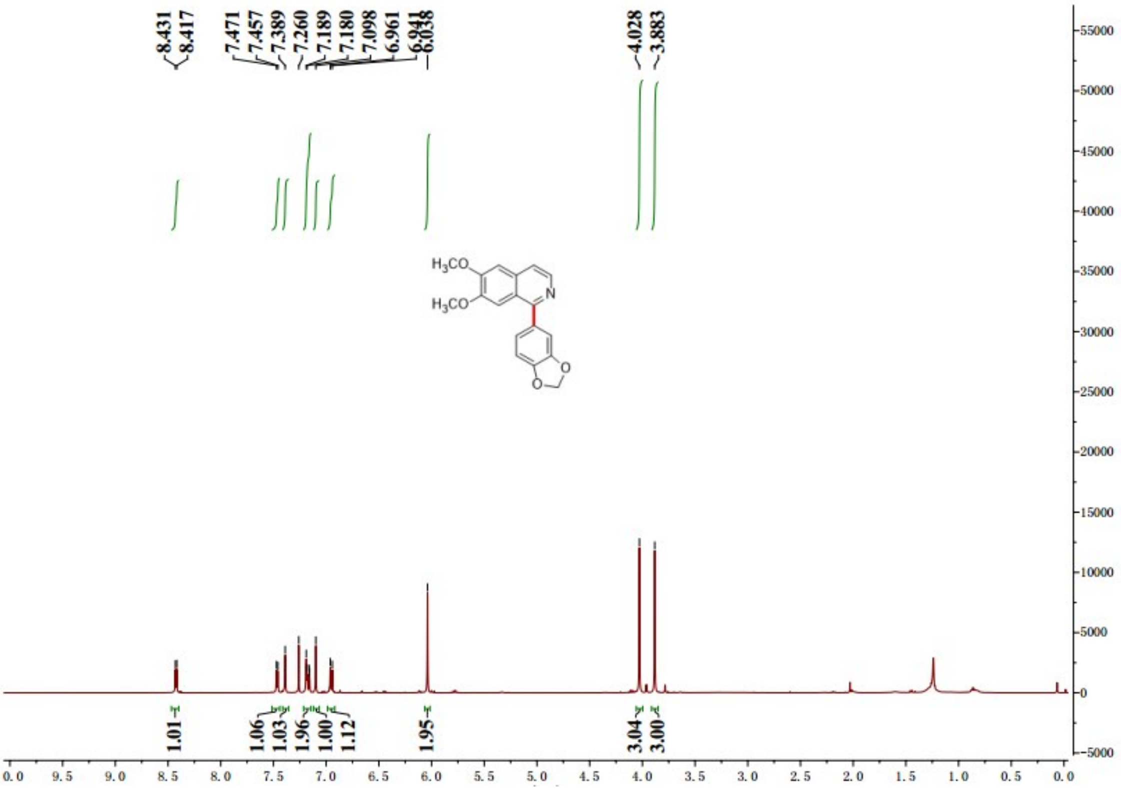
8.431
8.417
7.471
7.457
7.389
7.260
7.189
7.180
7.098
6.961
6.941
6.038
4.028
3.883
H3CO
H3CO
N
O
O
1.01
1.06
1.03
1.96
1.00
1.12
1.95
3.04
3.00

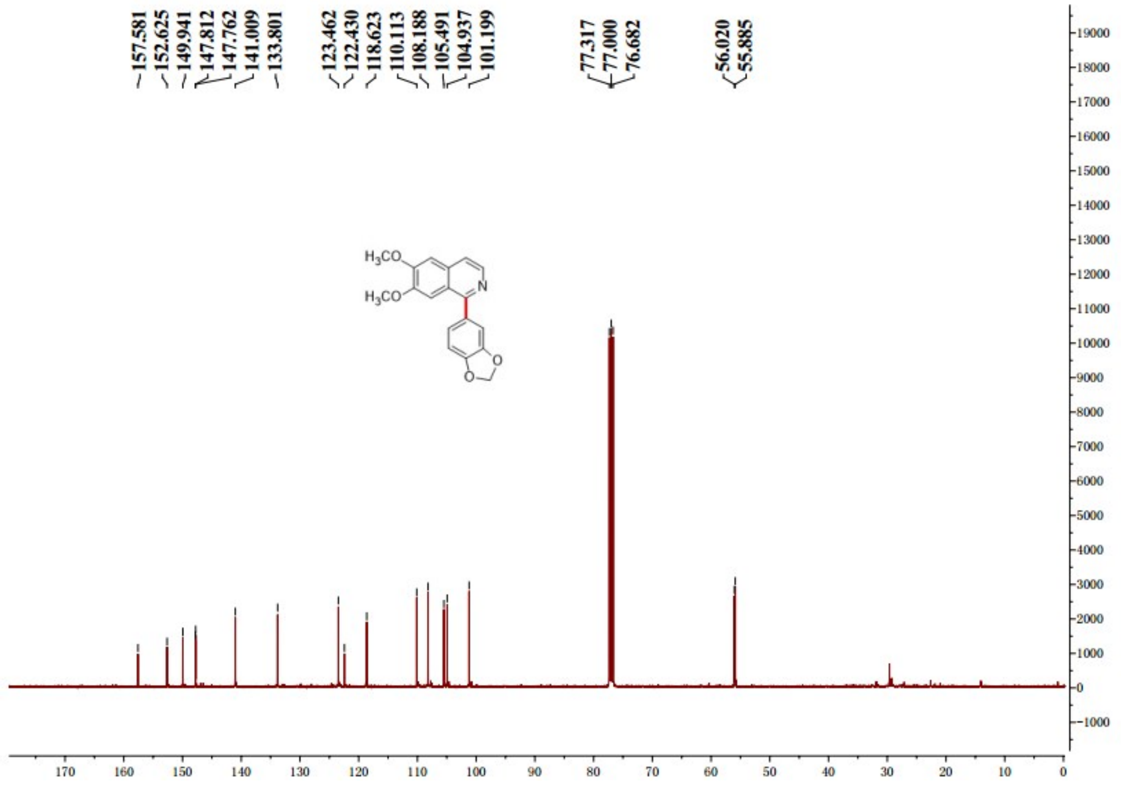
157.581
152.625
149.941
147.812
147.762
141.009
133.801
123.462
122.430
118.623
110.113
108.188
105.491
104.937
101.199
77.317
77.000
76.682
56.020
55.885
H3CO
H3CO
N
O
O

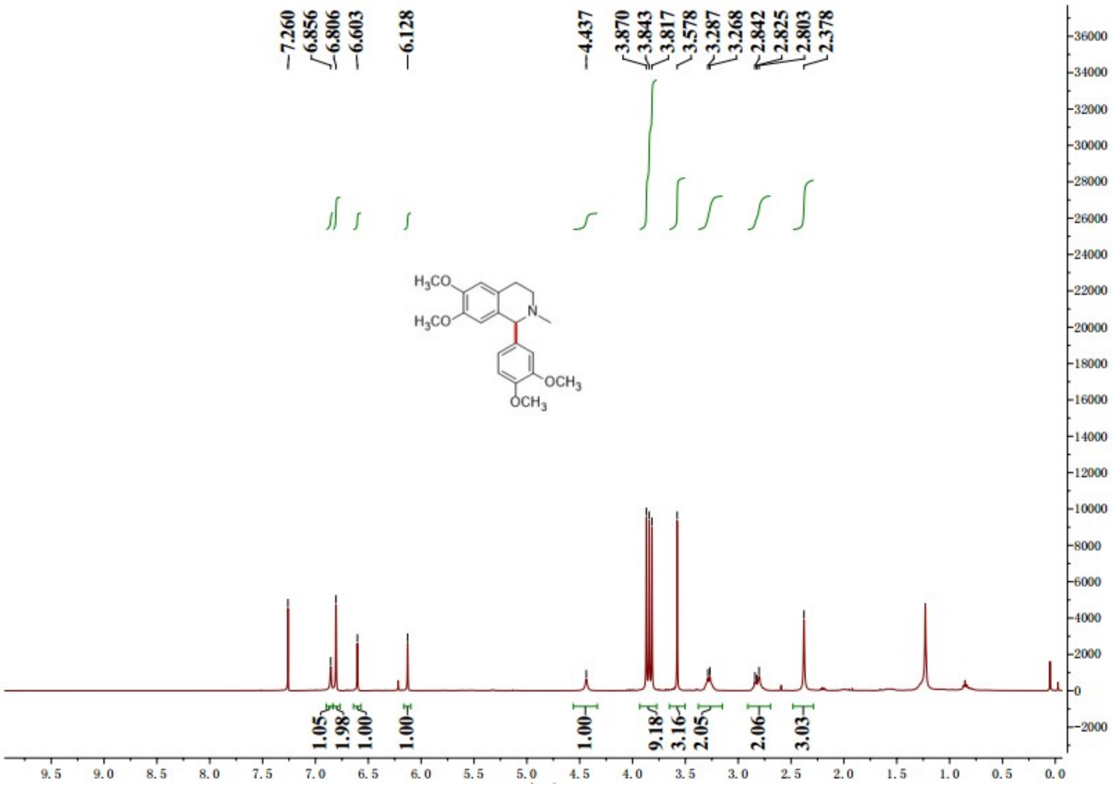

7.260
6.856
6.806
6.603
6.128
4.437
3.870
3.843
3.817
3.578
3.287
3.268
2.842
2.825
2.803
2.378
H3CO
H3CO
N
OCH3
OCH3
1.05
1.98
1.00
1.00
1.00
9.18
3.16
2.05
2.06
3.03

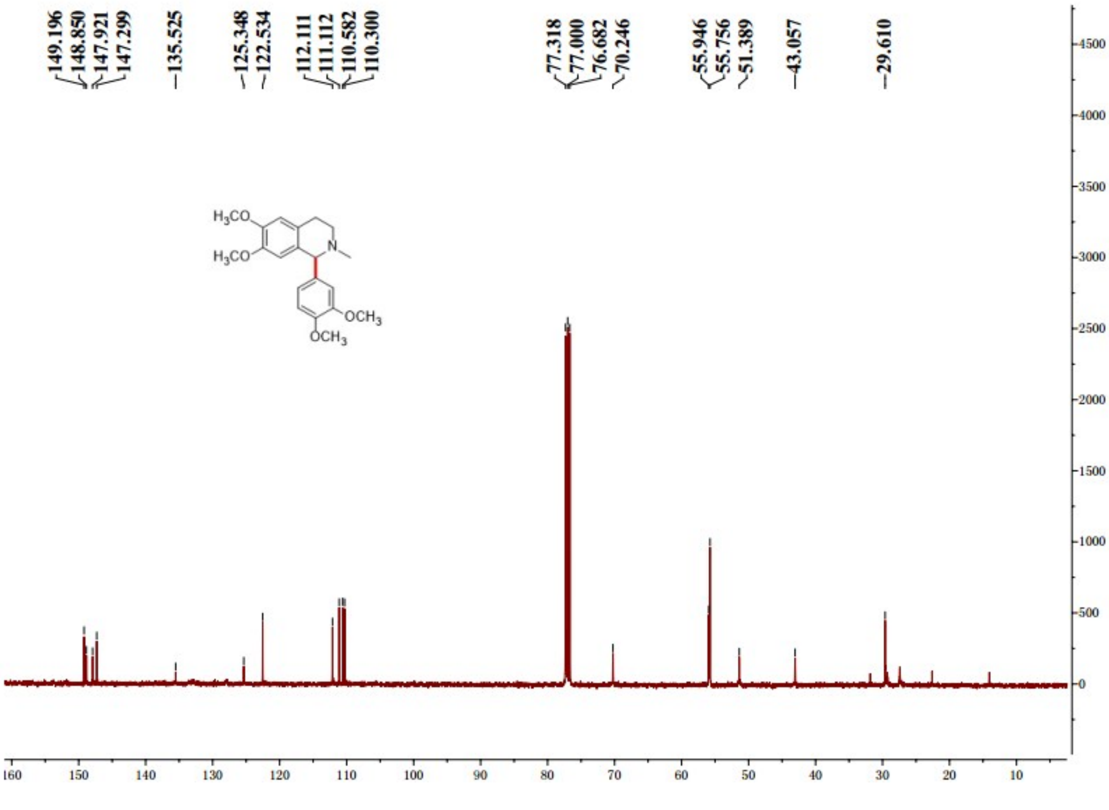

149.196
148.850
147.921
147.299
135.525
125.348
122.534
112.111
111.112
110.582
110.300
77.318
77.000
76.682
70.246
55.946
55.756
51.389
43.057
29.610
H3CO
H3CO
N
OCH3
OCH3

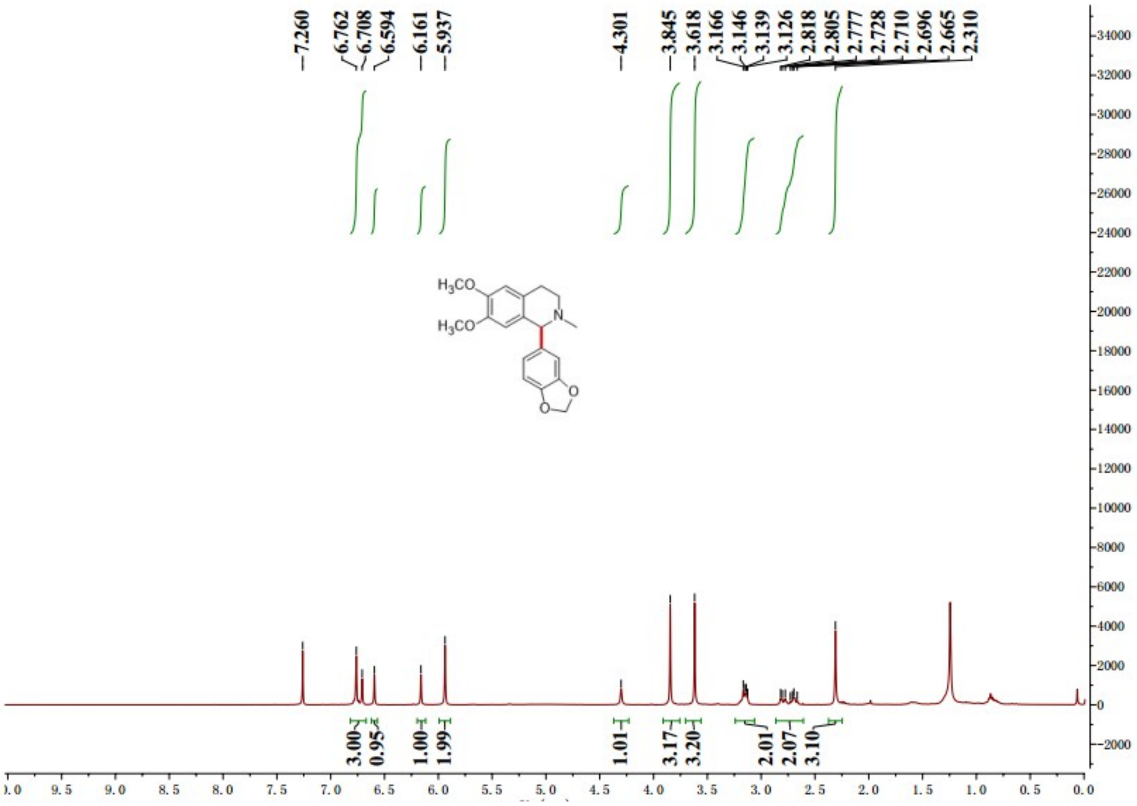

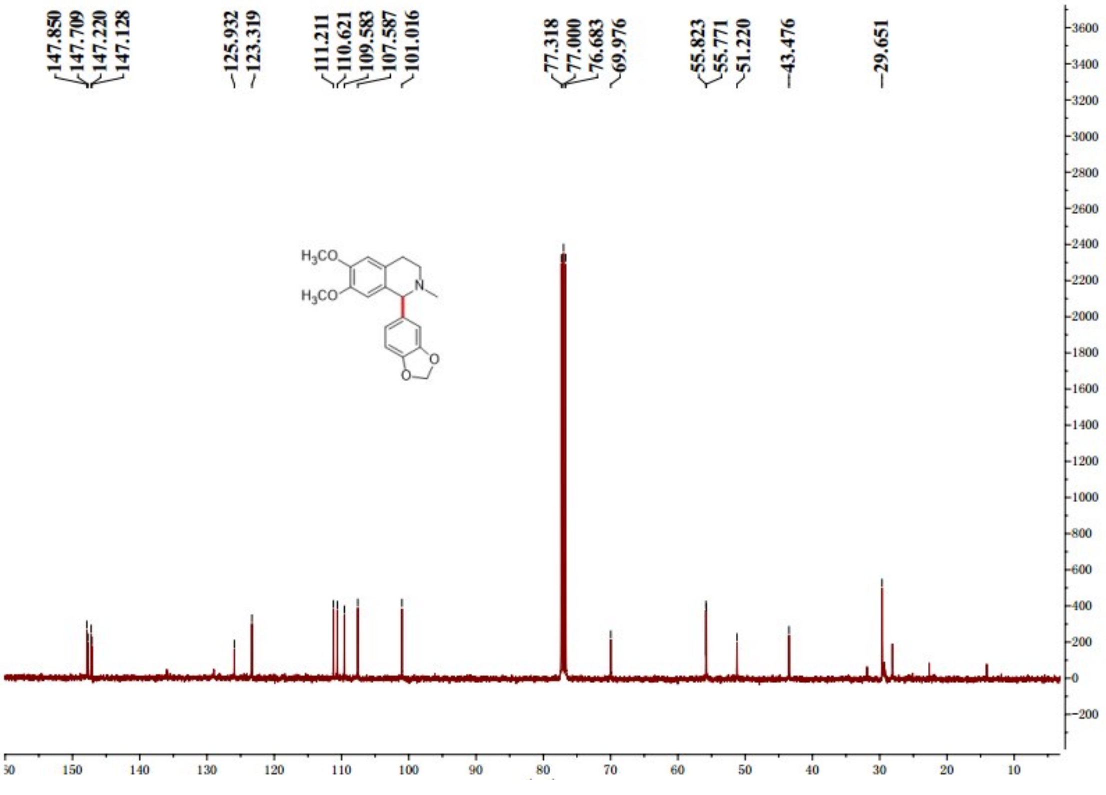

Supplement: Supplementary file 1 [file SC-010-C9SC00046A-s001.pdf]
